# The landscape in the gut microbiome of long-lived families reveals new insights on longevity and aging – relevant neural and immune function

**DOI:** 10.1080/19490976.2022.2107288

**Published:** 2022-08-08

**Authors:** Jingjing Wang, Jinlong Qie, Danrong Zhu, Xuemei Zhang, Qingqing Zhang, Yuyu Xu, Yipeng Wang, Kai Mi, Yang Pei, Yang Liu, Guozhong Ji, Xingyin Liu

**Affiliations:** a Department of Gastroenterology, Key Laboratory of Holistic Integrative Enterology, The Second Affiliated Hospital of Nanjing Medical University, Jiangsu, China; b Department of Pathogen Biology-Microbiology division, State Key Laboratory of Reproductive Medicine, Key Laboratory of Pathogen of Jiangsu Province, Key Laboratory of Human Functional Genomics of Jiangsu Province, Center of Global Health, Nanjing Medical University, Jiangsu, China; c The Affiliated Suzhou Hospital of Nanjing Medical University, Suzhou Municipal Hospital, Gusu School, Nanjing Medical University, Suzhou, China; d Jiangsu KeyLaboratory of Cancer Biomarkers, Prevention and Treatment, Collaborative Innovation Center for Cancer Personalized Medicine, Nanjing Medical University, Jiangsu, China

**Keywords:** Centenarians, longevity, gut microbiome, odoribacter splanchnicus, bifidobacterium pseudocatenulatum

## Abstract

Human longevity has a strong familial and genetic component. Dynamic characteristics of the gut microbiome during aging associated with longevity, neural, and immune function remained unknown. Here, we aim to reveal the synergistic changes in gut microbiome associated with decline in neural and immune system with aging and further obtain insights into the establishment of microbiome homeostasis that can benefit human longevity. Based on 16S rRNA and metagenomics sequencing data for 32 longevity families including three generations, centenarians, elderly, and young groups, we found centenarians showed increased diversity of gut microbiota, severely damaged connection among bacteria, depleted in microbial-associated essential amino acid function, and increased abundance of anti-inflammatory bacteria in comparison to young and elderly groups. Some potential probiotic species, such as *Desulfovibrio piger, Gordonibacter pamelaeae, Odoribacter splanchnicus, and Ruminococcaceae bacterium D5* were enriched with aging, which might possibly support health maintenance. The level of Amyloid-β (Aβ) and brain-derived neurotrophic factor (BDNF) related to neural function showed increased and decreased with aging, respectively. The elevated level of inflammatory factors was observed in centenarians compared with young and elderly groups. The enriched *Bacteroides fragilis* in centenarians might promote longevity through up-regulating anti-inflammatory factor IL-10 expression to mediate the critical balance between health and disease. Impressively, the associated analysis for gut microbiota with the level of Aβ, BDNF, and inflammatory factors suggests *Bifidobacterium pseudocatenulatum* could be a particularly beneficial bacteria in the improvement of impaired neural and immune function. Our results provide a rationale for targeting the gut microbiome in future clinical applications of aging-related diseases and extending life span.

**Abbreviations**: *16S rRNA*: 16S ribosomal RNA; *MAGs*: Metagenome-assembled genomes; *ASVs*: Amplicon sequence variants; *DNA*: Deoxyribonucleic acid; *FDR*: False discovery rate: *KEGG*: Kyoto Encyclopedia of Genes and Genomes; *PCoA*: Principal coordinates analysis; *PCR*: Polymerase chain reaction; *PICRUSt*: Phylogenetic Investigation of Communities by Reconstruction of Unobserved States; *Aβ*: Amyloid-β (Aβ); *BDNF*: Brain-derived neurotrophic factor

## Introduction

Gut microbiota and its metabolites play important roles in host’s physiological activities, such as digestion, metabolism, and immunity^1^. In a few regions, centenarians showed specific characteristics of the gut microbiome associated with longevity.^2–5^ For example, the diversity and abundance of gut microbiota in Italian centenarians were significantly higher than in younger group.^6^ Several studies from different countries and regions reported that healthy centenarians showed higher abundance of short-chain fatty acid-producing bacteria.^6,7^ Short-chain fatty acids provide the main energy source for colonic epithelial cells and have anti-inflammatory properties.^8^ The enrichment of these short-chain fatty acid-producing bacteria in centenarians may improve centenarians’ immune function. Consistent with this, a probiotic, *Lactobacillus fermentum PL9988* was found to have a significant advantage in enhancing host immunity, and that has anti-inflammatory and antioxidants effects were most abundant in healthy centenarians in Koran.^9^ Taken together, these studies suggested that increased microbiota diversity and an elevated abundance of anti-inflammatory bacteria may partially contribute to healthy longevity.

Emerging evidences suggest that gut bacteria collaborate with their animal hosts to modulate the immune, metabolic and nervous systems through dynamic bidirectional communication along the “gut-brain axis”.^10^ Changes in the microbial community have been linked to neurological disorders, such as autism, Alzheimer’s disease, and Parkinson’s disease.^11^ Nevertheless, little evidence has been reported on whether microbial community characteristics in long-lived families are associated with changes in immune factors and cognitive function.

Recently, using two disease models of aging in *Caenorhabditis elegans*, reproductive tumor and Alzheimer’s disease, Meng Wang’s group conducted genome-wide screening of *Escherichia coli* mutants and systematically revealed the relationship between individual genes of *E. coli* and host lifespan. The authors found that more than ten probiotic bacterial mutants can not only prolong the lifespan of the host body but also fight against these two different aging-related diseases, and inhibit tumor cell invasion and expansion and amyloid deposition in the host.^12^ In agreement with this study, by following up the gut microbiome, 51 human phenotypes and plasma levels of 1,183 metabolites of 338 individuals for 4 years, Chen et al. characterized microbial stability and variation in relation to host physiology.^13^ However, whether microbial gene variations serve as microbial signatures of longevity and suggest potential targets to promote healthy aging remains unknown.

At present, the United Nations has awarded ten towns in China for longevity, but only the bacterial community characteristics of the long-lived people in Xinjiang have been primarily reported in Chinese.^14^ Several studies about the gut microbiota of longevity people lack detailed analysis on gut microbiota composition, gene variation of bacterial strains, and association with host healthy status.^6,7^ The samples showed few genetic homogeneity and lifestyle consistency. Rugao City of Jiangsu Province is one of the ten longevity regions, but the characteristics of gut microbiota associated with longevity have not been investigated in this area. To determine globally gut microbiome signatures associated with longevity families, we collected feces and blood samples from 32 families including centenarians, elderly and young people in Baipu Village. Based on 16S rRNA sequencing and metagenomics data for the cohorts, we first outlined the landscapes of the gut ecosystem in long-lived families in terms of the diversity, microbiome relationship, altered bacteria abundance trajectory along aging, single nucleotide polymorphisms, and function landscape in the gut microbiome along aging. Combined the level of immune inflammatory factors, Amyloid-β (Aβ), brain-derived neurotrophic factor (BDNF) in blood serum across young, elderly and centenarians, we characterized the synergistic changes in gut microbiome associated with decline in neural and immune system with aging.

## Results

### Information of the cohort

A total of 32 centenarians (G1), 30 elderly people (G2) and 11 young people (G3) were recruited. Characteristics of study participants for 16S rRNA analysis were shown in Table S1A. Statistically significant differences in age, sex, body mass index (BMI), and smoking status were found among three groups (Table S1A). No significant differences for food preference and diseases among three groups were observed in 16S rRNA sequencing (Table S1A). Characteristics of study participants for metagenomics sequencing were listed in table S1B. No significant differences for food Preference, BMI, smoking, and disease status among three groups were observed in metagenomics samples (Table S1B). Elderly group in both 16S rRNA and metagenomics analysis showed significantly increased alcohol consumption in comparison to young and centenarian groups (Table S1A and Table S1B). The detailed characteristics, including the diseases, food preference, smoking status, taking probiotics or not and alcohol consumption for all samples are shown in Table S2.

### Centenarians (G1) displayed alterations in gut microbiota compared with elderly people (G2) and young people (G3) based on 16S rRNA data

**Figure 1.**
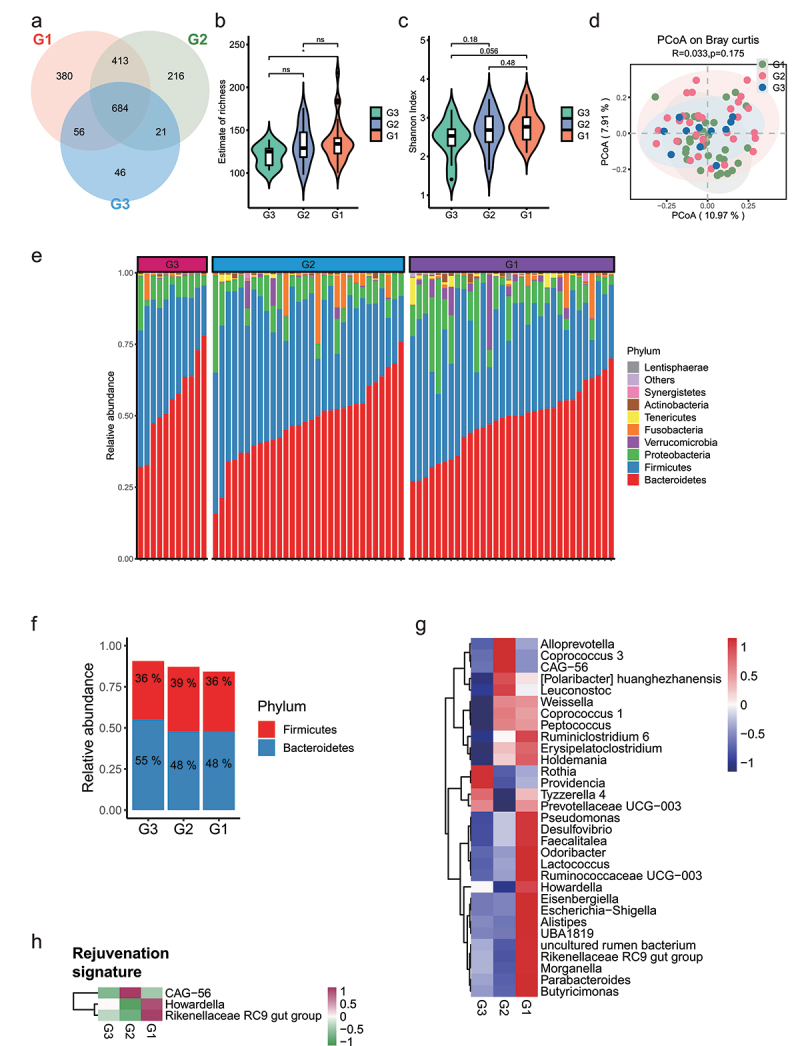
**Comparison of the gut microbiota between G3, G2 and G1 groups according to the 16S rRNA data**. (a). Venn diagram of the observed ASVs in G3 (n = 11, aged 16 to 52 years [mean age, 37.1 years]), G2 (n = 30, aged 52 to 83 years [mean age, 71 years]) and G1 (n = 32, aged 100 to 108 years, [mean age, 101.7 years]) groups. (b-c). Alpha diversity indices of genera between three groups according to Estimate of richness (b), Shannon index (c). *p < .05; Wilcoxon rank-sum test. ns, not significant. (d). Principal coordinate analysis (PCoA) of the microbiota based on the Bray-Curtis distance between three groups. ANOSIM, R = 0.033, p = .175. (e). Bar plots showing the relative abundance of microbiota of three groups of individuals at phylum level, with different colors correspond to different phyla. (f). Bar plots showing the relative abundance of Firmicutes and Bacteroidetes in three groups. (g). Heat map showing the relative abundance of three groups of individuals at genus level. Only the significantly different genera between any two groups were displayed. Based on the different genera obtained by Wilcoxon rank sum test (Benjamini–Hochberg-corrected P value < .05), 10 samples were randomly returned from each group to form self-help samples. The average difference statistics of relative genus abundance were calculated and repeated 1000 times to determine whether the difference was significant according to 90% confidence interval. (h). Changes in the relative abundance of genera between G3, G2, and G1 subjects grouped according to the rejuvenation signature.

After selecting the effective reads, each fecal sample produced 80,268 to 99,917 effective reads. Rarefaction curve analysis reached a stable level, indicating that the sequencing depth had covered new rare phylotypes and most of the diversity (Figure S1A). As shown in Figure 1a, the Venn diagram displayed 380 unique amplicon sequence variants (ASVs) in the G1 group, 216 unique ASVs in the G2 group, and 46 unique ASVs in the G3 group; furthermore, 684 ASVs were shared by the three groups. Estimates of richness and Shannon index were calculated to quantify bacterial alpha diversity. No statistical difference between G1 and G2 was found, but the G3 group showed significantly lower estimate of richness than the G1 group (Figure 1b, c). A principal coordinates analysis (PCoA) analysis showed three groups displaying similar microbiota composition (Figure 1d and Figure. S1B). The top 10 phyla in the relative abundance of gut microbiota were shown as histograms (Figure 1e). At the phylum level, we observed a moderate significant enrichment of *Verrucomicrobia* in G1 compared with G3 (Figure 1e and Table S3). As the Firmicutes/Bacteroidetes ratio is often used as an index for the structure of gut microbiota, we compared the ratio in three groups. The relative abundance of Firmicutes was higher in subjects of elderly than in the young and centenarian group, whereas the proportion of Bacteroidetes was lower in elderly group, accordingly, we found a higher Firmicutes/Bacteroidetes ratio in the elderly than in the young and centenarians (Figure 1f).

To reconstruct the microbiota changed trajectory along aging, the relative abundances of the genera with significant differences between any two groups were displayed in Figure 1g (Table S4). The commensal genera *Odoribacter, Ruminococcaceae UCG−003, and Desulfovibrio* enriched with aging (Figure 1g). Some opportunistic bacteria, e.g., *Parabacteroides, Butyricimonas*, and *Alistipes* only enriched in centenarian group. According to the previous report, rejuvenation signature included genera whose abundance was similar in younger and centenarian, but distinct from the elderly group.^15^ *Howardella* and *Rikenellaceae RC9* genera were included in rejuvenation signature and specifically enriched in G1 group (Figure 1h), and the absolute abundance of these two genera was also found to be increased in G1 group (Figure S1C), indicating these genera may have potential role in preservation of youth or reversing aging. Centenarian signature included centenarian-specific genera whose abundance was similar in G3 and G2 groups but distinct from the G1 group per previous definition.^15^ We found the absolute abundance of *Desulfovibrio* and *Pseudomonas* genera were included in the centenarian signature and specifically enriched in G1 group (Figure. S1D). Notably, *Parabacteroides* and *Odoribacter*, which were enriched in the G1 group, are known to resist inflammation and contribute to gut health.^16,17^ Functional analysis showed that the activities of vitamin B6 metabolism and thiamine metabolism were lower in G1, whereas retinol metabolism and tryptophan metabolism were higher in G1 group (Figure. S1E, F).

**Figure 2.**
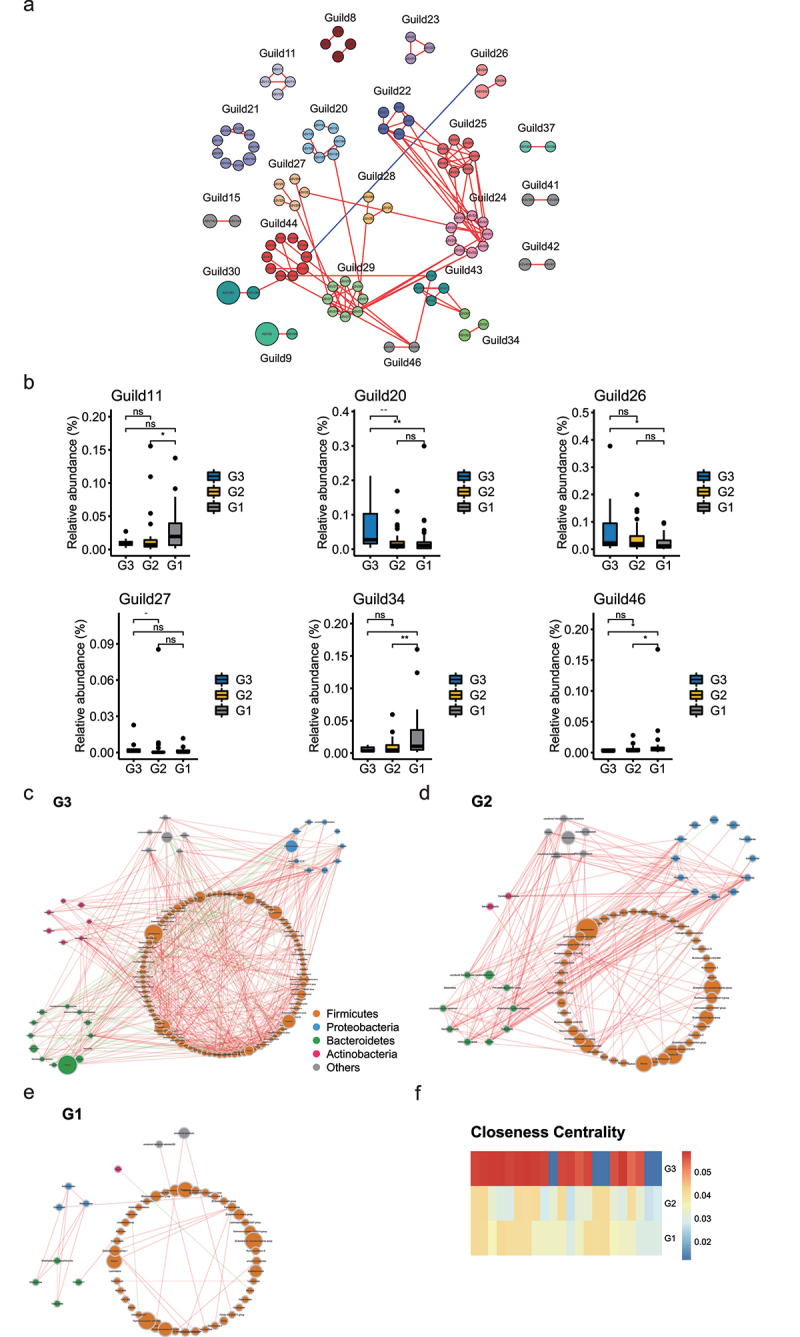
**Bacterial correlation analysis based on relative abundance between G3, G2 and G1 groups**. (a). Co-abundance groups interaction network. Node size represents the average abundance of each ASV. Lines between nodes represent correlations of each other, with the line width representing the correlation magnitude. The red ones represent positive correlations, and the blue ones represent negative correlations. Only lines whose absolute value of correlation coefficient greater than 0.70 were drawn, and unconnected nodes were omitted. (b). Group-level abundance differentiation of guilds. Data are visualized by boxplot. Box represents the interquartile range. The line inside the box represents the median. And whiskers denote the minimum and maximum value. *p < .05; **p < .01; Wilcoxon rank-sum test. ns, not significant. (c-e). Network plots describing co-occurrence of bacterial genera in the gut microbiota of G3, G2, and G1 group based on the Spearman correlation algorithms (r ≥ 0.7, FDR < 0.05). Bacterial genera with at least 0.1% of relative abundance in at least 20% of the samples in each group were plotted. Each node presents a bacterial genus. The node size indicates the relative abundance of each genus per group, and the density of the dashed line represents the Spearman coefficient. Red links stand for positive interactions between nodes, and green links stand for negative interactions. (f). Discrepancies of the genera co-occurrence networks between three groups based on the 16S rRNA data. Centralities (rank of the closeness) and discrepancies of nodes in G3, G2 and G1 co-occurrence networks were counted, respectively.

Given that bacteria act as interdependent functional groups (guilds) in the gut ecosystem,^18,19^ synergistic changes of bacteria in guilds may help identify bacteria that play an important role in long-lived families. Hence, we further constructed a co-abundance network at the ASV level. A total of 462 ASVs were shared by at least 20% of the samples and clustered into 46 guilds based on SparCC correlation coefficients (Table S5). Only 21 guilds with ASVs whose absolute value of correlation coefficient was greater than 0.70 were shown in Figure 2a and Table S6. According to the Kruskal–Wallis test, group level abundance analysis showed that six guilds showed significant differences among the three groups. Notably, guild2 showed decreased abundance in G1 compared with G3, and the guild contained the genera, *Dialister, Parasutterella*, and *Acidaminococcus* which showed decreased abundance in G1 group. In addition, guild34 and guild46 were enriched in G1 relative to G3, and the two guilds contained the genera, *Alistipes, Ruminococcaceae UCG-005*, and *Faecalitalea* which showed elevated abundance in G1 group (Figure 2b). To describe the potential relationships occurring among bacteria within the gut microbial communities, we further constructed co-occurrence networks of genera from each group based on Spearman correlations. G3, G2, and G1 groups mainly featured three co-occurrence networks with scattered genera from four primary phyla (Firmicutes, Bacteroidetes, Proteobacteria, and Actinobacteria) (Figure 2c-e). The correlation between the genera in G1 was distinctly decreased compared with that in G2 and G3. To quantify such differences, we counted the number of edges (connections) and the centrality of nodes (genera) in the two microbial networks. As shown in Figure S1G, the three groups shared four overlapped edges, whereas 36, 172, and 500 of the edges were specific to the G1, G2 and G3, respectively. The closeness and eigenvector of the shared genera in G3 were also quite different from those in G2 and G1 (Figure 2f). In general, the above analysis suggested that the microbial ecosystem in G1 was quite different from that in G2 and G3, and that the microbial relationship tended to be homogeneous with aging.

### Metagenomic sequencing revealed significant differences in terms of gut microbiota composition and gene function among the three groups

**Figure 3.**
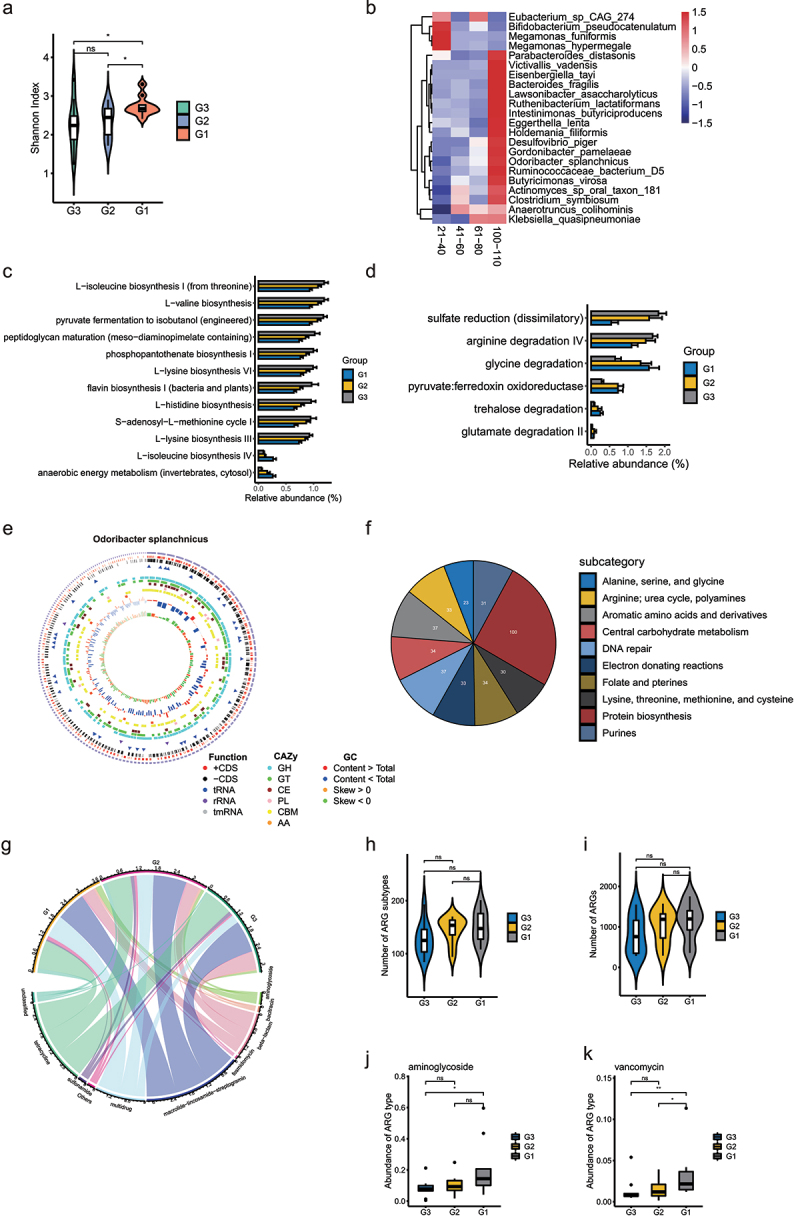
**Comparison of the gut microbiota and gene functions between G3, G2, and G1 based on the metagenomic sequencing data**. (a). Alpha diversity indices of species between three groups according to Shannon index. *p < .05; Wilcoxon rank-sum test. ns, not significant. (b). Heat map showing the relative abundance of individuals at different age stages at species level. Only the significantly differential genera were shown between any two groups in G3, G2, and G1 groups were displayed. Benjamini–Hochberg-corrected P value < .05; Wilcoxon rank-sum test. (c). Bar plot showing the relative abundance difference in MetaCyc pathways between three groups. Only the significantly differential pathways were shown between G3 and G1 individuals. (d). Bar plot showing the relative abundance difference in GBMs between three groups. Only the significantly different GBMs were shown between G3 and G1 individuals. (e). Genomic features of *Odoribacter splanchnicus*. The 3,788,833 bp genome containing 182 contigs, a N50 length of 30400 bp, a GC content of 43.5%. From the outer circle to the inner, it represents the length of contigs, coding sequences (CDS) on forward and reverse strands, tRNA, rRNA, tmRNA, CAZy annotation, GC content and GC skew curve, respectively. (f) Pie charts showing the function of *Odoribacter splanchnicus* genome at subcategory level based on RAST annotation. (g). The overview of ARGs in three groups. (h). Comparison of the number of ARGs between three groups. (i). Comparison of the number of ARG subtypes between three groups. (j-k). Abundance of significantly different ARG types between G3 and G1 individuals.

To further explore the role of bacterial species and function in long-lived families, we performed metagenomics sequencing for the three groups. The Shannon index was calculated to quantify the alpha diversity among the three groups at the species level. As shown in Figure 3a, the alpha diversity was significantly higher in G1 than in G2 and G3, but no difference was found between the G2 and G3. According to the dynamic changes across different age stages (Figure 3b and Table S7), the enrichment prevalence of health associated bacteria, e.g., *Gordonibacter pamelaeae, Odoribacter splanchnicus, Ruthenibacterium lactatiformans, Intestinimonas butyriciproducens, Butyricimonas virosa*, and *Bacteroides fragilis* are increased in centenarians. Some potential probiotic bacteria, e.g., *Bifidobacterium pseudocatenulatum, Megamonas funiformis* and *Megamonas hypermegale* are decreased during aging. Bacterial functional analysis based on MetaCyc pathways and gut-brain modules (GBMs) database were shown in Figure 3c-d (Table S8). Impressively, we observed significantly decreased enrichment of the L− lysine biosynthesis VI, flavin biosynthesis I and arginine degradation IV pathways in G1 compared with G2 and G3 group, as well as increased enrichment anaerobic energy metabolism and glycine degradation.

**Figure 4.**
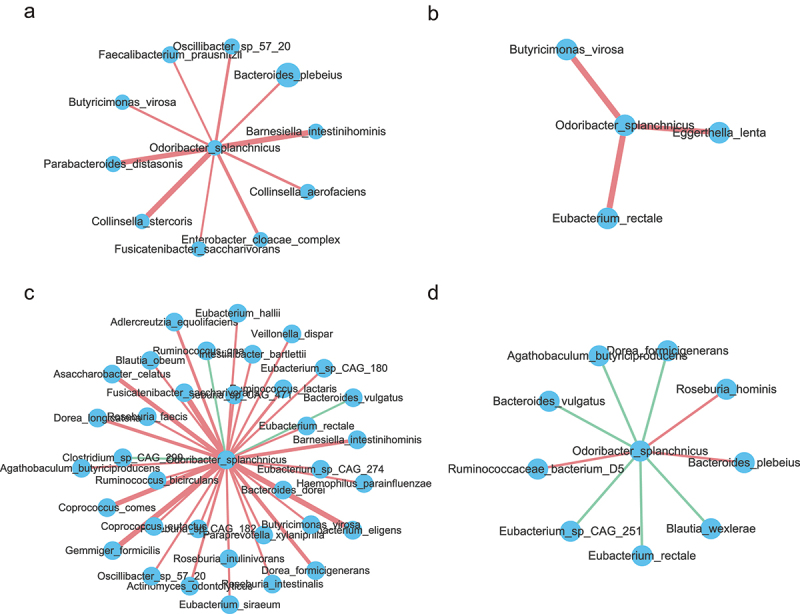
***Odoribacter_splanchnicus* correlations with other species at different age stages**. (a-d). Correlations of *Odoribacter splanchnicus* with other species in 21–40, 41–60, 61–80 and 100–110 y old respectively. Spearman correlation algorithms (r ≥ 0.5,FDR<0.05). Red links stand for positive interactions between nodes, and green links stand for negative interactions.

To identify the function of specific strain linked to longevity, we performed an analysis of individual draft genomes (bins) through MetaWRAP.^20^ A total of 261 acceptable bins (completion greater than 70% and contamination less than 10%) were generated for further analysis (Figure S2a,b and Table S9). A phylogenetic tree of the 261 bins is presented in Figure S2C. The tree is dominated by large numbers of genomes from the Firmicutes and Bacteroidetes phyla. Given that the relative abundance of *O. splanchnicus* was significantly increased in the centenarians in our study and *O. splanchnicus* strains have been reported to play an important role in centenarians by participating in specific bile acid metabolic activity,^15^ we reassembled the strain genome. As shown in Figure 3e, the *O. splanchnicus* genome consists of a single circular chromosome of 3,788,833 bp containing 182 contigs, an N50 length of 304,00 bp, and a GC content of 43.5%. The genome was near complete (96.77%) and had low evidence of possible contamination (1.08%). The genome encoded a total of 3,622 genes, of which 3,575 genes (98.7%) were protein-coding and 47 genes were RNA-coding. Functional analysis of the *O. splanchnicus* genome showed that it was enriched in the essential amino acid metabolism, protein biosynthesis pathway, and ribosome LSU bacterial pathway (Figure 3f and Figure S2D). Impressively, the relationship between *O. splanchnicus* and other microbes in centenarians and young showed a simpler and similar microbial network, however, the elderly group showed a substantially more complicated microbial network (Figure 4a-d), which implies that *O. splanchnicus* might play an important role in maintaining younger-like equilibrium of the gut ecosystem in centenarians.

Antibiotics overuse is a global public health problem, with the danger of accelerating the spread of drug-resistant bacteria.^21^ We analyzed the antibiotic resistance genes (ARGs) of longevity family members, as shown in Figure 3g-i, no difference in the total ARGs and their subtypes was found, but G1 showed decreased aminoglycosides and vancomycin genes compared with G3 (Figure 3j-k), which implicated that the gut microbiota of centenarians was relatively less contaminated with antibiotics.

### Gut microbiota–based prediction of three groups

**Figure 5.**
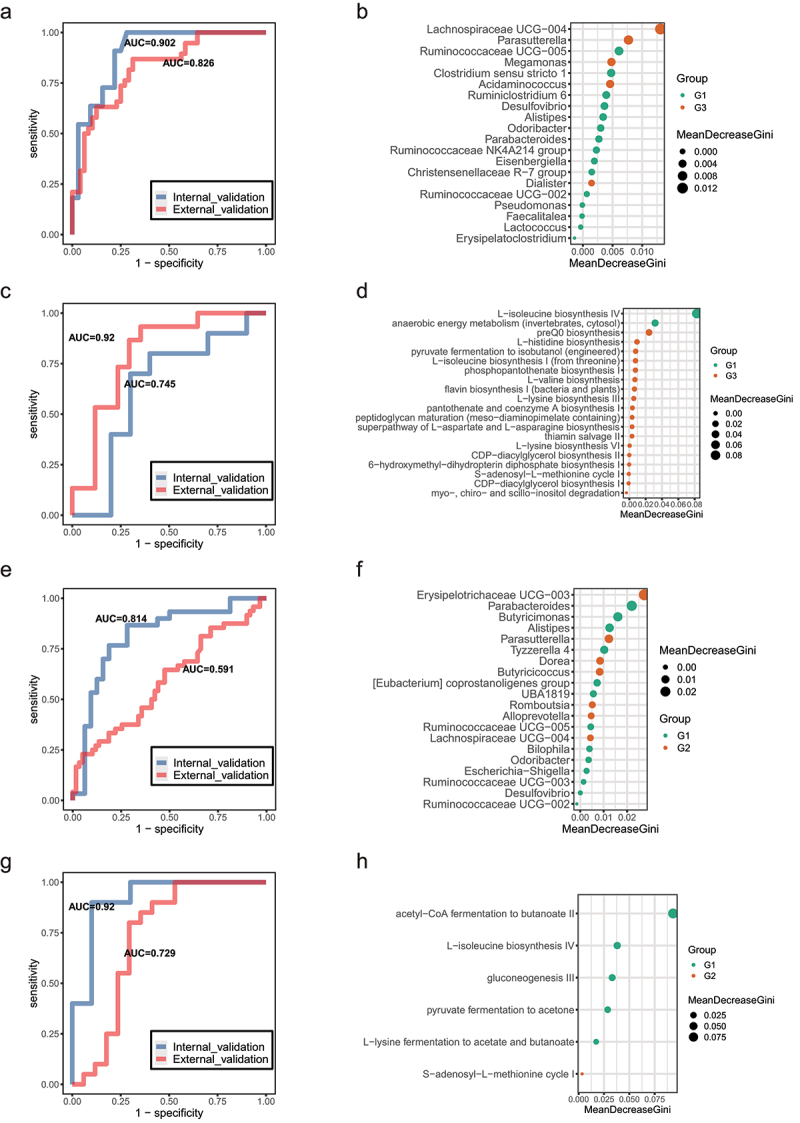
**Classifiers for distinguishing G3 from G1 group and G2 from G1 group**. (a). Receiver operating characteristic (ROC) curves for the G3 and G1 group were assessed by R Random Forest package. Only the top 20 significantly different genera between G3 and G1 individuals was used as predictors based on the 16S rRNA data. Another Chinese dataset as external validation set. Chinese young (n = 38) and centenarians (n = 48) from the SRA database under accession no. SRP107602. b. In order of importance, the genera used for predicting G3 and G1 groups were listed. (c). ROC curves for the G3 and G1 groups were assessed by R Random Forest package. Only the top 20 significantly different MetaCyc pathways between G3 and G1 individuals are used as predictors based on the metagenomic sequencing data. Sardinian dataset as external validation set. Sardinian young (n = 17) and centenarians (n = 19) from the European Nucleotide Archive (accession number PRJEB25514). (d). The MetaCyc pathways were ranked in order of importance for predicting G3 and G1 groups. (e). ROC curves for the G2 and G1 groups assessed by R Random Forest package. Using only the top 20 significantly different genera between G3 and G1 individuals as predictors based on the 16S rRNA data. Another Chinese dataset as external validation set. (f). Genera used to predict G2 and G1 groups were ranked in order of importance. (g). ROC curves for the G2 and G1 groups were assessed by R Random Forest package. Using only the top 20 significantly different MetaCyc pathways between G3 and G1 individuals as predictors based on the metagenomic sequencing data. Sardinian dataset as external validation set. (h). The MetaCyc pathways were ranked in order of importance for predicting G3 and G1 groups.

Next, to test whether potential diagnostic biomarkers can be used to predict age groups, we constructed a random forest model based on significant differential genera and MetaCyc pathways. The top 20 significantly differential genera between the G1 and G3 individuals were used as biomarkers to distinguish centenarians from young adults (Figure 5a, b). The area under the curve (AUC) displayed good predictive power for longevity in our set (AUC = 0.902). Another Chinese dataset^22^ as an external validation set also showed great prediction ability (AUC = 0.826). Similarly, the top 20 significantly different MetaCyc pathways between G1 and G3 showed great discriminatory power to distinguish centenarians from young adults (The current cohort, AUC = 0.92; Sardinian cohort,^5^ AUC = 0.745) (Figure 5c, d). Meanwhile, the constituted random forest model based on the differential genera between G1 and G2 cannot perform well discriminatory power to predict longevity status, but the discriminant ability based on differential MetaCyc function pathways (acetyl−CoA fermentation to butanoate II, L− isoleucine biosynthesis IV, gluconeogenesis III, pyruvate fermentation to acetone, L− lysine fermentation to acetate and butanoate, and S− adenosyl−L− methionine cycle) still showed great prediction ability (the current cohort, AUC = 0.92; Sardinian cohort, AUC = 0.729; Figure 5e-h), which implicating different bacteria may evolve similar metabolic function to involve in aging process.

### Enriched species in centenarians associated with decline of immune system, amino acid metabolic activity, and cognitive function

**Figure 6.**
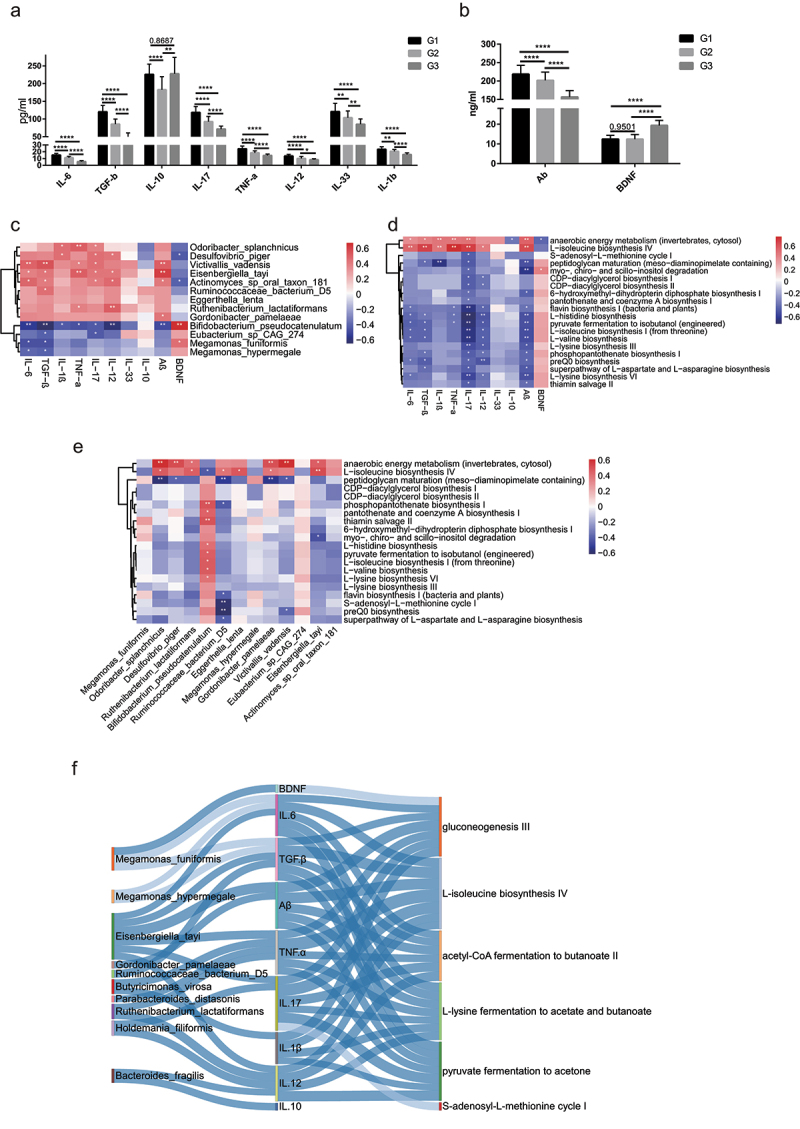
**Associations between species, immune cytokines and pathways**. (a-b). Analysis of the level of eight immune cytokines, Aβ, and BDNF in G3, G2 and G1 groups. Serum of all subjects was collected and detected by ELISA. The differences were calculated by t-test (*p < .05, **P < 0 .01, ***P < .005, ****P < .001). (c). Heatmap of associations between species and cytokines. (d). Heatmap of associations between MetaCyc pathways and cytokines. (e). Heatmap of associations between species and MetaCyc pathways. (f). Correlation between species, serum cytokines and MetaCyc pathways based on the Spearman correlation algorithms according to the metagenomic sequencing data (Benjamini–Hochberg-corrected P value < .05). Only the top 20 significantly different species and pathways between G2 and G1 individuals were used to calculate the correlation. In (c-e), only the top 20 significantly different species and MetaCyc pathways between G3 and G1 individuals were used to calculate correlation according to the metagenomic sequencing data. Spearman correlation, *p-value<0.05, **p-value<0.01.

The gut microbiota is increasingly recognized as an important regulator of host immunity and brain health.^11^ The preservation of host-microbes homeostasis can counteract inflammation, intestinal permeability, and decline in bone and cognitive health.^2^ To investigate the association between gut microbiota and inflammatory factors in longevity families, we first analyzed the expression levels of inflammatory cytokines in the blood samples of the three age groups (Table S10). As shown in Figure 6a, we observed the expression of seven cytokines, including IL-6, TGF-β, IL-17, TNF-α, IL-12, IL-33, and IL-1β, showed the highest level in G1 and lowest in G3. However, the expression of anti-inflammatory cytokine, IL-10, was higher in G1 than in G2 and no significant difference between G1 and G3. Notably, an elevated IL-10 expression in centenarians was observed compared with young and elderly people.

Recent studies have shown that the development of neurons is closely related to the development of gut microbiota.^23^ BDNF may take part in regulating neuronal survival and synaptic plasticity to present anti-oxidant effects, suppressing ROS and protecting the mitochondria.^24^ Furthermore, Aβ is thought to be a marker of neurological degeneration in the brain.^25^ To assess the correction of cognitive function of long-lived people, we analyzed the expression of BDNF and Aβ in the blood of long-lived families. As shown in Figure 6b, the expression of Aβ gradually increased with age. Nevertheless, BDNF expression showed no significant changes between G1 and G2. The correlation analysis of the significantly different species and immune cytokines showed the abundance of *Bifidobacterium pseudocatenulatum* in G1 was negatively correlated with IL-6, TGF-β, IL-1β, IL-17, TNF-a, IL-12, and Aβ level and positively associated with BDNF level. Furthermore, we found that the enrichment of *O. splanchnicus, Desulfovibrio piger* and *Victivallis vadensis* in G1 was positively associated with IL-17, TNF-a and IL-12 levels, implicating that these species may be core bacteria that play essential roles in stimulating an increase in these immune factors with aging (Figure 6c). To explore the links among metabolic pathways, species, and immune cytokines, we calculated the correlation between two of them. Interestingly, we found that anaerobic energy metabolism and L− isoleucine biosynthesis IV were positively correlated with most immune factors and species, and the level of IL-17, TGF-β, IL-6, IL-12 and Aβ were associated with almost all metabolic pathways (Figure 6d, e), indicating alteration of gut microbiota with aging matches metabolic and immune variations. Most of significantly increased species in G1 relative to G2 showed positive correlation with essential amino acids fermentation pathway. Interestingly, the enriched *Bacteroides fragilis* in G1 had no correlation with all of metabolism activity (Figure 6f and Figure S2E), in contrast, it showed strong positively correlated with level of anti-inflammatory IL-10 (Figure 6f).

The above results further support that gut microbiota might play an essential role in decline of immune system, metabolic activity, and cognitive function during aging.

### Identification of stable species in long-lived families

**Figure 7.**
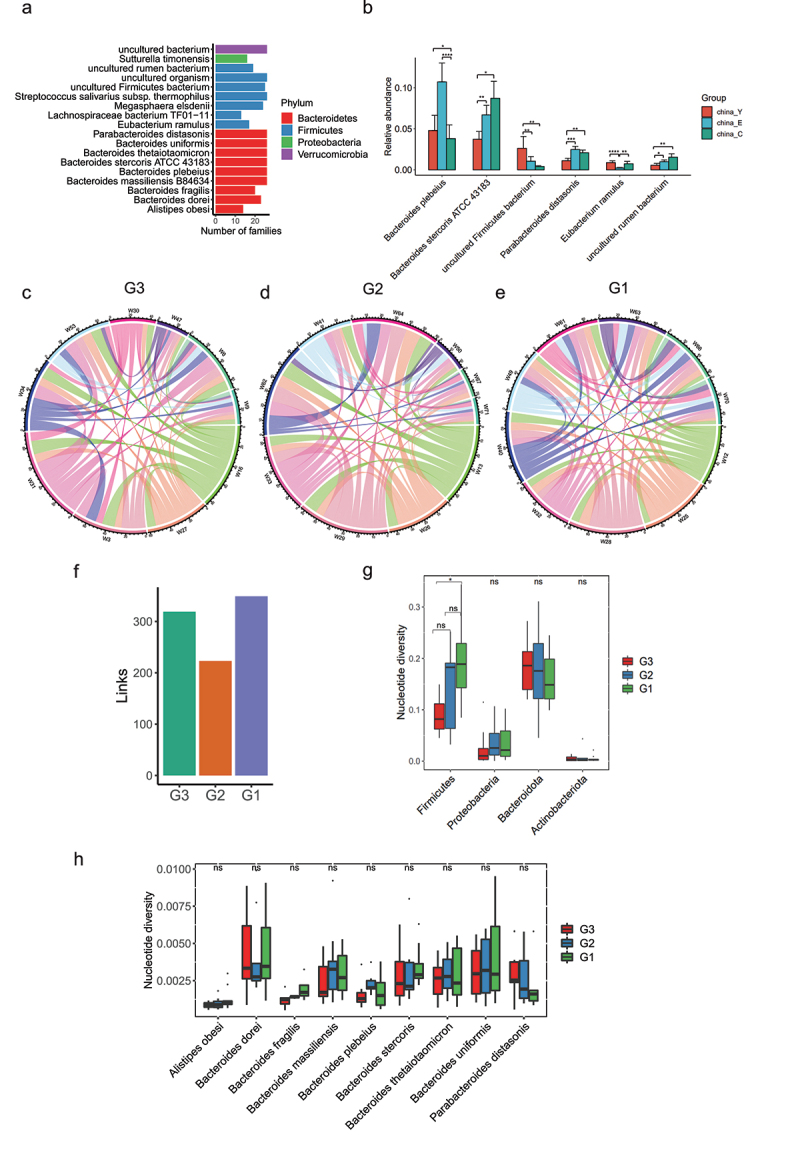
**Inheritance at the species level in long-lived families**. (a). Species that exist in at least half of long-lived families (n = 26) and show no significant differences in relative abundance between G3, G2 and G1 groups according to the 16S rRNA data. (b). Relative abundance of species presented in Figure 6a were shown in another Chinese dataset under accession no. SRP107602. Only the significantly differential species were shown between China_Y, China_E and China_C. **p < .01; *p < .05; Wilcoxon rank-sum test. ns, not significant. (c-e). A link was drawn for each strain shared between G3 individuals, G2 individuals and G1 individuals respectively according to the metagenomic sequencing data. (f). Enumeration of links drawn in Figure 6c-e. (g). Comparison of nucleotide diversity at the phylum level presented in Figure 2c. (h). Comparison of nucleotide diversity at the species level presented in Figure 6a. In (g-h), ***p < .001; **p < .01; *p < .05; Kruskal-Wallis with Dunn’s test. ns, not significant.

To further understand the importance of changes in microbial strains to phenotypic changes of host individuals, it is crucial to examine the stable and variable genetic components of gut microbiota over time.^13^As human longevity has a strong familial and genetic component,^26^ we then hypothesized that longevity families in Bapu village may have core and stable species that do not change with aging. . To investigate the presence of a stable inheritance of bacteria strains in longevity families, we compared the presence of species in 26 longevity families with at least two generations. Only species that exist in at least half of long-lived families and had no significant difference in relative abundance between G3, G2, and G1 groups were defined as stable genetic bacteria. A total of 18 stable species in long-lived families were identified in our study (Figure 7a), indicating that these stable species might be involved in the establishment of a new homeostasis with host, thus contributing to longevity. We further compared the relative abundance differences of the 18 species in unrelated young people, elderly people, and centenarians in another Chinese longevity dataset.^22^ We found that *Parabacteroides distasonis, Lachnospiraceae bacterium TF01 − 11, Eubacterium Ramulus, Bacteroides stercoris ATCC 43183* and *Bacteroides plebeius* showed significant differences among these unrelated three groups (Figure 7b). Impressively, we observed *Eubacterium Ramulus* in another Chinese longevity database showed rejuvenation signature whose abundance was similar in the young and centenarian group and higher than that in the elderly group. Through applying probiotic-based adjunctive treatment for ulcerative colitis (UC) patients, Chen et al. found the gut mucosal microbiota of the probiotic-receivers had significantly more beneficial bacteria *E. ramulus* and suggested that the bacteria might alleviate UC symptoms.^27^ As changes in single nucleotides can also lead to large changes in the function and pathogenic behavior of bacteria,^28^ we further analyzed the changes in bacterial strain level in the three generations of long-lived families, and thus found that more common bacterial strains were shared by individuals of G1 than individuals of G2 and G3 (Figure 7c-f). At the phylum level, the total nucleotide diversity in Firmicutes was higher in G1 and G2 than that of G3 (Figure 7g), implicating that the bacteria belonging to Firmicutes in the centenarians were more prone to producing bacteria genome mutations. Impressively, the stably inherited bacteria shown in Figure 6b such as *Parabacteroides distasonis, Bacteroides stercoris*, and *Bacteroides plebeius* showed no significant differences in nucleotide diversity among the three groups (Figure 7h). Taken together, the unique stable species in the long-lived families from Baipu village combined its’ validation in another longevity cohorts further suggested that long-lived families in Bapu village might have a specific unchanged microbiota contributing to their longevity.

## Discussion

Microbiota studies involving long-lived families across different age based on families is limited. Our study is unique in that the microbiota samples across three age stages were collected from long-lived families in the same village, and these families have almost similar diet structure, which reduced the interference brought by different environments and diets. We outlined the landscapes of the gut ecosystem in long-lived families from multiple levels including microbial composition, function, relationship profiles, the stable inherited bacteria, single nucleotide polymorphisms of microbial species, and specific bacteria associated with inflammatory factors, BDNF, and Aβ levels. Furthermore, we identified and independently validated bacteria and bacteria-associated function markers that could distinguish centenarians from young and elderly subjects with high accuracy. Taken together, the present microbiome study based on long-lived families provides preliminary insights on aging and longevity associated features including microbiota, neural and immune function.

Previous studies have highlighted mutualistic changes in the composition and diversity of the gut ecosystem of centenarians.^29^ For example, Biagi et al. reported the four age groups including semi-supercentenarians (105–109), adults (22–48), and elderly (66–78) showed a good separation on a principal coordinates analysis (PCoA),^2^ however, our present study showed three age groups have no significant change, indicating long-lived families might share much similar core microbiota composition independent on aging process. Santoro et al. reported that young adults and 70-y-olds maintain a highly similar diversity of gut microbiota, which markedly changes in centenarians.^30^ Consistent with this study, we also observed increased species α-diversity in centenarians compared with young adults and elderly people but no significant difference between the elderly and young adults. The result further supports the previous assumption that the diversity of gut microbiota seems to rest in a stable state from G3 to G2, while after 100 y of symbiotic association with the human host, it shows a profound, and possibly adaptive, remodeling.^30^ However, due to the present G2 lack of the age gap between 80 and 100 y of age, further analyses are still needed to fill in the age gap and dissect the age-related rebuilding of gut microbiota. In addition, we noted that centenarians showed signs of a typical senile connection between bacteria. Firmicutes are very successful competitors in the gut ecosystem.^31^ However, the co-occurrence microbial network around genera from Firmicutes in centenarians showed signs of a typical senile connection of bacteria compared with the young and elderly groups, indicating that the connection of other bacteria from Firmicutes in aging processes may be affected by the loss of bacteria gene function due to the variability caused by single nucleotides mutation in specific bacterial genes.

Kim et al. reported that *Akkermansia* and *Christensenellaceae* are well-known health-associated genera whose abundance increased in centenarians.^32^ Biagi et al. suggested that *Christensenellaceae* may represent a signature of the ecosystem of extremely longevity people.^2^ However, in the present study, no significant increase in *Akkermansia* and *Christensenellaceae* were observed in centenarians. Several butyrate produced bacteria (*Ruthenibacterium lactatiformans, Intestinimonas butyriciproducens, Butyricimonas virosa, Eubacterium ramulus and Bacteroides fragilis*) are increased in centenarians, indicating that our current cohort might possesses bacteria associated with longevity. A recent study provided evidence that *O. splanchnicus* strains which are increased in centenarians can efficiently synthesize isoalloLCA,^15^ a specific bile acid that can resist many infections such as *Clostridium difficile* and *Enterococcus faecalis*, thereby reducing the risk of infection and assisting in maintaining intestinal homeostasis. In agreement with the report, we observed centenarians showed increased *O. splanchnicus* and younger-like relationship between *O. splanchnicus* and other bacteria. Moreover, our present study showed that *O. splanchnicus* could produce essential amino acids and a positive correlation with L− isoleucine biosynthesis, which indicating *O. splanchnicus* may partially compensated for the decline of essential amino acids caused by aging. Future study based on animal experiments is needed to clarify whether *O. splanchnicus* contribute longevity partially through supplementing essential amino acid.

Age-related dysbiosis is responsible for the age-related increase in systemic inflammation.^33,34^ Consistent with this, the present study showed that the alteration in gut microbiota observed in centenarians correlate with an increase in most pro-inflammatory cytokines in the peripheral blood compared with G2 and G3. Significantly, all differential microbial gene function related to essential amino acid biosynthesis in centenarians were depleted and showed negative association with pro-inflammatory factor IL-17 and IL-12. Increasing studies from animal models have shown that a change in the composition and diversity of the microbiota contributes to human health and fitness by modulating the immune inflammation response and vice versa.^35^ Impressively, we observed that an increase of anti-inflammatory factor, IL-10 in centenarians compared with the elderly, was associated with an increased abundance of *B. fragilis. B. fragilis* strains can induce the induction of regulatory CD4+ and CD8 + T cells secreting IL-10 to control innate inflammatory responses and mediate beneficial anti-inflammatory effects on inflammatory bowel disease.^36,37^ Taken together, the result indicated enriched *B. fragilis* in centenarians might promote longevity through modulating anti-inflammatory factor IL-10 expression to mediate the critical balance between health and disease. Moreover, the data indicated that the host immune system may evolve with microbiome to develop complicated mechanisms to modulate the composition of gut microbiota of longevity.

Age-related changes in microbiota composition have been shown to play a role in development of neurodegenerative disorders, including Parkinson’s disease and Alzheimer’s disease.^38,39^ Normal brain aging is usually associated with changes in neural activity, deposition of Aβ and accumulation of tau proteins, leading to gradual cognitive decline in normal older adults.^40^ Gut microbiota interacts bidirectionally with the central nervous system through the immune, endocrine, and neural systems generally referred to as the microbiota-gut-brain axis.^41^ In our study, we observed a decreased abundance of *B. pseudocatenulatum* in centenarians associated with increased levels of Aβ and pro-inflammatory cytokines such as IL-12, TNF-a, IL-1β, TGF-β, IL-6, and IL-17. Moya-Pérez et al. reported that *B. pseudocatenulatum* can beneficially modulate the consequences of chronic stress on the hypothalamic–pituitary–adrenal axis, particularly response produced by maternal separation mice model.^42^ On the other hand, a growing amount of evidence has pinpointed the availability and metabolism of the essential amino acid, vitamins, and minerals nutrient pattern moderates the effect of brain structure on cognitive function in old age.^43^ It is interesting to note that *B. pseudocatenulatum* showed positively associated with essential amino acid pathways such as L− isoleucine biosynthesis I (from threonine) L− valine biosynthesis L− lysine biosynthesis VI. The above information suggests that *B. pseudocatenulatum* can be a particular beneficial bacterium in the improvement of brain function through modulating the availability and metabolism of the essential amino acid as a key regulator of the gut-brain axis.

Notably, IL-17 and Aβ showed the same trend of negative correlation with microbe-related metabolic activity, such as, peptidoglycan maturation, CDP−diacylglycerol biosynthesis, and L− histidine biosynthesis. Increasing evidences showed elevated pro-inflammatory IL-17 were involved in the procession of Aβ accumulation.^44–46^ Based on the information, the present study may provide important clues to reveal which bacterial metabolic activity is involved in the underlying mechanisms of the cross-talk between IL-17 and Aβ accumulation. Overall, these findings suggest that longevity may be characterized by a balance among core bacteria and between pro-and anti-inflammatory activity.

## Conclusion

Our findings provide a rationale for the establishment of new homeostasis of gut microbiota that thus contributes to improving neural and immune function as well as extending life span. Given the impossibility of performing a longitudinal longevity cohort in human, further studies based on animal experiments are needed to understand whether the interaction of immune response, amino acid metabolic activity, and candidate bacteria involved in the establishment of a new homeostasis with aging that thus promoting human longevity.

## Methods

### Study population recruitment and statical analysis

32 centenarians (G1, aged 100 to 108 years), 30 elderly (G2, aged 52 to 83 years) and 11 young people (G3, aged 16 to 52 years) were recruited from July 2019 to August 2019 in the Rugao, Nantong, Jiangsu. All participants, who had taken antibiotics, microbial agents, serious diseases or underwent intestinal surgery within half a year before admission were excluded. Among the variables we included, classified variables were expressed as percentages, and continuous variables were expressed as Mean ± SD. We used the Chi-Square test or Fisher exact test to compare classified variables, and Analysis of Variance (ANOVA) was used to compare continuous variables (SPSS25, USA). All P-values were two-tailed, and *P* < .05 was recognized as statistically significant.

### Fecal and blood sample collection

Feces and blood were collected at subjects’ homes. The feces were collected in 10 mL sterile container and delivered immediately at low temperatures. The frozen feces were shipped using dry ice overnight to Nanjing Medical University. Once received, fecal samples were divided into three parts of 200 mg and stored at −80°C until extraction. The blood was used coagulation tubes to collect, and then centrifuged at 4000 rpm and the supernatant serum was frozen and stored at −80°C until analysis.

### Blood serum elisa analysis

Eight immune cytokines including IL-6, TGF-β, IL-10, IL-17, TNF-α, IL-12, IL-33, and IL-1β, amyloid β-protein (Aβ), and brain-derived neurotrophic factor (BDNF), following the company’s kit procedures (Ruixinbio Quanzhou, China). The level of immune cytokines in the serum of blood among three groups were statistically analyzed by GraphPad Prism software, respectively.

### DNA isolation and 16S rRNA gene sequencing

In total, 0.18–0.22 g stool samples were used to extract total bacteria DNA following the protocol of the DNA extraction kit (#DP328, Tiangen Company, Beijing, China). The 16S rRNA V4 regions were performed using specific primers 515 F GTGCCAGCMGCCGCGGTAA and 806 R GGACTACHVGGGTWTCTAAT. Sequencing libraries were generated using the Illumina TruSeq DNA PCR-Free Library Preparation Kit (Illumina, USA) with following manufacturer recommendations, and index codes were added. Sequencing was performed in the Illumina Novaseq 6000 platform (Novogene, China).

Microbiome bioinformatics were performed with QIIME2 2021.4.^47^ Shannon index for alpha diversity and Bray-Curtis for beta diversity measures. Principle Coordinate Analysis (PCoA) were analyzed using the vegan v2.5–7 R package. Taxonomy was assigned to ASVs using the qiime feature-classifier classify-sklearn. Naive Bayes classifiers trained on Silva 138 99% OTUs from 515 F/806 R region of sequences. Gene functions analysis were predicted as previous method.^48^ Predicted functions were calibrated for all of samples (n = 73) using Meta-Apo^49^ based on the Kyoto Encyclopedia of Genes and Genomes (KEGG) database. Thirty paired metagenomic samples were used for training.

### Shotgun sequencing for metagenomics

Sequence libraries were generated using NEBNext® Ultra™ DNA Library Prep Kit for Illumina (NEB, USA). The libraries were sequenced on the Illumina Novaseq 6000 platform (insert size 350 bp, read length 150 bp) at the Novogene Bioinformatics Technology Co., Ltd. (Tianjin, China).

Raw sequence reads were trimmed using Trimmomatic v0.39 to remove adapters and low-quality regions and then removed of contaminating human reads using Bowtie2 v2.4.2 (Reference database: GRCh38).^50^ The taxonomic composition was profiled using the default parameters of MetaPhlAn3 v3.0.9.^51^ The functional gene pathway was profiled using the default settings of HUMAnN3 v3.0.0.alpha.3.^52^ Functional potential profiling of microbial communities was performed by HUMAnN3 using pangenomes annotated with UniRef90 on all species detectable per sample with MetaPhlAn3. Functional annotations rely on the MetaCyc database and gut-brain modules (GBMs) database.

### Sequence assembly and genome binning

After removal of host reads, the sequence data per sample were assembled individually using Megahit v1.1.3 included in MetaWRAP v1.3.2.^20^ Bowtie2 v2.4.2^50^ was used to map reads back to the assembled contigs. Then, metagenomic binning was applied to both single-sample assemblies and the co-assemblies using CONCOCT v1.0.0, MaxBin2 v2.2.6, and metaBAT2 v2.12.1. Next, all bins were aggregated and dereplicated using dRep v3.2.0. For the final bins, CheckM v1.0.12 was used to estimate the genome completeness and contamination. The draft metagenome-assembled genomes (MAGs) with completeness ≥70% and contamination ≤10% were retained for the subsequent analyses. The alignment results are then used by taxator-kt to estimate the most likely taxonomy of each contig. The metaWRAP::Annotate_bins module takes in a set of bins and quickly functionally annotates them with PROKKA v1.13.^53^ Using DIAMOND v0.9.24,^54^ all protein predictions were searched against the carbohydrate-active enzymes (CAZy) database^55^ using dbCAN2.^56^ To estimate the genetic relationships among all bins, a maximum likelihood phylogenetic tree was built based on a concatenated protein sequence alignment using the package PhyloPhlAn 3.0.60.^57^ The taxonomic and phylogenetic information were then combined and visualized by ggtree v2.4.2.^58^ Rapid Annotations using Subsystems Technology (RAST)^59^ was used to predict function of MAGs.

### Absolute abundance analysis according to the 16S rRNA data

Analysis of Compositions of Microbiomes with Bias Correction (ANCOM-BC)^60^ was used to calculate absolute abundance according to the 16S rRNA data.

### Co-occurrence microbial network analysis

To understand the correlations among different genera, we constructed co-occurrence network based on the 16S rRNA data. The bacterial correlations in the G3, G2, and G1 samples were analyzed, respectively. Then, the co-occurrence networks were constructed using Spearman’s correlation coefficient value based on the relative abundance of each genus. The significant correlated genus (Benjamini–Hochberg-corrected P value < .05, rho ≥ 0.7) were visualized by Cytoscape v3.8.2. Closeness centrality of the shared nodes were calculated by igraph R package. Only genera existed in at least 20% sample were included in the network analysis.

### Interdependent functional groups (guilds)

Bacterial species in the human gut may also survive, adapt, and decline as interdependent functional groups, which are regarded as guilds responding to environmental perturbations.^18^ Co-abundance analysis can help identify such groups. The ASVs shared by at least 20% among all the samples were considered as prevalent ASVs. Correlations between prevalent ASVs were calculated using the SparCC algorithm based on their abundance.^61^ The correlation values were converted to a correlation distance (1-correlation value), and the ASVs were clustered using the Ward clustering algorithm. The guilds were determined by dividing the cluster tree using permutational MANOVA (9999 permutations, P < .001). Permutational MANOVA was performed using vegan R package. And the guilds network was visualized in Cytoscape.

### Random forest model prediction

RandomForest v4.6–14 R package was used to build classification models using profiles of genera or pathways with significant differences between G3 and G1 or between G2 and G1. The evaluation of the random forest classification models was performed by receiver operator characteristic (ROC) curve analysis, and the area under curve (AUC) was used to assess the ROC effect, the closer the AUC to 1, the better the model performance.^62^ Internal validation used 5-fold cross-validation based on our cohort. External validation used our cohort as training set and another dataset as test set. The importance of markers being used to build classification models were calculated by randomForest v4.6–14 with options importance = TRUE.

### Correlation analysis of species, serum cytokines and pathways

To determine the association between species, cytokines and pathways in G3, G2 and G1 groups, we constructed a correlation analysis using Spearman’s correlations (Benjamini–Hochberg-corrected P value < .05) in psych R package. The results were visualized by networkD3 and pheatmap R packages.

### Nucleotide diversity analysis

InStrain,^62^ a program that uses metagenomic paired reads to profile intra-population genetic diversity (microdiversity) across whole genomes, was used to explore nucleotide diversity of the same species. InStrain considers both major and minor alleles during genomic comparison. The microdiversity-aware average nucleotide identity (ANI) metric is referred to as ‘population ANI (popANI)’ which is a new term to describe a unique type of ANI calculation performed by inStrain that considers both major and minor alleles. A threshold of 99.9% popANI was chosen as the threshold to define bacterial as the same strain. We run inStrain using the UHGG genome collection. The links drawn for each strain shared between G3 individuals, G2 individuals and G1 individuals, respectively, were visualized using circlize v0.4.13 R package.

### Resistance gene annotations

The predicated antibiotic resistance genes (ARGs) were searched by ARGs-OAP v2.0^63^ based on the CARD, ARDB and NCBI-NR databases. ARGs-OAP v2.0 improves cell number quantification by using the average coverage of essential single copy marker genes.

## Statistical analysis

All statistical analysis was conducted by using R version 4.0.5 and GraphPad Prism software. All data represented as the mean ± SEM. Bacterial taxonomic analyses and comparisons were conducted between two groups using Wilcoxon rank sum test. P value was corrected with the Benjamini-Hochberg method;^64^ *P < .05, **P < .01, ***P < .001, ****P < .0001 and n.s. indicates not significant (P > .05).

## Supplementary Material

Supplemental Material

## Data Availability

All 16S rRNA and metagenomics raw data have been deposited into CNGB Sequence Archive (CNSA) of China National GeneBank DataBase (CNGBdb) with accession number CNP0002519.
