# Supplementary material for: The landscape in the gut microbiome of long-lived families reveals new insights on longevity and aging – relevant neural and immune function
Source: Gut Microbes. 2022 Aug 8;14(1):2107288. doi: 10.1080/19490976.2022.2107288 (PMC9361766; doi:10.1080/19490976.2022.2107288)
Supplement: Supplemental Material [file KGMI_A_2107288_SM1981.zip › supplemental figues.pdf]

Supplementary online material

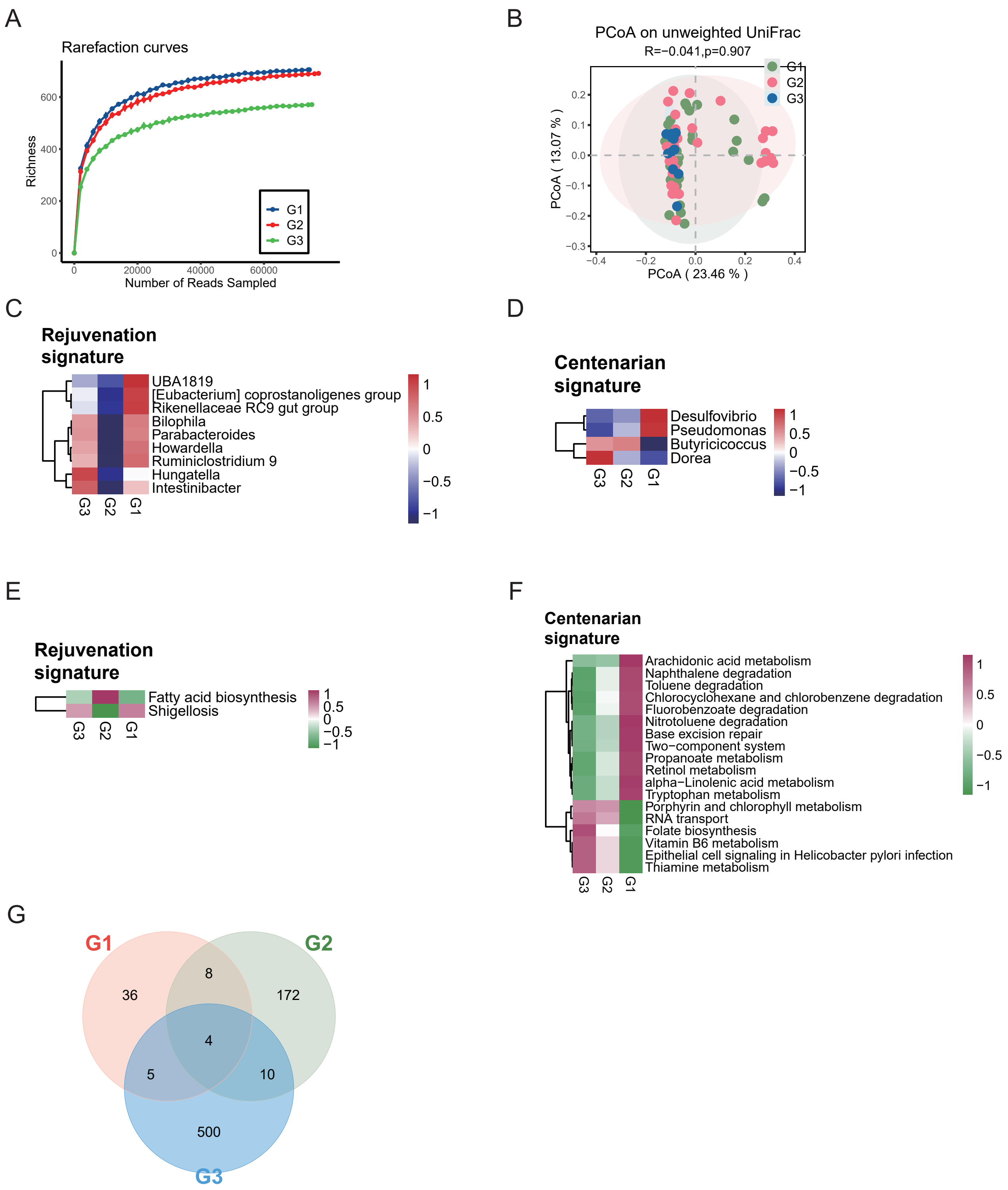

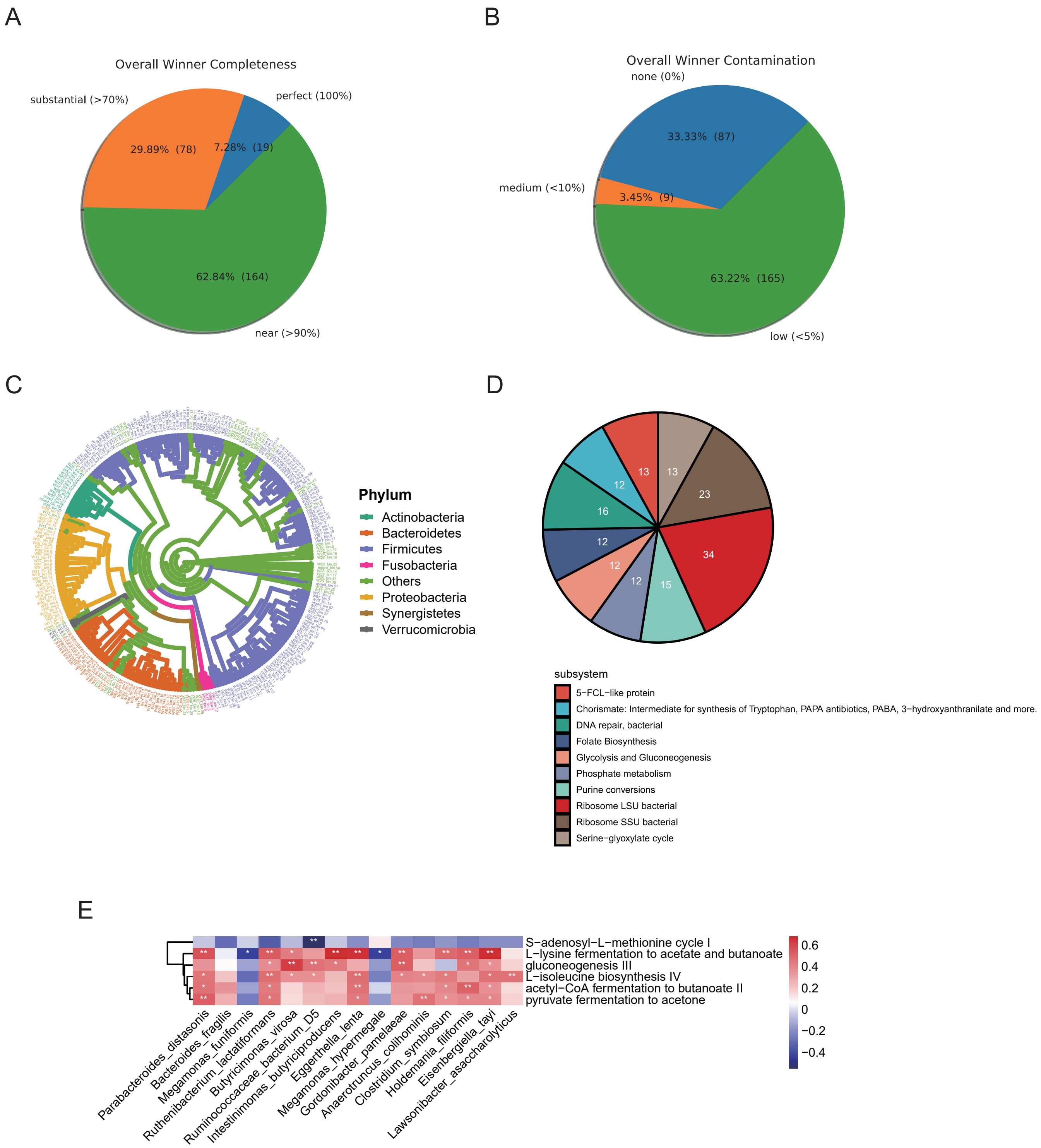
A
Overall Winner Completeness
substantial (>70%)
perfect (100%)
29.89% (78)
7.28% (19)
62.84% (164)
near (>90%)
B
Overall Winner Contamination
none (0%)
33.33% (87)
medium (<10%)
3.45% (9)
63.22% (165)
low (<5%)
C
Phylum
Actinobacteria
Bacteroidetes
Firmicutes
Fusobacteria
Others
Proteobacteria
Synergistetes
Verrucomicrobia
D
13
13
12
23
16
12
34
12
12
15
subsystem
5−FCL−like protein
Chorismate: Intermediate for synthesis of Tryptophan, PAPA antibiotics, PABA, 3−hydroxyanthranilate and more.
DNA repair, bacterial
Folate Biosynthesis
Glycolysis and Gluconeogenesis
Phosphate metabolism
Purine conversions
Ribosome LSU bacterial
Ribosome SSU bacterial
Serine−glyoxylate cycle
E
S−adenosyl−L−methionine cycle I
L−lysine fermentation to acetate and butanoate
gluconeogenesis III
L−isoleucine biosynthesis IV
acetyl−CoA fermentation to butanoate II
pyruvate fermentation to acetone
0.6
0.4
0.2
0
−0.2
−0.4
Parabacteroides_distasonis
Bacteroides_fragilis
Megamonas_funiformis
Ruthenibacterium_lactatiformans
Butyricimonas_virosa
Ruminococcaceae_bacterium_D5
Intestinimonas_butyriciproducens
Eggerthella_lenta
Megamonas_hypermegale
Gordonibacter_pamelaeae
Anaerotruncus_colihominis
Clostridium_symbiosum
Holdemania_filiformis
Eisenbergiella_tayi
Lawsonibacter_asaccharolyticus
