# Supplementary material for: The landscape in the gut microbiome of long-lived families reveals new insights on longevity and aging – relevant neural and immune function
Source: Gut Microbes. 2022 Aug 8;14(1):2107288. doi: 10.1080/19490976.2022.2107288 (PMC9361766; doi:10.1080/19490976.2022.2107288)
Supplement: Supplemental Material [file KGMI_A_2107288_SM1981.zip › Supplemental Tables.pdf]

Table S1A Characteristics of study participants for 16S rRNA sequencing

Parameter	G1 (n = 32)	G2 (n = 30)	G3 (n = 11)	*P*
Age, mean± SD	101.7 ± 2.5	71.0 ± 7.2	37.1 ± 10.5	**< 0.001**
Gender, n (%)				**< 0.001**
male	5 (15.6)	21 (70)	4 (36.4)	
female	27 (84.4)	9 (30)	7 (63.6)	
BMI, Mean ± SD	19.2 ± 2.7	22.5 ± 3.1	21.4 ± 2.2	**< 0.001**
Smoking status, n (%)				**0.012**
Ever	2 (6.2)	9 (30)	0 (0)	
Never	30 (93.8)	21 (70)	11 (100)	
Alcohol consumption, n (%)				**0.019**
Ever	17 (53.1)	17 (56.7)	1 (9.1)	
Never	15 (46.9)	13 (43.3)	10 (90.9)	
Food Preference, n (%)				0.051
Balanced-diet	14 (43.8)	15 (50)	1 (9.1)	
Vegetable preference	11 (34.4)	12 (40)	9 (81.8)	
Meat preference	7 (21.9)	3 (10)	1 (9.1)	
Diseases, n (%)				0.082
None	21 (65.6)	19 (63.3)	11 (100)	
Hypertension	11 (34.4)	9 (30)	0 (0)	
Diabetes mellitus	0 (0)	1 (3.3)	0 (0)	
Coronary heart disease	0 (0)	1 (3.3)	0 (0)	

Notes: The p-values of gender, smoking status, food preference and diseases were calculated using Fisher exact test, the p-value of alcohol consumption was calculated using Chi-Square Test, other p-values were calculated using ANOVA. Significant p-values are highlighted by bold font.

Table S1B Characteristics of study participants for metagenomics sequencing

Parameter	G1 (n = 10)	G2 (n = 10)	G3 (n = 10)	P
Age, median (Q1, Q3)	101.2 ± 1.5	69.7 ± 7.9	39.2 ± 8.3	**< 0.001**
Gender, n (%)				**0.008**
male	1 (10)	8 (80)	3 (30)	
female	9 (90)	2 (20)	7 (70)	
BMI, Mean ± SD	20.6 ± 2.7	22.8 ± 3.6	21.5 ± 2.3	0.249
Smoking status, n (%)				0.31
Ever	0 (0)	2 (20)	0 (0)	
Never	10 (100)	8 (80)	10 (100)	
Alcohol consumption, n (%)				**0.046**
Ever	6 (60)	6 (60)	1 (10)	
Never	4 (40)	4 (40)	9 (90)	
Food Preference, n (%)				0.09
Balanced-diet	6 (60)	4 (40)	1 (10)	
Vegetable preference	2 (20)	4 (40)	8 (80)	
Meat preference	2 (20)	2 (20)	1 (10)	
Diseases, n (%)				0.324
None	8 (80)	6 (60)	10 (100)	
Hypertension	2 (20)	2 (20)	0 (0)	
Diabetes mellitus	0 (0)	1 (10)	0 (0)	
Coronary heart disease	0 (0)	1 (10)	0 (0)	

Notes: The p-values of gender, smoking status, alcohol consumption, food preference and diseases were calculated using Fisher exact test, other p-values were calculated using ANOVA. Significant p-values are highlighted by bold font.

Table S2. The detailed characteristics for all samples

Group	Sample	Age	Gender	BMI (kg/m 2)	Smoking status	Alcohol consumptior	Taking probiotics or not	Food Preference	Diseases	16S RNA sequencir	Metagenomic sequencing	Elisa
G1	W1	100	female	17.77777778	Never	Ever	Never	Balanced-diet	Hypertension	yes	no	yes
	W4	100	female	18.73048907	Never	Never	Never	Balanced-diet	None	yes	no	yes
	W7	100	female	20	Never	Never	Never	Meat preference	None	yes	no	yes
	W10	100	female	17.578125	Never	Never	Never	Vegetable preference	None	yes	no	yes
	W11	100	female	18.36547291	Never	Never	Never	Vegetable preference	Hypertension	yes	no	yes
	W12	102	female	18.36547291	Never	Ever	Never	Balanced-diet	Hypertension	yes	yes	yes
	W14	100	female	20.44444444	Never	Ever	Never	Vegetable preference	Hypertension	yes	no	yes
	W19	106	female	17.31301939	Never	Ever	Never	Meat preference	None	yes	no	yes
	W21	108	female	18.87066338	Never	Ever	Never	Meat preference	None	yes	no	yes
	W25	101	female	16.82422689	Never	Never	Never	Balanced-diet	None	yes	yes	yes
	W28	100	female	19.53125	Never	Ever	Never	Vegetable preference	None	yes	yes	yes
	W32	102	female	25.10387812	Never	Never	Never	Balanced-diet	None	yes	yes	yes
	W36	104	female	15.625	Never	Never	Never	Meat preference	None	yes	no	yes
	W38	107	male	21.4532872	Never	Ever	Never	Balanced-diet	None	yes	no	yes
	W40	101	female	18.73048907	Never	Ever	Ever	Balanced-diet	None	yes	yes	yes
	W45	100	male	25.95155709	Never	Never	Never	Vegetable preference	Hypertension	yes	no	yes
	W48	100	female	19.55555556	Never	Never	Never	Balanced-diet	Hypertension	yes	no	yes
	W49	100	female	22.22222222	Never	Never	Never	Vegetable preference	None	yes	yes	yes
	W51	100	female	16.64932362	Never	Never	Never	Balanced-diet	Hypertension	yes	no	yes
	W54	101	female	17.85714286	Never	Never	Never	Vegetable preference	None	yes	no	yes
	W57	101	female	17.578125	Never	Ever	Never	Vegetable preference	Hypertension	yes	no	yes
	W59	103	male	17.30103806	Never	Ever	Never	Vegetable preference	None	yes	no	yes
	W61	101	female	23.11111111	Never	Ever	Never	Balanced-diet	None	yes	yes	yes
	W63	100	female	19.3877551	Never	Never	Never	Meat preference	Hypertension	yes	yes	yes
	W65	102	female	16.64932362	Never	Ever	Never	Balanced-diet	None	yes	no	yes
	W66	105	female	18.76524676	Never	Ever	Never	Meat preference	None	yes	yes	no
	W68	100	female	20.81165453	Never	Never	Never	Vegetable preference	None	yes	no	yes
	W70	100	male	23.52941176	Never	Ever	Never	Balanced-diet	None	yes	yes	no
	W72	108	female	14.69237833	Never	Ever	Never	Balanced-diet	Hypertension	yes	no	no
	W73	100	female	15.625	Never	Never	Never	Vegetable preference	None	yes	no	yes
	W75	101	male	20.51508648	Ever	Ever	Never	Meat preference	None	yes	no	no
	W78	100	female	19.11111111	Ever	Ever	Never	Balanced-diet	Hypertension	yes	no	no
G2	W2	75	male	23.7332384	Never	Ever	Never	Balanced-diet	Hypertension	yes	no	yes
	W5	70	male	22.03172568	Ever	Never	Never	Vegetable preference	Hypertension	yes	no	yes
	W8	72	male	23.87511478	Ever	Never	Never	Balanced-diet	None	yes	no	yes
	W13	66	male	25.01352082	Ever	Ever	Never	Balanced-diet	Hypertension	yes	yes	yes
	W15	78	female	16.64932362	Never	Ever	Ever	Vegetable preference	Hypertension	yes	no	yes
	W18	77	male	23.52941176	Never	Ever	Never	Balanced-diet	None	yes	no	yes
	W20	80	male	21.484375	Ever	Ever	Never	Balanced-diet	None	yes	no	yes
	W22	75	male	26.34649403	Ever	Ever	Never	Vegetable preference	None	yes	no	yes
	W24	83	female	22.89281998	Never	Never	Never	Balanced-diet	Hypertension	yes	no	yes
	W26	62	male	20.76124567	Never	Never	Never	Vegetable preference	Diabetes mellitus	yes	yes	yes
	W29	76	female	16.64932362	Never	Never	Never	Vegetable preference	None	yes	yes	yes
	W33	74	male	27.34375	Never	Never	Never	Vegetable preference	None	yes	yes	yes
	W37	71	female	19.14672216	Never	Never	Never	Vegetable preference	Hypertension	yes	no	yes
	W39	81	male	22.85714286	Never	Ever	Never	Balanced-diet	None	yes	no	yes
	W41	76	male	23.33546614	Never	Ever	Never	Vegetable preference	Hypertension	yes	yes	yes
	W46	56	male	22.85714286	Never	Never	Never	Vegetable preference	Hypertension	yes	no	yes
	W50	77	male	27.34375	Never	Ever	Never	Balanced-diet	Coronary heart di	yes	yes	yes
	W52	58	male	23.38868656	Ever	Ever	Never	Balanced-diet	None	yes	no	yes
	W55	64	male	22.49134948	Ever	Ever	Never	Balanced-diet	None	yes	no	yes
	W60	67	male	18.9370029	Never	Ever	Never	Balanced-diet	None	yes	no	yes
	W62	71	female	18.02595738	Never	Ever	Never	Balanced-diet	None	yes	yes	yes
	W64	74	male	25.390625	Never	Never	ever	Meat preference	None	yes	yes	yes
	W67	52	male	22.09317005	Ever	Ever	Never	Meat preference	None	yes	yes	yes
	W69	69	female	20.02884153	Never	Never	Never	Vegetable preference	None	yes	no	yes
	W71	69	male	21.97133586	Never	Ever	Never	Balanced-diet	None	yes	yes	no
	W74	75	female	19.53125	Never	Never	Never	Vegetable preference	None	yes	no	yes
	W76	72	male	30.11621315	Ever	Ever	Never	Vegetable preference	None	yes	no	no
	W77	70	female	21.64412071	Never	Never	Never	Meat preference	Hypertension	yes	no	no
	W79	68	female	20.2020202	Never	Never	Never	Balanced-diet	None	yes	no	no
	W80	71	male	23.98687034	Never	Ever	Never	Balanced-diet	None	yes	no	no
G3	W3	47	female	24.76756592	Never	Never	Never	Vegetable preference	None	yes	yes	yes
	W6	37	female	20.82999519	Never	Never	Never	Vegetable preference	None	yes	yes	yes
	W9	44	female	20.93663912	Never	Never	Never	Vegetable preference	None	yes	yes	yes
	W16	33	male	21.87755102	Never	Never	Never	Vegetable preference	None	yes	yes	yes
	W27	35	female	22.89281998	Never	Never	Never	Vegetable preference	None	yes	yes	yes
	W30	52	female	18.82711104	Never	Never	Ever	Vegetable preference	None	yes	yes	yes
	W31	29	female	17.1875	Never	Never	Never	Meat preference	None	yes	yes	yes
	W34	50	male	22.85714286	Never	Never	Never	Balanced-diet	None	yes	yes	yes
	W35	16	male	21.2585034	Never	Never	Never	Vegetable preference	None	yes	no	yes
	W47	31	female	23.87511478	Never	Never	Never	Vegetable preference	None	yes	yes	yes
	W53	34	male	20.51508648	Never	Ever	Never	Vegetable preference	None	yes	yes	yes

Table S3. Relative abundance at phylum level based on 16S rRNA data

Phylum	W10	W11	W12	W14	W19	W1	W21	W25	W28
Others	0	0	4.16E-05	0.014972111	0	0.001757469	0.00078675	7.57E-05	4.55E-05
Actinobacte	0.003175005	0.002535777	0.001456089	0.00184705	0.001147419	0.030577322	0.005846367	0.010026612	0.003493837
Bacteroidet	0.625447802	0.550992055	0.501282745	0.271234954	0.454670849	0.440701931	0.331520191	0.34687031	0.482991726
Firmicutes	0.269720852	0.302634241	0.392408925	0.506972306	0.197172955	0.392338491	0.244625005	0.335229351	0.388954011
Fusobacteri	0.00494514	0.008538016	0.002690297	0.000770623	0.002746481	0.003554581	0.007718289	0.000769337	0.000159328
Lentisphaer	0	0.000526039	9.71E-05	0	0.000915494	0.000581418	0.000963091	0.000580156	0
Proteobacte	0.063092679	0.081900214	0.077158825	0.107923965	0.327588101	0.101113945	0.403792678	0.170162318	0.114773128
Synergistete	0.031258341	0.000606968	0.000416025	0.000550445	0.001806574	0.000753201	0.000366246	0.000416199	0.00020485
Tenericutes	0.000154536	0.003439485	0.002135597	0.094211762	0.007616909	0.000264281	0.003214823	0.037420071	0.007761554
Verrucomic	0.002205645	0.048827203	0.022312823	0.001516782	0.006335217	0.028357361	0.00116656	0.098449974	0.001616042
Phylum	W32	W36	W38	W40	W45	W48	W49	W4	W51
Others	0.000274914	0	0	0	0.000294712	0.001206383	5.54E-05	0.00209157	0
Actinobacte	0.001003436	0.001563908	0.005092123	0.000680452	0.009964073	0.002120309	0.001343081	0.003188369	0.001061414
Bacteroidet	0.52467354	0.469729099	0.520362531	0.700212468	0.585438419	0.459412528	0.42610285	0.27273307	0.630721896
Firmicutes	0.369264605	0.200021206	0.327398485	0.257960589	0.282909509	0.505766876	0.516906206	0.565297794	0.133724758
Fusobacteri	0.000934708	0.002239835	0.000906326	0.003721654	0.003213764	0	0.007836947	0.004820814	0.158043236
Lentisphaer	0	0	0	0	0.000126305	5.48E-05	0.000263078	0.002359393	2.69E-05
Proteobacte	0.051958763	0.060369506	0.12118538	0.033661524	0.116074436	0.031329397	0.04594168	0.120584109	0.074003413
Synergistete	0.000274914	0.001537401	9.54E-05	0	0.000336814	0	0.000346155	0.001122306	0.001061414
Tenericutes	0.049594502	0.000702433	0.003112516	0.003763314	0.001277085	0	0	0.011745951	0
Verrucomic	0.002020619	0.263836611	0.021847236	0	0.000364882	0.000109671	0.001204619	0.016056625	0.001356998
Phylum	W54	W57	W59	W61	W63	W65	W66	W68	W70
Others	0.000574642	0.004702412	0.003388873	9.66E-05	0.005365518	0.001612121	4.75E-05	0.000695108	0.002340588
Actinobacte	0.002396381	0.004312863	0.028579497	0.002064818	0.002331682	0.003181442	0.001366072	0.001086107	0.003944085
Bacteroidet	0.554762196	0.320612705	0.338280147	0.553914702	0.501497046	0.499486404	0.284273547	0.526703739	0.515718147
Firmicutes	0.383848881	0.333885194	0.463724936	0.246003188	0.394478154	0.263046766	0.572692824	0.420569409	0.378619182
Fusobacteri	0.000916983	0.003770277	0.01296244	0.120713389	0.002146207	0.008788199	0.001164131	0.000419961	0.014651304
Lentisphaer	0.000354567	0.002086869	0.000423609	0.000229424	0.000278212	0.021571033	0.000451398	0.000188258	0.000310354
Proteobacte	0.050886416	0.261025627	0.106721265	0.039364374	0.08945179	0.155220133	0.135739995	0.034755409	0.077898902
Synergistete	7.34E-05	0.000695623	0.000720136	0	0	0.000485063	0.000356366	8.69E-05	0.000297423
Tenericutes	0.004633818	0.007707504	0.007949732	0.000531298	0.003563764	0.012283505	0.002850932	0.015046196	0.002870776
Verrucomic	0.001552757	0.061200924	0.037249365	0.037082206	0.000887629	0.034325335	0.001057221	0.000448924	0.003349239
Phylum	W72	W73	W75	W78	W7	W13	W15	W18	W20
Others	6.38E-05	0	0.000584315	0	0.000271044	0.000112988	0	0.000913409	0.000238892
Actinobacte	0.01149308	0.001173709	0.008478791	0.001488924	0.004089232	0.005098582	0.002817506	0.004701011	0.003042724
Bacteroidet	0.492735506	0.643056585	0.662564026	0.361290573	0.511896485	0.523840461	0.371152777	0.213774205	0.463650766
Firmicutes	0.464417374	0.328057821	0.262432244	0.584978702	0.306822065	0.192573866	0.443953101	0.601449275	0.488030276
Fusobacteri	0.002321577	0.009364962	0.006029638	0.000453151	0.041410844	0.11243715	0.000619093	0.003446596	0.002124877
Lentisphaer	0	0	0	0	5.89E-05	0.000254223	0.000113711	0.000280112	5.03E-05
Proteobacte	0.028088526	0.016123054	0.058071013	0.048215234	0.133836926	0.122690808	0.060191035	0.150140056	0.036424674
Synergistete	8.93E-05	0	7.46E-05	0.002175123	0.000353536	0.000494322	0.020910194	0.000523688	0.000402344
Tenericutes	0.000535748	0.000790709	0.001678353	0.001372399	0.000353536	0.002725835	0.002590084	0.02374863	0.003797118
Verrucomic	0.000255118	0.00143316	8.70E-05	2.59E-05	0.000907409	0.039771764	0.097652499	0.001023018	0.002238037
Phylum	W22	W24	W26	W29	W2	W33	W37	W39	W41
Others	4.70E-05	0.00015449	0.001465302	5.07E-05	0.000308748	0.000240842	0	0.009384459	0
Actinobacte	0.000798478	0.001351786	0.003882425	0.002028166	0.001269297	0.001734062	0.004785811	0.003925612	0.000845981
Bacteroidet	0.47940396	0.671464435	0.341064786	0.421820533	0.411606632	0.4053491	0.415372521	0.534742324	0.683438976
Firmicutes	0.427655527	0.170930158	0.593735519	0.451304998	0.454762722	0.512824836	0.376313684	0.386983359	0.223679891
Fusobacteri	0.004133299	0.080811072	0.00107706	3.80E-05	0.000903373	0.000794779	0.000234463	0.0047451	0.001502563
Lentisphaer	2.35E-05	3.86E-05	0.000275527	0	0	0	8.28E-05	6.61E-05	0
Proteobacte	0.085061412	0.065864178	0.033814671	0.121043491	0.124516867	0.066147251	0.093564671	0.059280701	0.089257304
Synergistete	0.000234847	0.002381719	0.00041329	0.000823943	0.000457404	9.63E-05	9.65E-05	2.64E-05	0.000580823
Tenericutes	0.001702637	0.002407467	0.021528674	0.000595774	0.005786164	0.001035621	0.01384713	0	0
Verrucomic	0.000939386	0.004596073	0.002742746	0.002294363	0.000388794	0.011777173	0.095702425	0.000845923	0.000694462
Phylum	W46	W50	W52	W55	W5	W60	W62	W64	W67
Others	0	0.00012545	9.81E-05	0.001042875	0.000244684	0.000354325	0.001667004	0.001819981	0.001763897
Actinobacte	0.001715636	0.000815425	0.020139579	0.002599844	0.006175357	0.003684981	0.001833704	0.008049445	0.001587507
Bacteroidet	0.52665235	0.498237427	0.616967779	0.517618719	0.394418876	0.156871545	0.467118345	0.485800486	0.54058726
Firmicutes	0.30009238	0.200920803	0.276029976	0.387905583	0.547078357	0.494236311	0.431337294	0.44293933	0.335328496
Fusobacteri	0.120922364	0.246145547	0.002477585	0.001321955	0.003425575	0.00338971	0.005786885	0.000879454	0.031585508
Lentisphaer	0.000131972	0	0.000110387	0.000308456	0.000955433	0.000330703	0.000369122	0.000378654	0.000117593
Proteobacte	0.048901633	0.053755347	0.079380849	0.085603913	0.040955433	0.339077337	0.086017408	0.056724768	0.08748927
Synergistete	0	0	0	0	0.000477716	0.000129919	8.34E-05	6.11E-05	0
Tenericutes	0.001487685	0	0.003115379	0.001248513	0.005662686	0.000826759	0.001274067	0.001123746	0.000787874
Verrucomic	9.60E-05	0	0.001680342	0.002350142	0.000605884	0.001098408	0.004512818	0.002223064	0.000752596
Phylum	W69	W71	W74	W76	W77	W79	W80	W8	W16
Others	0.001003166	0.001976123	0.000902321	0.001169414	0.001048565	3.89E-05	0	3.90E-05	0.000318975
Actinobacte	0.001745769	0.017348997	0.001588085	0.003910622	0.001766004	0.005693904	0.004847847	0.002156068	0.004545394
Bacteroidet	0.637922274	0.34549444	0.54277602	0.37079231	0.605339404	0.518417639	0.759380584	0.451423525	0.578035911
Firmicutes	0.307620152	0.589961295	0.368880761	0.577816339	0.284671634	0.435629053	0.15918944	0.310006234	0.336638269
Fusobacteri	0.000938025	0.001907981	0.041242075	0.000477825	0.001559051	0.003981842	0.00041553	0.148729738	0.000996797
Lentisphaer	0	0	0	0	0.002235099	0	0	0.000246779	0.000571497
Proteobacte	0.049389632	0.038813781	0.038282462	0.043972487	0.096495585	0.03542153	0.075529454	0.085879052	0.070333993
Synergistete	0	0.000163541	7.22E-05	0.000150892	0.000193157	0	0	0.0005585	0.000824019

Tenericutes	0.000651406	0.002412233	0.001672301	0.001295158	0.004925497	0.00079118	0.000637146	0.000415628	0.00594091
Verrucomicr	0.000729575	0.001921609	0.00458379	0.000414953	0.001766004	2.59E-05	0	0.000545511	0.001794235
Phylum	W27	W30	W31	W34	W35	W3	W47	W53	W6
Others	0	0.00028103	0	0	0	0	9.99E-05	0.00030906	0
Actinobacte	0.00292145	0.008512881	0.004935001	0.002503461	0.005347087	0.000717633	0.000836475	0.002627012	0.000993591
Bacteroidete	0.635998299	0.494145199	0.321762055	0.507363533	0.779278853	0.729410275	0.328697346	0.558816537	0.474194632
Firmicutes	0.278977904	0.382740047	0.476630174	0.399448959	0.175980694	0.218427681	0.554720467	0.399923924	0.431960032
Fusobacteri	0.001138405	0.000398126	0.002012961	0.002363603	0.003028439	0.000661348	0.092499188	0.008665573	0.004953959
Lentisphaer	0.000123442	0.000269321	0	5.59E-05	0	8.44E-05	0	0	0.000139942
Proteobacte	0.074668422	0.102775176	0.189127414	0.085607194	0.034401174	0.046829049	0.020861944	0.027494473	0.083265695
Synergistete	0.000233167	0.004871194	0.000168829	0.000125872	0	0.001969972	8.74E-05	0	0.000685717
Tenericutes	0.000891522	0.002903981	0.003831119	0.00093705	0.000674301	0.000211068	0.002084946	0.001747379	0.002854824
Verrucomicr	0.005047388	0.003103044	0.001532448	0.001594383	0.001289453	0.001688547	0.000112362	0.000416043	0.000951608
Phylum	W9								
Others	0								
Actinobacte	0.001067798								
Bacteroidete	0.638087645								
Firmicutes	0.273683761								
Fusobacteri	0.004698311								
Lentisphaer	0								
Proteobacte	0.081380449								
Synergistete	0.000327458								
Tenericutes	0.000242034								
Verrucomicr	0.000512543								

Table S4. Relative abundance at genus level based on 16S rRNA data									
Genus	W10	W11	W12	W14	W19	W1	W21	W25	W28
[Caedibacte	0	0	0	0	0	0	0	0	0
[Clostridium	0.000142566	6.98E-05	7.54E-05	4.09E-05	5.30E-05	0.00075157	0.000322538	0.000246248	0.000167989
[Eubacteriu	0	6.98E-05	0	0	0	0.000340334	0.000117287	0.000115882	0
[Eubacteriu	0.003706713	0.011132657	0.011033069	0.041083571	0.032132201	0.011372822	0.00873785	0.017150472	0.019426679
[Eubacteriu	0.002922601	0.003822491	0.005200012	0.008393857	0.002524673	0.004282534	0.00321072	0.003563358	0.006839535
[Eubacteriu	0.000413441	0.001701985	0.000768697	0.002098464	0.001041648	0.00097846	0.001304813	0.001303668	0.00131991
[Eubacteriu	0.00062729	0.000306915	0	0.000231649	0.000247171	0.000141806	0.001407439	0.000173822	0
[Eubacteriu	0.000242362	0.000683584	0.00090435	0.006090997	0.002524673	0.000439598	0.001949889	0.002578365	0.00235184
[Eubacteriu	0.000484724	0.002511126	0.000859132	0.001130991	0.001889091	0.00049632	0.001554047	0.000869112	0.001751881
[Eubacteriu	0.000142566	0.000585929	0.004853345	0.001798684	0.00167723	0.000155986	0.000381181	0.000289704	0.001331909
[Polaribacte	0	0	0	0	0	0	0	0	0
[Ruminococ	0	0.000474324	0	0.000231649	0	0	0	0	0.000191987
[Ruminococ	0.000826882	0.000530127	0.00464233	0.001185496	0.000211861	0.000893376	0.001260831	0.000478011	0.001331909
[Ruminococ	0.002224028	0.0039899	0.012118289	0.024786406	0.003513356	0.004637048	0.004119691	0.006083782	0.005375634
Abiotrophia	0	0	0	0	0	0	0	0	0
Acetanaerol	0	0	0	0.000122638	0	8.51E-05	4.40E-05	5.79E-05	0
Acetitomacu	0	0	0	0	0	0	0	8.69E-05	0
Acetobacter	0	0	0	0	0	0	0	0	0
Acidaminoc	0.027643528	0.000265063	0.000211015	0.000572308	0.000158895	0.000510501	0.000747702	0	0.001415904
Acinetobact	0	5.58E-05	0	0	0	0.000184347	0	0	0
Actinobacillu	0	0	0	0	0	0	0	0	0
Actinomyce	0	0.000181359	0.000135652	0.000367913	0	0.000581404	0.00071838	0.000376615	0.000347976
Adlercreutzi	0	0.000125556	0	0.00014989	0.000158895	0	0	0.001028449	0.000107993
Aeromonas	0.000270875	0	0	0.000122638	0	0	0	0	0.000263982
Aerosphaer	0	0	0.00048232	0	0	0	0	0	0
Aestuariicel	0	0	0	0	0	0	0	0	0
Agathobacte	0.003706713	0.026157559	0.088927742	0.007085724	0.008156636	0.006934301	0.003709188	0.004895996	0.006047589
Aggregatiba	0	0	0	0	0	0	0	0	0
Akkermansi	0.002238285	0.050501528	0.02425165	0.001689673	0.009162974	0.030431515	0.001260831	0.113071441	0.001703884
Alcaligenes	0	0	0	0	0	0	0	0	0
Alcanivorax	0	0	0	0	0	0	0	0	0
Alistipes	0.039547781	0.170268272	0.022593676	0.022878711	0.048215957	0.138062083	0.059552258	0.082203488	0.030693913
Allisonella	0.00054175	0.000251113	0.000828987	0.000763078	0.000600272	0.000226889	0.000557111	0.000318674	0.000719951
Allobaculum	0	0	0	0	0	0	0	0	0
Alloprevotel	0.000641547	0.000725436	0.00054261	0.002057585	0.000282481	0.000709029	0	0	0.00235184
Allorhizobiu	0	0	0	0	0	0	0	0	0
Alloscardov	0	0	0	0	0	0	0	0	0
Amphritea	0	0	0	0	0	0	0	0	0
Anaerofilum	0	0	0	8.18E-05	0	8.51E-05	4.40E-05	0	0
Anaeroglob	0	0	0	0	0	0	0	0	0
Anaerospor	0	0.000251113	0.0006029	0	0.000600272	0.000439598	0	0	0
Anaerostipe	0.000199592	0.001185809	0.001416815	0.000436045	0	0.000609765	0.000249234	0.000927053	0.000479967
Anaerotrunc	0.00067006	0.0009905	0.000211015	0.000286154	0.001182889	0.002240531	0.000102626	0.000217278	0.000167989
Angelakisell	0	0	0	0.00132176	0	0.000921737	0	0.000115882	0.000131991
Anoxybacillu	0	0	0	0	0	0	0	0	0
Anseongella	0	0	0	0	0	0	0	0	0
Arenibacter	0	0	0	0	0	0	0	0	0
Atopobium	0.000413441	0	0	0	0.000158895	0	0.000835667	0	8.40E-05
Atopostipes	0	0	0	0	3.53E-05	0	0.000967614	0	0
Aureimarina	0	0	0	0	0	0	0	0	0
Azospirillum	0.00071283	0.001548528	0.00054261	0.000272528	0.000264826	5.67E-05	0.001319474	0.028984877	0.000323978
Bacillus	0	0	0	0	0	0	0.00035186	0	0
Bacteroides	0.532269792	0.31269095	0.424984174	0.155368117	0.545859007	0.260837505	0.250538785	0.195666029	0.409448158
Balneola	0	0	0	0	0	0	0	0	0
Barnesiella	0.001311606	0.033830443	0.009450457	0.009279573	0.008086015	0.001744211	0.002360392	0.048264674	0.0014759
Bifidobacter	0.00058452	0.00119976	0.000437102	0.000136264	0.000247171	0.031594322	0	0.002520424	0.000767948
Bilophila	0.014498952	0.002552978	0.001296235	0.001594288	0.002983704	0.006437981	0.022812239	0.001622342	0.00367175
Blautia	0.003963332	0.010504876	0.008109004	0.0151798	0.007291538	0.004452701	0.007169142	0.007372965	0.009719339
Bosea	0	0	0	0	0	0	0	0	0
Brevibacillus	0	0	0	0	0	0	0	0	0
Brevundimo	0	0	0	0	0	0	0	0	0
Butyricicocc	0.000684316	0.002455323	0.005079432	0.004251434	0.00310729	0.001687488	0.001744638	0.00298395	0.001331909
Butyricimon	0.000527494	0.000502225	0.001642902	0.006826822	0.002153917	0.003389158	0.00499934	0.00482357	0.003251779
Butyrivibrio	0	0	0.000165797	0.000136264	0	0	0	0	0
C1-B045	0	0	0	0	0	0	0	0	0
CAG-352	0	0	0	0	0	0	0	0	0
CAG-56	0.000327902	0.000767288	0.000226087	0.000626814	0.000335446	0.000340334	0.000161269	0.000535952	0.000899939
CAG-873	0	0	0	0	0	0	0	0	0
Campylobac	0	0	0	0	0	0	0	0	0
Candidatus	0	0	0	0	0	0	0	0	0
Candidatus	0	0	0	0	0	0	0	0	0
Candidatus	0	0	0	0	0	0	0	0	0
Candidatus	0	0	0	0	0	0	0	0	0
Candidatus	0	0	0	0	0	0	0	0	0
Candidatus	0	0	0.00018087	8.18E-05	8.83E-05	0.000311973	0	8.69E-05	0
Candidatus	0.000213849	0	0	0	0	0	0	0	0
Caproicipro	0	0	9.04E-05	0	0	0	0	0	0
Catabacter	0	0	0	0.000218022	0	0	0	0	0
Catenibacte	0	0	0	0	0	0	0	0	0
Cellulosilytic	0	0	0	0	0	0	0	0.000231763	6.00E-05
Cellvibrio	0	0	0	0	0	0	0	0	0
Cephalotico	0	0	0	0	0	0	0	0	0
Christensen	0	0	0	0	0	0	0	0	0
Christensen	0.001454172	0.007840292	0.004597112	0.026367068	0.007838845	0.004084006	0.005307218	0.028419955	0.01205918
Chryseoline	0	0	0	0	0	0	0	0	0
Chthoniobac	0	0	0	0	0	0	0	0	0
Clavibacter	0	0	0	0	0	0	0	0	0
Cloacibacillu	0	0	0	0	0	0	0.000397056	0	0

Clostridiales	0	0	0	0	0	0	0	0	0
Clostridioide	0	0	0	0	0	0	0	0	0
Clostridium	0.000570264	0.001674084	0.00108522	0.008216714	0.003742872	0.002680129	0.002155141	0.005736138	0.005399633
Collinsella	0.000185336	0.000320866	0.000527537	0.001008353	0.000547307	0.000340334	0.000205252	0.003143288	0.002111856
Colwellia	0	0	0	0	0	0	0	0	0
Comamona	0	0.000111606	0	0.000735825	8.83E-05	0	0.000102626	0	0
Coprobacillu	0	0.000125556	0.00012058	5.45E-05	0	8.51E-05	0	0.00108639	0
Coprobacte	0.000769856	0.001074204	0.025351943	0.000504176	0.000529652	0.001176988	0	0.000521467	0.000359976
Coprococcu	0	0.000362718	0.000165797	0.001035606	0.000229516	0.000113445	0.000161269	0.000912567	0.000347976
Coprococcu	0.000299388	0.002120506	0.001296235	0.002234728	0.003195565	0.000652306	0.000601094	0.001564401	0.000611958
Coprococcu	0.000242362	0.000976549	0.000587827	0.001648793	0.000370756	0.000269431	0.000952953	0.001071905	0.000899939
Coriobacter	0.000156822	0	0	0	0	0	0	0	0
Coxiella	0	0	0	0	0	0	0	0	0
Crocinitomix	0	0	0	0	0	0	0	0	0
Cyclobacter	0	0	0	0	0	0	0	0	0
Defluviitalea	0	0.000292965	0	0.000231649	8.83E-05	0.000155986	0	0.000217278	0.000155989
Deinococcu	0	0	0	0	0	0	0	0	0
Delftia	0	0	0	0	0	0	0	0	0
Desulfovibri	0.009466376	0.01078389	0.003903777	0.007167482	0.002153917	0.003559324	0.006040259	0.003172258	0.001859874
Dialister	0.000798369	0.000711486	0.00084406	0.061972829	0.006779541	0.003885478	0.002331071	0.003795121	0.015598939
Dielma	0	9.77E-05	7.54E-05	9.54E-05	0	8.51E-05	0	0	0.000431971
Dolosicoccu	0	0	7.54E-05	2.73E-05	0	0	0.000293216	0	0
Donghicola	0	0	0	0	0	0	0	0	0
Dorea	0.000727086	0.001618281	0.001251017	0.004523962	0.001518335	0.001120265	0.001935228	0.001767194	0.002459833
DTU089	0	0	0	0	0	0	0	0	0
Dubosiella	0	0	0	0	0	0	0	0	0
Dysgonomo	0	0	0.000165797	0	0	0	0	0	0
Eggerthella	0.000955192	0.000139507	0.00024116	0.000136264	0.000300136	0.000127625	0.004207656	0.000347645	0.000179988
Eisenbergie	0.000556007	0.00080914	0.00150725	0.000858463	0.000335446	0	0.000219912	0.005678197	0.000431971
Enhydrobac	0	0	0	0	0	0	0	0	0
Enterococcu	0	0.000446422	0.00174841	0.000272528	0	0.00097846	0.00035186	0	0.000179988
Enterorhabd	0.000826882	0	0	0	0	0	0	0	0
Epulopisciu	0	0	0	0	5.30E-05	0	0	0.000144852	0
Erysipelatoc	0.001910383	0.000878894	0.006737407	0.000654067	0.000582617	0.000326153	0.001510065	0.00515673	0.000971934
Erysipelotric	0.00062729	0.001422971	0.000648117	0.004360445	0.001306474	0.000808293	0.001510065	0.001955501	0.001871873
Erysipelotric	0	0	0	0	0.000194206	0	0	0	0
Escherichia-	0.014541722	0.009821292	0.005878275	0.022292777	0.019261666	0.079155972	0.322420795	0.034894837	0.055052256
Eubacterium	5.70E-05	0	0	0	0	0	4.40E-05	0	0
Ezakiella	0	0	0	0	0	0	0	0	0
Fabibacter	0	0	0	0	0	0	0	0	0
Faecalibact	0.007698559	0.034430323	0.024538028	0.024759154	0.012464469	0.094456813	0.011039599	0.013529173	0.09793734
Faecalibacu	0	0	0	0	0	0	0	0	0
Faecalicocc	0	0	0	0	0	0	0	0	0
Faecalitalea	9.98E-05	5.58E-05	0.000271305	0	0	0.000113445	0.000117287	4.35E-05	7.20E-05
Family XIII A	0	0.000153458	4.52E-05	0.00083121	0.000335446	0	0.000205252	0.000420071	0.002171852
Family XIII U	0	0.000181359	7.54E-05	0.00083121	0	0	0	0.000231763	0.000215985
Flavonifract	0.000413441	0.00039062	0.000994785	0.000817583	0.000370756	0.000865015	0.00017593	0.000651834	0.00425971
Formosa	0	0	0	0	0	0	0	0	0
Fournierella	0	0	0.000165797	0	0	0.003289894	0	0	0
Fusicateniba	0.000299388	0.004464223	0.000648117	0.002343739	0.000935718	0.000609765	0.000923632	0.002578365	0.00205186
Fusobacteri	0.00501832	0.008830792	0.002924065	0.000858463	0.003972387	0.003814575	0.008342008	0.000883597	0.000167989
GCA-90006	0	0.000125556	0	0.000163517	0.000158895	0.000226889	0	0.000275219	0.00058796
GCA-90006	0	0	0	0	0.000123585	0	0	0.000217278	0
Gemella	5.70E-05	0	0	0.000258901	8.83E-05	0	0.000117287	0	0.00014399
Gimesia	0	0	0	0	0	0	0	0	0
Gluconobac	0	0	0	0	0	0	0	0	0
Gordonibact	0	0	0	0	0	0	8.80E-05	0	6.00E-05
Gottschalkia	0	0	0	0	0	0	0.000190591	0	0
Granulicatel	0	0	0	0	0	0	0	0	0
Haemophilu	0	0.000111606	0.000572755	0.001226375	0.00010593	0	0.001040918	7.24E-05	0.001079927
Harryflintia	0	0	0	0	0	0.000808293	0	0	0
hgcI clade	0	0	0	0	0	0	0	0	0
Hoeflea	0	0	0	0	0	0	0	0	0
Holdemanel	0.000327902	0.000655683	0.004054502	0.020562225	0.000988683	0.000581404	0.001642012	0.000825656	0.002711816
Holdemania	0	6.98E-05	0	0.000299781	0.000176551	8.51E-05	0.000117287	0.000260734	0.000323978
Howardella	7.13E-05	0	0	0	0	0	0	0	0
Hungatella	0.000242362	0.000362718	0.001823772	0.000749452	0.000370756	0.002268892	0.000908971	0.000506982	0.0014759
Hydrogenoa	0.000228105	0.000139507	0	0.000245275	0.000176551	0.000411237	5.86E-05	0.00072426	0.001199918
Ileibacterium	0	0	0	0	0	0	0	0	0
Intestinibact	0.000441954	0.001213711	0.000437102	0.002834289	0.002153917	0.000283612	0.000996936	0.001897561	0.003815741
Intestinimon	0.000470467	0.000585929	0.000105507	0.000258901	0.000706202	0.000538862	0	0.000666319	0.000695953
Jeotgalibaca	0	0	0.000316522	0	0	0	0	0	0
Kordiimonas	0	0	0	0	0	0	0	0	0
Lachnoclost	0.015482657	0.005775589	0.017499171	0.007044844	0.012799915	0.027467775	0.003797153	0.005402978	0.007223509
Lachnoclost	0	0	0.000828987	0	0.000459031	0.000382876	0	0	0
Lachnoclost	0	0	0	0	0	0.000170167	0	0	0
Lachnospira	0.004533596	0.024413722	0.026829048	0.020357829	0.007291538	0.01009657	0.014880734	0.009531259	0.013151106
Lachnospira	0	0.000251113	0.000135652	0.000504176	0	0	0.00017593	0.000217278	0.00029998
Lachnospira	0.000213849	0.001185809	0.00042203	0.000790331	0.000441376	0.000411237	0.000454485	0.000521467	0.001055928
Lachnospira	0.003934819	0.00559423	0.019187291	0.01488002	0.004043008	0.004410159	0.003679866	0.002476969	0.008291436
Lachnospira	0.000128309	0.000976549	0.003933922	0.001757805	0.000847443	0.000425417	0.000747702	0.000535952	0.001055928
Lachnospira	0	0	0.00030145	0.000299781	0.000282481	0	0.001202187	0.000289704	0.000131991
Lachnospira	0.000741343	0.001422971	0.001401742	0.002098464	0.003407426	0.001035182	0.001451421	0.001709253	0.003227781
Lachnospira	0	0	0	0	0	0.000198528	0	7.24E-05	0
Lachnospira	0.00054175	0.00139507	0.006526392	0.001894068	0.00150068	0.000935918	0	0.002447998	0.000815945
Lacticigeniu	0	0	0.000150725	0	0	0	0	0	0
Lactobacillu	0.000256619	0.000362718	7.54E-05	0.001335386	0.000988683	0.000311973	0.035112668	0.00262182	0.000323978
Lactococcu	0	0.000362718	0	0	0	0	5.86E-05	0	0
Lentibacter	0	0	0	0	0	0	0	0	0
Leuconosto	0	5.58E-05	0	8.18E-05	0	0	0	0	0

Leucothrix	0	0	0	0	0	0	0	0	0
Litoricola	0	0	0	0	0	0	0	0	0
Mailhella	0.000199592	0	0	0	0	0	0.000117287	0	0
Mannheimia	0	0	0	0	0	0	0	0	0
Maribacter	0	0	0	0	0	0	0	0	0
Marinicella	0	0	0	0	0	0	0	0	0
Marinobacte	0	0	0	0	0	0	0	0	0
Marivita	0	0	0	0	0	0	0	0	0
Marvinbryar	0.000128309	5.58E-05	0	0.000286154	0	0	0	0	0
Massilia	0	0	0	0	0	0	0	0	0
Megamonas	0.012388977	0.001660133	0.001281162	0.000504176	0.018131742	0.001148627	0.002389714	0.00036213	0.001895871
Megasphae	0.106126057	0.005440772	0.00211015	0.012985951	0.011811232	0.002141267	0.005453826	0.004360044	0.007127515
Merdibacter	0	0	0	6.81E-05	0	0	0	0	0
Methanobre	0	0	4.52E-05	0.016378923	0	0.001786753	7.33E-05	2.90E-05	4.80E-05
Methylobact	0	0	0	0	0	0	0	0	0
Methylotene	0	0	0	0	0	0	0	0	0
Mf105b01	0	0	0	0	0	0	0	0	0
Micrococcus	0	0	0	0	0	0	0	0	0
Mitsuokella	0	0	0.000135652	0	0	0	0.004647481	0	4.80E-05
Mogibacteri	0	0	0	0.001076485	0.00014124	0	0	0.000101396	0.000191987
Morganella	0	0	0.00024116	0	0	0	0	0	0.000131991
Moryella	0	0.000320866	0.00012058	0.001280881	0.000123585	0	0.000131947	0.000188308	0.000179988
Murimonas	0	0	0	0	0	0	0	0	0
Mycobacter	0	0	0	0	0	0	0	0	0
Negativibac	0.000484724	0.000544077	0.000376812	0.001934948	0.000264826	0.000311973	0.000425164	0.000999479	0.000203986
Nitrosomona	0	0	0	0	0	0	0	0	0
Nosocomiic	0	0	0	0	0	0	0	0	0
NS3a marin	0	0	0	0	0	0	0	0	0
Oblitimonas	0	0	0	0	0	0	0.000131947	0	0
Oceanicocc	0	0	0	0	0	0	0	0	0
Oceanobact	0	0	0	0	0	0	0	0	0
Oceanospiri	0	0	0	0	0	0	0	0	0
Odoribacter	0.001069244	0.010225862	0.002366382	0.003338466	0.002507018	0.004466881	0.001744638	0.00446144	0.005279641
Olsenella	9.98E-05	0	0	0	0	0	0	0	0
OM27 clade	0	0	0	0	0	0	0	0	0
Oribacteriur	0	0	0	0	0	0	0	0	0
Oscillibacter	0.000413441	0.000669634	0.001899135	0.000940221	0.000264826	0.000950099	0.000102626	0.000767715	0.001403905
Oscillospira	0	0	0	0	0	9.93E-05	0	0	0
Ostreobium	0	0	0	0	0	0	0	0	0
Oxalobacter	0	0.0009905	0.00012058	0.000231649	0.000600272	0.000297792	0.000645076	0.000304189	6.00E-05
Paenalcalig	0	0	0	8.18E-05	0	0	0.00321072	5.79E-05	0
Paeniclostri	0	0	0	0	0	0	0	0	0
Parabacterc	0.044138403	0.031500677	0.041464444	0.030100699	0.026747409	0.048001248	0.033866499	0.055072716	0.040257263
Paraclostrid	0	0	0	0	0	0	0	0	0
Paracoccus	0	0	0	0	0	0	0	0	0
Parahaliea	0	0	0	0	0	0	0	0	0
Paralcaliger	0	0	0	0	0	0	0	0	0
Paraprevote	0.001439916	0.001771739	0.001431887	0.004047038	0.003866457	0.003928019	0.000615755	0.001941016	0.004679682
Parasuttere	0.001140527	0.002134457	0.002984355	0.003392972	0.001377094	0.003573505	0.00161269	0.002737702	0.0191147
Patulibacter	0	0	0	0	0	0	0	0	0
Pediococcu	0	0	0	0	0	0	0	0	0
Pedobacter	0	0	0	0	0	0	0	0	0
Peptoclostri	0	0	0	0	0.00010593	0	0	0	0
Peptococcu	0	0	0.000648117	0.000122638	0.000158895	0	0	8.69E-05	0
Phascolarct	0.010008126	0.017410471	0.001371597	0.00200308	0.007944775	0.001659127	0.024278321	0.006214149	0.001715883
Phocea	0	8.37E-05	0.000226087	0.000122638	0	0	7.33E-05	0	0.000227984
Planctomicr	0	0	0	0	0	0	0	0	0
Polynucleob	0	0	0	0	0	0	0	0	0
Porticoccus	0	0	0	0	0	0	0	0	0
Prevotella	0	0	0	0	0	0	0	0	0
Prevotella 2	0.001938896	0.001116056	0.001537395	0.008802649	0.001235854	0.00099264	0.001084901	0.000854627	0.001967866
Prevotella 6	0	0	0	0	0	0	0	0	0
Prevotella 7	0.000171079	5.58E-05	0.000271305	0.000749452	0.000229516	4.25E-05	0.000117287	5.79E-05	0.0044157
Prevotella 9	0.008169026	0.003655083	0.009977994	0.047910393	0.014000459	0.005927481	0.00178862	0.002361087	0.003587756
Prevotellace	0.000755599	0	0	0.000218022	0.000176551	0	8.80E-05	0.000144852	0
Prevotellace	0.000755599	0.001311366	0.000633045	0	0	0.000453778	0.000278556	0.000927053	0.000251983
Prevotellace	0	0	0	0.000231649	0	0	0	0	3.60E-05
Prevotellace	9.98E-05	5.58E-05	6.03E-05	0	0	0	0	0	0
Proteocatell	0	0	0.000165797	0	0	0	0	0	0
Proteus	0	0	0	0	0	0	0.000190591	0	0
Providencia	0	0	0	0	0	0	0	0	0
Pseudoalter	0	0	0	0	0	0	0	0	0
Pseudochro	0	0	0	0	0	0	0	0	0
Pseudoflavc	0	0	0	0	0	0	0	0	0
Pseudohonc	0	0	0	0	0	0	0	0	0
Pseudomon	0	0.000181359	9.04E-05	0.00034066	0.00014124	0	0.002301749	0.000101396	2.40E-05
Pseudophae	0	0	0	0	0	0	0	0	0
Pseudovibri	0	0	0	0	0	0	0	0	0
Pyramidoba	0.031720913	0.000627781	0.000452175	0.000613188	0.002612948	0.000411237	0.000395842	0.000478011	0.000215985
Raoultibacte	0	0	0	5.45E-05	0	0	0	0	0
Reichenbac	0	0	0	0	0	0	0	0	0
Rheinheime	0	0	0	0	0	0	0	0	0
Rhodococcu	0	0	0	0	0	0	0	0	0
Rhodoferax	0	0	0	0	0	0	0	0	0
Rikenellacea	0.000228105	0.00039062	0.00030145	0	0.000353101	0.000184347	0	0	0.000119992
Romboutsia	0.00067006	0.001911246	0.000527537	0.004660226	0.001818471	0.001517322	0.001319474	0.00298395	0.003515761
Roseburia	0.016494875	0.007142757	0.025487595	0.021693215	0.015730654	0.005814036	0.009661482	0.00624312	0.013343093
Roseibacillu	0	0	0	0	0	0	0	0	0
Rothia	0	2.79E-05	0	0	0	0	0	0	0
Ruegeria	0	0	0	0	0	0	0	0	0

Ruminiclost	0	0	9.04E-05	0	0	0.000340334	0	0	0
Ruminiclost	0	0	0	0.000109011	0	0	0	0	0.000791946
Ruminiclost	0.000527494	0.000906795	0.000859132	0.001975827	0.001147579	0.000425417	0.000659737	0.000927053	0.001643888
Ruminiclost	0	0.001618281	0.0006029	0.004210555	0.000794477	0.00148896	0.000483807	0.001607857	0.001967866
Ruminiclost	0.000427698	0.001757788	0.002486962	0.000313407	0.000688547	0.002311434	0.00035186	0.001071905	0.00088794
Ruminococ	0.001967409	0.020368019	0.007807554	0.008393857	0.004908105	0.006608148	0.003049451	0.012500724	0.005819604
Ruminococ	0.00192464	0.010881545	0.008350164	0.0132176	0.016631062	0.022107517	0.009441569	0.071513413	0.004823672
Ruminococ	0.000741343	0.001339267	0.000648117	0.001607914	0.003195565	0.000595584	0.001084901	0.001260212	0.003419767
Ruminococ	0.000598777	0.00039062	0.000256232	0.000558682	0.000229516	0.001446419	0.000498468	0.001042934	0.000371975
Ruminococ	0.001140527	0.005887195	0.00440117	0.007630779	0.005384792	0.004069825	0.004427568	0.022292717	0.024154358
Ruminococ	0	0	0	0.000136264	0	0.00048214	0	0	0
Ruminococ	5.70E-05	0.00119976	0.00078377	0.002125717	0.001306474	0.002127086	0.000835667	0.002476969	0.000599959
Ruminococ	0.00062729	0.001102105	0.006541464	0.002861542	0.001942056	0.001304613	0.001759299	0.001158816	0.002603823
Ruminococ	0.001496942	0.00520361	0.011907274	0.044858081	0.008721597	0.002268892	0.005673738	0.030896923	0.003179784
Ruminococ	0.002224028	0.003766689	0.003768125	0.007058471	0.00646175	0.00320481	0.002653609	0.003012921	0.00559162
Ruminococ	0.005374734	0.009877094	0.007882917	0.005150776	0.002630603	0.007841858	0.002565644	0.009241555	0.022090498
Sanguibacte	0	0	6.03E-05	2.73E-05	0	0.000198528	0.000161269	0	0
Sarcina	0	0	0	0	0	0	0	0	0
Sediminibac	0	0	0	0	0	0	0	0	0
Selenomona	0	0	0	0	0	0	0.000483807	0	0
Sellimonas	0	0	0.000376812	0	0	0	0	0	9.60E-05
Senegalima	0	0.000306915	0	8.18E-05	0.000194206	5.67E-05	0.000205252	0.003389536	0
Serratia	0	0	0	0	0	0	0	0	0
Shuttleworth	0	0	0	0	0	0	0	0	0
Slackia	0	0.000223211	0.00024116	6.81E-05	5.30E-05	0.000113445	5.86E-05	0.000101396	0
Sneathiella	0	0	0	0	0	0	0	0	0
Solobacteriu	2.85E-05	0	0.000105507	0.000136264	0	0	5.86E-05	0	0
Sphingobac	0	0	0	0	0	0	0	0	0
Sphingomor	0	0	0	0	0	0	0	0	0
Staphylococ	0	0	0	0	0	0	0	0	0
Stenotropho	0	0	0	0	0	0	0	0	0
Streptococc	0.001525455	0.003850393	0.006209869	0.006881328	0.001271164	0.00346006	0.001832603	0.001796164	0.008051453
Subdoligran	0.001397146	0.018052204	0.00518494	0.033125758	0.005967409	0.041690892	0.005629756	0.01034243	0.010235304
Succinatimo	0	4.19E-05	0	0	0	0	0	0	0
Succinivibrio	0	0	0	0	0	0	0	0	0
Sulfitobacte	0	0	0	0	0	0	0	0	0
Sutterella	0.014898137	0.031430923	0.013625539	0.003188576	0.018325948	0.008054567	0.002184462	0.007068776	0.001643888
Synergistes	0	0	0	0	0	0	0	0	0
Taeseokella	0	0	0	0	0	0	0	0	0
Tepidimicro	0	0	0	0	0	0	0.002037854	0	0
Terrisporob	0	0.000167408	0	0.000667693	0.000370756	0.000141806	0.000234573	0	0.000515965
Thalassotale	0	0	0	0	0	0	0	0	0
Thiopseudo	0	0	0	0	0	0	0	0	0
Tissierella	0	0	0	0.000572308	0.000723857	0	0.02300283	0.000506982	0
Turicibacter	0.000128309	0	0	0	0.000406066	0.000141806	0.000234573	0.00033316	0.001055928
Tyzzerella	0.000171079	0.000153458	0.00048232	0.000313407	0.000370756	0	0.00035186	0.000608378	0.000179988
Tyzzerella 3	5.70E-05	0.000669634	0.000256232	5.45E-05	0.000423721	0.000198528	0	0.000173822	0.000191987
Tyzzerella 4	0.000142566	0.000627781	0.001522322	0.000994727	0.000317791	0.001602405	0.000615755	0.001854105	0.00058796
UBA1819	0.002095719	0.00100445	0.0006029	0.000940221	0.000158895	0.001744211	0.000278556	0.000564923	0.00117592
uncultured	0.003393068	0.006082504	0.009420312	0.012086609	0.007591674	0.0059133	0.003723849	0.010429341	0.008399429
uncultured b	7.13E-05	0.001813591	0.000497392	0.102211563	0.006867817	0.000283612	0.002580305	0.023480503	0.007535488
uncultured b	9.98E-05	0	0.000256232	0	0	0.000212709	0	0.000637349	0
uncultured E	0	0	0	0	0	8.51E-05	0	0	0
uncultured E	0	0	0	0	0	0	0	0	0
uncultured C	0	0	0	0	0	0	0	0	0
uncultured C	0	0	0	5.45E-05	0	0	0.000322538	0	0
uncultured g	0	0	0	0	0	0	0	0	0
uncultured r	0	0	0	0	0	0	0	0	0
uncultured o	0.000940935	0.00259483	0.001959425	0.000994727	0.000706202	0.02292999	0.0014221	0.006880468	0.00014399
uncultured F	0.000456211	0.000376669	0.000467247	0	0	0.000269431	0	0	0
uncultured p	0	0	0	0	0	0	0	0	0
uncultured r	0	0	0	0	0.000882753	0	0.000263895	8.69E-05	0
uncultured S	0	0	0	0	0	0	0	0	0
uncultured T	0	0.000181359	0	0.000299781	0	0.000269431	0	0.001463005	0.000119992
uncultured V	0	0	0	0	0	0	0	0	0
unidentified	0	0	0.000874205	0.000286154	0.000317791	0	0.000190591	0.000304189	0
Veillonella	0.000798369	0.00020926	0.000889277	9.54E-05	0	0.000113445	0.000263895	0	0.000539963
Vibrio	0	0	0	0	0	0	0	0	0
Victivallis	0	0.000544077	0	0	0.001324129	0.000623945	0.000777024	0.000579408	0
W5053	0	0	0.000165797	0	0	0	0.000131947	0	0
Weissella	0	0	0.000150725	0.001076485	8.83E-05	0	0.000234573	0.000115882	0.00044397
Wenyingzhu	0	0	0	0	0	0	0	0	0
Woeseia	0	0	0	0	0	0	0	0	0
Genus	W32	W36	W38	W40	W45	W48	W49	W4	W51
[Caedibacte	0	0	0	0	0	0	0	0	0
[Clostridium	7.27E-05	0.000415488	0.000188407	0	0	9.59E-05	0	0.000120647	0
[Eubacteriu	0	0	0	0	0	0	0	2.68E-05	0
[Eubacteriu	0.024837495	0.006098296	0.025598191	0.005502257	0.005261347	0.000882545	0.004570488	0.037574734	0.001844714
[Eubacteriu	0.006951008	0.001125839	0.002348804	0.004077314	0.008730682	0.002916235	0.016919321	0.025134722	0.00122522
[Eubacteriu	0.000581674	0	0.001055078	0.000282167	0.000702469	0.001285446	0.000340657	0.001005389	0.00035793
[Eubacteriu	0	0.000321668	0	0	0	0	0.000326463	9.38E-05	0.000192731
[Eubacteriu	0.004158972	0.001005214	0.00252465	0.000804176	0.000802821	0.001554046	0.006685403	0.000214483	0.000798458
[Eubacteriu	0.001105181	0.000147431	0.000690825	0.001848194	0.00117556	0.000805802	0.005947312	0.002935736	0.000702093
[Eubacteriu	0.010033883	0.000214446	0.000753627	0.005064898	0.000501763	0	0.000369046	0.000951768	0.000192731
[Polaribacte	0	0	0	0	0	0	0	0	0
[Ruminococ	0	8.04E-05	0.000226088	0.000239842	0	0	0	0.000107241	0
[Ruminococ	0.000363546	0.002747584	0.001507254	0.004754515	0.001003527	0.065231572	0.001036166	0.000656854	0.001115088
[Ruminococ	0.001585063	0.002332096	0.006003894	0.005276524	0.003282966	0.005295269	0.004527906	0.00437009	0.00185848
Abiotrophia	0	0	0	4.23E-05	0	0	0	0	0

Acetanaerol	0	0.000241251	7.54E-05	2.82E-05	0	0	0	0	0
Acetitomacu	8.73E-05	0	0	0	0	0	0	0	0
Acetobacter	0	0	0	0	0	0	0	0	0
Acidaminoc	0.001236058	0.001769176	0.000778748	0.000310384	0.002107406	0	0.008928064	0.00142095	0.00094989
Acinetobact	0	0	0	0	0	0	0	0	0
Actinobacillu	0	0	0	0	0	0	0	0	0
Actinomyce	0.00015996	0.000107223	0.00056522	0.000197517	0.000186369	0.000230229	0.000312269	0.000241293	0.000192731
Adlercreutzi	0.000145419	0	0	9.88E-05	0.000114689	0	0	0	5.51E-05
Aeromonas	0.000101793	0.000227848	0.000226088	8.47E-05	0	0	0	0	0.00593337
Aerosphaer	0	0	7.54E-05	0.000112867	0	0	0	0	0
Aestuariicel	0	0	0	0	0	0	0	0	0
Agathobacte	0.006791048	0.001447508	0.008742071	0.004303047	0.007698483	0.000422087	0.004911145	0.007734792	0.00284967
Aggregatiba	0	0	0	0	0	0	0	0	0
Akkermansi	0.002137653	0.266810524	0.023010739	0	0.000286722	0	0.001234883	0.01662243	0.001390419
Alcaligenes	0	0	0	0	0	0.000249415	0	0	0
Alcanivorax	0	0	0	0	0	0	0	0	0
Alistipes	0.023775939	0.154213186	0.079947246	0.032406885	0.026005677	0.005352826	0.171194572	0.023955066	0.095167952
Allisonella	0.000421714	0.000160834	0.000376813	0.000479684	0.00058778	0.000287786	0.001107137	0.000348535	0.000165198
Allobaculum	0	0	0	0	0	0	0	0	0
Alloprevotel	0.000741635	0	0	0.002031603	0.000157697	0	0	0.001179656	0
Allorhizobiu	0	0	0	0	0	0.000211043	0	0	0
Alloscardov	0	0	0.000138165	0	0	0	0	0	0
Amphritea	0	0	0	0	0	0	0	0	0
Anaerofilum	0	0	0	0	0	0	0	0	0
Anaeroglobu	0	0	0	0	0.000129025	0	0	0	0
Anaerospor	0.001788649	0	0.000175846	0	0	0	0.000425822	0.001487976	0
Anaerostipe	0.000450798	0.000495905	0.000427055	0.000366817	0.000473091	0.003760408	0.000283881	0.00075069	0
Anaerotrunc	0.000247212	0.000710351	0.000427055	9.88E-05	0.000172033	0.0001343	0.000170329	0.000281509	0.000247797
Angelakisell	0	0.000134028	0.000100484	7.05E-05	0	0	0.000113552	0	0
Anoxybacillu	0	0	0	0	0	0	0	0	0
Anseongella	0	0	0	0	0	0	0	0	0
Arenibacter	0	0	0	0	0	0	0	0	0
Atopobium	0	0	7.54E-05	0	0.000301058	0	0.000156135	0.000107241	8.26E-05
Atopostipes	0	0	0	0	0	0	0.000113552	0	0
Aureimarina	0	0	0	0	0	0	0	0	0
Azospirillum	0.000305379	0.020626985	0.000502418	0	0	0	7.10E-05	0	0.000412996
Bacillus	0	0	0	0	0	0	0	0	0
Bacteroides	0.442247008	0.289528354	0.372027884	0.599619074	0.524615076	0.462760447	0.219255663	0.169709644	0.501844714
Balneola	0	0	0	0	0	0	0	0	0
Barnesiella	0.005075109	0.000308266	0.002122716	0.000931151	0.000860166	0.000249415	0.003562709	0.002694442	0.000344163
Bifidobacter	0	0	0.001482133	0.000112867	0.008759354	0.000191858	0.000184523	0.001005389	0.000302863
Bilophila	0.001963151	0.010507834	0.001997111	0.001721219	0.000731141	0.000153486	0.001263271	0.002614011	0.003496696
Blautia	0.02206	0.004355926	0.007649312	0.009424379	0.004960289	0.01876367	0.006273775	0.005925092	0.003125
Bosea	0	0	0	0	0	0	0	0	0
Brevibacillus	0	0	0	0	0	0	0	0	0
Brevundimo	0	0	0	0	0	0	0	0	0
Butyricicocc	0.00101793	0.000522711	0.001469572	0.001834086	0.002265103	0.008384176	0.001206495	0.001260087	0.000894824
Butyricimon	0.015138075	0.000428891	0.004308233	0.016055305	0.005548069	0	0.008686765	0.008284404	0.003661894
Butyrivibrio	0	0	0.000113044	0	0	0	0	0	0
C1-B045	0	0	0	0	0	0	0	0	0
CAG-352	0	0	0	0	0	0	0.000283881	0.000174267	0
CAG-56	0.00023267	0.000308266	0.000200967	0.00025395	0.000215041	0	0.000553568	0.000254699	0.002656938
CAG-873	0.00039263	0	0	0	0	0	0	0	0
Campylobac	0	0	0	0	0	0	0	0	0
Candidatus	0	0	0	0	0	0	0	0	0
Candidatus	0	0	0	0	0	0	0	0	0
Candidatus	0	0	0	0	0	0	0	0	0
Candidatus	0	0	0	0	0	0	0	0	0
Candidatus	0	0	0	0	0	0	0	0	0
Candidatus	7.27E-05	4.02E-05	0.000389374	0	0	0	0	0.001072415	0
Candidatus	0	9.38E-05	0	0.000112867	0	0	9.94E-05	0	0
Caproicipro	0	0	0	0	0	0	0	0	0
Catabacter	8.73E-05	0	0.000113044	5.64E-05	0	0	0	0	0
Catenibacte	0	0	0	0	0.000172033	0	0	0.00105901	0
Cellulosilytic	0	0	0	0	0	0.002359848	0	0	0
Cellvibrio	0	0	0	0	0	0	0	0	0
Cephalotico	0	0	0	0	0	0	0	0	0
Christensen	0	0	0	0	0	0	0	0	0
Christensen	0.010542848	0.001943413	0.004923695	0.002863995	0.003541016	0.000901731	0.02184466	0.028150889	0.002271476
Chryseoline	0	0	0	0	0	0	0	0	0
Chthoniobac	0	0	0	0	0	0	0	0	0
Clavibacter	0	0	0	0	0	0.000402901	0	0	0
Cloacibacillu	0	0	0	0	0	0	0	0	0
Clostridiales	5.82E-05	0	0	0	0	0	0	0.000308319	0
Clostridioide	0	0.000268057	0	0	0	0	0	0	0
Clostridium	0.005089651	0.000629934	0.002373925	0.003371896	0.004243484	0.067342005	0.001987169	0.001045604	0.001555617
Collinsella	0.000552591	0.000308266	0.002361364	0.000282167	0.00065946	0.000556387	9.94E-05	0.000978579	0.000110132
Colwellia	0	0	0	0	0	0	0	0	0
Comamona	0	0	0	9.88E-05	0.000114689	0	0.000227105	0.001501381	0
Coprobacillu	0	0	0.000163286	0	7.17E-05	0	0	0.000844527	0
Coprobacte	0.00023267	0.000147431	0.001884067	0.002624153	0.000372738	0	0.000567762	0.000857932	0.000578194

Coprococcu	0.000189044	0.000120626	0.00028889	0.000112867	0.000243714	0	0	0.000911553	5.51E-05
Coprococcu	0.002966539	5.36E-05	0.000577781	0.000648984	0.000215041	0	0.000283881	0.000804311	0
Coprococcu	0.000334463	0.000321668	0.000728506	0.000352709	0.000444419	0.000575573	0.000851644	0.000898147	0.000234031
Coriobacter	0	8.04E-05	0	0	0	0	0	0	0
Coxiella	0	0	0	0	0	0	0	0	0
Crocinitomix	0	0	0	0	0	0	0	0	0
Cyclobacter	0	0	0	0	0	0	0	0	0
Defluviitalea	0	0	0	0	0	0	0	0.000294914	0
Deinococcu	0	0	0	0	0	0	0	0	0
Delftia	0	0	0	0	0	0	0	0	0
Desulfovibri	0.000698009	0.001179451	0.003994222	0.000902935	0.001060871	0.000326158	0.001632317	0.002975951	0.004859581
Dialister	0.004522518	0.001728968	0.003064749	0.007759594	0.002852883	0.001208703	0.00090842	0.004088581	0.000647026
Dielma	5.82E-05	0	0.000125604	5.64E-05	0	3.84E-05	2.84E-05	5.36E-05	0
Dolosicoccu	0	0	0	0	0	0	0	0	0
Donghicola	0	0	0	0	0	0	0	0	0
Dorea	0.003170125	0.002224873	0.002009672	0.000832393	0.00124724	0.021603162	0.000723897	0.001487976	0.000564427
DTU089	0	0.00018764	0	0	0	0	0	0	0
Dubosiella	0	0	0	0	0	0	0	0	0
Dysgonomo	0	0.000696948	6.28E-05	0	0	0	0	2.68E-05	8.26E-05
Eggerthella	0.000145419	0.000897991	0.000125604	0	7.17E-05	0	0.000496792	4.02E-05	0.000261564
Eisenbergie	0.000290837	0.004637386	0.002625133	0.000747743	0.000157697	0	0	0.00183651	0.000454295
Enhydrobac	0	0	0	0	0	0	0	0	0
Enterococcu	0	0	0.00027633	0	0	0	0.000212911	0.000160862	0
Enterorhabd	0	0.00018764	0	0	0	0	0	0	8.26E-05
Epulopisciu	0	0	0	0	0	0.000249415	0	0	0
Erysipelatoc	0.000741635	0.042017933	0.002122716	0.000959368	0.000458755	0.000306972	0.000212911	0.000442371	0.001390419
Erysipelotric	0.001163349	0.000897991	0.000979715	0.000634876	0.000860166	0.010130079	0.00083745	0.000402156	0.001238987
Erysipelotric	7.27E-05	0	0	0	0	0	0	0	0
Escherichia-	0.004406183	0.015158623	0.061923004	0.011766366	0.022636695	0.007923717	0.013044342	0.07929167	0.025894824
Eubacterium	0	0.000120626	0	0	0	0	0	0	0
Ezakiella	0	0	0	0	0	0	0	0	0
Fabibacter	0	0	0	0	0	0	0	0	0
Faecalibact	0.013567554	0.006379756	0.009005841	0.019836343	0.019382401	0.034054718	0.024413785	0.072669509	0.013560022
Faecalibacu	0	0	0	0	0	0	0	0	0
Faecalicocc	0	0	0.000389374	0	0	0	0	0	0
Faecalitalea	0	0	0.000100484	0.000141084	0	0.000172672	8.52E-05	6.70E-05	0.000234031
Family XIII A	0.002210363	6.70E-05	0.000364253	0.000126975	0	7.67E-05	0.000113552	0.00072388	0
Family XIII U	0.000130877	0	8.79E-05	0.000183409	0.000401411	0	0.000113552	0.000227888	0.000123899
Flavonifract	0.001381477	0.001541328	0.000741066	0.00153781	0.00025805	0.000230229	0.000283881	0.000321724	0.000440529
Formosa	0	0	0	0	0	0	0	0	0
Fournierella	0	0	0	0	0	0	0	0.000442371	0
Fusicateniba	0.00101793	0.000294863	0.001092759	0.000874718	0.002050062	0.015271862	0.000695509	0.00108582	0.000344163
Fusobacteri	0.000988846	0.002265082	0.000954594	0.003781038	0.003282966	0	0.008033839	0.00506716	0.161935573
GCA-90006	0	0	0	0	0	0	0.000354852	0.000254699	0
GCA-90006	0	0	0	0	0	0	0	0.000281509	0
Gemella	5.82E-05	9.38E-05	0	0.000141084	0	0.000402901	0.000113552	0	0.000536894
Gimesia	0	0	0	0	0	0	0	0	0
Gluconobac	0	0	0	0	0	0	0	0	0
Gordonibact	0	0	0	0	0	0	0	6.70E-05	0
Gottschalkia	0	0	0	0	0	0	0	0	0
Granulicatel	0	0	0	8.47E-05	8.60E-05	0.000613944	0	0	0.000178965
Haemophilu	0.000349005	0.000294863	0.000489857	0.000719526	0.00837228	0.007194659	0.001135525	0.000536207	0.000261564
Harryflintia	0	0	0	0	0	0	0	0	0
hgcI clade	0	0	0	0	0	0	0	0	0
Hoeflea	0	0	0	0	0	0	0	0	0
Holdemanel	0.000799802	0.000696948	0.091452616	0.000959368	0.000487427	0.000153486	0.001178107	0.007775007	0
Holdemania	0.000305379	0	0	0.000451467	7.17E-05	9.59E-05	7.10E-05	0.000201078	6.88E-05
Howardella	0	0	0	0	7.17E-05	0	0	0.000482587	0
Hungatella	0	0.000884588	0.001582616	0.002003386	0.000286722	0	0.000298075	0.002345907	0.000220264
Hydrogenoa	0	0	0.000113044	7.05E-05	0	0	0.000198717	0.000214483	0
Ileibacterium	0	0	0	0	0	0	0	0	0
Intestinibact	0.005336862	0.00164855	0.001733342	0.002878104	0.000946182	0.001745904	0.000723897	0.000683664	0.000385463
Intestinimor	0.000799802	0.000227848	0.000389374	0.000846501	0	0	0.000227105	0	0.00027533
Jeotgalibaca	0	0	0	0	0	0	0	0	0
Kordiimonas	0	0	0	0	0	0	0	0	0
Lachnoclost	0.01150261	0.029405852	0.006832883	0.013854402	0.006379562	0.037373854	0.005478908	0.004665004	0.006401432
Lachnoclost	0.000319921	0	0.00055266	0.000225734	0.00032973	0	0.000354852	0	0.000192731
Lachnoclost	0	0	0.000150725	0	0	0	0	0	0
Lachnospira	0.011473527	0.00442294	0.013087986	0.019963318	0.015626344	0.092897433	0.015755408	0.011796563	0.00375826
Lachnospira	0.000843428	0	0.000238648	8.47E-05	0.000129025	0.000115115	0.000340657	0.000201078	0
Lachnospira	0.000872512	8.04E-05	0.000314011	0.000225734	0.000602116	0.000383715	0.00052518	0.001353924	0.000165198
Lachnospira	0.002326697	0.001621745	0.004333354	0.012570542	0.007985205	0.001323817	0.011454607	0.078514169	0.010421256
Lachnospira	0.0031265	0.00028146	0.001620298	0.000846501	0.001003527	0	0.001050361	0.001005389	0.000302863
Lachnospira	8.73E-05	0	0.000326572	0.000310384	0.000401411	0	0.008076421	8.04E-05	0.000123899
Lachnospira	0.002384865	0.000804171	0.00111788	0.000846501	0.001462282	0.002686006	0.000965196	0.003002761	0.000729626
Lachnospira	0	0	0	0	0	0	0	0	0
Lachnospira	0.001163349	0.000482503	0.001745902	0.000846501	0.001017863	0.000479644	0.001163913	0.001849916	0.001156388
Lacticigeniu	0	0	8.79E-05	4.23E-05	0	0	0	0	0
Lactobacillu	0	0	0.000665704	9.88E-05	0.001032199	0.001496489	0.004244024	0.000402156	0.000165198
Lactococcus	0	0	0	0	0	0	9.94E-05	6.70E-05	0
Lentibacter	0	0	0	0	0	0	0	0	0

Leuconosto	0	0	0	0	0	0.000191858	2.84E-05	2.68E-05	0
Leucothrix	0	0	0	0	0	0	0	0	0
Litoricola	0	0	0	0	0	0	0	0	0
Mailhella	0	0.00018764	0	8.47E-05	2.87E-05	0	0	0	0.003069934
Mannheimia	0	0	0	0	0	0	0	0	0
Maribacter	0	0	0	0	0	0	0	0	0
Marinicella	0	0	0	0	0	0	0	0	0
Marinobacte	0	0	0	0	0	0	0	0	0
Marivita	0	0	0	0	0	0	0	0	0
Marvinbryar	0	0	0	0	0	0	0	0.000174267	0
Massilia	0	0	0	0	0	0.000402901	0	0	0
Megamonas	0.017275728	0.001367091	0.001256045	0.002511287	0.004859936	0	0.005124056	0.00289552	0.00280837
Megasphae	0.008027106	0.006151908	0.002449287	0.003287246	0.015698024	0.000518015	0.028530063	0.00796268	0.00530011
Merdibacter	0	6.70E-05	0	0	0	0	0	0	0
Methanobre	0	0	0	0	0	0	5.68E-05	0.000174267	0
Methylobact	0	0	0	0	0	0.000422087	0	0	0
Methylotene	0	0	0	0	0	0	0	0	0
Mf105b01	0	0	0	0	0	0	0	0	0
Micrococcus	0	0	0	0	0	0	0	0	0
Mitsuokella	0	0	0	0.002341986	0.00235112	0.000786616	0.133537728	0	0.001197687
Mogibacteri	0	0	0	0	0	0	0	8.04E-05	0
Morganella	7.27E-05	0.000120626	0.005275388	0	0	0	0	0	0
Moryella	0.000247212	0	0	0.000211625	0.000114689	0	0.000156135	0.000120647	0
Murimonas	0	0	0	0	0	0	0	0	0
Mycobacter	0	0	0	0	0	0	0	0	0
Negativibac	0.000189044	0.006889065	0.000213528	0.000141084	0	0	0.000638733	0.000348535	0.000330396
Nitrosomon	0	0	0	0	0	0	0	0	0
Nosocomiic	0	0	0	0	0	0	0	0	0
NS3a marin	0	0	0	0	0	0	0	0	0
Oblitimonas	0	0	0	0	0	0	0	0	0
Oceanicocc	0	0	0	0	0	0	0	0	0
Oceanobact	0	0	0	0	0	0	0.000141941	0	0
Oceanospiri	0	0	0	0	0	0	0	0	0
Odoribacter	0.000974305	0.000790768	0.013326634	0.002581828	0.003555352	0.000230229	0.010191336	0.007842033	0.0094163
Olsenella	0	0	0	0	0	0	4.26E-05	0	0
OM27 clade	0	0	0	0	0	0	0	0	0
Oribacteriur	0	0	0	0	0	0	0	0	0
Oscillibacter	0.000814344	0.000817574	0.000439616	0.002102144	0.000473091	0.000115115	0.000269687	0.00075069	0.000371696
Oscillospira	0	0	0	0	0	0	0	0	0
Ostreobium	0	0	0	0	0	0.000940102	0	0	0
Oxalobacter	0.000814344	0	0.000314011	0	0	0	0	0.00038875	6.88E-05
Paenalcalig	0	0	0	0	0	0	0.000170329	0	0
Paeniclostri	0.000174502	0	0	0	0	0.001055217	0	0	0
Parabacterc	0.045210639	0.025170551	0.060742322	0.03236456	0.026923187	0.010379494	0.01761483	0.019424113	0.026927313
Paraclostrid	0	0	0	0	0	0	0	0	0
Paracoccus	0	0	0	0	0	0	0	0	0
Parahaliea	0	0	0	0	0	0	0	0	0
Paralcaliger	0	0	0	0	0	0	0	0	0
Paraprevote	0.011909782	0.00084438	0.003177793	0.010228555	0.001605643	0	0.001547153	0.000469181	0.001004956
Parasuttere	0.003301002	0.002908418	0.004509201	0.00376693	0.005232675	0.000479644	0.002370408	0.004222633	0.001789648
Patulibacter	0	0	0	0	0	0.000287786	0	0	0
Pediococcu	0	0	0	0	0	0	0	0	0
Pedobacter	0	0	0	0	0	0.000460458	0	0	0
Peptoclostri	0	0	0	0	0	0	0	0	0
Peptococcu	5.82E-05	0	5.02E-05	0.000409142	4.30E-05	0	4.26E-05	0	9.64E-05
Phascolarct	0.010920936	0.00193001	0.002022232	0.005347065	0.013031511	0.000115115	0.004130472	0.008699965	0.009581498
Phocea	0	0	0	0	0	0	0	0	9.64E-05
Planctomicr	0	0	0	0	0	0	0	0	0
Polynucleob	0	0	0	0	0	0	0	0	0
Porticoccus	0	0	0	0	0	0	0	0	0
Prevotella	0	0	0	0	0	0	0	0	0
Prevotella 2	0.000145419	0.000603128	0.002122716	0.001678894	0.001261576	0	0	0.011086088	0.000412996
Prevotella 6	0	0	0	0	0	0	0	0	0
Prevotella 7	0.000101793	0	0.000138165	0.000423251	0.000788485	0	0	0.001032199	0
Prevotella 9	0.00571495	0.001125839	0.007372982	0.009706546	0.004530206	0.002340662	0.002200079	0.011126304	0.005768172
Prevotellace	0	0.000549517	0	0	0	0	0	0	0.000674559
Prevotellace	0.000276295	0.000227848	0.000238648	0	0	9.59E-05	0.001149719	0.000777501	0
Prevotellace	0	0	0	0	0	0	0	0	0
Prevotellace	0	0	0	0	5.73E-05	0	0	0.000924958	0
Proteocatell	0	0	8.79E-05	0	0	0	0	0	0
Proteus	0	0	0	8.47E-05	0.000100353	0	0.005280191	0	0
Providencia	0	0	0	0	0	0	0	0	0
Pseudoalter	0	0	0	0	0	0	0	0	0
Pseudochro	0	0	0	0	0	0	0	0	0
Pseudoflavo	0	0	0	0	0	0	0	0	0
Pseudohong	0	0	0	0	0	0	0	0	0
Pseudomon	4.36E-05	0	0.000251209	0.000239842	0	0.001822647	0.000198717	0	0
Pseudophae	0	0	0	0	0	0	0	0	0
Pseudovibri	0	0	0	0	0	0.000268601	0	0	0
Pyramidoba	0.000290837	0.001554731	0.000100484	0	0.000344066	0	0.000354852	0.001179656	0.001087555
Raoultibacte	0	0	0	0	0	0	0	0	0

Reichenbac	0	0	0	0	0	0	0	0	0
Rheinheime	0	0	0	0	0	0	0	0	0
Rhodococcu	0	0	0	0	0	0.000287786	0	0	0
Rhodoferax	0	0	0	0	0	0	0	0	0
Rikenellace	0.002879288	2.68E-05	3.77E-05	5.64E-05	0.001046535	0	0	0.021193598	0
Romboutsia	0.001846816	0.000951602	0.001570056	0.001678894	0.000946182	0.018015425	0.00090842	0.000831121	0.000289097
Roseburia	0.023732313	0.003524949	0.007523708	0.025761851	0.009404479	0.003760408	0.008530631	0.00977238	0.006456498
Roseibacillu	0	0	0	0	0	0	0	0	0
Rothia	0	0	0	0	0	0	0	0	0
Ruegeria	0	0	0	0	0	0	0	0	0
Ruminiclost	0	0.000227848	0	0.000141084	0	0	0	0	0
Ruminiclost	0	0	0	0	0	0	0	0	0
Ruminiclost	0.00085797	0.004503357	0.000602901	0.001932844	0.000544772	0.000326158	0.000709703	0.000630044	0.00063326
Ruminiclost	0.00578766	0.000254654	0.001570056	0.000465576	0	0	0.002980753	0.000308319	0
Ruminiclost	0.002573909	0.00065674	0.00111788	0.001086343	0.000530436	0	0.000482598	0.001045604	0.000468062
Ruminococ	0.022278128	0.001018617	0.007774917	0.001876411	0.005519397	0.002283105	0.007664793	0.01158208	0.001624449
Ruminococ	0.025942676	0.00249293	0.006229982	0.004049097	0.007755828	0.006216185	0.066059161	0.034223438	0.007819383
Ruminococ	0.010077508	0.000804171	0.000929473	0.000902935	0.001734668	0.000422087	0.002072333	0.001514786	0.005947137
Ruminococ	0.000349005	0.00028146	0	0.00025395	0	0.000115115	0.00045421	0.000107241	0.000206498
Ruminococ	0.0195588	0.000924797	0.005677322	0.002948646	0.004601887	0.001170331	0.03764265	0.045483793	0.001018722
Ruminococ	0	0	0	0	0	0	0	0	0
Ruminococ	0.000698009	0.000147431	0.000728506	0.000465576	0.000903174	7.67E-05	0.002498155	0.007734792	5.51E-05
Ruminococ	0.003475504	0.000790768	0.002650254	0.001594244	0.001304585	0	0.000695509	0.001353924	0.000399229
Ruminococ	0.002094028	0	0.005514036	0.002412528	0.003454999	0.001784275	0.000127747	0.047159441	0
Ruminococ	0.01478907	0.001862996	0.002951705	0.010045147	0.037187832	0.000748245	0.003406575	0.010898416	0.003359031
Ruminococ	0.00210857	0.002908418	0.002838661	0.004500564	0.0029389	0.001247074	0.00234202	0.005013539	0.005520374
Sanguibacte	0	0	6.28E-05	0	0	0	7.10E-05	0	0
Sarcina	0	0	0	0	0	0	0	0	0
Sediminibac	0	0	0	0	0	0	0	0	0
Selenomona	0	0	0	0.000324492	0.000372738	0	0.015003123	0	0.000137665
Sellimonas	0	0.000134028	0	0	0	0	0	0	0
Senegalima	0	0	0	0	0	0	8.52E-05	0.000281509	0
Serratia	0	0	0	0	0	0	0	0	0
Shuttleworth	0	0	0	0	0	0	0	0	0
Slackia	5.82E-05	0	0.000477297	0	8.60E-05	0	0	0.000630044	0
Sneathiella	0	0	0	0	0	0	0	0	0
Solobacteriu	0	0	0	5.64E-05	0.000172033	7.67E-05	0	0.000764096	2.75E-05
Sphingobac	0	0	0	0	0	0	0	0	0
Sphingomor	0	0	0	0	0	0.000326158	0	0	0
Staphylococ	0	0	0	0	0	0	0	0	0
Stenotropho	0	0	0	0	0	0.003146464	0	0	0
Streptococc	0.002922914	0.001956816	0.044401181	0.004133747	0.002150414	0.009151606	0.002228468	0.002681037	0.000702093
Subdoligran	0.008361569	0.001246465	0.009734347	0.003710497	0.006709293	0.001669161	0.00408789	0.017721655	0.001073789
Succinatimo	0	0	0	0	0	0	0	0	0
Succinivibri	0	0	0	0	0	0	0	0	0
Sulfitobacte	0	0	0	0	0	0	0	0	0
Sutterella	0.006005788	0.002318693	0.004710168	0.007886569	0.066648508	0.008576033	0.003037529	0.011086088	0.011261013
Synergistes	0	0	0	0	0	0	0	0	0
Taeseokella	0	0	0	0	0	0	0	0	0
Tepidimicro	0	0	0	0	0	0	0.000113552	0	0
Terrisporob	0.000450798	0	0.000113044	0.000225734	0.000172033	0.000460458	0	0.000428966	0
Thalassotal	0	0	0	0	0	0	0	0	0
Thiopseudo	0	0	0	0	0	0	0	0	0
Tissierella	0	0	0	0	0	0	0.001092943	0	0
Turicibacter	0.000218128	0.000120626	0	0.000409142	4.30E-05	0.010552166	0	5.36E-05	0
Tyzzerella	0.000101793	0.000268057	0	0	0	0	0	0.000174267	0
Tyzzerella 3	0.000189044	0.000134028	0.000263769	0	4.30E-05	0.00126626	0.000127747	5.36E-05	0.000110132
Tyzzerella 4	0.000494423	0	0.000364253	0.002835779	0.000172033	0.000249415	0.000510986	0.000227888	0.000165198
UBA1819	0.001134265	0.015507097	0.001004836	0.000606659	0.000243714	0	0.000212911	0.000616639	0.003950991
uncultured	0.004551602	0.004690997	0.004835772	0.019441309	0.004157468	0.016921837	0.009396469	0.008981474	0.003097467
uncultured b	0.03395524	0.000455697	0.001620298	0.003879797	0.000143361	0	0.001305854	0.002091209	0
uncultured b	0	0	0.000125604	0	0.000530436	0	0	0.005603367	0
uncultured E	0	0	0	0	0	0	0	0	0
uncultured E	0	0	0	0	0	0	0	0	0
uncultured C	0	0	0	0	0	0	0	0	0
uncultured C	0	0	0	0	0	0	0	0	0
uncultured g	0	0	0	0	0	0	0	0	0
uncultured r	0	0	0	0	0	0	0	0	0
uncultured o	0	0	0.000251209	0.000423251	0.001892365	7.67E-05	0.00786351	0.01664924	0.000151432
uncultured F	0	0	0	0	0.000530436	0	0	0.005160996	0
uncultured p	0	0	0	0	0	0	0	0	0
uncultured r	0.022990679	0.000201043	0.000138165	0	0	0	0	0.000281509	0
uncultured S	0	0	0	0	0	0	0	0	0
uncultured T	0	0	0	0	0	0	0	0	0
uncultured V	0	0	0	0	0	0	0	0	0
unidentified	0	0	0.000665704	0	0	0	0	0	0
Veillonella	0.000261753	0.00738497	0.000628022	0.000973476	0.038263039	0.000191858	0.001490376	0.001662243	0.001197687
Vibrio	0	0	0	0	0	0	0	0	0
Victivallis	0	0	0	0	7.17E-05	5.76E-05	0.000269687	0.001045604	0
W5053	0	0	0.000100484	0	0	0	2.84E-05	0	0
Weissella	4.36E-05	6.70E-05	0.000100484	0.000239842	0	0	9.94E-05	8.04E-05	0

Wenyingzhu	0	0	0	0	0	0	0	0	0
Woeseia	0	0	0	0	0	0	0	0	0

Genus	W54	W57	W59	W61	W63	W65	W66	W68	W70
[Caedibacte	0	4.74E-05	0	0	0.000189003	0	0	0	0
[Clostridium	0	7.91E-05	0.000282692	4.90E-05	0	0	2.71E-05	0	0
[Eubacteriu	0	0	0	0	0	0.000112408	5.42E-05	0	0
[Eubacteriu	0.008620472	0.015736201	0.016559789	0.009650403	0.027710738	0.011738635	0.039781655	0.014322438	0.008308928
[Eubacteriu	0.008911191	0.009267753	0.007632679	0.003114615	0.00409991	0.005395597	0.013111557	0.004973069	0.015173401
[Eubacteriu	0.000922719	0.001075439	0.003600601	0.000465966	0.00066878	0.000337225	0.00186921	0.001193537	0.001166165
[Eubacteriu	0	0	0.000327327	0.000858359	0.000159926	0.000128467	0	0	0
[Eubacteriu	0.003766716	0.002198324	0.003035217	0.001201702	0.001671949	0.011032068	0.030124072	0.007008202	0.000927631
[Eubacteriu	0.007204793	0.000790764	0.001339067	0.000208459	0.00055247	0.000915324	0.000555345	0.027895091	0.003021428
[Eubacteriu	0.001617918	0.000790764	0.002157385	0.000465966	0.002340729	0.001364958	0.006758953	0.001729098	0.000450564
[Polaribacte	0.000682559	0.000521904	0.000312449	0	0.000974092	0.001124083	0	0.000566165	0.000543327
[Ruminococ	0	0	0.001487852	0	0	0.000240875	0.00029799	0	0
[Ruminococ	0.002667037	0.001739681	0.008644418	0.010852105	0.00287866	0.01637949	0.001977569	0.006074795	0.003723778
[Ruminococ	0.009088151	0.0034161	0.004686733	0.001483734	0.002297113	0.00345254	0.008235358	0.007390746	0.00479718
Abiotrophia	0	0	0	0	0	0	0	0	0
Acetanaerol	0	0	0	0	0	3.21E-05	0	0	0
Acetitomacu	0	0.000142337	0	0	0.000130848	0	0	0.000137716	0
Acetobacter	0	6.33E-05	0.001443216	0	7.27E-05	9.63E-05	0	0	0
Acidaminoc	0	0.00026886	0	0	0	0	0	0	0
Acinetobact	0.00020224	0.003811482	0.006710211	0	0.000567009	0.000144525	0	0.000244828	0.002279323
Actinobacillu	0	0	0	0	0	0	0	0	0
Actinomyce	0.00021488	0.000237229	0.00080344	0.000355606	0.000348928	0.000321166	0.000148995	7.65E-05	0.000225282
Adlercreutzi	0.0002528	0.000158153	0.000208299	0.00011036	0.000290774	0	0	0	6.63E-05
Aeromonas	0.00035392	0.001328483	0.001220038	0.000331081	0.001933645	0.001043791	0.000690795	0.000153018	0
Aerosphaer	0	0	0	0	0	0	0	0	0
Aestuariicel	0	0	0	0	0	0	0	0	2.65E-05
Agathobact	0.042950679	0.011893089	0.012646739	0.003715466	0.006571487	0.005122605	0.011919597	0.011369201	0.049959582
Aggregatiba	0	0	0.000133907	0	0	0	0	0	0
Akkermansi	0.001478879	0.065048237	0.037984854	0.037657417	0.000974092	0.038090344	0.001205505	0.000474354	0.001656485
Alcaligenes	0.00011376	0	0	9.81E-05	0	0	0	0	0
Alcanivorax	0	0	0.000163664	0	0	0	0	0	0
Alistipes	0.035771166	0.04815752	0.014075077	0.057240255	0.05206304	0.024777994	0.023270304	0.093646713	0.023363062
Allisonella	0	0.000253044	0.000654655	7.36E-05	0	0	0.00029799	0	0.000304793
Allobaculum	0	0	0.001220038	8.58E-05	0	8.03E-05	0	0	0
Alloprevotel	6.32E-05	0.000189783	0	0	0	0.003853998	0	0.000550863	0.000556579
Allorhizobiu	0	0	0	0	0	0	0	0	0
Alloscardov	0	0	0	0	0	0	0	0	0
Amphritea	0	0	0	0	0	0.000529925	0	0	0
Anaerofilum	0	0	0	0	0	0	0	0	0
Anaeroglob	0	0	0	0	0	0	0	0	0.000344549
Anaerospor	0.000834239	0	0.000743926	0.001495996	0.003227589	0.000256933	0	0.000596768	0.000755357
Anaerostipe	0.000631999	0.001470821	0.002380563	0.000306557	0.000712396	0.000626275	0.001097145	0.0008722	0.002146804
Anaerotrunc	0.00010112	9.49E-05	0.000357084	0.001250751	0.000101771	0.000160583	0.000230265	7.65E-05	0.000198778
Angelakisell	0	0	0	0	0	0.000144525	0.00010836	0	0
Anoxybacillu	0	0	0.000163664	0	0	0	0	0	0
Anseongella	0	0	0.00010415	0	0	0	0	0	0
Arenibacter	0	0	0	0	0	8.03E-05	0	0	0
Atopobium	0	0.000205599	0	6.13E-05	0	0.000417516	0.00010836	0	0.000185526
Atopostipes	0	0	0	0.000196196	0	0	0	0	0
Aureimarina	0	0	0	0	0	0	0	0	0
Azospirillum	0	0.000332121	4.46E-05	0	0.000101771	0	0	0	0.000265038
Bacillus	0	0.00026886	0.000461234	0.027590097	5.82E-05	0	0	0	9.28E-05
Bacteroides	0.464797634	0.240360588	0.29423755	0.395188287	0.449754296	0.427825864	0.127458417	0.395550251	0.446575052
Balneola	0	0.000759133	0.000952225	0	0	0.000963499	0	0	0.000609586
Barnesiella	0.001744318	0.006484264	0.004954546	0.018123628	0.00553924	0.00200729	0.000880425	0.00026013	0.003829793
Bifidobacter	0.00051824	0.001834572	0.014774367	0.00026977	0.000843244	0.000738683	0.00016254	0.000321337	0.001881767
Bilophila	0.002502718	0.002340661	0.001309309	0.003433434	0.001381175	0.001766416	0.00067725	0.002218754	0.001152913
Blautia	0.010971509	0.00857188	0.030560474	0.006486738	0.007981739	0.005957638	0.013883621	0.012455625	0.022514941
Bosea	6.32E-05	0	0.000371963	0	0	3.21E-05	0	0	0
Brevibacillus	0	0	0	0.001397898	0	0	0	0	0
Brevundimo	0	0	0.000119028	0	0	0	0	0	0
Butyricicocc	0.002325758	0.001739681	0.002097871	0.000821572	0.00220988	0.001991232	0.001977569	0.003029747	0.002517857
Butyricimon	0.00030336	0.00686383	0.003555966	0.010422926	0.001686488	0.003420423	0.000528255	0.000137716	0.000927631
Butyrivibrio	0.00034128	0.000189783	0.00029757	0.000159409	0.000159926	0	0.00024381	0	0
C1-B045	0	9.49E-05	0	0	0	0.000353283	0	0	0
CAG-352	0.00026544	0.000474458	0.000952225	0.000122623	0.000407083	0.000208758	0.00016254	0	0.000132519
CAG-56	0.000846879	0.000759133	0.00080344	0.000147147	0.000421622	0.000353283	0.000880425	0.002341168	0.001682989
CAG-873	0	0	0	0	0	0	0	0	0
Campylobac	0	0	0	0	0	0	0	0	0
Candidatus	0	0	0.000282692	0	0	0.000545983	0	0	0
Candidatus	0	9.49E-05	0.000163664	0	0	9.63E-05	0	0	5.30E-05
Candidatus	6.32E-05	4.74E-05	0	0	0	0	0	7.65E-05	0
Candidatus	0	6.33E-05	0	0	0	0	0	0	7.95E-05
Candidatus	0	0.00030049	0	0	0	0	0	0	0.000331297
Candidatus	5.06E-05	0.000253044	0.000267813	0.000159409	4.36E-05	8.03E-05	9.48E-05	0	0.000106015
Candidatus	0	0	0	0	0	0	0	0	0
Caproicipro	0	0	0	0	0	0	0	4.59E-05	0

Catabacter	0	0	0	0	0	0	0	0	0
Catenibacte	0	0	0	0	0	0.000224817	5.42E-05	0	0
Cellulosilytic	3.79E-05	0	0	0	0.00021808	0.001043791	2.71E-05	0.000198923	0.000238534
Cellvibrio	0	0	0.000193421	0	0	0	0	0	0
Cephalotico	0	0	0.000193421	0	0	0	0	0	0
Christensen	0	0	0.000133907	0	0	0	0	0	0
Christensen	0.011603509	0.013854183	0.006308491	0.001643144	0.006164403	0.018675831	0.004307309	0.043349859	0.005671804
Chryseoline	0.00010112	7.91E-05	0.000223178	0	0.000101771	0	0	4.59E-05	0.000132519
Chthoniobac	0	0.000158153	0	0	0	0	0	0	0.000119267
Clavibacter	0	0	0	0	0	0	0	0	0
Cloacibacillu	0	0	0.000535627	0	0	0	0	0	0
Clostridiales	0	0	0	0	0	0	0	0	0
Clostridioide	0	0	0	0	0	0	0	0	0
Clostridium	0.005624795	0.002340661	0.013792385	0.002268519	0.004579687	0.006856904	0.003873869	0.007972212	0.007262029
Collinsella	0.001314559	0.001012178	0.010087634	0.000747998	0.000799628	0.0007708	0.00075852	0.000749786	0.000662594
Colwellia	0	0	0	0	0	0.000112408	0	0	0
Comamona	6.32E-05	0.004048711	0.000267813	9.81E-05	7.27E-05	0.000240875	0.00013545	0	0.000437312
Coprobacillu	0	0.000189783	8.93E-05	0	8.72E-05	0.000128467	0	0	0.000159023
Coprobacte	0.001630558	0.000237229	8.93E-05	0.000134885	0.000508854	0.000240875	6.77E-05	0.004054964	0.000543327
Coprococcu	0.000581439	0.000711687	0.000877832	0.000588589	0.000436161	0.000353283	0.00024381	0.000306035	0.000357801
Coprococcu	0.001238719	0.001375929	0.002112749	0.000588589	0.002268035	0.001188316	0.002207834	0.000336639	0.000622838
Coprococcu	0.001908638	0.000759133	0.002365684	0.000478228	0.001250327	0.000835033	0.002587094	0.00096401	0.00133844
Coriobacter	0	0	0	0	0	0	0	0	0
Coxiella	3.79E-05	0	0.000208299	0	0	0	0	0	0
Crocinitomix	0.0001264	0.000221414	0	0	0.000145387	0	0	9.18E-05	0.000145771
Cyclobacter	0.00017696	7.91E-05	0	0	0	0.000353283	0	0.000122414	7.95E-05
Defluviitalea	0	0.000253044	5.95E-05	9.81E-05	7.27E-05	0	5.42E-05	0	0
Deinococcu	0	0	0.000148785	0	0	0	0	0	0
Delftia	0	0	0.000208299	0	0	0	0	0	0
Desulfovibri	0.000669919	0.002293215	0.002514469	0.004966217	0.009435608	0.007499237	0.007246573	0.000244828	0.000742105
Dialister	0.001542079	0.002293215	0.001919329	0.001103604	0.001119479	0.004656914	0.045511188	0.001438365	0.00169624
Dielma	0.00021488	0.000189783	0.000223178	0.000502753	0.000130848	0.000610216	0.00016254	0.00026013	0.000304793
Dolosicoccu	0	0	0	0	0	0	0	0	0
Donghicola	0	0.000158153	0.000446356	0	0	0.007162012	0	0	0.000119267
Dorea	0.002072958	0.001755496	0.004820639	0.001140391	0.001628333	0.002376632	0.002045294	0.001882115	0.004015319
DTU089	0	0	0	0	0	0	0	0	0
Dubosiella	7.58E-05	7.91E-05	0.002276413	0	8.72E-05	6.42E-05	0.00016254	0	0
Dysgonomo	0	0	0	0	0	0	0	0	0
Eggerthella	0.00013904	0.000221414	0.001577123	0.000551802	0.000145387	0.000256933	0.00024381	0	0.000106015
Eisenbergie	0.00020224	0.000332121	0.000223178	0.00167993	0.00055247	0.001364958	0	0	0.000278289
Enhydrobac	0	0	0.000148785	0	0	0	0	0	0
Enterococcu	0.0001896	0.000205599	0.001934207	0.000318819	0.001759181	0.00048175	0.000257355	0	0
Enterorhabd	0	0	0	0	0	0	0	0	0
Epulopisciur	5.06E-05	6.33E-05	0.000119028	9.81E-05	0.000305312	0.000112408	2.71E-05	0	3.98E-05
Erysipelatoc	0.000695199	0.001012178	0.013956049	0.009736239	0.001381175	0.00200729	0.00113778	0.00113233	0.002517857
Erysipelotric	0.002287838	0.002182508	0.004493312	0.000478228	0.001250327	0.000754741	0.001476405	0.00235647	0.004479135
Erysipelotric	0	0	0	0	0	0	0	0	0
Escherichia-	0.003096797	0.134002847	0.024103197	0.012274528	0.007763659	0.008270037	0.015170396	0.002081038	0.040908549
Eubacteriun	0	3.16E-05	0.000148785	0.000134885	0	4.82E-05	0	0	0
Ezakiella	0	0	0	2.45E-05	0	0	0	0	0
Fabibacter	0	0	0	0	0.000189003	0	0	0	0
Faecalibacte	0.029982051	0.021904159	0.045766318	0.015033537	0.033060975	0.007964929	0.027103538	0.024192068	0.017651502
Faecalibacu	0	4.74E-05	0.00069929	0	0	0	0	0	0
Faecalicocc	0	4.74E-05	0	0	0	0	0	0	0
Faecalitalea	0.00022752	0.000316306	0.005534808	0.003470221	0.000378006	0.000369341	0.00037926	0.000168319	0.000106015
Family XIII A	0.00010112	0.000363751	0.000922468	0.000331081	0.000116309	0.00028905	0.00021672	0.000535561	0
Family XIII U	0.00010112	9.49E-05	0.000327327	0	0.000203542	8.03E-05	0.0002709	0.000122414	0.000132519
Flavonifract	0.000543519	0.000363751	0.001398581	0.001250751	0.000247158	0.000915324	0.00035217	0.000459053	0.000344549
Formosa	0.000695199	0	0	0	0.000523393	0	0	0.000826295	7.95E-05
Fournierella	0	0	0.000163664	0	0	0	0	0	0
Fusicatenib	0.003716156	0.002862565	0.007989764	0.000637638	0.004172603	0.001750357	0.005553449	0.002907333	0.00430686
Fusobacteri	0.000947999	0.00428594	0.013658479	0.122585867	0.002355267	0.009891927	0.00132741	0.000443751	0.015014378
GCA-90006	0	0	0	0	0	0	0	0.000719182	0
GCA-90006	0	0	0	0	0.00021808	0	0.000176085	0	0
Gemella	0.00035392	0.000110707	0.00050587	0.000208459	0.000276235	8.03E-05	0.00029799	0.000168319	0.000132519
Gimesia	0	0.001676419	0.001472973	0	0	0.000224817	0	0	0.001033647
Gluconobac	0	0	0.00029757	0	0	0	0	0	0
Gordonibac	0	0	8.93E-05	0	0	0	0	0	0
Gottschalkia	0	0	0	0	0	0	0	0	0
Granulicatel	0.00011376	0	0	0	0.000305312	0.000176642	0.00021672	0.000168319	0.000119267
Haemophilu	0.000669919	0.000759133	0.001487852	0.000453704	0.000683318	0.004030639	0.000446985	0.000673277	0.001842011
Harryflintia	0	0	0	0	0	0	0	0	0
hgcI clade	0	0.000854025	0	0	0	0	0	0	0.000583083
Hoeflea	0	0	0.000446356	0	0	0	0	0	0
Holdemanel	0.0003792	0.000506089	0.000922468	0.000576326	0.022549504	0.003821881	0.000636615	0.001285347	0.001351692
Holdemania	0.00016432	0	0.000178542	0.000122623	0.000101771	0.000208758	0.000176085	0.000198923	9.28E-05
Howardella	6.32E-05	0	5.95E-05	4.90E-05	0.000392545	0	4.06E-05	0	0
Hungatella	0.00027808	0.000205599	0.000595141	0.000870621	0.000378006	0.001348899	0.000230265	0	0.000371053
Hydrogenoa	0.00011376	0.000442828	8.93E-05	0.000183934	4.36E-05	0	0	0.000336639	5.30E-05
Ileibacteriun	0.00021488	0.000126522	0.005653836	0.000147147	0.000159926	0.000160583	0.000121905	0	0
Intestinibact	0.001858078	0.000632611	0.001934207	0.000441442	0.002122648	0.001429191	0.00132741	0.001193537	0.000371053

Intestinimor	0.0001896	0.000205599	0.000476113	0.000735736	8.72E-05	0.000963499	0.000176085	0	0
Jeotgalibaca	0	0	0	0	0	0	0	0	0
Kordiimonas	0	0.000648426	0.000312449	0	0	0.000706566	0	0	0.000397556
Lachnoclost	0.01007407	0.006136328	0.014789246	0.017608613	0.004783228	0.006840846	0.008086363	0.007528461	0.008189661
Lachnoclost	0	0	0.001190281	0	0	0.000112408	0	0	0
Lachnoclost	0	0	0	9.81E-05	0	0	0	0	0
Lachnospira	0.05216523	0.029416416	0.046138281	0.016321075	0.013608211	0.016010149	0.164978057	0.027420737	0.057287871
Lachnospira	8.85E-05	0	0.000357084	0	0.000145387	0.000401458	0.000176085	0.000107112	0.00021203
Lachnospira	0.001301919	0.000727503	0.003154246	0.000245245	0.000814166	0.000835033	0.001368045	0.000795691	0.00063609
Lachnospira	0.008039032	0.005187411	0.00471649	0.007418671	0.005859091	0.007900695	0.008953243	0.005600441	0.004293609
Lachnospira	0.002995677	0.003004903	0.006769725	0.001545045	0.001860952	0.001702182	0.018421195	0.00122414	0.000768609
Lachnospira	0	0.000316306	0	0.000196196	0.001352098	0	0.001557675	0.000336639	0.000278289
Lachnospira	0.001655838	0.001455006	0.008168306	0.000588589	0.002238958	0.00028905	0.001977569	0.002463582	0.002279323
Lachnospira	0	0	0.000252935	0	0	0	0.00029799	0.000367242	0
Lachnospira	0.000872159	0.000632611	0.000773683	0.000907408	0.000567009	0.000594158	0.001882755	0.002050435	0.001404699
Lacticigeniu	0	0	0	0	0	0	0	0	0
Lactobacillu	0.00041712	0.000774949	0.010370326	0.003715466	0.000319851	0.001156199	0.000446985	0	0.000265038
Lactococcus	0.00027808	0.000442828	0.000654655	0.00038013	0.011267483	0.000626275	0.00062307	0	0
Lentibacter	0	0	0	0	0	0	0	0	0
Leuconostoc	2.53E-05	0	0	0	0	0.000818975	6.77E-05	6.12E-05	0.000106015
Leucothrix	0	0	0	0	0	0	0	0	0
Litoricola	0	0.000126522	0.000119028	0	0	0.000802916	0	0	0.000159023
Mailhella	0	0	0.000208299	0	0	0.000562041	4.06E-05	0	0
Mannheimia	0	0	0.000848075	0	0	0	0	0	0
Maribacter	7.58E-05	0	0	0	5.82E-05	0	0	0.000122414	0
Marinicella	0.00016432	4.74E-05	0	0	0	0	0	0.000168319	0
Marinobacte	0	0	0	0	0	0.000224817	0	0	0
Marivita	0	0.000553535	0.002454955	0	0	0.00287444	0	0	0.000371053
Marvinbryar	0	0	0.000862954	0.000306557	0	0	0	0	0
Massilia	2.53E-05	0	0.00040172	0	0	0	0	0	0
Megamonas	0.00051824	0.001186146	0.002023478	0.00038013	0.000392545	0.000562041	0.000555345	0	0.000357801
Megasphae	0.00030336	0.007607149	0.005103331	0.00091967	0.000421622	0.000449633	0.007544563	0.001545477	0.030161275
Merdibacter	0	0	0	0	0	0	0	0	0
Methanobre	0	0.000711687	0	0	0	0.00028905	0	0	0
Methylobact	0	0	0.000193421	0	4.36E-05	0	0	0	0
Methylotene	0	0	0.000163664	0	0	0	0	0	0
Mf105b01	0	0	4.46E-05	0	0	0	0	0	3.98E-05
Micrococcus	0	0	0.000371963	0	0	0	0	0	0
Mitsuokella	0.00013904	0	0.00029757	0.000159409	5.82E-05	0.000160583	0	0	0
Mogibacteri	0	0.000110707	0	0	0	0	0	0	9.28E-05
Morganella	0	0	0	0	0	0	0.00021672	0	0
Moryella	0.00010112	0.000110707	0.000550505	0.000134885	0.000203542	0	0.000555345	9.18E-05	0
Murimonas	0	0	0	0	0	0	0	0	0
Mycobacter	0	0	0.000178542	0	0	0	0	0	0
Negativibac	0	0.000158153	0	9.81E-05	0.000508854	9.63E-05	0	0	0
Nitrosomona	8.85E-05	0.001644789	0.000416598	0	0	0.001830649	0	4.59E-05	0.000516823
Nosocomiic	0	0	0.000133907	0	0	0	0	0	0
NS3a marin	0	0	0	0	0.003372975	0.002713857	0	0	0
Oblitimonas	0	0	0	0	0	0	0	0	0
Oceanicocc	0	0	0	0	0	6.42E-05	0	0	0
Oceanobact	0	0	0	0	0	0	0	0	0
Oceanospiri	0	0	0	0	0	0.003629181	0	0	0
Odoribacter	0.002818717	0.002530444	0.001487852	0.007541293	0.003402053	0.002232107	0.002004659	0.008752601	0.001629981
Olsenella	0	0	0.000342206	0	0	0	0	0	0
OM27 clade	0	0	0.000371963	0	0	0.000144525	0	0	0
Oribacteriur	0	0	0	0	0	0	0	0	0
Oscillibacter	0.00032864	0.00083821	0.000476113	0.000870621	0.000174464	0.000417516	0.00040635	0.000229526	0.000371053
Oscillospira	0	0	0	0	0	0.000160583	0	0	0
Ostreobium	0	0	0	0	0	0	0	0	0
Oxalobacter	0.00010112	0.000427012	0.000327327	0	0.000494315	0.000433575	0.00035217	0.000214225	7.95E-05
Paenalcalig	0	0.000110707	0	0.000564064	0	3.21E-05	0	0	0
Paeniclostri	0	0	0	0	0.00033439	0	0	0	0
Parabacterc	0.024963976	0.028926143	0.016425883	0.033243001	0.015556396	0.031554606	0.016348811	0.024146162	0.01615404
Paraclostrid	0	0	0	0	0	0	0	0	0
Paracoccus	0	0	0.000416598	0	0	0	0	0	0
Parahaliea	0	0	0	0	0	0	0	0	0
Paralcaliger	0	0	0.000148785	0	0	0	0	0	0
Paraprevote	0.005902874	0.006104697	0.00190445	0.000564064	0.002137187	0.002183932	0.00094815	0.005263802	0.013278382
Parasuttere	0.024774376	0.006737308	0.005534808	0.001778028	0.001337559	0.003372248	0.001259685	0.011124373	0.012297743
Patulibacter	0	0	0	0	0	0	0	0	0
Pediococcu	0	0	0.000252935	0.001961962	0.000872321	0	0.000121905	0	0
Pedobacter	0.0001896	0	0	0	0	0	0	0	0
Peptoclostri	0	0	0	0	0	0	0	0	0
Peptococcu	7.58E-05	0.000110707	5.95E-05	0.000220721	5.82E-05	0.002071524	0.00032508	0	5.30E-05
Phascolarct	0.007280633	0.008144868	0.007632679	0.004659661	0.00299497	0.002762032	0.007070488	0.004835353	0.02617246
Phocea	0	0	0	6.13E-05	0.000116309	0	0.00032508	0	7.95E-05
Planctomicr	0	0.000600981	0.000520748	0	0	0.00028905	0	0	0.000397556
Polynucleob	0	0.000237229	0	0	0	0	0	0	0.000106015
Porticoccus	0	0.00030049	0.000357084	0	0	0.0003854	0	0	0.000159023
Prevotella	0	0	5.95E-05	0	0	0	0	0	0
Prevotella 2	0.001630558	0.003779851	0.00090759	0.001912913	0.001061324	0.003934289	0.028349678	0	0.00049032

Prevotella 6	0	0	0	0	0	0	0	0	0
Prevotella 7	0	3.16E-05	0.000476113	0	0	0	0	0	0
Prevotella 9	0.023662057	0.009599873	0.005891893	0.037755515	0.005248466	0.047869863	0.124004443	0.016112743	0.012271239
Prevotellace	0	0	0.000193421	0	0	0	0	0	0
Prevotellace	0.00013904	0.000142337	0.000357084	0.000159409	7.27E-05	0.001011674	0.000148995	0	0
Prevotellace	0	0	2.98E-05	0	0	0	0	0	0
Prevotellace	0	0	0	0	0	0	0	0	0
Proteocatell	0	0	0	0	0	0	0	0	0
Proteus	0	0	0	0	5.82E-05	0	0.00178794	0	0
Providencia	0	0	0.000148785	0	0	0	0.000121905	0	0
Pseudoalter	0	0	0	0	0	0.004030639	0	0	0
Pseudochro	0	0	0.000193421	0	0	0	0	0	0
Pseudoflavo	0	0	0	0	0	0.000272992	0	0	0
Pseudohong	0	0	0	0	0	0.000465691	0	0	0
Pseudomon	0.00013904	0.000553535	0.005638958	0	0	0.000642333	0	0.000214225	0.00042406
Pseudophae	0	0	0	0	0	0.001252549	0	0	0
Pseudovibri	0	0	0	0	0	0	0	0	0
Pyramidoba	7.58E-05	0.000759133	0.000223178	0	0	3.21E-05	0.00040635	9.18E-05	0.000304793
Raoultibacte	0	0	0	0	0	0	0	0	0
Reichenbac	0.00016432	0.000126522	0.000148785	0	0	0	0	0.000168319	9.28E-05
Rheinheime	0	7.91E-05	0.000238056	0	0	0.000240875	0	0	9.28E-05
Rhodococcu	0	0	0	0	0	0	0	0	0
Rhodoferax	0	0.000173968	0	0	0	0	0	0	0.000119267
Rikenellace	3.79E-05	7.91E-05	0.000238056	0	0	3.21E-05	0	0	0
Romboutsia	0.003665596	0.001170331	0.002127628	0.001177177	0.001308482	0.001557657	0.002316194	0.002631901	0.002040789
Roseburia	0.016141265	0.021904159	0.016604425	0.011134137	0.031476258	0.009121128	0.032724711	0.010512303	0.012589285
Roseibacillu	0	0.000632611	5.95E-05	0	0	0.000337225	0	0	0.000477068
Rothia	0	0	0	0	0.000130848	0	0.00013545	0	0
Ruegeria	0	0	0.000952225	0	0	0.002151815	0	0	0
Ruminiclost	0	0	0	0	0	0	0	0	0
Ruminiclost	0.00034128	0	0	0	0	0	0	0.001805607	0
Ruminiclost	0.000631999	0.000521904	0.00050587	0.000527277	0.001192172	0.000562041	0.00051471	0.001117028	0.000742105
Ruminiclost	0.001011199	0.001360114	0.001651515	0.000527277	0.006397022	0.001043791	0.009183508	0.001101726	0.000728853
Ruminiclost	0.001023839	0.000158153	0.000357084	0.000613113	0.000581547	0.000545983	0.0008127	0.000581467	0.000318045
Ruminococ	0.007040473	0.008034161	0.01083156	0.003936187	0.012227036	0.006455446	0.029135287	0.006671563	0.001775752
Ruminococ	0.009581111	0.066661395	0.011560608	0.006781033	0.011674566	0.029306441	0.007178848	0.022998531	0.011953194
Ruminococ	0.002174078	0.002388107	0.00391305	0.004598349	0.001831875	0.002087582	0.0016254	0.001254744	0.000463816
Ruminococ	0.000316	0.00086984	0.001472973	0.000551802	0.000290774	0.000883208	0.000636615	0.000428449	0.000278289
Ruminococ	0.014346386	0.023485687	0.009998363	0.004316317	0.004768689	0.003950348	0.003426884	0.077335047	0.008149905
Ruminococ	0	0	0	0	0	0	0	0	0
Ruminococ	0.000846879	0.001043808	0.000223178	3.68E-05	0.000697857	0.001348899	4.06E-05	0.001667891	0.000291541
Ruminococ	0.003071517	0.002372292	0.002960825	0.002121372	0.001250327	0.001782474	0.002600639	0.003703024	0.005459774
Ruminococ	0.002161438	0.01423375	0.002231778	0.001005506	0.004521532	0.001702182	0.003453974	0.002264659	0.003763533
Ruminococ	0.019996461	0.005313933	0.011352308	0.003997498	0.006964031	0.006648146	0.014073251	0.006457339	0.001523966
Ruminococ	0.013550067	0.005266487	0.007855857	0.004414416	0.064188305	0.011947393	0.008844883	0.003764231	0.001219173
Sanguibacte	0	0	0	0	0	0	0	0	0
Sarcina	0.001554719	0.000237229	0.000357084	0.000147147	0.000305312	0.000224817	0.00018963	0.000397846	0
Sediminibac	0	0.000237229	0	0	0	0	0	0	0
Selenomona	0	0.000427012	7.44E-05	0	0	0	0	0	0.000119267
Sellimonas	0	0	0	0	0	0	0.00021672	0	0
Senegalima	0	7.91E-05	8.93E-05	0	0	0.000497808	0	0	0
Serratia	0	0	0.001309309	0	0	0	0	0	0
Shuttleworth	0	0	0	0	0	0	0	0	0
Slackia	0	0	0	0	0	0	0	0	0
Sneathiella	0	0.000237229	8.93E-05	0	0	0	0	0	0.000238534
Solobacteriu	5.06E-05	0.000110707	7.44E-05	8.58E-05	0	0	5.42E-05	0	0.000145771
Sphingobac	3.79E-05	0	0.000163664	0	0	0	0	0	0
Sphingomor	0	0	0.000877832	0	4.36E-05	0	0	0	0
Staphylococ	0	0	0.00029757	0	0.000523393	0	0	0	0
Stenotropho	0	0	0.000327327	0	0	4.82E-05	0	0	0
Streptococc	0.002945117	0.004665507	0.005996042	0.005419921	0.044982699	0.007595587	0.02095411	0.001790305	0.002915413
Subdoligran	0.012260788	0.007354104	0.016291976	0.002011012	0.006062633	0.004833555	0.010998537	0.00550863	0.004730921
Succinatimo	0	0	0	0	0	0	0	0	0
Succinivibrio	0	0	0.000133907	0	0	0	0	0	0
Sulfitobacte	0	0.000822394	0.000967104	0	0	0.001220433	0	0	0.000543327
Sutterella	0.002439518	0.003510992	0.004523069	0.006744246	0.003009508	0.012316734	0.010673457	0.003366385	0.002769643
Synergistes	0	3.16E-05	0	0	0	0.000513866	0	0	0
Taeseokella	0.00027808	0.000205599	0.000386841	0	0.000697857	0	0	0.000367242	0.000198778
Tepidimicro	0	0	0	0	0	0	0	0	0
Terrisporob	0.000745759	0	0.000252935	0	0	0.000401458	0.00021672	0.000183621	0.000185526
Thalassotale	0	0	0	0	0	0.000562041	0	0	0
Thiopseudo	0	0	8.93E-05	0.000159409	0	0	0	0	0
Tissierella	0	0	0.000119028	2.45E-05	0	0	0	0	0
Turicibacter	0.0001896	0.000759133	0.00040172	0.000355606	0	0.000321166	0.000744975	0.00035194	0.000993891
Tyzzerella	0.0005056	0	0.000684412	0	0	0.000272992	0	0.000198923	0
Tyzzerella 3	0.001301919	0.001201961	0.002484712	0.000698949	0.000857783	0.001091966	0.00105651	0.000244828	0.000265038
Tyzzerella 4	0.000796319	0.000901471	0.002440077	0.005272774	0.001715565	0.005010197	0.001286775	0.002249357	0.000477068
UBA1819	0.00039184	0.000600981	0.001770544	0.001385636	0.00055247	0.00067445	0.0002709	0.00052026	0.000503571
uncultured	0.017784463	0.028973588	0.023969291	0.006057559	0.014407839	0.020442246	0.009332503	0.014184723	0.014842104
uncultured b	0.000758399	0.002261585	0.007900492	0.000134885	0.000450699	0.001429191	4.06E-05	0.00209634	0.000781861
uncultured b	0.00024016	0.003052349	0.00040172	6.13E-05	0.000203542	0.000144525	0.00010836	0.00026013	0.001007143

uncultured E	0	0	0.000357084	0	0	0	0	0	0
uncultured E	3.79E-05	7.91E-05	5.95E-05	0	8.72E-05	8.03E-05	0	0	9.28E-05
uncultured (	0	0.00026886	0	0	0	0.000160583	0	0	0.000119267
uncultured (	0	0	0	0	0	0	0	0	0
uncultured g	0	0.000110707	0	0	0	0	0	0	9.28E-05
uncultured r	0.00017696	0.000221414	7.44E-05	0	0	0.000112408	0	9.18E-05	0.000119267
uncultured d	0.001200799	0.00428594	0.000654655	0.000232983	0.000363467	0.001172258	0.000203175	0.004223283	0.001285432
uncultured F	0	0	0.00010415	0	0	0.001300724	0	0	0
uncultured p	0	0	0	0	0	0.000128467	0	0	0.000106015
uncultured r	0	0.000110707	0.001145646	0	0	0	0	0	5.30E-05
uncultured S	5.06E-05	0	0	0	0	0	0	0.000137716	0
uncultured T	6.32E-05	0.000316306	0	0	0	0	0	0	0
uncultured V	2.53E-05	0.001233592	0.000833197	0	0	0.000144525	0	0	0.000834868
unidentified	0	0	0	0	0	0	0	0	0
Veillonella	0.00045504	0.001328483	0.000967104	0.000392392	0.000450699	0.000979558	0.008560438	0.000413147	0.002411842
Vibrio	0	0	0.000133907	0	0	0.000321166	0	0	0
Victivallis	0.00036656	0.002261585	0.000416598	0.000232983	0.000305312	0.023316686	0.00051471	0.000198923	0.000265038
W5053	0	0	0	0	0	0	0	0	0
Weissella	0.0001896	0.000316306	0.000342206	0.000122623	0.001512023	0.00028905	0.00051471	6.12E-05	5.30E-05
Wenyingzhu	0	0	0	0	0.00021808	0.000144525	0	0	0
Woeseia	0	0.000221414	0	0	0	0	0	0	0.000185526

Genus	W72	W73	W75	W78	W7	W13	W15	W18	W20
[Caedibacte	0	0	0.00015313	0	0	0	0	0	0
[Clostridium	0.000158286	3.74E-05	0	7.99E-05	0.000188901	0.000130687	7.83E-05	5.94E-05	5.23E-05
[Eubacteriu	0	0	0	0	0	0.000145207	0	0	0
[Eubacteriu	0.013718145	0.004530478	0.010259682	0.069191435	0.006152767	0.007086123	0.005418179	0.19457612	0.012962576
[Eubacteriu	0.0012531	0.001123259	0.002628725	0.013342921	0.004574096	0.003731831	0.003263963	0.003848726	0.004312147
[Eubacteriu	0.003627394	0.000823723	0.001008103	0.004434324	0.001295319	0.002265236	0.000678904	0.003551527	0.001215241
[Eubacteriu	0	0.000312016	0	0	0.000485745	0.00018877	0.000208894	0.000237759	0.000209074
[Eubacteriu	0.000606764	0.000299536	0.00080393	0.010772877	0.005208263	0.001640844	0.001475311	0.004606583	0.036627117
[Eubacteriu	0.001582863	0.002109231	0.001263319	0.003062746	0.002010444	0.000450143	0.000391676	0.005691359	0.001254443
[Eubacteriu	0.000237429	0.000112326	0.000727366	0.00516672	0.000971489	0.000232332	0.000261117	0.001500854	0.004338281
[Polaribacte	0	0	0.001365405	0	0	0	0	0	0
[Ruminococ	0	0	0	0.000439438	0	0	0.000143614	0.000534958	0.000522685
[Ruminococ	0.012161663	0.013928411	0.006980157	0.006258656	0.012791278	0.000377539	0.000718072	0.000653838	0.001280577
[Ruminococ	0.014285337	0.004343268	0.003547502	0.01255726	0.027997787	0.003644706	0.006018748	0.007221933	0.009617395
Abiotrophia	0	0	0	5.33E-05	0	0	0	0	0
Acetanaerol	0	0	0	0	6.75E-05	4.36E-05	0	0	7.84E-05
Acetitomacu	0	0	0	0	0	0	0	0	0.000156805
Acetobacter	0	0	0	0	0	0	0	0	0
Acidaminoc	0	0	0	0.000292958	0.000269858	0.000275894	0.004895945	0	0.000365879
Acinetobact	0	0	0.00048491	0	0	0	0	0	0
Actinobacillu	0	0	0.000178651	0	0	0	0	0	0
Actinomyce	0	0.000174729	0.000191412	0.000159795	0.000377801	0.00058083	0.000169726	0.000297199	0.000104537
Adlercreutzi	0	0	0	0	0.000107943	0.000101645	7.83E-05	0.000178319	7.84E-05
Aeromonas	0	0	0.000127608	0	0	0	0	0.000252619	0
Aerosphaer	0	0	0	0	0	0	0	0	0
Aestuariicel	0	0	0	0	0	0	0	0	0
Agathobact	0.016817918	0.004318307	0.004427997	0.036806221	0.003562128	0.006708583	0.010209677	0.011457018	0.017836609
Aggregatiba	0	0	0	0	0	0	0	0	0
Akkermansi	0.00026381	0.001447756	8.93E-05	2.66E-05	0.001038954	0.040890412	0.100908687	0.001248235	0.002325946
Alcaligenes	0	0	0	0	0	0	0	0	0
Alcanivorax	0	0	0	0	0	0	0	0	0
Alistipes	0.053025906	0.022327892	0.049818159	0.008589006	0.023693549	0.054888408	0.053777058	0.024474329	0.038521848
Allisonella	0	0.000124807	0	0.000292958	0.000148422	0	0.000757239	0.000475518	0.000130671
Allobaculum	0	0	0	0	0	0	0	0	0
Alloprevotel	0.000277001	0.000336978	0.000382824	0.000266326	0.000431773	0	0	0	0
Allorhizobiu	0	0	0	0	0	0	0	0	0
Alloscardov	0	0	0	0	0	0	0	0	0
Amphritea	0	0	0	0	0	0	0	0	0
Anaerofilum	0	0	0	0	0	0	0	0	0
Anaeroglobu	0	0	0	0	0	0	0	0	0
Anaerospor	0	0.000336978	0.000829452	0.000292958	0	0.000101645	0.000182782	0.000490378	0.000222141
Anaerostipe	0.000646336	0.000436823	0.000969821	0.002064025	0.00032383	0.000755079	0.000443899	0.001530574	0.000483483
Anaerotrunc	0.000791431	0.000137287	0	0.000519335	0.000863546	0.000667954	0.000352508	0.000163459	0.000535752
Angelakisell	0	0	0	0	6.75E-05	0.000232332	0	0.000356639	0.000392013
Anoxybacillu	0	0	0	0	0	0	0	0	0
Anseongella	0	0	0	0	0	0	0	0	0
Arenibacter	0	0	5.10E-05	0	0	0	0	0	0
Atopobium	0	8.74E-05	0	0	0	0	0	0.000237759	7.84E-05
Atopostipes	0	0	0	0	0	8.71E-05	5.22E-05	0.00010402	7.84E-05
Aureimarina	0	0	0	0	0	0	0	0	0
Azospirillum	0	3.74E-05	5.10E-05	0	0	0.000363019	0.000274173	0.000564678	0.000209074
Bacillus	0	0.028168838	0	0	0	0	0	0	0
Bacteroides	0.424761252	0.232601967	0.575448223	0.284023117	0.49863047	0.441459625	0.242982479	0.078965748	0.380631926
Balneola	0	0	0	0	0	0	0	0	0
Barnesiella	0.000277001	0.004667765	0.001212276	0.000266326	0.00393993	0.003339771	0.003198684	0.00265993	0.014922643
Bifidobacter	0.000118715	0.000262094	0.004734256	0.000199744	0.000634167	0.001684406	0.000117503	0.000861877	0.001345913
Bilophila	0.000237429	0.001684888	0.004657692	0.000319591	0.006894877	0.003978684	0.004086482	0.001277955	0.001803262
Blautia	0.06998892	0.017984624	0.012161041	0.037045915	0.010146668	0.004370744	0.004569549	0.009332045	0.014909576

Bosea	0	0	0	0	0	0	0	0	0
Brevibacillus	0	0.001123259	0	0	0	0	0	0	0
Brevundimo	0	0	0	0	0	0	0	0	0
Butyricicocc	0.002136865	0.000998452	0.001926881	0.001970811	0.003764522	0.002787983	0.001579758	0.004606583	0.024775246
Butyricimon	0.001741149	0.001485198	0.000446628	9.32E-05	0.000472252	0.000711516	0.005731519	0.001203656	0.000365879
Butyrivibrio	0	0	0	0	0	0	9.14E-05	0	0
C1-B045	0	0	0	0	0	0	0	0	0
CAG-352	0	0	0	0	0	0	0	0	0
CAG-56	0.000844194	0.000524188	0.000829452	0.001238415	0.000229379	0.000566309	0.000117503	0.002526191	0.000810161
CAG-873	0	0	0	0	0	0	0	0	0
Campylobac	0	0	0	0	0	0	0	0	0
Candidatus	0	0	0	0	0	0	0	0	0
Candidatus	0	0	0	0	0	0	0	0	0
Candidatus	0	0	0	0	0	0	0	0	0
Candidatus	0	0	0	0	0	0	0	0	0
Candidatus	0	0	0	0	0	0	0	0	0
Candidatus	7.91E-05	0.000112326	0.000114847	0	0.000121436	0	0	0.000445798	0.00058802
Candidatus	0	0	0	0	0	0	0	0	0
Caproicipro	0	0	0	0	8.10E-05	0	0	0	5.23E-05
Catabacter	0.000118715	0	0	0	8.10E-05	0	0	0	0
Catenibacte	0	0	0	0	0	0	0	0	0
Cellulosilytic	0.000131905	9.98E-05	0.000127608	0.000266326	0	0	0	0	0
Cellvibrio	0	0	0	0	0	0	0	0	0
Cephalotico	0	0	0	0	0	0	0	0	0
Christensen	0	0	0	0	0	0	0	0.00013374	0
Christensen	0.002585343	0.001922021	0.002922223	0.002370299	0.004843955	0.003847997	0.005117894	0.019570548	0.018411562
Chryseoline	0	0	2.55E-05	0	0	0	0	0	0
Chthoniobac	0	0	0	0	0	0	0	0	0
Clavibacter	0	0	0	0	0	0	0	0	0
Cloacibacillu	0	0	0	0	0	0	0	0	0
Clostridiales	0	0	0	0	0	0	0	0	0
Clostridioide	0	0	0	0	0	0	0	0	0
Clostridium	0.004748589	0.00666467	0.00782237	0.00785661	0.002415231	0.00332525	0.000822519	0.01627164	0.00226061
Collinsella	0.011528518	0.0003869	0.003394372	0.000519335	0.001889007	0.00214907	0.002101992	0.001634594	0.000901631
Colwellia	0	0	0	0	0	0	0	0	0
Comamona	0	0	0	0	0	0	0	0.00013374	0.000117604
Coprobacillu	0	0	0	0	0.000769096	8.71E-05	0	8.92E-05	0.000156805
Coprobacte	0.000936527	0.000237132	0.00048491	0.000173112	0.000350816	0.000421102	0.000182782	5.94E-05	9.15E-05
Coprococcu	0	0.000212171	0.000191412	0	0.000121436	0.000101645	0.000130559	0.000638978	0.000509617
Coprococcu	0.00025062	0.000237132	0.000880495	0.000985405	0.001079433	0.000450143	0.00053529	0.001173936	0.006023939
Coprococcu	0.009734607	0.000474265	0.000880495	0.001171833	0.000634167	0.000290415	0.001031412	0.001723754	0.001306711
Coriobacter	0	0	0	0	0	0	0	0	0
Coxiella	0	0	0	0	0	0	0	0	0
Crocinitomi	0	0	0	0	0	0	0	0	0
Cyclobacter	0	0	0	0	0	0	0	0	0
Defluviitalea	0	0	0	9.32E-05	0	4.36E-05	5.22E-05	0.00010402	0
Deinococcu	0	0	0	0	0	0	0	0	0
Delftia	0	0	0	0	0	0	0	0	0
Desulfovibri	0.000422097	0.000923568	0.010081031	0.012064557	0.000877039	0.005430758	0.00865603	0.002065532	0.002861698
Dialister	0.000923337	0.001235585	0.001263319	0.029122723	0.003359734	0.005241988	0.030054573	0.007652872	0.021691407
Dielma	2.64E-05	0.000162249	0.000114847	0	0.000215887	0	0	8.92E-05	0
Dolosicoccu	0	0	0	0	0	0	0	0	0
Donghicola	0	0	0	0	0	0	0	0	0
Dorea	0.002651295	0.001085817	0.002360748	0.001531373	0.001983458	0.001466595	0.000770295	0.006895014	0.003201443
DTU089	0	0	0	0	0	0	0	0.000178319	0.000326678
Dubosiella	0	0	0	0	0	0	0	0	0
Dysgonomo	0	0	0	0	0	0	0	0	0
Eggerthella	5.28E-05	0.00018721	0.000382824	0.000652498	0.000485745	0.000290415	0.00031334	0.000178319	0.000300544
Eisenbergie	0.000184667	0.000424342	0.001901359	0	0.001632642	0.000246853	0.000496122	0.000475518	0.000392013
Enhydrobac	0	0	0	0	0	0	0	0	0
Enterococcu	0	0	0.000446628	0	0	0.000217811	0	0.000148599	0
Enterorhabd	0	0	0	0	0	0	0	0	0
Epulopisciu	0	6.24E-05	0.000114847	0	0.000148422	0	0	0	0
Erysipelatoc	0.004234158	0.008736458	0.004823582	0.002303718	0.000431773	0.000261373	0.000274173	0.000505238	0.000261342
Erysipelotric	0.001398196	0.001634966	0.002322465	0.001970811	0.00213188	0.003455937	0.001475311	0.002273572	0.008219214
Erysipelotric	0	0	0	0	0	0	0	0	0
Escherichia-	0.004445207	0.003881484	0.027397435	0.002982849	0.008433068	0.011602074	0.010823302	0.10008173	0.006821033
Eubacterium	0	0	0	0	0	2.90E-05	0	0	0
Ezakiella	0	0	0	0	0	0	0	0	0
Fabibacter	0	0	0.000293498	0	0	0	0	0	0
Faecalibact	0.011594471	0.013004842	0.040643144	0.030227975	0.016353406	0.012182903	0.008159908	0.032052901	0.023298662
Faecalibacu	0	0	0	0	0	0	0	0	0
Faecalicocc	0	0	0	0	0	0	0	0	0
Faecalitalea	0.001319052	0.001173182	5.10E-05	0.000479386	0.001727092	0	0	0	0.000418148
Family XIII A	0.000145096	0.000112326	0	0	0	0.000159728	0.000365564	0.000817297	0.000444282
Family XIII U	0.00026381	6.24E-05	0	0.00010653	9.45E-05	0	0	0.000653838	0.000444282
Flavonifract	0.001173957	0.00076132	0.000459389	0.001065303	0.003076383	0.00058083	0.000731128	0.000326919	0.000888564
Formosa	0	0	0.00048491	0	0	0	0	0	0
Fournierella	0	0	0	0	0.000107943	0.00020329	0	0.000237759	0
Fusicatenib	0.006423785	0.001759772	0.005308492	0.021385959	0.001295319	0.001045493	0.00069196	0.004948362	0.003697993
Fusobacteri	0.002400675	0.009460336	0.006188987	0.00046607	0.047414084	0.115599634	0.000639737	0.004205364	0.002208342

GCA-90006	0	0	0	0	0.000269858	0.000159728	0	0.000252619	0.002757161
GCA-90006	0	0	0	6.66E-05	0	0	0	0.00011888	0.000313611
Gemella	0.000197858	0.000112326	0.000102086	0.000133163	0	0	0.000130559	0.00011888	6.53E-05
Gimesia	0	0	3.83E-05	0	0	0	0	0	0
Gluconobac	0	0	0	0	0	0	0	0	0
Gordonibac	0	0	0	0	0	0.000130687	0	0	0.000130671
Gottschalkia	0	0	0	0	0	0	0	0	0
Granulicatel	0.00025062	0.000174729	0.00016589	0	0	0	0.000248061	0	0.000104537
Haemophilu	0.001292671	0.001198143	0.001531296	0.001025354	8.10E-05	0.000116166	0.000731128	0.000817297	0
Harryflintia	0	0	0	0	0	0	0	0	0
hgcI clade	0	0	0	0	0	0	0	0	0
Hoeflea	0	0	0	0	0	0	0	0	0
Holdemanel	0.10675091	0.001672408	0.00590825	0.001877597	0.000553209	0.000667954	0.000731128	0.020001486	0.000757893
Holdemania	0.000408906	0.000174729	0.000127608	0.000519335	0.000877039	7.26E-05	0.000195838	0.000178319	0.000300544
Howardella	0	0	0.000127608	0	0	0	0	0	0
Hungatella	0.000474859	0.00037442	0.001569578	0.000492703	0.002658103	0.000479185	0.001057524	0.000282339	0.000352812
Hydrogenoa	0	6.24E-05	0	0	0	0	0.000143614	0.000208039	0.000130671
Ileibacteriun	0	0	0	0	0	0	0	0	0
Intestinibact	0.000976099	0.000561629	0.001212276	0.00082561	0.000161915	0.00078412	0.000522234	0.004249944	0.000522685
Intestinimor	0.00076505	0.000112326	0	0	0	0.000145207	0.000208894	0	9.15E-05
Jeotgalibaca	0	0	0	0	0	0	0	0	0
Kordiimonas	0	0	0	0	0	0	0	0	0
Lachnoclost	0.022252414	0.037491888	0.01070631	0.009241504	0.009472022	0.007071602	0.008225187	0.0053793	0.009238449
Lachnoclost	0	0	0	0	0	0.000319456	0	0	0
Lachnoclost	0	0	0	0	0	0	0	0	0
Lachnospira	0.015010816	0.08269682	0.022254833	0.092188665	0.020711616	0.017163518	0.00528762	0.013032172	0.038077566
Lachnospira	0	0	0	0.000133163	0.000121436	0	0	0.000951036	0.000392013
Lachnospira	0.000171477	8.74E-05	0.000344542	0.003568765	0.001119911	0.000348498	0.000274173	0.001055056	0.00117604
Lachnospira	0.002901915	0.003195048	0.003215721	0.008296048	0.007448086	0.00410937	0.001958378	0.006419496	0.012060945
Lachnospira	0.000474859	0.00399381	0.000612518	0.005566209	0.001362784	0.000566309	0.000613625	0.001902073	0.00244355
Lachnospira	0.000356144	0.000287055	0.000459389	0.000812294	0.000242872	0.000130687	0	0.000208039	0.002639557
Lachnospira	0.00150372	0.001597524	0.002692529	0.003675296	0.001254841	0.001655365	0.00138392	0.00260049	0.00606314
Lachnospira	0	0	0	0	0	0	0	0	0
Lachnospira	0.008916794	0.000973491	0.000918778	0.007057633	0.000917518	0.001786051	0.001031412	0.003239468	0.00606314
Lacticigeniu	0	0	0	0	0	0	0	0	0
Lactobacillu	0.000131905	0.003007838	0	0.00010653	0.000377801	0.000914807	0.00053529	0.003046289	0.002325946
Lactococcus	0.000118715	0	0.002207618	0	0.000148422	0	0	0	0
Lentibacter	0	0	0	0	0	0	0	0	0
Leuconosto	0.000145096	0.000149768	0.000191412	0.000226377	0	0	9.14E-05	5.94E-05	0
Leucothrix	0	0	0	0	0	0	0	0	0
Litoricola	0	0	0	0	0	0	0	0	0
Mailhella	0	0	0	0	4.05E-05	0	0	0	0
Mannheimia	0	0	0	0	0	0	0	0	0
Maribacter	0	0	6.38E-05	0	0	0	0	0	0
Marinicella	0	0	0	0	0	0	0	0	0
Marinobacte	0	0	0	0	0	0	0	0	0
Marivita	0	0	0	0	0	0	0	0	0
Marvinbryar	0	0	0	0	0	0	0	0.00011888	0.000222141
Massilia	0	0	0	0	0	0	0	0	0
Megamonas	0	0	0	0	0.003521649	0.000435622	0.002728673	0.004532283	0.001816329
Megasphae	0.001213528	0.003968848	0.001378166	0.008309364	0.002158866	0.004152932	0.270804502	0.006508656	0.00352812
Merdibacter	0	0	0	0	0	0	0	0	0
Methanobre	0	0	0	0	0	5.81E-05	0	0	0.000248275
Methylobact	0	0	0	0	0	0	0	0	0
Methylotene	0	0	0	0	0	0	0	0	0
Mf105b01	0	0	0	0	0	0	0	0	0
Micrococcus	0	0	0	0	0	0	0	0	0
Mitsuokella	0	0	0	0	0.000121436	0	0	0	0
Mogibacteri	0	0	0	0	0	0	0	0.001055056	0.000300544
Morganella	0	0	0	0	0	0	0	0	0
Moryella	0	0	0	0	0	0	7.83E-05	0.000638978	0.000457349
Murimonas	0	0	0	0	0	0	0	0	0
Mycobacter	0	0	0	0	0	0	0	0	0
Negativibac	0	0	0.00015313	0	0.007110763	0.000319456	0.000417787	0.00279367	0.00135898
Nitrosomona	0	0	2.55E-05	0	0	0	0	0	0
Nosocomiic	0	0	0	0	0	0	0	0	0
NS3a marin	0	0	0.003611306	0	0	0	0	0	0
Oblitimonas	0	0	0	0	0	0	0	0	0
Oceanicocc	0	0	0	0	0	0	0	0	0
Oceanobact	0	0	0	0	0	0	0	0	0
Oceanospiri	0	0	0	0	0	0	0	0	0
Odoribacter	0.005737878	0.002308921	0.0015951	0.000599233	0.001227855	0.000943848	0.002415333	0.000847017	0.003240644
Olsenella	0	0	0	0	0	0	0	0.000401219	0
OM27 clade	0	0	0	0	0	0	0	0	0
Oribacteriur	0	0	0	0	0	0	0	0	0
Oscillibacter	0.000949718	0.000886127	0.000586997	0.000705763	0.006652004	0.000696996	0.000261117	0.000445798	0.002339013
Oscillospira	0	0	0	0	0	0	0	0	0
Ostreobium	0	0	0	0	0	0	0	0	0
Oxalobacter	0	0	0.000114847	7.99E-05	0.000607181	0.001931259	0.000130559	0.000921317	0.000483483
Paenalcalig	0	0.000162249	0	0	0	5.81E-05	2.61E-05	0	0.000130671
Paeniclostri	0	0	0	0	0	0	0	0	0

Parabacterc	0.011634042	0.015788029	0.028137561	0.046154256	0.047279155	0.027792702	0.038201426	0.009956163	0.041357412
Paraclostrid	0	0	0	0	0	0	0	0	0
Paracoccus	0	0	0	0	0	0	0	0	0
Parahaliea	0	0	0	0	0	0	0	0	0
Paralcaliger	0	0	0	0	0	0	0	0	0
Paraprevote	0.004643064	0.000436823	0.00095706	0.000506019	0.005478121	0.003847997	0.000300285	0.001798053	0.000601087
Parasuttere	0.003719728	0.005791024	0.001173993	0.001238415	0.006840905	0.010382331	0.009818001	0.003640687	0.00497857
Patulibacter	0	0	0	0	0	0	0	0	0
Pediococcu	0	0.00170985	0.000370063	0	0	0	0	0	0
Pedobacter	0	0	0	0	0	0	0	0	0
Peptoclostri	0	0	0	0	0	0	0	0	0
Peptococcu	0.000118715	0	0	5.33E-05	0.000445266	0.000101645	5.22E-05	0.002124972	0.000222141
Phascolarct	0.007874743	0.014377715	0.005053276	0.002889635	0.002981933	0.00274442	0.006214586	0.005542759	0.002639557
Phocea	0	6.24E-05	8.93E-05	0.000918824	0.000202394	7.26E-05	0.000182782	0.000267479	0.000130671
Planctomicr	0	0	0	0	0	0	0	0	0
Polynucleob	0	0	0	0	0	0	0	0	0
Porticoccus	0	0	0	0	0	0	0	0	0
Prevotella	0	0	0	0	0	0	0	0	0
Prevotella 2	0	0	0	0.003528816	0.000890532	0	0.027913414	0.000713277	0
Prevotella 6	0	0	0	0	0	0	0	0	0
Prevotella 7	0	0	0	0	0	0.000101645	0.00015667	0.000638978	6.53E-05
Prevotella 9	0.006489738	0.369327542	0.009685446	0.027391605	0.002523174	0.003281688	0.007415724	0.01887213	0.001319778
Prevotellace	0	0	0	0	0	0.00018877	0.000130559	0.000282339	0.000130671
Prevotellace	0	0	0	0	0.000296844	0	0	0.000951036	0
Prevotellace	0	0	0	0	0	0	0	7.43E-05	0
Prevotellace	0	0	0	0	0	0	0	0	0
Proteocatell	0	0	0	0	0	0	0	0	0
Proteus	0	0	0	0.00010653	0	0.000101645	0	0.001738614	0
Providencia	0	0	0	0	0	0	0	0	0
Pseudoalter	0	0	0	0	0	0	0	0	0
Pseudochro	0	0	0	0	0	0	0	0	0
Pseudoflavo	0	0	0	0	0	0	0	7.43E-05	0
Pseudohong	0	0	0	0	0	0	0	0	0
Pseudomon	0	0	0	0	0	0	0	0.000163459	0.000104537
Pseudophae	0	0	0	0	0	0	0	0	0
Pseudovibri	0	0	0	0	0	0	0	0	0
Pyramidoba	9.23E-05	0	7.66E-05	0.002237136	0.000404787	0.000363019	0.021607437	0.000638978	0.000418148
Raoultibacte	0	0	0	0	0	0	0	4.46E-05	0
Reichenbac	0	0	0	0	0	0	0	0	0
Rheinheime	0	0	0	0	0	0	0	0	0
Rhodococcu	0	0	0	0	0	0	0	0	0
Rhodoferax	0	0	0	0	0	0	0	0	0
Rikenellace	0	0	0	0	0.000269858	0	0	0	0
Romboutsia	0.001741149	0.00094853	0.001863077	0.002103974	0.001524699	0.002047425	0.001240306	0.012200015	0.002966235
Roseburia	0.005315781	0.005054665	0.00974925	0.025527325	0.013034151	0.009598211	0.003603415	0.026302103	0.027009722
Roseibacillu	0	0	0	0	0	0	0	0	0
Rothia	0	8.74E-05	0	0	0	0	0	0	0
Ruegeria	0	0	0	0	0	0	0	0	0
Ruminiclost	0	0	0	0	0	0.000145207	0	0	0
Ruminiclost	0.000118715	6.24E-05	0.000140369	9.32E-05	0	0	0.00015667	0	0
Ruminiclost	0.006186356	0.00094853	0.002424552	0.001358261	0.000809575	0.00039206	0.000900854	0.001084776	0.001881664
Ruminiclost	0.000540811	0.000461784	0.001646143	0.01043997	0.000337323	0.00156824	9.14E-05	0.000549818	0.001698725
Ruminiclost	0.001609244	0.001210624	0.002105532	0.001597955	0.006921863	0.001205222	0.000208894	0.000802437	0.001097637
Ruminococ	0.004563921	0.001223104	0.002615964	0.008309364	0.003400213	0.001640844	0.006018748	0.009837284	0.008637361
Ruminococ	0.007241598	0.002521092	0.004912908	0.00444764	0.005127306	0.009235192	0.005627073	0.08112044	0.019796676
Ruminococ	0.000237429	0.002046827	0.001263319	0.001225099	0.00116039	0.000624392	0.000979189	0.007311093	0.005448986
Ruminococ	0.000461668	0.000324497	0.000280738	0.000173112	0.000823067	0.000624392	0.000261117	0.000787577	0.000352812
Ruminococ	0.003152535	0.003494583	0.004262107	0.008122936	0.002914468	0.001974821	0.00412565	0.025514526	0.01045369
Ruminococ	0	0	0	0	0	0	0	0	0.000156805
Ruminococ	2.64E-05	2.50E-05	0.000293498	0	0.000458759	0.000479185	0.000326396	0.008589048	0.000614154
Ruminococ	0.008204506	0.005553891	0.001505774	0.002356983	0.001848529	0.001510157	0.001501423	0.00527528	0.005200711
Ruminococ	0.000962908	0.000736359	0.002105532	0.001491424	0.003643086	0.005140343	0.004922057	0.03252842	0.014334623
Ruminococ	0.001081623	0.001572563	0.002271422	0.022904016	0.003427199	0.004283619	0.002924511	0.011323278	0.007735731
Ruminococ	0.005685116	0.000998452	0.01373062	0.01245073	0.003184327	0.008436552	0.003159516	0.007519132	0.005435919
Sanguibacte	0	0	0	0	0	0	6.53E-05	0	0
Sarcina	0	0	0	0	0	0	0	0	0
Sediminibac	0	0	0	0	0	0	0	0	0
Selenomona	0	0	0	0	0	0	0	0	0
Sellimonas	0	7.49E-05	0	0.001358261	0	0	0	0	0
Senegalima	0.000184667	0	0	0	0	0.000101645	6.53E-05	0.001902073	0
Serratia	0	0	0	0	0	0	0	0	0
Shuttleworth	0	0	0	0	0	0	0	0	0
Slackia	0	0	0	0	0.001187376	0.00020329	2.61E-05	0	0.000222141
Sneathiella	0	0	0	0	0	0	0	0	0
Solobacteriu	7.91E-05	4.99E-05	0	3.99E-05	0.000134929	0.000159728	0	0	7.84E-05
Sphingobac	0	0	0	0	0	0	0	0	0
Sphingomor	0	0	0	0	0	0	0	0	0
Staphylococ	0	0	0.000178651	0	0	0	0	0	0
Stenotropho	0	0	0	0	0	0	0	0	0
Streptococ	0.010776658	0.004555439	0.01134435	0.005126771	0.046253694	0.002221674	0.004843721	0.004190504	0.003384382
Subdoligran	0.013625811	0.001585043	0.002896701	0.006018962	0.00858149	0.007942846	0.007846568	0.020952522	0.021469266

Succinatimo	0	0	0	0	0	0.000174249	0	0	0
Succinivibrio	0	0	0	0	0	0	0	0	0
Sulfitobacte	0	0	0	0	0	0	0	0	0
Sutterella	0.001437767	0.001697369	0.001709947	0.024728348	0.003184327	0.077308435	0.002976734	0.004457984	0.008754966
Synergistes	0	0	0	0	0	0.000145207	0	0	0
Taeseokella	0	0	0.000446628	0	0	0	0	0	0
Tepidimicro	0	0	0	0	0	8.71E-05	0	0	0
Terrisporoba	0.000171477	0.000112326	0.000127608	0.000159795	0.001646135	0.000145207	0.000195838	0.002333011	0.000444282
Thalassotale	0	0	0	0	0	0	0	0	0
Thiopseudo	0	0	0	0	0	0	0	0	0
Tissierella	0	0	0	0	0	0.000304936	0.000352508	0.000638978	0.000287476
Turicibacter	0.000277001	0.000474265	0.000382824	0.00046607	0.000107943	0.000174249	0.000143614	0.002927409	0.000483483
Tyzzerella	0.000158286	0.00019969	0.000523193	0	0.002617624	0.000493705	0.000326396	0.000490378	0.000718691
Tyzzerella 3	0.000474859	0.000124807	0.000242455	0.000399489	0.000337323	0.000174249	6.53E-05	0.000163459	0.000731758
Tyzzerella 4	0.001411386	0.001310469	0.002143814	0.003262491	0.002347766	0.002628254	0.000456955	0.000505238	0.000431215
UBA1819	0.00050124	0.000748839	0.002386269	0.000332907	0.010632413	0.000377539	0.000391676	0.000416078	0.001881664
uncultured	0.029639107	0.006252808	0.008217954	0.004860445	0.014923158	0.005808297	0.00321174	0.017683335	0.01289724
uncultured b	0.000118715	0.00018721	0.000382824	0.000226377	0.000337323	0.001931259	0.002062825	0.004532283	0.002796362
uncultured b	0	0	0	0	0.000121436	0	0	0.00011888	0
uncultured E	0	0	0	0	0	0	0	0	0
uncultured E	0	0	0	0	0	0	0	0	0
uncultured C	0	0	0	0	0	0	0	0	0
uncultured C	0	0	0	0	0	0	0	0	0
uncultured g	0	0	0	0	0	0	0	0	0
uncultured r	0	0	0	0	0	0	0	0	0
uncultured o	0.000171477	0.000174729	0.000216934	0.00035954	0.001605656	0.002613734	0.000391676	0.003819006	0.000378946
uncultured F	0	0	0	0	0.000242872	0	0	0.000980756	0
uncultured p	0	0	0	0	0	0	0	0	0
uncultured r	0	0	0	0	0	0	3.92E-05	2.97E-05	0
uncultured S	0	0	0	0	0	0	0	0	0
uncultured T	0	0	0	0	6.75E-05	0	0	0	0
uncultured V	0	0	0	0	0	0	0	0	0
unidentified	0	0	0	0	0	0.000246853	0.000143614	0.000401219	0.000313611
Veillonella	0.000699098	0.000536668	0.000255216	0.00175775	0.000107943	0	0.001122803	0.000534958	0
Vibrio	0	0	0	0	0	0	0	0	0
Victivallis	0	0	0	0	6.75E-05	0.000261373	7.83E-05	0.000312059	5.23E-05
W5053	0	0	0	0	0	0	0	0	0
Weissella	0.000237429	0	0.000561475	0.000146479	0.000553209	0.000174249	0.000261117	0.000445798	0.000300544
Wenyingzhu	0	0	0.000114847	0	0	0	0	0	0
Woeseia	0	0	0	0	0	0	0	0	0
Genus	W22	W24	W26	W29	W2	W33	W37	W39	W41
[Caedibacte	0	0	0	0	0	0	0	0	0
[Clostridium	7.50E-05	5.33E-05	0	2.72E-05	7.33E-05	0.000106104	0	0.000246066	0
[Eubacteriu	0	0	0	0	0	0	0	5.47E-05	0
[Eubacteriu	0.005335966	0.00585427	0.036519149	0.002992424	0.016134548	0.006631476	0.062287575	0.001790816	0.000640648
[Eubacteriu	0.000349899	0.005760922	0.007946968	0.005318353	0.049893739	0.011724449	0.004465365	0.014422223	0.002511339
[Eubacteriu	0.001349612	0.000840134	0.001489233	0.000340048	0.001282458	0.000862092	0.000967022	0.001052617	0.000576583
[Eubacteriu	0.00017495	0.000320051	0.000184507	0	9.77E-05	0	0.000255976	0	0
[Eubacteriu	0.001324619	0.001466901	0.007129866	0.001686639	0.000757261	0.050067641	0.006925582	0.002829763	0.000499705
[Eubacteriu	0.000724792	0.001266869	0.001752814	0.000408058	0.000781689	0.000915144	0.000583057	0.001052617	0.000243446
[Eubacteriu	0.000287417	0.000266709	0.002714884	0.000190427	0.000806117	0.004151304	0.000739487	0.000177715	0.00012813
[Polaribacte	0	0	0	0	0	0	0	0	0
[Ruminococ	0	0	0.000645773	0	0.000317561	0.000530518	0.001166114	0	0
[Ruminococ	0.000724792	0.00100016	0.000685311	0.000720902	0.000989325	0.003382053	0.000767929	0.002952796	0.00758527
[Ruminococ	0.002911663	0.008134635	0.022799758	0.001469008	0.005361898	0.004111515	0.00250288	0.011086656	0.001332547
Abiotrophia	0	0	0	0	0	0	0	0	0
Acetanaerol	0	6.67E-05	2.64E-05	0	2.44E-05	0	7.11E-05	0	0
Acetitomacu	0	0	0	0	0	0.000623359	0	0	0
Acetobacter	0	0	0	0	0	0	0	0	0
Acidaminoc	0	0.001640262	0	0.068757736	0.000500769	0.000915144	0.001109231	0.001271343	0.004125772
Acinetobact	0	0	0	2.72E-05	0	0	0	0	0
Actinobacillu	0	0	0	0	0	0	0	0	0
Actinomyce	5.00E-05	0.000106684	0.00014497	9.52E-05	0.000305347	0.0003581	0.001336765	0.000478462	6.41E-05
Adlercreutzi	6.25E-05	0	0.000210865	0	0	0	0.000284418	0.000410111	3.84E-05
Aeromonas	0	0.000266709	0	9.52E-05	0	0	0	4.10E-05	0
Aerosphaer	0	0	0	0	0	0	0	0	0
Aestuariicel	0	0	0	0	0	0	0	0	0
Agathobacte	0.001712008	0.004880781	0.022667967	0.00303323	0.00467792	0.002612801	0.003498343	0.029322907	0.003818261
Aggregatiba	0	0	0	0	0	0	0	0	0
Akkermansi	0.000999713	0.004760762	0.002846675	0.002461949	0.000415272	0.012971166	0.098678878	0.000874903	0.000704713
Alcaligenes	0	0	0	0	0	0	0	0	0
Alcanivorax	0	0	0	0	0	0	0	0	0
Alistipes	0.003149095	0.02212354	0.032934447	0.024442661	0.01419254	0.024602775	0.086207142	0.035283181	0.010775696
Allisonella	0.000112468	0	0.000263581	0.000720902	0.000293133	0	0.000184872	0.000697188	0.000871281
Allobaculum	0	0	0	0	0	0	0	0	0
Alloprevotel	0	0	0	0	0.036360749	0	0	0	0
Allorhizobiu	0	0	0	0	0	0	0	0	0
Alloscardov	0	0	0	0	0	0	0	0	0
Amphritea	0	0	0	0	0	0	0	0	0
Anaerofilum	0	0	0	0	0	0	0	0	0

Anaeroglobu	0	0	0	0	0	0	0	0	0.000115317
Anaerospor	0	0.000320051	0.002003216	0.000190427	0.000207636	7.96E-05	0	0.002064223	0
Anaerostipe	0	0.000280045	0.000527162	0	0.000390844	0.000490729	0.000810592	0.000587825	0.000499705
Anaerotrunc	0	0.000226703	0.000171328	0	0.000122139	0.00013263	0.000839034	0	6.41E-05
Angelakisell	0	0	0.000184507	0	0.000207636	0	0.000199093	0.000123033	0
Anoxybacillu	0	0	0	0	0	0	0	0	0
Anseongella	0	0	0	0	0	0	0	0	0
Arenibacter	0	0	0	0	0	0	0	0	0
Atopobium	0	0.000120019	0	0.000190427	0	9.28E-05	0	0.000136704	0.000102504
Atopostipes	8.75E-05	0	0	0	0	0	0	0	0
Aureimarina	0	0	0	0	0	0	0	0	0
Azospirillum	0.000249928	0.001066837	0.000316297	0.000435262	0	0.000212207	0.024474182	0	0
Bacillus	0	0	0	0	0	0	0	0	0
Bacteroides	0.490758907	0.576118846	0.150728801	0.398740462	0.146383467	0.398379267	0.255620814	0.456097661	0.656920278
Balneola	0	0	0	0	0	0	0	0	0
Barnesiella	0.001224648	0.001373553	0.012190622	0.002053891	0.004226006	0.006485583	0.005631479	0.000437451	0.001729749
Bifidobacter	0.000112468	0.000133355	0.000329476	0.000680096	0.000403058	0.000344837	0.001763393	0.002118905	0.000320324
Bilophila	0.000699799	0.002720435	0.001080682	0.005127926	0.001477881	0.001671132	0.003612111	0.000656177	0.001063475
Blautia	0.015145646	0.003667253	0.00846095	0.003278064	0.007560398	0.011180668	0.016325602	0.013916419	0.002524152
Bosea	0	0	0	0	0	0	0	0	0
Brevibacillus	0	0	0	0	0	0	0	0	0
Brevundimo	0	0	0	0	0	0	0	0	0
Butyricicocc	0.021206403	0.001613592	0.007406626	0.001469008	0.002161858	0.005318443	0.001749172	0.005645856	0.000615022
Butyricimon	0.00017495	0.000386729	0.007419805	0.000326446	0.002161858	0.000464203	0.00046929	0.000546814	0.000576583
Butyrivibrio	0	0	0.001568307	0	0	0	0	0	0
C1-B045	0	0	0	0	0	0	0	0	0
CAG-352	0	0	0	0	0	0	0	0	0
CAG-56	0.003761419	0.000493412	0.000935713	0.000176825	0.000354203	0.000384626	0.00046929	0.000929584	0
CAG-873	0	0	0	0	0	0	0	0	0
Campylobac	0	0	0	0	0	0	0	0	0
Candidatus	0	0	0	0	0	0	0	0	0
Candidatus	0	0	0	0	0	0	0	0	0
Candidatus	0	0	0	0	0	0	0	0	0
Candidatus	0	0	0	0	0	0	0	0	0
Candidatus	0	0	0	0	0	0	0	0	0
Candidatus	0	5.33E-05	0.000197686	0	0	0.000185681	9.95E-05	0	0
Candidatus	0.000112468	0	0	0	0	0	0	5.47E-05	0.000294698
Caproicipro	0	0	0.000355834	0	0	0	0	0	0
Catabacter	0	0	0	0	0	0	0	0	0
Catenibacte	0	0	0	0.000448864	0	0	0	0	0
Cellulosilytic	0	0	0	0	0	0	4.27E-05	0	0
Cellvibrio	0	0	0	0	0	0	0	0	0
Cephalotico	0	0	0	0	0	0	0	0	0
Christensen	0	0	0	0	0	0	0	0	0
Christensen	0.002024418	0.002320371	0.031721975	0.0021219	0.006925275	0.003342264	0.050313571	0.001613102	0.000794403
Chryseoline	0	0	0	0	0	0	0	0	0
Chthoniobac	0	0	0	0	0	0	0	0	0
Clavibacter	0	0	0	0	0	0	0	0	0
Cloacibacillu	0	0	0	0	0	0	0	0	0
Clostridiales	0	0	0	0	0	0	0	0	0
Clostridioide	0	0	0	0	0	0	0	0	0
Clostridium	0.00062482	0.000986825	0.002372229	0.000557679	0.003004617	0.002281228	0.001464753	0.020109089	0.000743151
Collinsella	0.000324907	0.000320051	0.001001608	0.001047348	0.000488556	0.000663148	0.001137673	0.00076554	0.000269072
Colwellia	0	0	0	0	0	0	0	0	0
Comamona	0	0	0	0	0.000109925	0	0	0.000109363	0
Coprobacillu	0	0	6.59E-05	0	0	9.28E-05	9.95E-05	0	0
Coprobacte	0	0.000186697	0.002200901	0.000163223	0.000842758	0.000291785	0.000241755	0.002474334	0.00021782
Coprococcu	0	0.000453406	0.000658953	0	0.000378631	0.000663148	0.00031286	0.000642507	0
Coprococcu	0.000324907	0.000306716	0.005482485	9.52E-05	0.001844297	0.000557044	0.00015643	0.000451122	0
Coprococcu	0.000199943	0.000893476	0.002438124	0.000612087	0.004433642	0.001114088	0.000839034	0.000232396	0.000281885
Coriobacter	0	0	0	0	0	0	0	0	0
Coxiella	0	0	0	0	0	0	0	0	0
Crocinitomix	0	0	0	0	0	0	0	0	0
Cyclobacter	0	0	0	0	0	0	0	0	0
Defluviitalea	0	0	6.59E-05	0	0	7.96E-05	0.00015643	0	0
Deinococcu	0	0	0	0	0	0	0	0	0
Delftia	0	0	0	0	0	0	0	0	0
Desulfovibri	0.000274921	0.001680269	0.002741243	0.000965737	0.010259667	0.000702936	0.0119029	0.001025276	0.000653461
Dialister	0.002999138	0.00144023	0.00250402	0.072933527	0.041966925	0.00427067	0.003085937	0.031455482	0.004010455
Dielma	0.000149957	0	0	0	8.55E-05	9.28E-05	5.69E-05	0	0
Dolosicoccu	0	0	6.59E-05	0	0	0	0	0	0
Donghicola	0	0	0	0	0	0	0	0	0
Dorea	0.005485923	0.001186857	0.003900999	0.000938533	0.003603097	0.00220165	0.002545542	0.004114776	0.000358763
DTU089	0	9.33E-05	7.91E-05	0	0	0	0	0	0
Dubosiella	0	0	0	0	0	0	0	0	0
Dysgonomo	0	0	0	0	2.44E-05	0	0	0	0
Eggerthella	0.000224935	0.000373393	0.00027676	0.000136019	0.000109925	0.000172418	9.95E-05	8.20E-05	6.41E-05
Eisenbergie	0.000199943	0.000533419	0.000777564	0.000272039	0.000244278	0.0003581	0.000895917	0	0.000550957
Enhydrobac	0	0	0	0	0	0	0	0	0
Enterococcu	0	0	0.000303118	0	0	0	0	0	0
Enterorhabd	0	0.000133355	0	0	0	0	0	0	0

Epulopisciu	0	0	0	0	0	0	0	0	0
Erysipelatoc	0.000749784	0.002053662	0.001502412	0.000340048	0.000525197	0.000623359	0.001180335	0.000628836	0.000140943
Erysipelotric	0.007672794	0.00600096	0.002385408	0.000911329	0.000964897	0.014509669	0.007608186	0.000587825	0.000205007
Erysipelotric	0	0	0	0	0	0	0	0	0
Escherichia-	0.064181548	0.014589001	0.0080524	0.006719351	0.06930161	0.01757341	0.010608797	0.006438736	0.019052866
Eubacterium	0	0	2.64E-05	0	0	0	0	0	0
Ezakiella	0	0	0.000105432	0	0	0	0	0	0
Fabibacter	0	0	0	0	0	0	0	0	0
Faecalibact	0.026767304	0.005494212	0.025751865	0.006596934	0.031804969	0.026645269	0.024388856	0.028037894	0.003907952
Faecalibacu	0	0	0	0	0	0	0	0	0
Faecalicocc	0	0	0	0	0	2.65E-05	0	0	0
Faecalitalea	0.00017495	0	6.59E-05	0	0.000146567	0.000251996	8.53E-05	8.20E-05	0
Family XIII A	0	9.33E-05	0.000658953	0.000136019	0.000390844	0.00022547	0.000284418	0	0
Family XIII U	0	9.33E-05	0.00055352	0.000176825	0.000280919	0.000119367	0.000270197	0.000300748	0
Flavonifract	0.001337116	0.000240038	0.000250402	0.000530475	0.000793903	0.001193666	0.000839034	0.000533144	0.002152577
Formosa	0	0	0	0	0	0	0	0	0
Fournierella	0	0	0	0	0.000928256	0	0	0.000123033	0
Fusicateniba	0.005248491	0.000866805	0.001120219	0.000516873	0.001331314	0.00346163	0.006470513	0.002515345	0.000307511
Fusobacteri	0.004398735	0.083706726	0.001133398	4.08E-05	0.000964897	0.000875355	0.000241755	0.004907657	0.001524742
GCA-90006	8.75E-05	0	0.000579878	0	0	0.000371363	0.000327081	0	0
GCA-90006	0	0	0	0	0	0.000318311	0	0	0
Gemella	0	0.000253374	6.59E-05	0	0.000207636	0.000106104	0	0.000246066	0.000166568
Gimesia	0	0	0	0	0	0	0	0	0
Gluconobac	0	0	0	0	0	0	0	0	0
Gordonibact	0	0	0	0	0	9.28E-05	7.11E-05	0	0
Gottschalkia	0	0	0	0	0	0	0	0	0
Granulicatel	8.75E-05	0	0	0	0.000122139	0	0	0.000123033	0
Haemophilu	5.00E-05	4.00E-05	0.000395372	0.000476067	0.000256492	0.00045094	0.000383965	0.000314418	0.000307511
Harryflintia	0	0	0	0	0	0	0	0	0
hgcI clade	0	0	0	0	0	0	0	0	0
Hoeflea	0	0	0	0	0	0	0	0	0
Holdemanel	0.000437374	0.000613431	0.01914916	0.001183368	0.003603097	0.000862092	0.001450533	0	8.97E-05
Holdemania	5.00E-05	0	0.000171328	0	0.000146567	0.000238733	0.000227535	0	7.69E-05
Howardella	0	0	0	0	7.33E-05	0	0	0	0
Hungatella	0	0.00029338	0.000711669	0	0.000671764	0.000291785	0.000255976	0.000410111	0.00034595
Hydrogenoa	0	0.000173361	0.000513983	0	0.000183208	0.000119367	0.000213314	5.47E-05	0
Ileibacterium	0	0	0	0	0	0	0	0	0
Intestinibact	0.000524849	0.00056009	0.000843459	0.000816116	0.001172533	0.001021247	0.001279882	0.000656177	0.000269072
Intestinimor	0	0	9.23E-05	0.000136019	0.000378631	0.000119367	0.004337377	0.000123033	0
Jeotgalibaca	0	0	0	0	0	0	0	0	0
Kordiimonas	0	0	0	0	0	0	0	0	0
Lachnoclost	0.018257251	0.004974129	0.003531986	0.00692338	0.00577717	0.009005544	0.00580213	0.008667004	0.034338723
Lachnoclost	0	0	0.000909354	0	0	0	0	0.001818157	0.000397202
Lachnoclost	0	0	0	0	0	0	0	0	0
Lachnospira	0.096872149	0.016402624	0.013982973	0.00508712	0.041392872	0.189355155	0.009371578	0.048420391	0.002857289
Lachnospira	0.00031241	6.67E-05	0.000237223	5.44E-05	0.000354203	0.000265259	0.000142209	0.000423781	0
Lachnospira	0.001374605	0.000453406	0.000909354	0.000312844	0.000696192	0.001817024	0.000383965	0.000806551	0.00012813
Lachnospira	0.003161591	0.004200672	0.037705264	0.000924931	0.018467401	0.009005544	0.00359789	0.004743613	0.000653461
Lachnospira	0.012483911	0.000320051	0.003162972	0.000503271	0.002283997	0.006299902	0.000583057	0.001367035	0.000333137
Lachnospira	0.000199943	0	0.003136614	0	0	0.000238733	0	0.000314418	0.000192194
Lachnospira	0.021143921	0.004013976	0.004731279	0.000870523	0.001673303	0.00301069	0.001222998	0.00270673	0.000512518
Lachnospira	0	0	0	0	0	0	0	0	0
Lachnospira	0.000224935	0.003827279	0.002332692	0.0007073	0.002980189	0.00080904	0.001606963	0.004948668	0.000666274
Lacticigeniu	0	0	0	0	0	0	0	0	0
Lactobacillu	0.000387389	0.000373393	0.001014787	6.80E-05	0.000134353	0.000477466	0.000327081	0.002105234	0.00012813
Lactococcus	0	0.000133355	0	0.000258437	0	0	0	0	0
Lentibacter	0	0	0	0	0	0	0	0	0
Leuconosto	0	0	0	0.000176825	3.66E-05	5.31E-05	0	0	0
Leucothrix	0	0	0	0	0	0	0	0	0
Litoricola	0	0	0	0	0	0	0	0	0
Mailhella	0	0.000160026	0	0	0	0	0	0	0
Mannheimia	0	0	0	0	0	0	0	0	0
Maribacter	0	0	0	0	0	0	0	0	0
Marinicella	0	0	0	0	0	0	0	0	0
Marinobacte	0	0	0	0	0	0	0	0	0
Marivita	0	0	0	0	0	0	0	0	0
Marvinbryar	0	0	0.00014497	0	0	0	0	0.000191385	0
Massilia	0	0	0	0	0	0	0	0	0
Megamonas	0.000149957	0.000840134	0.000448088	0.006855371	0.001661089	0.021207459	0.002062032	0.002460664	0.004189837
Megasphae	0.00194944	0.007401184	0.003149793	0.240672479	0.003542028	0.004297196	0.004138284	0.003814029	0.069215591
Merdibacter	0	0	0	0	0	0	0	0	0
Methanobre	0	5.33E-05	0.001541949	0	8.55E-05	0	0	0	0
Methylobact	0	0	0	0	0	0	0	0	0
Methylotene	0	0	0	0	0	0	0	0	0
Mf105b01	0	0	0	0	0	0	0	0	0
Micrococcus	0	0	0	0	0	0	0	0	0
Mitsuokella	0	0	0	0	0.000122139	0	0	0.001804487	0.001447864
Mogibacteri	0	0	9.23E-05	0	8.55E-05	0	0	9.57E-05	0
Morganella	0	0	0.000171328	0	0	0	0	0	0
Moryella	0	0	0.000329476	0	0.000683978	0	0	0	0
Murimonas	0	0	0	0	0	0	0	0	0

Mycobacter	0	0	0	0	0	0	0	0	0
Negativibac	0.000124964	0.000586761	0.000461267	0.000231233	0.000952683	0.000517255	0.000355523	0	3.84E-05
Nitrosomona	0	0	0	0	0	0	0	0	0
Nosocomiic	0	0	0	0	0	0	0	0	0
NS3a marin	0	0	0	0	0	0	0	0	0
Oblitimonas	0	0	0	0	0	0	0	0	0
Oceanicocc	0	0	0	0	0	0	0	0	0
Oceanobact	0	0	0	0	0	0	0	0	0
Oceanospiri	0	0	0	0	0	0	0	0	0
Odoribacter	0.002961649	0.000960154	0.002108648	0.000911329	0.004409214	0.001830287	0.002787298	0.002556356	0.000820029
Olsenella	0	0	0	0	0	0	4.27E-05	0	0
OM27 clade	0	0	0	0	0	0	0	0	0
Oribacteriur	0	0	0	0	0.000122139	0	0	0	0
Oscillibacte	6.25E-05	0.000160026	0.000461267	0.000217631	0.000891614	0.001286506	0.001151894	0.000451122	7.69E-05
Oscillospira	0	0	0	0	0	0	0.000227535	0	0
Ostreobium	0	0	0	0	0	0	0	0	0
Oxalobacter	0	0.000133355	0.000237223	0	9.77E-05	7.96E-05	0.000127988	0	0
Paenalcalig	0	9.33E-05	5.27E-05	0	0	0	0	0	0
Paeniclostri	0	0	0	0	0	0	0	0.000259737	0
Parabacterc	0.00821014	0.025244039	0.020032157	0.020457297	0.017404793	0.011764238	0.046317496	0.017238315	0.010160674
Paraclostrid	0	0	0	0	0	0	0	0	0
Paracoccus	0	0	0	0	0	0	0	0	0
Parahaliea	0	0	0	0	0	0	0	0	0
Paralcaliger	0	0	0	0	0	0	0	0	0
Paraprevote	0.000124964	0	0.001950499	0.001264979	0.003786306	0.001538502	0.01880004	0.010211754	0.000550957
Parasuttere	0.003873886	0.009574865	0.00193732	0.047443518	0.004311503	0.008819863	0.00470712	0.020013397	0.059464931
Patulibacter	0	0	0	0	0	0	0	0	0
Pediococcu	0	0	0	0	0	0	0	0	0
Pedobacter	0	0	0	0	0	0	0	0	0
Peptoclostri	0	0	0	0	0	0	0	0	0
Peptococcu	1.00E-04	0	0.000408551	0	0	0.000119367	2.84E-05	5.47E-05	0
Phascolarct	0.023293303	0.004400704	0.003031182	0.003373278	0.001514522	0.003832993	0.01159004	0.003034818	0.038438869
Phocea	0.00013746	8.00E-05	0	6.80E-05	0.000183208	0	0	0	0
Planctomicr	0	0	0	0	0	0	0	0	0
Polynucleob	0	0	0	0	0	0	0	0	0
Porticoccus	0	0	0	0	0	0	0	0	0
Prevotella	0	0	0	0	0	0	0	0	0
Prevotella 2	0.00066231	0.009068118	0.000764385	0.001577823	0.067359601	7.96E-05	0.000170651	0	0.002588217
Prevotella 6	0	0	0	0	6.11E-05	0	0	0	0
Prevotella 7	1.00E-04	0.000200032	0.000105432	0	0.000537411	0	0	0	0
Prevotella 9	0.001886957	0.049221209	0.125886291	0.002380337	0.13458485	0.00013263	0.000597278	0.02478435	0.009148451
Prevotellace	0.000124964	0.006601056	0.000131791	0	0	0	0	0	0
Prevotellace	0	0	0	0.000231233	0.000696192	0.000106104	0.011305622	0	0
Prevotellace	0	0	0	0	0.000280919	0	0	0	0
Prevotellace	0	0	0	0	0	0	0	0	0
Proteocatell	0	0	6.59E-05	0	0	0	2.84E-05	0	0
Proteus	0	4.00E-05	0	0	0	0	0	6.84E-05	7.69E-05
Providencia	0	0	0	0	0	0	0	0	0
Pseudoalter	0	0	0	0	0	0	0	0	0
Pseudochro	0	0	0	0	0	0	0	0	0
Pseudoflavo	0	0	0	0	0	0	0.000184872	0.0003691	0
Pseudohong	0	0	0	0	0	0	0	0	0
Pseudomon	0	0	0	0	4.89E-05	0	0	0	0
Pseudophae	0	0	0	0	0	0	0	0	0
Pseudovibri	0	0	0	0	0	0	0	0	0
Pyramidoba	0.000249928	0.002467061	0.000434909	0.000884125	0.000488556	0.000106104	9.95E-05	2.73E-05	0.000589396
Raoultibacte	0	0	0	2.72E-05	0	0	0	0	0
Reichenbac	0	0	0	0	0	0	0	0	0
Rheinheime	0	0	0	0	0	0	0	0	0
Rhodococcu	0	0	0	0	0	0	0	0	0
Rhodoferax	0	0	0	0	0	0	0	0	0
Rikenellace	0	0	0	0	0.000867186	6.63E-05	7.11E-05	0	0
Romboutsia	0.000924734	0.001120179	0.002319513	0.001142562	0.001966436	0.002904586	0.00359789	0.004716272	0.000371576
Roseburia	0.053397148	0.007174481	0.019808113	0.00338688	0.070755063	0.026353484	0.003967633	0.012221296	0.001294109
Roseibacillu	0	0	0	0	0	0	0	0	0
Rothia	0	0	0	0	0	0	0	0	0
Ruegeria	0	0	0	0	0	0	0	0	0
Ruminiclost	0	0	0	0	0	0	0	0	0
Ruminiclost	0	0	0.00042173	0	4.89E-05	0	0.000383965	0	0
Ruminiclost	0.001212152	0.000773457	0.001858246	0.000680096	0.001709945	0.000875355	0.003811203	0.000287077	0.00090972
Ruminiclost	0.000187446	0.000200032	0.000738027	0.000394456	0.001184747	0.000477466	0.008091696	0.000505803	0
Ruminiclost	0.00017495	0.000413399	0.001133398	0.000136019	0.001343528	0.001405873	0.001151894	0.00079288	0
Ruminococ	0.001499569	0.001160186	0.033514326	0.004760674	0.007829103	0.003673838	0.004650237	0.003034818	0.001422238
Ruminococ	0.003099109	0.003880621	0.04011703	0.002013085	0.009832181	0.003222897	0.01143361	0.005249416	0.002139764
Ruminococ	0.00031241	0.001013495	0.002095469	0.000816116	0.001294672	0.001485451	0.0015643	0.000642507	0.000371576
Ruminococ	7.50E-05	0.000160026	0.000434909	0.000204029	0.00021985	0.000212207	0.000455069	0.000273407	7.69E-05
Ruminococ	0.001149669	0.000813463	0.019478637	0.00148261	0.006644356	0.002493435	0.010878994	0.001011606	0.000858468
Ruminococ	0	0	0.000158149	0	0	0	0	0	0
Ruminococ	0.000187446	0.000213367	0.003821925	5.44E-05	0.001038181	7.96E-05	0.004195168	0.000164044	6.41E-05
Ruminococ	0.010384514	0.000613431	0.005060755	0.001781852	0.002540489	0.00602138	0.001493195	0.003007478	0.000589396
Ruminococ	0.004323757	0.003800608	0.102019031	0	0.006754281	0.000543781	0.001621183	0	6.41E-05

Ruminococc	0.000999713	0.010241639	0.008236907	0.001305785	0.005227545	0.005915276	0.00219002	0.022310016	0.002754786
Ruminococc	0.001049698	0.012682029	0.008302802	0.002353133	0.005386325	0.00660495	0.00359789	0.032480759	0.001447864
Sanguibacte	0	0	0.000724848	0	0	0	0	0	0
Sarcina	0	0	0	0	0	0	0	0	0
Sediminibac	0	0	0	0	0	0	0	0	0
Selenomona	0	0	0	0	0	0	0	9.57E-05	0.000281885
Sellimonas	0	0	0	0	0	0	0	0	0
Senegalima	0	0.000160026	0.000224044	0	0	0	0.000199093	0	0
Serratia	0	0	0	0	0	0	0	0	0
Shuttleworth	0	0	0.000210865	0	0	0	0	0	0
Slackia	7.50E-05	5.33E-05	0.001700098	0	4.89E-05	0.000185681	0	6.84E-05	0
Sneathiella	0	0	0	0	0	0	0	0	0
Solobacteriu	6.25E-05	5.33E-05	3.95E-05	0	4.89E-05	7.96E-05	9.95E-05	0.000191385	0
Sphingobac	0	0	0	0	0	0	0	0	0
Sphingomor	0	0	0	0	0	0	0	0	0
Staphylococ	0	0	0	0	0	0	0	0	0
Stenotropho	0	0	0	0	0	0	0	0	0
Streptococc	0.001886957	0.002573745	0.004045969	0.002801997	0.039560789	0.006352954	0.003939191	0.009774302	0.001383799
Subdoligran	0.012821314	0.002107004	0.012757321	0.00402617	0.009966534	0.005398021	0.015586115	0.004142117	0.001832253
Succinatimo	0	0	0	0	0	0	0	0	0
Succinivibrio	0	0	0	0	0	0	0	0	0
Sulfitobacte	0	0	0	0	0	0	0	0	0
Sutterella	0.00403634	0.015029071	0.003571523	0.001264979	0.002931334	0.001737447	0.021430908	0.028516357	0.001857879
Synergistes	0	0	0	0	0	0	0	0	0
Taeseokella	0	0	0	0	0	0	0	0	0
Tepidimicro	0.00013746	0	0.000158149	0	0	0	0	0	0
Terrisporob	0	0.00014669	0.000237223	0.000136019	0.000280919	0.0003581	0.000142209	8.20E-05	0
Thalassotal	0	0	0	0	0	0	0	0	0
Thiopseudo	0	0	0	0	0	0	0	0	0
Tissierella	0.000224935	0.000333387	0.000527162	0	0	0	0	0	0
Turicibacter	0.00017495	8.00E-05	0.000369013	0	0.000390844	7.96E-05	0.00015643	0	0
Tyzzerella	0.012246479	0.000213367	0.000645773	0.000122417	0.000109925	0.000265259	0	0	0
Tyzzerella 3	0.000574835	0.000213367	5.27E-05	0	4.89E-05	0.00220165	0.00031286	0.000341759	3.84E-05
Tyzzerella 4	0.000512353	0	0.000500804	0.000312844	0.000586267	0.000968195	0.000369744	0.000601496	0.000397202
UBA1819	0.00017495	0.001680269	0.000303118	0.000516873	0.000781689	0.001710921	0.00203359	0.000642507	0.000140943
uncultured	0.009359809	0.004507388	0.01207201	0.001822658	0.00709627	0.0040452	0.034087515	0.005167393	0.006726802
uncultured b	0.001312123	0.001546914	0.00888268	0.000489669	0.005728314	0.00080904	0.009499566	5.47E-05	0
uncultured b	0	0	0.001172936	0	0.000170994	0	9.95E-05	0	0
uncultured E	0	0	0	0	0	0	0	0	0
uncultured E	0	0	0	0	0	0	0	0	0
uncultured C	0	0	0	0	0	0	0	0	0
uncultured C	0	0	0	0	0	0	0	0	0
uncultured g	0	0	0	0	0	0	0	0	0
uncultured r	0	0	0	0	0	0	0	0	0
uncultured o	0.000224935	0.000306716	0.003347479	0	0.0017588	0	0.002616647	0.001982201	0.000243446
uncultured F	0	0	0	0	0.000390844	0	0	0	0
uncultured p	0	0	0	0	0	0	0	0	0
uncultured r	0	0	5.27E-05	0.000217631	0	0	0	0	0
uncultured S	0	0	0	0	0	0	0	0	0
uncultured T	0	8.00E-05	0.000197686	0	0	0	8.53E-05	0	0
uncultured V	0	0	0	0	0	0	0	0	0
unidentified	0	0.000253374	0.00694536	0	0	0	0	0	0
Veillonella	0.001212152	0.000880141	0.000184507	0.000503271	0	0.000464203	0.000270197	0.001257672	0.002178203
Vibrio	0	0	0	0	0	0	0	0	0
Victivallis	2.50E-05	4.00E-05	5.27E-05	0	0	0	8.53E-05	6.84E-05	0
W5053	0	0	9.23E-05	0	0	0	0	0	0
Weissella	5.00E-05	6.67E-05	0.000184507	0.000462465	0.000256492	0.00058357	9.95E-05	4.10E-05	0.000307511
Wenyingzhu	0	0	0	0	0	0	0	0	0
Woeseia	0	0	0	0	0	0	0	0	0
Genus	W46	W50	W52	W55	W5	W60	W62	W64	W67
[Caedibacte	0	0	0	0	0	0	6.36E-05	0	0
[Clostridium	0	0	9.00E-05	0	8.59E-05	0.000126662	6.36E-05	6.34E-05	0
[Eubacteriu	0	0	0	0	4.91E-05	0	0	0	0
[Eubacteriu	0.011650033	0.000337314	0.00488344	0.005243904	0.022973553	0.010024428	0.0082383	0.005602241	0.002895601
[Eubacteriu	0.002983096	0.001025433	0.001850567	0.010126702	0.01306989	0.006061703	0.084302732	0.013270467	0.023697888
[Eubacteriu	0.001153955	0.000391284	0.000282725	0.001632832	0.005448856	0.001664706	0.001156922	0.002332155	0.000484619
[Eubacteriu	0	0	0	0	0.000159539	0	0.000127134	0	0
[Eubacteriu	0.001841417	0.000283343	0.000950986	0.003454069	0.000490888	0.002334208	0.008251014	0.002978567	0.001526551
[Eubacteriu	0.001166231	0.000310329	0.001490734	0.000832117	0.001656747	0.000199041	0.000864513	0.000950606	0.001514436
[Eubacteriu	0.000208694	0.000202388	0.000257023	0.003877977	0.002061729	0.001067583	0.002339271	0.005044552	0.00037558
[Polaribacte	0	0	0	0.001664233	0	0	0.000775519	0.002040635	0
[Ruminococ	0.000306903	0	0	0	0.00039271	0.000180946	0.000533964	0.000570364	0
[Ruminococ	0.001387201	0.002455643	0.001837716	0.001460129	0.001276309	0.006043608	0.001309483	0.008187891	0.001247895
[Ruminococ	0.005794326	0.002158807	0.007016732	0.003469769	0.00720378	0.004469375	0.002682533	0.005576891	0.011679327
Abiotrophia	8.59E-05	0.000148418	0	0	0	0	0	0	0
Acetanaerol	0	0	0	0	0	0	0	0	0
Acetitomacu	0	0	0	0	0	0	0	0.00024082	0
Acetobacter	0	0	0	0	0	0.000108568	7.63E-05	0.000114073	4.85E-05
Acidaminoc	0.000466492	0.000283343	0.00021847	0	0.000429527	0	0	6.34E-05	0
Acinetobact	0	0	0	0.000125602	0	0.000126662	7.63E-05	0.000152097	4.85E-05

Actinobacillu	0	0	0	0	0	0	0	0	0
Actinomyce	0.000122761	0	0.000604004	0.000423908	0.000147266	0.000886637	0.000241555	0.000177447	0.000193848
Adlercreutzi	0	0	0.000128512	0.000157003	0	0	0.00044497	0.000202796	0
Aeromonas	0.000245522	0	5.14E-05	0.000361107	0.000208627	0.0010133	0.000457683	0.000519665	0.015350319
Aerosphaer	0	0	0	0	0	0	0	0	0
Aestuariicel	0	0	0	7.85E-05	0	0	3.81E-05	0	0
Agathobacte	0.021618237	0.003508062	0.006836816	0.045609408	0.006099282	0.007853072	0.010628425	0.006261328	0.00512485
Aggregatiba	0	0	0	0	0	0	0	0	0.001005585
Akkermansi	9.82E-05	0	0.001760609	0.001460129	0.000638154	0.001628517	0.002517258	0.001875863	0.000775391
Alcaligenes	0	0	0	0	0	0	0	0	0
Alcanivorax	0	0	0	0	0	0	0	0.000202796	0
Alistipes	0.016867381	0.003130271	0.021358624	0.021572229	0.027747438	0.019632679	0.028617923	0.030926398	0.003683107
Allisonella	0.000503321	0	0.000205619	0.000157003	0.000920415	0	0	0.000304194	0
Allobaculum	0	0	0	0	0	0	0	7.60E-05	0
Alloprevotel	0	0	0	4.71E-05	0.00061361	0	8.90E-05	0	0
Allorhizobiu	0	0	0	0	0	0	0	0	0
Alloscardov	0	0	0	0	0	0	0	0	0
Amphritea	0	0	0	0	0	0	0.000317836	0.000164772	0
Anaerofilum	0	0	0	0	0	0	0	0	0
Anaeroglobu	0	0	0	0	0	0	0	0	0
Anaerospor	0.000233246	0.000134925	0.00011566	0.00048671	0.000576793	0.000832353	0.000546677	0.001153403	0.000278656
Anaerostipe	0.000368283	0.000323821	0.000398386	0.003893677	0.001693563	0.000651407	0.000622958	0.002940543	0.000484619
Anaerotrunc	0	0	0.000244172	0.000125602	0.000490888	0.000488555	0.000216128	0.000139422	4.85E-05
Angelakisell	0	0	0	0	0.000159539	0	0	0	0
Anoxybacillu	0	0	0	0	0	0	0	0	0
Anseongella	0	0	0	0	0	0	0	0	0
Arenibacter	0	0	0	0	0	0	0.000114421	0	0
Atopobium	0.000135037	0.00010794	0	0.000172703	0	0	0	0	0
Atopostipes	0	0	0	0	0	0	0	0	0
Aureimarina	0	0	0	0	0	0	0.000203415	0	0
Azospirillum	9.82E-05	0	0.001195158	0	0.000515432	0	0	0	0
Bacillus	0	0	0	0	0	0.000126662	0	0	0
Bacteroides	0.183000037	0.521109087	0.566504742	0.481010472	0.255863042	0.199457161	0.373571329	0.432804796	0.20116551
Balneola	0	0	0	0.000408208	0	0	0.000355976	6.34E-05	0
Barnesiella	0.001792313	0.000215881	0.00476778	0.004647292	0.005068418	0.00124853	0.008492569	0.007072512	0.000726929
Bifidobacter	0.000540149	0.000377791	0.019430951	0.00152293	0.004013009	0.00023523	0.000381403	0.005336071	0.00037558
Bilophila	0.000797947	0.000215881	0.00088673	0.001099022	0.001043137	0.001393287	0.000559391	0.000760485	0.000278656
Blautia	0.005757498	0.00360251	0.007248053	0.013988978	0.010235013	0.039355831	0.0090901	0.014702714	0.020681133
Bosea	0	0	0	0	0	0	0	0	0
Brevibacillus	0	0	0	0	0	0	0	0	0
Brevundimo	0	0	0	0	0	0	0	0	0
Butyricicocc	0.003277723	0.002496121	0.005448891	0.002653353	0.008885071	0.00246087	0.001983295	0.003701028	0.008468724
Butyricimon	0.001178507	0.000188896	0.000719665	0.001177523	0.000994048	0.001176151	0.001284056	0.001444922	0.000181732
Butyrivibrio	0	0	0	0.000282606	0	0.000325703	0.004678541	0.000152097	0.000109039
C1-B045	0	0	0	0	0	0	0	0	0
CAG-352	0	0	0	0.000251205	0	0.000253325	0.00322921	0.000291519	0.000157501
CAG-56	0.000613806	0.000499224	0.000321279	0.003548271	0.001718108	0.000361893	0.000902653	0.000608388	0.004567538
CAG-873	0	0	0	0	0	0	0	0	0
Campylobac	0	0	0	0	0.000257716	0	0	0	0
Candidatus	0	0	0	0	0	3.62E-05	7.63E-05	0.000735136	0
Candidatus	0	0	0	7.85E-05	0	0	0	0.000139422	0
Candidatus	0	0	0	0	0	0	8.90E-05	0	0
Candidatus	0	0	0	0	0	0	0	0	0
Candidatus	0	0	0	0	0	0	0	0	0
Candidatus	0	0	2.57E-05	0	0.00011045	7.24E-05	0	5.07E-05	0
Candidatus	0.000135037	0.002010389	0.000141363	0	0	0	0	0	0
Caproicipro	0	0	0	0	0.000306805	0	0	0	0
Catabacter	0	0	0	0	0	0.000126662	0	0	0
Catenibacte	0	0	0	0	4.91E-05	0	0	0	0
Cellulosilytic	0	0	0	0	0	0	0.000139848	0.000278845	0
Cellvibrio	0	0	0	0	0	0	0	0	0
Cephalotico	0	0	0	0.000141303	0	0	0	0	0
Christensen	0	0	0	0	0	0	0	0	0
Christensen	0.002602536	0.000836538	0.006387025	0.007347746	0.009682764	0.00273229	0.00586089	0.008086493	0.001708283
Chryseoline	0	0	0	3.14E-05	0	0	0	0	0
Chthoniobac	0	0	0	0	0	0	0	0	0
Clavibacter	0	0	0	0	0	0	0	0	0
Cloacibacillu	0	0	0	0	0	7.24E-05	0	0	0
Clostridiales	0	0	0	0	0	0	0	0	0
Clostridioide	0	0	0	0	0	0	0	0	0
Clostridium	0.003302275	0.000971463	0.001812013	0.003187163	0.002969872	0.002044694	0.007551775	0.017503834	0.005766971
Collinsella	0.00066291	0.000283343	0.00033413	0.000361107	0.001718108	0.003998914	0.000572104	0.001622368	0.000981354
Colwellia	0	0	0	0	0	0	5.09E-05	0	0
Comamona	0	0	0	0.000188404	0.00011045	0.00023523	0.000190701	0.000215471	0.000157501
Coprobacillu	0	0	0	0	0.000368166	0	0	5.07E-05	3.63E-05
Coprobacte	0.000650634	0.000175403	0.001272265	0.001884037	0.000834509	0.000488555	0.000165275	0.00074781	4.85E-05
Coprococcu	0.000233246	0	0.000244172	0.000580912	0.000147266	0.000162852	0.000572104	0.001191427	7.27E-05
Coprococcu	0.002909439	0	0.000154214	0.007944358	0.001264036	0.001411382	0.010641138	0.004600935	0.00075116
Coprococcu	0.001092574	0.000229373	0.003919603	0.003689573	0.001988096	0.003130372	0.001538325	0.00306729	0.000363465
Coriobacter	0	0	0	0	0	0	0	0	0
Coxiella	0	0	0	0.000109902	0	0	0	7.60E-05	0

Crocinitomix	0	0	0	0.000172703	0	0	0.00052125	0	0
Cyclobacter	0	0	0	0.000172703	0	0	0.000419543	0	0
Defluviitalea	0	0	0	0	0.000122722	0	0	6.34E-05	0
Deinococcu	0	0	0	0	0	0	0	0	0
Delftia	0	0	0	0	0	0	0	0	0
Desulfovibri	0.000356007	0	0.003392706	0.000690814	0.00100632	0.000832353	0.001729026	0.000811184	0.000399811
Dialister	0.00407567	0.001861971	0.000950986	0.001161823	0.005227956	0.00175518	0.008734124	0.003143339	0.001114625
Dielma	6.14E-05	5.40E-05	6.43E-05	9.42E-05	0.000171811	0.000108568	0	0	7.27E-05
Dolosicoccu	0	0	0	0	0	0	0	0	0
Donghicola	0	0	0	7.85E-05	0	0	0.006432994	0.005665615	0
Dorea	0.001964178	0.00074209	0.001092348	0.003579671	0.003264404	0.00171899	0.002771527	0.005209324	0.007766026
DTU089	0	0	0	0	0.000122722	0	0	0	0
Dubosiella	0	0	0	0.000125602	0	9.05E-05	7.63E-05	0	0
Dysgonomo	0	0	0	0	0	0	0	0	0
Eggerthella	0	0.00010794	0.000282725	0.000141303	0	0.000398082	0.000101707	0.000101398	8.48E-05
Eisenbergie	0	0	0.00033413	0.000141303	0	0.00023523	0.000139848	0	0
Enhydrobac	0	0	0	0	0	0	0	0	0
Enterococcu	0	0	0	0.000251205	0	0.00097711	0.000661098	0.00053234	0.000230194
Enterorhabd	0	0	0	0	0	0	0	0	0
Epulopisciu	0	0	0	7.85E-05	0	9.05E-05	0	6.34E-05	8.48E-05
Erysipelatoc	0.000294627	0.003548539	0.000642558	0.000753615	0.000404983	0.004939835	0.000648385	0.00103933	0.001102509
Erysipelotric	0.000675186	0.000836538	0.007273755	0.00453739	0.001583113	0.00050665	0.002186709	0.005424794	0.003743685
Erysipelotric	0	0	0	0	0	0	0	0	0
Escherichia-	0.005413766	0.004358092	0.013236693	0.004631592	0.009780941	0.009590157	0.007373787	0.004322091	0.003525606
Eubacterium	0	0	0	0	0	0.000108568	0	5.07E-05	0
Ezakiella	4.91E-05	0	0	0	0	0	0	0	0
Fabibacter	0	0	0	0.000109902	0	0	0.004856529	0	0
Faecalibact	0.01096257	0.006314511	0.040159868	0.021430926	0.079302939	0.009535873	0.019693098	0.030964422	0.033281237
Faecalibacu	0	0	0	0	0	0	0	0	0
Faecalicocc	0	0	0	0	0	0	0	0	0
Faecalitalea	4.91E-05	0.000229373	0	0.000392508	0.000134994	0.00074188	0.000266982	0.000608388	0.000205963
Family XIII /	6.14E-05	0	6.43E-05	0.000298306	0	0	0	0.000139422	0
Family XIII U	0.000147313	0	6.43E-05	0.000172703	0.000564521	0.000108568	0.000266982	0.000126748	0
Flavonifract	0.000638358	0.000944478	0.000706814	0.000329707	0.000883598	0.000325703	0.000228842	0.00024082	0.000496735
Formosa	0	0	0	0.000219804	0	0	0.000139848	0	0
Fournierella	0	0	0	0	8.59E-05	0	0	0	0
Fusicatenib	0.002234253	0.001065911	0.001516437	0.003705274	0.001926735	0.003076088	0.003572473	0.007909046	0.007293522
Fusobacteri	0.123730957	0.264737233	0.002595934	0.001413028	0.003608026	0.00519316	0.006178725	0.000912582	0.032542192
GCA-90006	0	0	6.43E-05	0	0.000208627	0	0	0	0
GCA-90006	0	0	0	0.000612312	0.00072406	0	0.000203415	0.000114073	0
Gemella	8.59E-05	6.75E-05	5.14E-05	0.000141303	0	0.000416177	0.000114421	0.000177447	0.000193848
Gimesia	0	0	0	0.000109902	0	0	0.000610244	0.00053234	0
Gluconobac	0	0	0	0	0	0	0	0	0
Gordonibac	0	0	0	0	0	0	0	0	0
Gottschalkia	0	0	0	0	0	0	0	0	0
Granulicatel	0	8.10E-05	0	0	0	0.000343798	0	0.000139422	0.00024231
Haemophilu	0.001129403	0.000215881	0.000321279	0.000800716	0.000441799	0.000560934	0.001182349	0.003624979	0.011291632
Harryflintia	0	0	0	0	0	0	0	0	0
hgcI clade	0	0	0	0	0	0	2.54E-05	0	0
Hoeflea	0	0	0	0	0	0	0	0	0
Holdemanel	0.002246529	0	0.00011566	0.00050241	0.059630607	0.014747127	0.000661098	0.000671762	0.000230194
Holdemania	0.000122761	5.40E-05	0.000321279	0.000172703	0.000233172	0.00023523	0.000114421	0	9.69E-05
Howardella	0	0	0	0	0	0	5.09E-05	0	0
Hungatella	0.000159589	0.000485732	0.000205619	0.000219804	0.001043137	0	0.000203415	8.87E-05	0.000315003
Hydrogenoa	4.91E-05	0	0	0	0.000245444	0	0	0.000114073	0
Ileibacteriun	0	0	0	0.000125602	0	0.000253325	0.000228842	0.00024082	0.000169617
Intestinibact	0.000638358	0.000404776	0.0005526	0.00102052	0.001067681	0.001103773	0.003445339	0.00231948	0.000860199
Intestinimor	9.82E-05	0.00010794	0.000436939	9.42E-05	0.000478616	0.000217136	0.000165275	0.000228146	0
Jeotgalibaca	0	0	0	0	0	0	0	0	0
Kordiimonas	0	0	0	0.000235505	0	0	0.000228842	0.000126748	0
Lachnoclost	0.020709805	0.014167173	0.022669443	0.013015559	0.011094066	0.183895775	0.004627687	0.01282685	0.008456608
Lachnoclost	0.000466492	0.000242866	0.000244172	0	0	0	0.000648385	0	0
Lachnoclost	0	0	0	0	0	0	0	0	0
Lachnospira	0.017260217	0.01404574	0.006528388	0.069740788	0.020617292	0.016719443	0.070216255	0.055173201	0.075794473
Lachnospira	0.000245522	0	0.000179916	0.000392508	0.00100632	0.000199041	0.000279695	0.001254801	0.000823853
Lachnospira	0.00060153	0.000202388	0.000192767	0.001868337	0.003546665	0.000416177	0.003839455	0.001470271	0.002047517
Lachnospira	0.005106864	0.003885853	0.001901971	0.011272824	0.007559674	0.004541753	0.009738485	0.023106075	0.005318698
Lachnospira	0.002332462	0.001578628	0.000758218	0.003359867	0.010320918	0.001918031	0.009840192	0.00744008	0.002835023
Lachnospira	0.000368283	0.000256358	0.000308428	0.00100482	0	0.000379987	0.002809667	0.000709786	0.000157501
Lachnospira	0.002209701	0.00456048	0.003135683	0.003061561	0.007633307	0.00148376	0.001563751	0.002040635	0.004555422
Lachnospira	0.000196418	0	0	0	0	0	0.000152561	0	0
Lachnospira	0.002946267	0.000701612	0.006759709	0.000596612	0.003166227	0.002605627	0.00129677	0.000861883	0.00150232
Lacticigeniu	0	0	0	0	0	0	0	0	0
Lactobacillu	0.000982089	0.000161911	0.000424088	0.000423908	0.000404983	0.000705691	0.000381403	0.000494315	0.000218079
Lactococcus	0	0	0.000102809	0.000266905	0	0.001465665	0.000317836	0.000468966	0.000327118
Lentibacter	0	0	0	9.42E-05	0	0	0.000470397	0.000139422	0
Leuconosto	0	0	0.000231321	0	0	0	6.36E-05	0	0
Leucothrix	0	0	0	0	0	0	0.000343263	0.000126748	0
Litoricola	0	0	0	0.000141303	0	0	0.000190701	0.000583039	0
Mailhella	4.91E-05	5.40E-05	0	4.71E-05	0	0	0	0	0
Mannheimia	0	0	0	0	0	0	0	0	0

Maribacter	0	0	0	0	0	0	0	7.60E-05	0
Marinicella	0	0	0	0.000188404	0	0	8.90E-05	0	0
Marinobacte	0	0	0	0	0	0	0	0	0
Marivita	0	0	0	0.000471009	0	0	0.000775519	0.002256106	0
Marvinbryar	0.000196418	0	0.000167065	7.85E-05	0	0	0.000190701	0	0
Massilia	0	0	0	0	0	0	0	0	0
Megamonas	0.033035024	0.001942927	0.003084278	0.000533811	0.007166963	0.000633312	0.000533964	0.006324702	0.00600928
Megasphae	0.010446973	0.001767523	0.003264194	0.00051811	0.004430263	0.00050665	0.00226299	0.00077316	0.000230194
Merdibacter	0	0	0	0	0	0	0	0	0
Methanobre	0	0	0	0	0	0	0	0	0
Methylobact	0	0	0	0	0	0	0	0	0
Methylotene	0	0	0	0	0	0	0	0	0
Mf105b01	0	0	0	0.000109902	0	0	0	0	0
Micrococcus	0	0	0	0	0	0	0	0	0
Mitsuokella	0.002909439	0.001821494	0.002403167	0	0.00011045	0.000144757	0.00185616	3.80E-05	0
Mogibacteri	4.91E-05	0	0	0	0.00011045	0	0	0	2.42E-05
Morganella	0	0	0	0	0	0	0	0	0
Moryella	0.000122761	0	0.000629707	0.000690814	0	0	0.000419543	0.000291519	0
Murimonas	0	0	0	0	0	0	0	0	0
Mycobacter	0	0	0	0	0	0	0	0	0
Negativibac	0.000331455	0	0.000269874	0	0.001165859	0	0.000139848	0	0
Nitrosomon	0	0	0	0.001774135	0	0	0.00278424	0	0
Nosocomiic	0	0	0	0	0	0	0	0	0
NS3a marin	0	0	0	4.71E-05	0	0	0.001716313	0.002002611	0
Oblitimonas	0	0	0	0	0	0	0	0	0
Oceanicocc	0	0	0	0	0	0	0	5.07E-05	0
Oceanobact	0	0	0	0	0	0	0	0	0
Oceanospiri	0	0	0	0	0	0	0.000190701	0.000139422	0
Odoribacter	0.000527873	0.000755583	0.001824864	0.002417848	0.000871326	0.001936126	0.002809667	0.003371484	0.00024231
Olsenella	0	0	0	0	0	0	0	0	0
OM27 clade	0	0	0	0	0	0	0	0.000101398	0
Oribacteriur	0	0	0	0	0	0	0	0	0
Oscillibacter	0.000282351	0.000175403	0.001066646	0.000439609	0.001484936	0.000633312	0.000203415	0.000202796	0.000545197
Oscillospira	0	0	0	0	0	0	0	0	0
Ostreobium	0	0	0	0	0	0	0	0	0
Oxalobacter	0.000159589	0	9.00E-05	9.42E-05	4.91E-05	0	0.000597531	0.000114073	8.48E-05
Paenalcalig	0	0	0	3.14E-05	0	0	0	0	0
Paeniclostri	0.000159589	0	0	0	0.000196355	0	0	0.000202796	0
Parabacter	0.009734959	0.006274034	0.034029866	0.012199143	0.011192244	0.007708314	0.012942268	0.010950987	0.006154666
Paraclostrid	0	0	0	0	0	0	0	0	0
Paracoccus	0	0	0	0	0	0	0	0	0
Parahaliea	0	0	0	0	0	0	0.000127134	0	0
Paralcaliger	0	0	0	0	0	0	0	0	0
Paraprevote	0.00837231	0.001200837	0.01232426	0.010142402	0.001681291	0.002696101	0.006928818	0.003181363	0.001950593
Parasuttere	0.004210707	0.003481077	0.009072917	0.010958818	0.012505369	0.002895142	0.003140216	0.002104009	0.000799622
Patulibacter	0	0	0	0	0	0	0	0	0
Pediococcu	0	0	0	0	0	0	0	0	0
Pedobacter	0	0	0	0	0	0	0	0	0
Peptoclostri	0	0	0	0	0	0	0	0	0
Peptococcu	0.000282351	0	2.57E-05	0.000172703	0.000466343	7.24E-05	6.36E-05	0.00024082	0
Phascolarct	0.012153353	0.003130271	0.013326651	0.003077261	0.006860158	0.003564643	0.004691254	0.002750421	0.001405396
Phocea	0	0	0	9.42E-05	0.000184083	0	0	0	0
Planctomicr	0	0	0	0.000408208	0	0	0.000368689	0.00024082	0
Polynucleob	0	0	0	0	0	0	0	0	0
Porticoccus	0	0	0	0	0	0	0	0.000608388	0
Prevotella	0.000122761	0	0	0	0	0	0	0	0
Prevotella 2	0	0	0	0.001648533	0.001092226	0.001266624	0.001970581	0.001178752	0.034565478
Prevotella 6	0.000343731	0	0	0	0	0	0	0	0
Prevotella 7	0.000368283	0	0	0	0.008455544	0	0	0	0
Prevotella 9	0.310340171	0.002631046	0.002595934	0.007347746	0.014137571	0.004632227	0.042068729	0.004626285	0.307999855
Prevotellace	0	0	0	0	0	0	0	0	0
Prevotellace	0.001006641	5.40E-05	0.00088673	0.000188404	0.05574032	9.05E-05	0.003903022	0.000114073	0.000145386
Prevotellace	0	0	0	0	0	0	0	0	0
Prevotellace	0	0	0	0	3.68E-05	0	0	0	0
Proteocatell	0	0	0	0	0	0	0	0	0
Proteus	0	0.000148418	0	0	0	0	0	0	0
Providencia	0	0	0	0	0	0	0	0	0
Pseudoalter	0	0	0	0.000157003	0	0	0.000368689	0.000823859	0
Pseudochro	0	0	0	0	0	0	0	0	0
Pseudoflavo	0	0	0	0	0	0	0	0	0
Pseudohong	0	0	0	4.71E-05	0	0	0.001106068	0.00026617	0
Pseudomon	0	0	0	0	0	0	0	0	0
Pseudophae	0	0	0	0	0	0	0.000330549	0.000811184	0
Pseudovibri	0	0	0	0	0	0	0	0	0
Pyramidoba	0	0	0	0	0.00050316	0.000126662	2.54E-05	6.34E-05	0
Raoultibacte	0	0	0	0	0	0	0	0	0
Reichenbac	0	0	0	0.000157003	0	0	0.000165275	0.000152097	0
Rheinheime	0	0	0	0.000188404	0	0	0.000317836	0.000202796	0
Rhodococcu	0	0	0	0	0	0	0	0	0
Rhodoferax	0	0	0	0	0	0	0	0	0
Rikenellacea	0	0	0	0	0.000147266	0	0	0	0

Romboutsia	0.002798954	0.000620657	0.001092348	0.001915438	0.003497576	0.005012214	0.002733387	0.004740358	0.005561008
Roseburia	0.009366675	0.003926331	0.002416018	0.023267863	0.021869056	0.009734914	0.024422493	0.021318935	0.016137826
Roseibacillu	0	0	0	0.000471009	0	0	0.000368689	0	0
Rothia	0	0	0	0	0	0	6.36E-05	0	0
Ruegeria	0	0	0	0	0	0	0.000355976	0.002509601	0
Ruminiclost	0	0	0	0	0	0	0	0	0
Ruminiclost	0	0	0	0	0	0	0	0	0
Ruminiclost	0.000257798	0.000539702	0.000822474	0.000973419	0.00100632	0.004704605	0.000228842	0.000456291	0.000593659
Ruminiclost	0.000196418	0	0.000539749	0.001946839	0.000441799	0.001302814	0.003623327	0.005551542	0.000702698
Ruminiclost	0.000687462	0.000134925	0.000321279	0.000596612	0.00312941	0.000488555	0.000152561	7.60E-05	0.000351349
Ruminococc	0.00429664	0.000647642	0.009972499	0.003611072	0.044106277	0.005102687	0.008505282	0.010874938	0.002192903
Ruminococc	0.011011675	0.002037374	0.022566633	0.005212504	0.031711358	0.008576857	0.007615343	0.0064261	0.002071748
Ruminococc	0.001276716	0.000202388	0.001747757	0.000926318	0.002663067	0.000796164	0.001385764	0.000950606	0.000593659
Ruminococc	0.000356007	8.10E-05	0.000167065	0.000266905	0.001079953	0.00027142	0.000190701	0.000354893	8.48E-05
Ruminococc	0.002909439	0.00063415	0.003264194	0.002637652	0.009682764	0.001936126	0.004716681	0.002458902	0.000957123
Ruminococc	0	0	0	0	0.000196355	0	0	0	0
Ruminococc	0.000687462	8.10E-05	0.000526897	0.000109902	0.002098546	0.000108568	0.000203415	0.000152097	0
Ruminococc	0.002185148	0.001241314	0.000424088	0.00202534	0.005019329	0.003564643	0.00816202	0.003561606	0.002568483
Ruminococc	0.001865969	0	0.001349372	0.010864616	0.007522857	0.002388492	0.004004729	0.00925257	0.005694278
Ruminococc	0.022403909	0.002981853	0.004523608	0.017254643	0.010136835	0.004306523	0.021320416	0.013612685	0.003961764
Ruminococc	0.004799961	0.00137624	0.004009561	0.011005919	0.026152053	0.007165475	0.004347992	0.013295816	0.002762331
Sanguibacte	0	0	0	0	0	0	0	0	0
Sarcina	0	0	0	0.000235505	0	0.000253325	0.000279695	0.001926562	0.000145386
Sediminibac	0	0	0	0	0	0	0	0	0
Selenomona	0.000392836	0.000242866	0.000231321	0	0	0	0	0	0
Sellimonas	0	0	0	0	0	0	0	0	0
Senegalima	0.000294627	0	0.000257023	0	0.000490888	9.05E-05	0	0	0
Serratia	0	0	0	0	0	0	0	0	0
Shuttleworth	0	0	0	0	0	0	0	0	0
Slackia	0	0	0	0	3.68E-05	0	0	0	0
Sneathiella	0	0	0	0.000235505	0	0	0	0	0
Solobacteriu	0.000135037	0	6.43E-05	0.000172703	0.00011045	0.000162852	7.63E-05	3.80E-05	7.27E-05
Sphingobac	0	0	0	0	0	0	0	0	0
Sphingomon	0	0	0	9.42E-05	0	0	6.36E-05	0	0
Staphylococ	0	0	0	0	0	0	0	0	0
Stenotropho	0	0	0	4.71E-05	0	0	0	0	0
Streptococc	0.00660455	0.013978277	0.002171846	0.00651563	0.004982512	0.009933955	0.003483479	0.006286678	0.007875065
Subdoligran	0.006113505	0.001915941	0.040262678	0.007127942	0.018469657	0.009047317	0.012573579	0.036021648	0.008916997
Succinatimo	0	0	0	0	0	0	0	0	0
Succinivibri	0	0	0	0	0	0	0	0	0
Sulfitobacte	0	0	0	0.002056741	0	0	0.000292409	0.000633738	0
Sutterella	0.029438122	0.003885853	0.017606086	0.003438368	0.004908879	0.002623722	0.004691254	0.007326007	0.043688438
Synergistes	0	0	0	0	0	0	6.36E-05	0	0
Taeseokella	0	0	0	0.000314006	0	0	0.000635671	0.000253495	0
Tepidimicro	0	0	0	0	0	0	0	0	0
Terrisporob	0.000319179	0.000215881	0	0.000266905	0.000306805	0.000144757	0.000495824	0.000342218	0.000302887
Thalassotal	0	0	0	0	0	0	0	0.000164772	0
Thiopseudo	0	0	0	0	0	0	0	0	0
Tissierella	0	0	0	0	0	0	0	2.53E-05	0
Turicibacter	0.000184142	0	0	0.000329707	0.000441799	0.000398082	0	0.001140728	0.000545197
Tyzzerella	0	0.001079404	0	0	0.000196355	0.000687596	0	0	0
Tyzzerella 3	0.000491045	0.000175403	0.000102809	0.019264283	9.82E-05	0.002913236	0.00133491	0.001951912	0.000908661
Tyzzerella 4	0.001129403	0.000499224	0.000411237	0.000659413	0.000429527	0.001085678	0.000508537	0.000697111	0.00037558
UBA1819	0.000233246	0.000161911	0.000758218	0.000282606	0.00072406	0.000307609	0.000165275	0.000253495	8.48E-05
uncultured	0.006408132	0.042110234	0.006001491	0.020363305	0.006676075	0.288120872	0.015039984	0.01746581	0.006021396
uncultured b	0.000343731	5.40E-05	0.001734906	0.000816416	0.004295269	0	0.000343263	0.000139422	4.85E-05
uncultured b	0	0	0	0.000188404	0.0002209	0.000217136	0	0.000164772	9.69E-05
uncultured E	0	0	0	0	0	0	0	0	0
uncultured E	0	0	0	0.000172703	0	0	2.54E-05	0.000101398	0
uncultured C	0	0	0	0.000141303	0	0	2.54E-05	3.80E-05	0
uncultured C	0	0	0	0	0	0.000199041	0	0	0
uncultured g	0	0	0	9.42E-05	0	0	0	0	0
uncultured r	0	0	0	0.000266905	0	0	0.000165275	0.000114073	0
uncultured o	0.000589253	0.000175403	0.000950986	0.000565211	0.002221268	0.000307609	0.001029788	0.000481641	7.27E-05
uncultured F	0.000871604	0	0	0	0.014567098	0	0	7.60E-05	0
uncultured p	0	0	0	0.000172703	0	0	0.000127134	0	0
uncultured r	0	0	0	0	6.14E-05	0	0	7.60E-05	0
uncultured S	0	0	0	0	0	0	0	0	0
uncultured T	0	0	9.00E-05	0	0	0	0	0	0
uncultured V	0	0	0	0.000345407	0	0	0.001741739	0.000430942	0
unidentified	0	0	0	0	0	0	0	0	0
Veillonella	0.001350373	0.023450044	0.001118051	0.001365927	0.000466343	0.000615218	0.001233202	0.003434858	0.00163559
Vibrio	0	0	0	0	0	0	0.000177988	0	0
Victivallis	0.000135037	0	0.00011566	0.000329707	0.000920415	0.000452366	0.000394116	0.000354893	0.000121155
W5053	0	0	0	0	0	0	0	0	0
Weissella	0.000110485	8.10E-05	3.86E-05	0.000298306	0.000122722	0.001230435	0.000635671	0.000291519	0.000254425
Wenyingzhu	0	0	0	0	0	0	0	7.60E-05	0
Woeseia	0	0	0	0	0	0	0	0	0
Genus	W69	W71	W74	W76	W77	W79	W80	W8	W16
[Caedibacte	0	0	0	6.61E-05	0	0	0	0	0

[Clostridium	6.79E-05	0.000264938	0	0	0	0	0	0.000191713	8.18E-05
[Eubacteriu	0	0	0	0	0	0	0	0	0
[Eubacteriu	0.005606842	0.006762881	0.007102887	0.004876695	0.005363789	0.002784223	0.000744916	0.003765782	0.018534177
[Eubacteriu	0.003312517	0.002691208	0.029930948	0.007017683	0.007430051	0.014676831	0.000646531	0.009407608	0.007241831
[Eubacteriu	0.00290524	0.001519905	0.001111756	0.001784157	0.000693471	0.000941332	0.000716806	0.000958563	0.001963886
[Eubacteriu	0	9.76E-05	6.18E-05	0	0	0	5.62E-05	0.000232794	0.000122743
[Eubacteriu	0.000936736	0.001017918	0.004002322	0.00088547	0.002915411	0.000464037	0.00036543	0.000794238	0.002318477
[Eubacteriu	0.00297312	0.002495991	0.00381703	0.003026458	0.002405921	0.009598939	0.002276912	0.000999644	0.001991163
[Eubacteriu	0.000936736	0.000808757	0.001062345	0.001189438	0.000481184	0.000304939	8.43E-05	0	0.002659429
[Polaribacte	0.001344013	0.000320714	0.000457055	0.002154204	0.001033131	0	0.00019677	0	0
[Ruminococ	0	0.00030677	0.000197646	7.93E-05	0.000622709	0	0.00047787	0	0
[Ruminococ	0.00396416	0.027372237	0.003236446	0.043705231	0.005646839	0.00324826	0.001433611	0.001328294	0.000313676
[Ruminococ	0.004846592	0.002440215	0.004051734	0.010123437	0.00348151	0.009095128	0.009979058	0.007065977	0.005018821
Abiotrophia	0	0	0	0	0	0	0	0	0
Acetanaerol	0	0	0	0	0	0	0	0	0
Acetitomacu	0	0	0	0	0	0	0	0	0
Acetobacter	0	0.00030677	0	0	0	0	0	0	0
Acidaminoc	0	0	0	0	0	0	0	0.001109194	0.001063772
Acinetobact	0	0.002426271	0	2.64E-05	0	0	7.03E-05	0	0
Actinobacillu	0	0	0	0	0.001047283	0	0	0	0
Actinomyce	0.000176487	0.000195217	0.000111176	0.000171808	0	0.000278422	0.00039354	0.000273875	0
Adlercreutzi	0.00029867	0	0.000247057	0.000105728	0	0	0	0	0.000136381
Aeromonas	0	0	9.88E-05	0	0	0.001246271	0	0	0
Aerosphaer	0	0	0	0	0	0	0	0	0
Aestuariicel	5.43E-05	0	0	0	0	0	0	0	0
Agathobact	0.018449633	0.006665272	0.016985164	0.007374514	0.013175958	0.007132913	0.002305022	0.013187084	0.003327696
Aggregatiba	0	0	0	0	0.000297202	0.000570103	0	0	0
Akkermansi	9.50E-05	0.001129471	0.002853508	0.000105728	0.001471858	2.65E-05	0	0.000575138	0.001813867
Alcaligenes	0	0	0	0	0	0	0	0	0
Alcanivorax	0	0.000139441	0	0	0	0	0	0	0
Alistipes	0.032717893	0.007585582	0.069744173	0.012964872	0.02026635	0.005183958	0.051806772	0.020239367	0.035063554
Allisonella	0	0	0	0.000475775	5.66E-05	0	4.22E-05	0.000314956	0.000531886
Allobaculum	0	0.000250994	0	0	0	0	0	0	0
Alloprevotel	0.000475156	0	0.000419997	0.000502207	0.016133826	0.000278422	0	0.000575138	4.09E-05
Allorhizobiu	0	0	0	0	0	0	0	0	0
Alloscardov	0	0	0	0	0	0	0	0	0
Amphritea	0	0	0.000148234	0.000211456	0.000537794	0	0	0	0
Anaerofilum	0	0	3.71E-05	0	0	0	0	0	0
Anaeroglob	0	0	0	0	0	0	0	0	6.82E-05
Anaerospor	0	0.000237049	0.000679407	0.000211456	0	0	0	0.000205406	0.000422781
Anaerostipe	0.00297312	0.003053754	0.000642348	0.006356884	0.000622709	0.000503812	0.001433611	0.000561444	0.001718401
Anaerotrunc	0.000135759	0.000125497	0.000247057	0.000158592	2.83E-05	0.000198873	0	0.000164325	0.000163657
Angelakisell	0	0	0	0	0	0	0	0	0.000136381
Anoxybacillu	0	0	0	0	0	0	0	0	0
Anseongella	0	8.37E-05	0	0	0	0	0	0	0
Arenibacter	0	0	0.000111176	0	7.08E-05	0	0	0	0
Atopobium	0	0.000111553	0	0	0.000127372	7.95E-05	8.43E-05	0.000246488	0
Atopostipes	0	0	0	0	0	0	0	0.0002191	6.82E-05
Aureimarina	0	0	0.000308821	0	0	0	0	0	0
Azospirillum	0	0	0	0	0	0	4.22E-05	0	0.000272762
Bacillus	0	0.000181273	4.94E-05	0	0	0	0	0	0
Bacteroides	0.583179473	0.32249878	0.252776302	0.345544895	0.174400996	0.447278754	0.641962642	0.418700189	0.49038514
Balneola	0.000285094	0.000892421	0.00043235	0	0.000764234	0	0	0	0
Barnesiella	0.006625034	0.000962142	0.001964103	0.00121587	0.002207787	0.000357971	0.001574161	0.002410101	0.015670176
Bifidobacter	0.000855281	0.014766785	0.000382938	0.001519837	0.000495337	0.001657275	0.000407595	0.000670994	0.001827505
Bilophila	0.00214499	0.000529875	0.000407644	0.000607935	0.000452879	0.000238648	0.009416858	0.002259469	0.00330042
Blautia	0.02275319	0.04066095	0.014292244	0.033978273	0.013841124	0.035624793	0.013787966	0.007641114	0.007637336
Bosea	0	6.97E-05	0	0	0	0	0	0	0
Brevibacillus	0	0	0	0	0	0	0	0	0
Brevundimo	0	9.76E-05	0	0	0	0	0	0	0
Butyricicoc	0.003298941	0.001380464	0.002322335	0.008881136	0.001401095	0.005621478	0.000983851	0.002656588	0.00272762
Butyricimon	0.000366549	0	0.000185293	0.000502207	0.001018978	0	0.00019677	0.001383069	0.002195734
Butyrivibrio	0	0	0.000716465	0	0	0	0	0	0
C1-B045	0	0	0	0	0.00016983	0	0	0	0
CAG-352	0	0.000111553	0.000778229	0	0	0	7.03E-05	0	0
CAG-56	0.002728754	0.000780869	0.003347621	0.001255518	0.001089741	0.015167385	0.000295155	0.000383425	0.000613714
CAG-873	0	0	0	0	0	0	0	0	0
Campylobac	0	0	0	0	0	0	0	0	0
Candidatus	0	9.76E-05	8.65E-05	0.000555071	0.000268897	0	0	0	0
Candidatus	9.50E-05	0.000125497	2.47E-05	9.25E-05	0	0	0	0	0
Candidatus	8.15E-05	0	6.18E-05	0	2.83E-05	0	0	0	0
Candidatus	0	0	0	0	0	0	0	0	0
Candidatus	0	0	0	0	0	0	0	0	0
Candidatus	0	9.76E-05	0	3.96E-05	0	9.28E-05	0	0.00010955	0.000109105
Candidatus	0	0	0	0	0	9.28E-05	0	0	0
Caproicipro	0	0	0	0	0	0	0	0.000164325	4.09E-05
Catabacter	0	0	0	0	0	0	0	0	0
Catenibacte	5.43E-05	8.37E-05	0	5.29E-05	0.000934064	0	0	0	0.000136381
Cellulosilytic	0.00029867	0.000334658	0.000494114	0.000911902	0.003000326	0.000198873	0	0	0
Cellvibrio	0	0	0	0	0	0	0	0	0
Cephalotico	8.15E-05	0.000153385	0	0	0	0	0	0	0

Christensen	0	0	0	0	0	0	0	0	0
Christensen	0.004385012	0.00334658	0.004088792	0.003938361	0.004925063	0.001498177	0.002445572	0.003341276	0.008060117
Chryseoline	0	0.000111553	0	0	4.25E-05	0	4.22E-05	0	0
Chthoniobac	0	0	0	0	0	0	0	0	0
Clavibacter	0	0	0	0	0	0	0	0	0
Cloacibacillu	0	9.76E-05	0	0	0	0	0	0	0
Clostridiales	0	0	0	0	0	0	0	0	0
Clostridioide	0	0	0	0	0	0	0	0	0
Clostridium	0.006815096	0.04994771	0.011537559	0.083472101	0.019318134	0.010142526	0.000491925	0.003190644	0.004255087
Collinsella	0.000407277	0.001938228	0.000691759	0.001599133	0.000594404	0.003752072	0.004033788	0.000451894	0.002100267
Colwellia	4.07E-05	0	0	0	0.000141525	0	0	0	0
Comamona	0	0	0	0	0	0	0	5.48E-05	0.00021821
Coprobacillu	0	0	0	0	0	0	0	4.11E-05	0.000136381
Coprobacte	0.001995656	0.000250994	0.000333527	0.000488991	0.000806691	0.000212131	0.001082236	0.000479281	0.000381867
Coprococcu	0.000488732	0.000264938	0.000271763	0.000396479	0.000452879	6.63E-05	0.000126495	0.0004382	0.000381867
Coprococcu	0.001493348	0.000822701	0.007337591	0.001202654	0.000636862	0.000278422	0	0.00054775	0.000763734
Coprococcu	0.003231062	0.00105975	0.002445864	0.002762139	0.002306854	0.000530328	0	0.007682195	0.000204571
Coriobacter	0	0	0	0	0	0	0	0	0
Coxiella	8.15E-05	5.58E-05	0	0	0	0	0	0	0
Crocinitomix	0.000162911	0	0.000407644	0	0	0	0	0	0
Cyclobacter	0.00029867	0	0.000506467	0	0.000311355	0	0.00014055	0	0
Defluviitalea	5.43E-05	0	0	0	0	0	0	0	0
Deinococcu	0	0	0	0	0	0	0	0	0
Delftia	0	0	0	0	0	0	0	0	0
Desulfovibri	0.000448004	0.000864533	0.000654701	0.000634367	0.001103893	0.00014584	0	0.001122888	0.001609296
Dialister	0.001099647	0.001631458	0.003162329	0.005590358	0.01153427	0.000875041	0	0.001821269	0.005277944
Dielma	0	5.58E-05	4.94E-05	9.25E-05	0.000183982	0	0	0	0
Dolosicoccu	0	0	0	0	0	0	0	0	0
Donghicola	0.000149335	0.000278882	0.006584067	0.00522031	0.00530718	0	0	0	0
Dorea	0.004900896	0.002217109	0.004854669	0.015356963	0.003042783	0.008432217	0.001082236	0.002122532	0.003450439
DTU089	0	0	0	0	0	0	0	0	0
Dubosiella	0	0.000529875	0	0	0	0	0	0	0
Dysgonomo	0	0	0	0	0	0	0	0	0
Eggerthella	8.15E-05	0.000348602	8.65E-05	0	5.66E-05	0	0	0.000479281	0.000150019
Eisenbergie	0.000135759	0	6.18E-05	0.000145376	0.000297202	7.95E-05	0	0	0.000381867
Enhydrobac	0	0	0	0	0	0	0	0	0
Enterococcu	0	0.000362546	0.000370585	0	0.00022644	0	0	0	0
Enterorhabd	0	0	0	0	0	0	0	0	0
Epulopisciu	0	8.37E-05	0	0.00013216	0	0.000212131	0	0	0
Erysipelatoc	0.001941352	0.05404727	0.00134646	0.003000026	0.002023804	0.003937686	4.22E-05	0.000383425	0.000450057
Erysipelotric	0.005769753	0.004015896	0.007782293	0.027555309	0.002023804	0.023228373	0.001489831	0.002505957	0.00079101
Erysipelotric	0	0	0	0	0	0	0	0	0
Escherichia-	0.005824056	0.004531827	0.002927625	0.002471387	0.002830496	0.001484919	0.0005622	0.03393312	0.025980579
Eubacteriun	0	0	0	0	0	0	0	0	0
Ezakiella	0	0	0	0	0	0	0	0	0
Fabibacter	8.15E-05	5.58E-05	0.004842316	0	0	0	0	0	0
Faecalibact	0.027776269	0.089465244	0.019653379	0.025718288	0.01352977	0.015220418	0.014645322	0.022895955	0.04039605
Faecalibacu	0	0.000139441	0	0	0	0	0	0	0
Faecalicocc	0	0	0	0	0	0	0	0	0
Faecalitalea	0.000244366	0.001185247	0	0.000449343	0	0.000357971	9.84E-05	0.000479281	0
Family XIII /	0	0.000278882	0	0	0.00011322	3.98E-05	0	0	0.000300038
Family XIII U	0.000705946	0.000167329	9.88E-05	0.000105728	7.08E-05	0	0	0	0.000177295
Flavonifract	0.00029867	0.000571707	0.000197646	0.000462559	0.000396269	0.00080875	0.00039354	0.000342344	0.000177295
Formosa	0.00046158	0	8.65E-05	5.29E-05	0	0	0.000520035	0	0
Fournierella	0	4.18E-05	0	0	0	0	0	0	0
Fusicatenib	0.005837632	0.00372307	0.003359974	0.016652129	0.00308524	0.007782565	0.005101969	0.000999644	0.003191315
Fusobacteri	0.000977464	0.001952172	0.042345559	0.000502207	0.00159923	0.004070268	0.00042165	0.156807165	0.001022857
GCA-90006	0	0	0	0	0	0	0	0	0.000231848
GCA-90006	0.000352973	0	0.00017294	0	0.000141525	0.000198873	0	0	8.18E-05
Gemella	0.000122183	0.000125497	8.65E-05	0.000277535	7.08E-05	0.000238648	0.000182715	0	8.18E-05
Gimesia	6.79E-05	0.001045806	0.00034588	0.000449343	0.00016983	0	0	0	0
Gluconobac	0	0	0	0	0	0	0	0	0
Gordonibact	0	0	0	0	0	0	0	0	0
Gottschalkia	0	0	0	0	0	0	0	0	0
Granulicatel	6.79E-05	0	0	0.000555071	0	0.000251906	0	0.000260181	0
Haemophilu	0.001276134	0.001464129	0.002334688	0.009132239	0.028446482	0.014888963	0.00025299	0.001876044	0.000818286
Harryflintia	0	0	0	0	0	0	0	0	0
hgcI clade	0	0	0	7.93E-05	0	0	0	0	0
Hoeflea	0	0	0	0	0	0	0	0	0
Holdemanel	0.001493348	0.00266332	0.00143293	0.0019031	0.024200739	0.001166722	0	0.00521732	0.001527467
Holdemania	8.15E-05	0.000153385	8.65E-05	7.93E-05	0.00022644	0.000411004	0.00014055	0.000232794	0
Howardella	0	0	0	0	0	0	0	0	0.000122743
Hungatella	0.000122183	0.000292826	9.88E-05	0.000171808	0.000410422	0.000702685	0	0.000314956	0.000736457
Hydrogenoa	0	0	4.94E-05	0	0	0	0	6.85E-05	0
Ileibacteriun	0	0.001115527	0	0	0	0	0	0	0
Intestinibact	0.00046158	0.000669316	0.00125999	0.001466973	0.001344485	0.000503812	0.00019677	0.000588831	0.001131962
Intestinimor	0	0.000125497	0.000160587	0	8.49E-05	0	0	0	0
Jeotgalibaca	0	0	0	0	0	0	0	0	0
Kordiimonas	0.000122183	0.000292826	8.65E-05	0.00013216	0.000495337	0	0	0	0
Lachnoclost	0.011145805	0.026981803	0.004854669	0.033568578	0.004104219	0.009598939	0.013352261	0.010078602	0.00308221
Lachnoclost	0	0.000237049	0	0	0	0	0	0.000314956	0

Lachnoclost	0	0	0	0	0	0	0	0	0
Lachnospira	0.043918002	0.147221641	0.042123207	0.088943515	0.030102322	0.083646006	0.014125286	0.052789417	0.023334788
Lachnospira	0.000176487	0	0.000123528	0.000118944	0.000891606	0.000172357	4.22E-05	9.59E-05	0.000272762
Lachnospira	0.000882433	0.001087639	0.00117352	0.00081939	0.000636862	0.001272788	0.000182715	0.001328294	0.000722819
Lachnospira	0.007738257	0.002691208	0.017145751	0.007903153	0.008816994	0.015353	0.001349281	0.014063484	0.011728765
Lachnospira	0.00267445	0.001784843	0.006547009	0.001770941	0.000495337	0.002055022	0	0.001711719	0.001131962
Lachnospira	0.000257942	0.00030677	0.006386422	0.000621151	0.000212287	0.000503812	0	0.000903788	0.000122743
Lachnospira	0.001846321	0.012730949	0.00169234	0.004229112	0.001924737	0.009983427	0.000632476	0.002218388	0.003191315
Lachnospira	0	0	0	0	0	0	0	0	0
Lachnospira	0.003679066	0.001533849	0.004113498	0.001876669	0.001273723	0.00649652	0.00042165	0.000479281	0.001500191
Lacticigeniu	0	0	0	0	0	0	0	0	0
Lactobacillu	0	0.002621488	0.000111176	0	0.000183982	7.95E-05	0	0.002560732	0.001022857
Lactococcus	0	0	0	0	0	0	0	0	0
Lentibacter	0	0	0.000370585	0.000290751	0.000155677	0	0	0	0
Leuconosto	6.79E-05	0.000125497	0.000148234	0.000224672	0.002986173	2.65E-05	0	0	0
Leucothrix	0	0	0.000296468	0.000105728	0	0	0	0	0
Litoricola	0	0	9.88E-05	0.000383263	0.000594404	0	0	0	0
Mailhella	0	0	0	0	5.66E-05	0	0	0.000273875	0
Mannheimia	0	0	0	0	0	0	0	0	0
Maribacter	0	0	0	9.25E-05	0	0	0	0	0
Marinicella	9.50E-05	0	0	0	0	0	7.03E-05	0	0
Marinobacte	0	0	0	6.61E-05	0.000183982	0	0	0	0
Marivita	0.000502308	0.001784843	0.000654701	0.001665213	0.002137024	0	0	0	0
Marvinbryar	0	0	0	0	0	0	0	0	0.000245486
Massilia	0	0	0	0	0	0	0	0	0
Megamonas	0	0.000348602	0	0.001453757	0	0.00140537	0	0.004135513	0.011606023
Megasphae	0.001411892	0.021696995	0.002692921	0.002259932	0.002179482	0.001246271	0.001180621	0.004738039	0.02655338
Merdibacter	0	0	0	0	0	0	0	0	0
Methanobre	0	0	0	0	0	0	0	0	6.82E-05
Methylobact	0	0.000111553	0	0	0	0	0	0	0
Methylotene	0	0	0	0	0	0	0	0	0
Mf105b01	0	0.000125497	0	0	0	0	0	0	0
Micrococcus	0	0	0	0	0	0	0	0	0
Mitsuokella	0	0	0.000370585	0	0	0	0	0.006983814	0
Mogibacteri	5.43E-05	0	0	0	0	0	0	0.00010955	0
Morganella	0	0	0	0	0	0	0	0	0
Moryella	0.00029867	6.97E-05	0.000321174	0	9.91E-05	0	0	0	0.00021821
Murimonas	0	0	0	0	0	0	0	0	0
Mycobacter	0	0	0	0	0	0	0	0	0
Negativibac	0	0	0.000222351	5.29E-05	5.66E-05	0	0	0.000314956	0.000340952
Nitrosomona	0.001194678	0.000432267	0.001815868	0.000118944	0.001471858	0	0	0	0
Nosocomiic	0	4.18E-05	0	0	0	0	0	0	0
NS3a marin	8.15E-05	0	0.001927044	0.001810589	0.002165329	0	0	0	0
Oblitimonas	0	0	0	0	0	0	0	0	4.09E-05
Oceanicocc	0	0	0	0	2.83E-05	0	0	0	0
Oceanobact	0	0	0	0	0	0	0	0	0
Oceanospiri	0	0	0	0.000185024	0.002377616	0	0	0	0
Odoribacter	0.003787673	0.000627484	0.006781713	0.001176222	0.00137279	0.00014584	0.001784986	0.001383069	0.001800229
Olsenella	0	0	0	0	0	0	0	0	0
OM27 clade	0	0.000209161	0	3.96E-05	2.83E-05	0	0	0	0
Oribacteriur	0	0	0	0	0	0	0	0	0
Oscillibacte	0.000393701	0.00030677	0.000333527	0.000158592	0.000424574	0.00103414	0.00019677	0.000616219	0.000354591
Oscillospira	0.000122183	0	0	0	0.00011322	0	0	0	0
Ostreobium	0	0	0	0	0	0	0	0	0
Oxalobacte	0.000149335	0.000111553	8.65E-05	0	0	0	0	0	0.00021821
Paenalcalig	0	0	0	0	0	0	0	0.000232794	0
Paeniclostri	0	0	0	0.000515423	0	0	0	0	0
Parabacterc	0.012354059	0.007836575	0.016997517	0.011088203	0.029224869	0.012847199	0.034280173	0.018335935	0.031326714
Paraclostrid	0	0	0	0	0	0	0	0	0
Paracoccus	0	0	0	0	0	0	0	0	0
Parahaliea	0	0	0	0	0	0	0	0	0
Paralcaliger	0	0	0	0	0	0	0	0	0
Paraprevote	0.00366549	0.000725092	0.002248218	0.001268734	0.000877454	0.000822009	0.033141717	0.000999644	0.000368229
Parasuttere	0.013304371	0.00570313	0.006238188	0.001889884	0.002278549	0.001073914	0.06268535	0.002519651	0.003464077
Patulibacter	0	0	0	0	0	0	0	0	0
Pediococcu	0	0	0	0	0	0	0	0	0
Pedobacter	0	0	0	0	0	0	0	0	0
Peptoclostri	0	0	0	0	0	0	0	0	0
Peptococcu	8.15E-05	0	0.000358233	0	0.00028305	2.65E-05	0	0	2.73E-05
Phascolarct	0.005145262	0.003778847	0.00676936	0.003885497	0.00182567	0.002823997	0.020984132	0.003450826	0.006341716
Phocea	0.000203638	0	9.88E-05	3.96E-05	8.49E-05	0	0.00011244	0.000123244	8.18E-05
Planctomicr	0.00046158	0.000264938	0.000321174	0.000185024	0.00016983	0	0	0	0
Polynucleob	0	0	0	0	0	0	0	0	0
Porticoccus	0.000176487	0.000195217	8.65E-05	0.000409695	0.00016983	0	0	0	0
Prevotella	0	0	0	0	0	0	0	0	0
Prevotella 2	0	0	3.71E-05	0	0.000820844	0.004653629	0	0.0008764	0.001595658
Prevotella 6	0	0	0	0	0	0	0	0	0
Prevotella 7	0	0.000125497	0	0	0	0	0	0	0.008114669
Prevotella 9	0.011308716	0.006386391	0.191296184	0.008048529	0.366987928	0.058150481	0.001040071	0.003368663	0.001718401
Prevotellace	0	2.79E-05	0	0	0	0	0	0	0.000109105
Prevotellace	0	2.79E-05	0.001062345	0	0.000155677	0	0	0.004464163	0

Prevotellace	0	0	0	0	0	0	0	0	0
Prevotellace	0	0	0	0	0	0	0	0	0.000122743
Proteocatell	0	0	0	0	0	0	0	0	0
Proteus	0	0	0	0	0	0	0	0.000246488	4.09E-05
Providencia	0	0	0	0	0	0	0	0	0
Pseudoalter	0	0	8.65E-05	0.000528639	0.002547446	0	0	0	0
Pseudochro	0	0	0	0	0	0	0	0	0
Pseudoflavo	0	0	0	0	0	0	0	0	0
Pseudohon	0	0	0.000765876	0	0.000240592	0	0	0	0
Pseudomon	0	0.003639406	0	0	0.000268897	0	5.62E-05	0.00032865	0
Pseudopha	0	0	0.000222351	0.000700447	0.000962369	0	0	0	0
Pseudovibri	0	0	0	0	0	0	0	0	0
Pyramidoba	0	6.97E-05	7.41E-05	0.000158592	7.08E-05	0	0	0.000588831	0.000845562
Raoultibact	0	0	0	0	0	0	0	0	0
Reichenbac	0.000108607	0.000181273	0.000197646	0.000145376	0	0	0.000154605	0	0
Rheinheime	0.000190062	0	0.000222351	9.25E-05	0.000183982	0	0	0	0
Rhodococcu	0	0	0	0	0	0	0	0	0
Rhodoferax	0	0	0	0	0	0	0	0	0
Rikenellace	0	6.97E-05	0	0	0	0	0	0.000178019	0.001186515
Romboutsia	0.002063535	0.0025936	0.004360555	0.009026511	0.002137024	0.004255883	0.000871411	0.001027031	0.001977524
Roseburia	0.016901982	0.0060099	0.023136882	0.009277615	0.007868778	0.031554524	0.007575651	0.041149727	0.013065299
Roseibacillu	0.000257942	6.97E-05	0.000296468	0	0.00022644	0	0	0	0
Rothia	0	0	0	0	0	5.30E-05	0	0	0
Ruegeria	0.000108607	0.000780869	0.000395291	0.002061692	0.00182567	0	0	0	0
Ruminiclost	0	0	0	0	0	0	0	0	4.09E-05
Ruminiclost	9.50E-05	6.97E-05	9.88E-05	5.29E-05	7.08E-05	0	0	0	0
Ruminiclost	0.00175129	0.000808757	0.000778229	0.001995612	0.001471858	0.001829632	0.000801136	0.000397119	0.000681905
Ruminiclost	0.001601955	0.00068326	0.001161168	0.001770941	0.000410422	0.000357971	0	0.000465588	0.000300038
Ruminiclost	0.00046158	0.000460155	0.000333527	0.000383263	0.000509489	0.000331455	0.000829246	0.001602169	0.00079101
Ruminococ	0.001303285	0.00228683	0.003224093	0.002881083	0.002306854	0.000835267	0.000632476	0.003820557	0.005755278
Ruminococ	0.003461852	0.003639406	0.012525787	0.002815003	0.006425225	0.001020882	0.000969796	0.006983814	0.013092575
Ruminococ	0.00076025	0.00105975	0.001074698	0.000555071	0.000905759	0.000172357	0.000660586	0.000492975	0.002482134
Ruminococ	0.000176487	0.000250994	0.000148234	7.93E-05	0.000325507	0	8.43E-05	0.000301263	0.000381867
Ruminococ	0.003326093	0.004908318	0.014835769	0.003911929	0.004118371	0.002439509	0.003541863	0.004245063	0.007310021
Ruminococ	0	0	0	0	0	0	0	0	0
Ruminococ	2.72E-05	0	0.000111176	0.00013216	0.000410422	0	5.62E-05	0.00076685	0.001841143
Ruminococ	0.003909856	0.002203165	0.004484083	0.003026458	0.005349637	0.008737156	0.00044976	0.001287213	0.002113905
Ruminococ	0.003746945	0.001575682	0.003656443	0.004044088	0.000778386	0.017898575	0.000899521	0.00208145	0.015956576
Ruminococ	0.006557154	0.002816705	0.006929947	0.004784183	0.004302354	0.001803116	0.001419556	0.002177307	0.011756042
Ruminococ	0.002769481	0.00198006	0.00212469	0.003951577	0.002646514	0.000768976	0.002080142	0.003642538	0.005346135
Sanguibacte	0	0	0	0	0	0	0	0	0
Sarcina	0	0	0	0.000396479	0	0	9.84E-05	0	0
Sediminibac	0	0	0	0	0	0	0	0	0
Selenomona	0	0	0	0	0	0	0	0.000534056	0
Sellimonas	0	0	0	0.000105728	0	0.000159098	0	0	0
Senegalima	0	0	0	0	9.91E-05	0	0	0.00010955	0.000231848
Serratia	0	0	0	0	0	0	0	0	0
Shuttleworth	0	0	0	0	0	0	0	0	0
Slackia	0	0	2.47E-05	0	0	0	0	4.11E-05	0.000150019
Sneathiella	8.15E-05	0.000111553	0	0	0	0	0	0	0
Solobacteriu	0	0	3.71E-05	5.29E-05	4.25E-05	0	0	0.000136938	0.000136381
Sphingobac	0	0	0	0	0	0	0	0	0
Sphingomor	0	0.000139441	0	0	0	0	0	0	0
Staphylococ	0	0	0	0	0	0	0	0	0
Stenotropho	0	0.000139441	0	0	0	0	0	0	0
Streptococc	0.002959544	0.002621488	0.002507628	0.007638834	0.012949518	0.005899901	0.000997906	0.002752444	0.0008592
Subdoligran	0.005783329	0.005368472	0.012599904	0.013189544	0.007260222	0.016479947	0.002768837	0.006449758	0.014701871
Succinatimo	0	0	0	0	0	0	0	0	0
Succinivibrio	0	0	0	0	0	0	0	0	0
Sulfitobacte	0.001887049	0.001045806	0.000321174	0.000541855	0.000976521	0	0	0	0
Sutterella	0.002280749	0.001966116	0.002099984	0.009528718	0.027625639	0.01512761	0.00019677	0.005299482	0.025639627
Synergistes	0	0	0	0	0.000127372	0	0	0	0
Taeseokella	0.000325821	0.000334658	0.000827641	0.000356831	0	0	8.43E-05	0	0
Tepidimicro	0	0	0	0	0	0	0	0.0002191	0
Terrisporob	0.000190062	0.000181273	0.000197646	0.000330399	0.000976521	0.000450779	0	0	0.000477333
Thalassotal	0	0	3.71E-05	0.00019824	0.000325507	0	0	0	0
Thiopseudo	0	0	0	0	0	0	0	0	0
Tissierella	0	0	0	0	0	0	0	0.001889738	0.000490972
Turicibacter	0.000420853	0.000599596	0.000580584	0.005458198	0.000721776	0.001272788	0.00014055	0	0.000245486
Tyzzerella	0	0	0	0	0	0.000464037	0.001293061	0.000314956	0.000422781
Tyzzerella 3	0.00640782	0.00037649	0.000358233	0.001110142	0.00028305	0.000185615	0.000267045	6.85E-05	0.000231848
Tyzzerella 4	0.000339397	0.001422297	0.00034588	0.000515423	0.001018978	0.000304939	0.000323265	0.000739463	0.000204571
UBA1819	0.000312245	0.000529875	0.000407644	0.00026432	0.000311355	0.000159098	0.00030921	0.000753156	0.000245486
uncultured	0.01367092	0.012075577	0.010092276	0.015845955	0.00850564	0.005541929	0.010316378	0.018185304	0.005059735
uncultured b	0.00076025	0.001798787	0.000308821	0.000436127	0.002731428	0.000198873	0.00014055	0.000273875	0.003109487
uncultured b	0	0	0	0	0	0	5.62E-05	0.000232794	0.000831924
uncultured E	0	0.000111553	0	0	0	0	0	0	0
uncultured E	0.000108607	4.18E-05	3.71E-05	6.61E-05	5.66E-05	0	0	0	0
uncultured C	0.000108607	0	3.71E-05	3.96E-05	0.000127372	0	0	0	0
uncultured C	0	0	0	0	0	0	0	0	0
uncultured g	9.50E-05	0	0	0	0	0	0	0	0

uncultured r	0.000176487	0.000125497	0.000148234	5.29E-05	0.00011322	0	0	0	0
uncultured c	0.000380125	0.000474099	0.000457055	0.000687231	0.000410422	0.00014584	0.000351375	0.002396407	0.003450439
uncultured F	0	0	0	0	0.000198135	0	0	0.000397119	0.000668267
uncultured p	0.000162911	6.97E-05	0.000135881	2.64E-05	0	0	0	0	0
uncultured r	0	0.000167329	0	0	0	0	0	4.11E-05	5.46E-05
uncultured S	0	0	0	0	0	0	5.62E-05	0	0
uncultured T	0	0	0	0	0	0	0	0	0.000109105
uncultured V	0.000325821	0.00061354	0.001371166	0.000330399	0.00011322	0	0	0	0
unidentified	0	0	0	0	0	0	0	0	0.000395505
Veillonella	0.000583763	0.000599596	0.001321755	0.002154204	0.00176906	0.003526682	0.00059031	0.000191713	0.001745677
Vibrio	0	0	0	0	0	0	0	0	0
Victivallis	0	0	0	0	0.002151177	0	0	0.0002191	0.000231848
W5053	0	0	0	0	0	0	0	2.74E-05	0
Weissella	0.000407277	4.18E-05	0.00025941	5.29E-05	0.00011322	0.00014584	0	0	0.000204571
Wenyingzhu	0	0	3.71E-05	0	0.000141525	0	0	0	0
Woeseia	0	0	0.000111176	0	0	0	0	0	0
Genus	W27	W30	W31	W34	W35	W3	W47	W53	W6
[Caedibacte	0	0	0	0	0	0	0	0	0
[Clostridium	0.000129679	0	7.62E-05	0	0.000120871	0.000114575	0	0	0
[Eubacteriu	0	0	0	0	0	0	0	0	0
[Eubacteriu	0.00374629	0.009278326	0.016245295	0.003878652	0.002308631	0.00438251	0.004952695	0.00406969	0.046264347
[Eubacteriu	0.003069076	0.009423678	0.009067496	0.018398362	0.001232882	0.027899116	0.001777891	0.002119129	0.010040726
[Eubacteriu	0.001786692	0.001283946	0.001569667	0.000937221	0.000604354	0.00088796	0.000215887	0.001601387	0.001155128
[Eubacteriu	0	0	0	0	0	0.000114575	0	0	0.000251759
[Eubacteriu	0.001714648	0.002059158	0.002468797	0.001860022	0.00099114	0.001704309	0.000749254	0.000987321	0.000592373
[Eubacteriu	0.001152705	0.000738874	0.000868651	0.003792139	0.000217567	0.000673131	0.000584164	0.004454987	0.000681229
[Eubacteriu	0.000691623	0.001138593	0.000975327	0.000331632	0.000338438	0.000214829	0.000698457	0.000650187	0.001007034
[Polaribacte	0	0	0	0	0	0	0	0	0
[Ruminococ	0.000475491	0	0.00028955	0	0	0	0	0.000192648	0
[Ruminococ	0.002017233	0.000702536	0.00565385	0.002725149	0.004653524	0.001031179	0.000546066	0.00668248	0.000488708
[Ruminococ	0.004380277	0.001477749	0.003992746	0.00366237	0.001970193	0.007160964	0.002831926	0.005406186	0.003524621
Abiotrophia	0	0	0	0	9.67E-05	0	0	0	0
Acetanaerol	0	0	4.57E-05	0	3.63E-05	0	0	0	4.44E-05
Acetitomacu	0	0	0	0	0	0	0	0	0
Acetobacter	0	0	0	0	0	0	0	0	0
Acidaminoc	0.000864528	0.016703409	0.001356314	0.000836289	0.000676876	0.003165146	0.000355578	0.000325094	0.000385043
Acinetobact	4.32E-05	0	0	0	0	0	0	0	0
Actinobacillu	0	0	0	0	0	0	0	0	0
Actinomyce	0.000461082	0.000290705	0.00059434	0.000360469	8.46E-05	8.59E-05	8.89E-05	0.000156527	0
Adlercreutzi	0.000187314	0.000121127	0	0	0	0	0	0	8.89E-05
Aeromonas	0.00011527	0	0.005562413	0.000100931	0	0	6.35E-05	0	0
Aerosphaer	0	0	0	0	0	0	0	0	0
Aestuariicel	0	0	0	0	0	0	0	0	0
Agathobact	0.034163281	0.004736064	0.003474603	0.004455403	0.000229654	0.003766667	0.048003048	0.060912911	0.009552018
Aggregatiba	0	0	0	0	0	0	0	0	0
Akkermansi	0.005302441	0.003173526	0.00179826	0.001643741	0.001317491	0.001718631	0.000114293	0.000421418	0.001007034
Alcaligenes	0	0	0	0	0	0	0	0	0
Alcanivorax	0	0	0	0	0	0	0	0	0
Alistipes	0.025445953	0.112212021	0.016504366	0.020445829	0.004290912	0.054953239	0.008064004	0.013461284	0.025931137
Allisonella	0	0.000787325	0.001981134	0.000432563	0	0.00037237	0.00129532	0.000313053	0.000533136
Allobaculum	0	0	0	0	0	0	0	0	0
Alloprevotel	0	0.000145352	0	0	0	0.000615843	0.000177789	0	0.001436505
Allorhizobiu	0	0	0	0	0	0	0	0	0
Alloscardov	0	0	0	0	0	0	0	0	0
Amphritea	0	0	0	0	0	0	0	0	0
Anaerofilum	0	0	0	0	0	0	0	0	0
Anaeroglobu	0	0.000508733	0	0	0	0	0	0	0
Anaerospor	0.000158497	0.000387606	0.000228592	0.00011535	0	0.001160076	0.000152391	0.000301013	0.000414661
Anaerostipe	0.003472522	0	0.000487664	0.00059117	0.001510884	0.001074145	7.62E-05	0.001023443	0.00022214
Anaerotrunc	0.000389038	0.000557184	0.000182874	0.000158607	7.25E-05	0.000128897	0.000101594	0	0.000370233
Angelakisell	0.000100862	4.85E-05	0	0	0	8.59E-05	0	0	0
Anoxybacillu	0	0	0	0	0	0	0	0	0
Anseongella	0	0	0	0	0	0	0	0	0
Arenibacter	0	0	0	0	0	0	0	0	0
Atopobium	0	0	0.000243832	0.000245119	0	0	0	0	0
Atopostipes	0	0	0	0	0	0	0	0	0
Aureimarina	0	0	0	0	0	0	0	0	0
Azospirillum	0.00036022	0.000496621	0.00088389	0.00047582	0.000374699	0	0	0	0
Bacillus	0	0	0	0	0	0	0	0	0
Bacteroides	0.596524596	0.378243174	0.323366708	0.462640944	0.766223877	0.366956447	0.155184456	0.501836177	0.245168456
Balneola	0	0	0	0	0	0	0	0	0
Barnesiella	0.000662805	0.001635214	0.001051525	0.006877758	0.00029009	0.025607608	0.001396914	0.00764572	0.002191781
Bifidobacter	0.001613786	0.001744228	0.001554428	0.001009315	0.000882356	0.000200507	0.000342879	0.001806076	0.000355424
Bilophila	0.004265007	0.004796628	0.001889697	0.002537705	0.000592267	0.008120533	0.000927043	0.000939159	0.00242873
Blautia	0.008256246	0.008127619	0.012450662	0.020705367	0.008690607	0.003236756	0.009981586	0.006104536	0.006279156
Bosea	0	0	0	0	0	0	0	0	0
Brevibacillus	0	0	0	0	0	0	0	0	0
Brevundimo	0	0	0	0	0	0	0	0	0
Butyricicocc	0.005028674	0.001223382	0.004632805	0.002133979	0.003879951	0.000859316	0.002450949	0.003010126	0.001599408
Butyricimon	0.000172906	0.000993241	0.000441945	0.002797243	0	0.005356401	0.001079434	0.002721154	0.000784894

Butyrivibrio	0	0	0	0	0	0	0	9.63E-05	0
C1-B045	0	0	0	0	0	0	0	0	0
CAG-352	0	0	0	0	0	0	0	0	0
CAG-56	0.002118095	0.00013324	0.001447751	0.000158607	0.00029009	0	0.000215887	0.000216729	0.000355424
CAG-873	0	0	0	2.88E-05	0	0	0	0	0
Campylobac	0	0	0	0	0	0	0	0	0
Candidatus	0	0	0	0	0	0	0	0	0
Candidatus	0	0	0	0	0	0	0	0	0
Candidatus	0	0	0	0	0	0	0	0	0
Candidatus	0	0	0	0	0	0	0	0	0
Candidatus	0	0	0	0	0	0	0	0	0
Candidatus	4.32E-05	0.00013324	0	0	0	5.73E-05	0	0	0
Candidatus	0	0	0	0.000100931	0.000108784	0	6.35E-05	9.63E-05	0
Caproicipro	0	0	0	0	0	0	0	0	0
Catabacter	0	0	0	0	0	0	0	0	0
Catenibacte	0	0.000230141	0	0	0	0	0	0	0
Cellulosilytic	0	0	0	0	0	0	0	0	7.40E-05
Cellvibrio	0	0	0	0	0	0	0	0	0
Cephalotico	0	0	0	0	0	0	0	0	0
Christensen	0	0	0	0	0	0	0	0	0
Christensen	0.002262183	0.005305361	0.006156753	0.002898175	0.001087837	0.002133967	0.001346117	0.002925843	0.005420215
Chryseoline	0	0	0	0	0	0	0	0	0
Chthoniobac	0	0	0	0	0	0	0	0	0
Clavibacter	0	0	0	0	0	0	0	0	0
Cloacibacillu	0	0	0	0	0	0	0	0	0
Clostridiales	0	0	0	0	0	0	0	0	0
Clostridioide	0	0	0	0	0.000278003	0	0	0	0
Clostridium	0.00325639	0.001720003	0.011780125	0.001643741	0.000568093	0.000272117	0.00146041	0.001276293	0.00331729
Collinsella	0.000662805	0.006250151	0.002819305	0.000273957	0.004266738	0.00030076	0.000368277	0.000337134	0.000281377
Colwellia	0	0	0	0	0	0	0	0	0
Comamona	0	0.000242254	0	0	0	0	0	0	0
Coprobacillu	0.000273767	0.00013324	0	0	0	0	0	0	4.44E-05
Coprobacte	0.000288176	6.06E-05	0.000106676	7.21E-05	0	0.001203042	0.00049527	0.001938521	0.001954832
Coprococcu	0.000374629	0.000302817	0	0	7.25E-05	8.59E-05	0	0.000180608	0.000251759
Coprococcu	7.20E-05	9.69E-05	0.000624819	0	8.46E-05	0.001732953	0.000952441	0.000650187	0.011906701
Coprococcu	0.000201723	0.000423944	0.006492022	0.00059117	0.00020548	0.000802028	0.000368277	0.001348536	0.000992225
Coriobacter	0	0	0	0	0	0	0	0	0
Coxiella	0	0	0	0	0	0	0	0	0
Crocinitomix	0	0	0	0	0	0	0	0	0
Cyclobacter	0	0	0	0	0	0	0	0	0
Defluviitalea	0	7.27E-05	0	0	0	0	0	0	0
Deinococcu	0	0	0	0	0	0	0	0	0
Delftia	0	0	0	0	0	0	0	0	0
Desulfovibri	0.000878937	0.005898883	0.000441945	0.000634426	0.000169219	0.00207668	0.000203188	0.000337134	0.0011107
Dialister	0.006210196	0.039475278	0.047211936	0.003114456	0.014178139	0.008335362	0.001981078	0.000854876	0.018393188
Dielma	0	0.00013324	7.62E-05	0	0.000386786	4.30E-05	0	0	4.44E-05
Dolosicoccu	0	0	0	0	0	0	0	0	0
Donghicola	0	0	0	0	0	0	0	0	0
Dorea	0.002680038	0.001065917	0.006933967	0.001787929	0.000942792	0.00156109	0.001485809	0.003419503	0.00176231
DTU089	0	0	0	0	0	0	0	0	0
Dubosiella	0	0	0	0	0	0	0	0	0
Dysgonomo	0	0	0	0	0	0	0	2.41E-05	0
Eggerthella	8.65E-05	0.000157465	7.62E-05	0.00059117	0.000229654	0.000100253	5.08E-05	0.000168567	0.000118475
Eisenbergie	0.000187314	0.000302817	0.000335269	0.000216282	0.00029009	0.000157541	0	0.000385296	0
Enhydrobac	0	0	0	0	0	0	0	0	0
Enterococcu	0.000230541	0	0	0	0	0.000286439	0	0.000264891	0.000192521
Enterorhabd	0	0	3.05E-05	0	0	0	0	0	0
Epulopisciu	0	3.63E-05	0	0	0	0	0	0	8.89E-05
Erysipelatoc	0.000734849	0.001162819	0.001158201	0.000663264	0.001160359	0.000386692	0.000126992	0.000830795	0.000266568
Erysipelotric	0.005043082	0.000738874	0.001173441	0.003864233	0.002054803	0.000429658	0.001892184	0.002889721	0.001243984
Erysipelotric	0	0	0	0	0	0	0	0	0.000414661
Escherichia-	0.025590041	0.015371012	0.031637178	0.034114831	0.007941208	0.006273005	0.001574703	0.002817478	0.004220659
Eubacterium	2.88E-05	0	0	0	0	0	0	0	0
Ezakiella	0	0	0	0	0	0	0	0	0
Fabibacter	0	0	0	0	0	0	0	0	0
Faecalibact	0.015892914	0.01661862	0.019597982	0.042218185	0.008436779	0.037795569	0.006502	0.014641253	0.060066642
Faecalibacu	0	0	0	0	0	0	0	0	0
Faecalicocc	0	0	0	0	0	0	0	0	0
Faecalitalea	0	0	0.000274311	0.000173025	0	0.000100253	0	9.63E-05	0.000118475
Family XIII A	0.000100862	0.00018169	0.000198113	0.000100931	0	0	0	0.000132446	0.000251759
Family XIII U	7.20E-05	0.000145352	0.000243832	5.77E-05	0	0.000114575	7.62E-05	0	0
Flavonifract	0.000734849	0.000726762	0.001767781	0.000432563	0.002792114	0.000558555	0.000393676	0.000529782	0.000473899
Formosa	0	0	0	0	0	0	0	0	0
Fournierella	0	0	0	0	0	8.59E-05	0	0	0.000103665
Fusicateniba	0.00181551	0.002265074	0.003931788	0.002148398	0.002816289	0.00037237	0.000419074	0.00381684	0.000947797
Fusobacteri	0.001195931	0.000411832	0.002362121	0.002436774	0.003094291	0.000673131	0.094088514	0.008777528	0.005242503
GCA-90006	0	0	0	0	0	0	0	0	0
GCA-90006	0	0	0	0	0.000350525	0	0	0	0
Gemella	0	0.000145352	0.000777214	0.000158607	0	5.73E-05	5.08E-05	8.43E-05	0
Gimesia	0	0	0	0	0	0	0	0	0
Gluconobac	0	0	0	0	0	0	0	0	0

Gordonibac	0	0	0	0	0	0	0	0	0
Gottschalkia	0	0	0	0	0	0	0	0	0
Granulicatel	0	0.00013324	0.000640059	0.000173025	7.25E-05	0	6.35E-05	0	0
Haemophilu	0.00060517	0.001344509	0.012587818	0.018874182	0.000386786	8.59E-05	0.000228586	0.000770592	0.000162903
Harryflintia	8.65E-05	0	0	0	0	0	0	0	0
hgcI clade	0	0	0	0	0	0	0	0	0
Hoeflea	0	0	0	0	0	0	0	0	0
Holdemanel	0.000662805	0.001223382	0.003947028	0.001254434	0.000652702	0.000716096	8.89E-05	0.002323817	0.000696039
Holdemania	0.000273767	8.48E-05	0.000167634	0.000100931	0.000108784	0	0	0	0
Howardella	0	3.63E-05	0	0	0	4.30E-05	0	0	0
Hungatella	0.000979799	0.001574651	0	0.000519076	0.003275597	0.000229151	0.000126992	0.000349175	0
Hydrogenoa	0.00011527	0.000278592	0	0	0	0	0	0	8.89E-05
Ileibacteriun	0	0	0	0	0	0	0	0	0
Intestinibact	0.002118095	0.003536907	0.001889697	0.001023733	0.001027401	0.000358048	0.000558766	0.000493661	0.001125509
Intestinimor	0.000129679	0.000339156	0.000320029	0.000100931	0.000108784	0.000257795	0	0	0.00022214
Jeotgalibaca	0	0	0	0	0	0	0	0	0
Kordiimonas	0	0	0	0	0	0	0	0	0
Lachnoclost	0.021209763	0.013130163	0.017098706	0.009242437	0.02190178	0.00713232	0.077516033	0.005791483	0.011669752
Lachnoclost	0.000936572	0	0	0	0	0	0.000507969	0.011077264	0.001969641
Lachnoclost	0.00036022	0	0	0	0	0	0	0	0
Lachnospira	0.035042218	0.015637491	0.063548667	0.115234882	0.013730918	0.004998353	0.006679789	0.123631898	0.014202147
Lachnospira	0.000273767	0	0.000777214	0.000533495	0	0	0.000215887	0	0.000192521
Lachnospira	0.000835711	0.00089634	0.00120392	0.000778614	0.000314264	0.00037237	0.00016509	0.000421418	0.001125509
Lachnospira	0.003342843	0.009823397	0.003108856	0.002941431	0.001099924	0.004296578	0.001777891	0.004635594	0.007330618
Lachnospira	0.000446673	0.001187044	0.00149347	0.004599591	0.000918618	0.002019392	0.000774652	0.024562629	0.000799704
Lachnospira	0	8.48E-05	0	0.000793033	0	0	0.000406375	0.000565904	0
Lachnospira	0.002247774	0.003900288	0.003657477	0.003474926	0.007868686	0.001475159	0.001841387	0.003624192	0.003287671
Lachnospira	0.000302585	0	0	0	0	0	0	0	0
Lachnospira	0.000706032	0.001005354	0.014584191	0.001629322	0.000459309	0.001432193	0.000546066	0.002504425	0.001140318
Lacticigeniu	0	0	0	0	0	0	0	0	0
Lactobacillu	0.000533126	0.00044817	0.000335269	0.002451192	4.83E-05	7.16E-05	0.000126992	0.000734471	0
Lactococcus	0	0	0	0	0	0.000100253	0	0	0
Lentibacter	0	0	0	0	0	0	0	0	0
Leuconostoc	0	0	0	0	0	0	0	0	0
Leucothrix	0	0	0	0	0	0	0	0	0
Litoricola	0	0	0	0	0	0	0	0	0
Mailhella	0	0	0	0	0	0	0	4.82E-05	0
Mannheimia	0	0	0	0	0	0	0	0	0
Maribacter	0	0	0	0	0	0	0	0	0
Marinicella	0	0	0	0	0	0	0	0	0
Marinobacte	0	0	0	0	0	0	0	0	0
Marivita	0	0	0	0	0	0	0	0	0
Marvinbryar	0	0.000363381	0	0	0	0	0	0	0
Massilia	0	0	0	0	0	0	0	0	0
Megamonas	0.001167113	0.005608178	0.028330209	0.016610433	0.001208708	0.001919138	0.264639025	0.00215525	0.069041096
Megasphae	0.002521541	0.096513966	0.01729682	0.004426565	0.001897671	0.019119774	0.035214934	0.00238402	0.028922621
Merdibacter	0	0	0	0	0	0	0	0	0
Methanobre	0	0	0	0	0	0	0	0	0
Methylobact	0	0	0	0	0	0	0	0	0
Methylotene	0	0	0	0	0	0	0	0	0
Mf105b01	0	0	0	0	0	0	0	0	0
Micrococcus	0	0	0	0	0	0	0	0	0
Mitsuokella	0	0	0	0.003734464	0	0	0.002171566	0.002131169	0.000148093
Mogibacteri	0	0	0.000152395	0.00011535	0	0	0	0	0
Morganella	0.000158497	0	9.14E-05	8.65E-05	0.000108784	0	0	0.000132446	0
Moryella	0	0	0	0	0	0	0	9.63E-05	7.40E-05
Murimonas	0	0	0	0	0.000120871	0	0	0	0
Mycobacter	0	0	0	0	0	0	0	0	0
Negativibac	0.000288176	0	0.000350508	0.000245119	0.000181306	0.000272117	5.08E-05	0	0.000266568
Nitrosomon	0	0	0	0	0	0	0	0	0
Nosocomiic	0	0	0	0	0	0	0	0	0
NS3a marin	0	0	0	0	0	0	0	0	0
Oblitimonas	0	0	0	0	0	0	0	0	0
Oceanicocc	0	0	0	0	0	0	0	0	0
Oceanobact	0	0	0	0	0	0	0	0	0
Oceanospiri	0	0	0	0	0	0	0	0	0
Odoribacter	0.000979799	0.001925919	0.000579101	0.002537705	0.00078566	0.004554373	0.000571465	0.00262483	0.000710848
Olsenella	0	0	0	0	0	0	0	0	0
OM27 clade	0	0	0	0	0	0	0	0	0
Oribacteriur	0	0	0	0	0	0	0	0	0
Oscillibacte	0.000417855	0.000351268	0.000563861	0.000562332	0.000398873	0.000673131	0.000177789	0.000361215	0.000799704
Oscillospira	0.000129679	0	0	0	0	0	0	0	0
Ostreobium	0	0	0	0	0	0	0	0	0
Oxalobacter	0.000144088	0.000145352	9.14E-05	8.65E-05	0	0.000100253	0.000126992	0	0.000177712
Paenalcalig	0	0	0	0.000331632	0	0	0	0	0
Paeniclostri	0	0	0.002042091	0	0	0	0	0	0
Parabacterc	0.018486499	0.010525933	0.0131212	0.023012371	0.023424752	0.046044999	0.015823227	0.015785101	0.020836727
Paraclostrid	0	0	0.000137155	0	0	0	0	0	0
Paracoccus	0	0	0	0	0	0	0	0	0
Parahaliea	0	0	0	0	0	0	0	0	0
Paralcaliger	0	0	0	0	0	0	0	0	0

Paraprevote	0.02354399	0.001223382	0.008610311	0.003748883	0.000834008	0.000243473	0.013004	0.011137466	0.003184006
Parasuttere	0.011613498	0.057583759	0.017601609	0.010756409	0.015821982	0.019119774	0.001777891	0.015941628	0.001614217
Patulibacter	0	0	0	0	0	0	0	0	0
Pediococcu	0	0	0	0	0	0	0	0	0
Pedobacter	0	0	0	0	0	0	0	0	0
Peptoclostri	0	0	0	0	0	0	0.000203188	0	0
Peptococcu	0	3.63E-05	0.000213353	0	0	8.59E-05	0	0	0
Phascolarct	0.005374485	0.003258315	0.002529755	0.005363786	0.002610808	0.016112169	0.024014223	0.002119129	0.005005553
Phocea	0	0	0	0.000144188	0	0.000157541	0	7.22E-05	8.89E-05
Planctomicr	0	0	0	0	0	0	0	0	0
Polynucleob	0	0	0	0	0	0	0	0	0
Porticoccus	0	0	0	0	0	0	0	0	0
Prevotella	0	4.85E-05	7.62E-05	0	0	0	0	0	0
Prevotella 2	7.20E-05	0.001005354	0.000121916	8.65E-05	0.000120871	0.004196325	0	0.000337134	0.000932988
Prevotella 6	0	0	0	0	0	0	0	0	0
Prevotella 7	0	0.000545071	0.000243832	0.00011535	0	0.000171863	0	0	0.000192521
Prevotella 9	0.00096539	0.000411832	0.004541368	5.77E-05	0	0.230812197	0.138230999	0.008380191	0.196268049
Prevotellace	0	0	0	0	0	0	0	0	0
Prevotellace	0.000259359	0.000157465	0.001219159	0.000346051	0.000132958	0.000873638	0	0	0.000755276
Prevotellace	0	0	0	0	0	0	0	0	0
Prevotellace	0	0.000145352	0	0	0	4.30E-05	0	0	5.92E-05
Proteocatell	0	0	0	0	0	0	0	4.82E-05	0
Proteus	0.000590761	0	0.000137155	0.000446982	0	0	6.35E-05	0	0
Providencia	0	0	0.000167634	0	0	0	0	0	0.000192521
Pseudoalter	0	0	0	0	0	0	0	0	0
Pseudochro	0	0	0	0	0	0	0	0	0
Pseudoflavo	0	0	0	0	0	0	0	0	0
Pseudohong	0	0	0	0	0	0	0	0	0
Pseudomon	0	0	0	0.000201863	0	0	0	0	0
Pseudophae	0	0	0	0	0	0	0	0	0
Pseudovibri	0	0	0	0	0	0	0	0	0
Pyramidoba	0.00024495	0.005038882	0.000198113	0.000129769	0	0.00200507	8.89E-05	0	0.000725657
Raoultibacte	0	0	0	0	0	0	0	0	0
Reichenbac	0	0	0	0	0	0	0	0	0
Rheinheime	0	0	0	0	0	0	0	0	0
Rhodococcu	0	0	0	0	0	0	0	0	0
Rhodoferax	0	0	0	0	0	0	0	0	0
Rikenellacea	2.88E-05	0.000799438	6.10E-05	0	0	0.000243473	8.89E-05	0	0.000266568
Romboutsia	0.002550359	0.002240849	0.01638245	0.002523286	0.001063663	0.000386692	0.000546066	0.000638147	0.001258793
Roseburia	0.014985159	0.015031856	0.055471738	0.035412521	0.006079799	0.00401014	0.003631977	0.015002468	0.018511662
Roseibacillu	0	0	0	0	0	0	0	0	0
Rothia	5.76E-05	0	0.000243832	0.000100931	0	0	0	0	2.96E-05
Ruegeria	0	0	0	0	0	0	0	0	0
Ruminiclost	5.76E-05	0	0	0	0	0	0	0	0
Ruminiclost	0	0.000169578	7.62E-05	0	0	0	0	0	0
Ruminiclost	0.000590761	0.000472395	0.001295357	0.001009315	0.001752626	0.000572877	0.000266684	0.000313053	0.000636801
Ruminiclost	0.000706032	0.001029579	0.000822932	0.001744672	0.000217567	0	0.000380977	0.000180608	0.000266568
Ruminiclost	0.000734849	0.000423944	0.001508709	0.000620007	0.000882356	0.000415336	0.000711156	0.000397337	0.001643836
Ruminococ	0.002795308	0.003536907	0.005425258	0.004094933	0.001051576	0.003995818	0.00210807	0.003684394	0.00463532
Ruminococ	0.003731881	0.00754621	0.008854143	0.004887966	0.000737312	0.004625983	0.004076449	0.00597209	0.009744539
Ruminococ	0.001152705	0.002822258	0.002103049	0.00070652	0.000362612	0.000501267	0.000838148	0.000589985	0.00155498
Ruminococ	0.000345811	6.06E-05	0.00059434	0.0002307	7.25E-05	0.000802028	6.35E-05	0.000120405	0.000251759
Ruminococ	0.00168583	0.005886771	0.003840351	0.003619114	0.001148272	0.002620913	0.001739793	0.002371979	0.006990004
Ruminococ	0	0	0	0	0	0	0	0	0.000118475
Ruminococ	0.000403447	0.001090143	0.000990567	0.000317213	6.04E-05	0.00030076	0.000342879	0.000397337	0.002399111
Ruminococ	0.004898994	0.005002544	0.01580335	0.003200969	0.002175674	0.000544233	0.001333418	0.004129893	0.001081081
Ruminococ	0	0.003730711	0.00207257	0	0	0.001589734	0.00081275	0.004442946	0.013195113
Ruminococ	0.003097893	0.008200296	0.004983313	0.004440984	0.00099114	0.003465907	0.005028891	0.014532889	0.006930766
Ruminococ	0.008991095	0.004796628	0.008198845	0.001816766	0.000809834	0.002363118	0.001663598	0.002745235	0.003465383
Sanguibacte	0	0	0	0	0	0	0	6.02E-05	0
Sarcina	0	0	0	0	0	0	0	0	0
Sediminibac	0	0	0	0	0	0	0	0	0
Selenomona	0	0	0	0.000576751	0	0	0.000342879	0.00022877	0
Sellimonas	0	0.000460282	0.000106676	0	0	0	0	0	0
Senegalima	0	0.000121127	0.000228592	0	0	0	0	0	5.92E-05
Serratia	0	0	0	0	0	0	0	0	0
Shuttleworth	0	0	0	0	0	0	0	0	0
Slackia	0	0.000121127	0	0	0	4.30E-05	0	0.000144486	0.000118475
Sneathiella	0	0	0	0	0	0	0	0	0
Solobacteriu	0	0.000254367	0.000137155	7.21E-05	0	7.16E-05	5.08E-05	0	8.89E-05
Sphingobac	0	0	0	0	0	0	0	0	0
Sphingomor	0	0	0	0	0	0	0	0	0
Staphylococ	0	0	0	0	0	0	0	0	0
Stenotropho	0	0	0	0	0	0	0	0	0
Streptococc	0.005215988	0.002979723	0.026333836	0.005926118	0.004411782	0.002606591	0.002298559	0.004009488	0.003124769
Subdoligran	0.010230253	0.006443955	0.01639769	0.010684315	0.002453676	0.003995818	0.003365293	0.003973366	0.007686042
Succinatimo	0	0	0	0	0	0	0	0	0
Succinivibri	0	0	0	0	0	0	2.54E-05	0	0
Sulfitobacte	0	0	0	0	0	0	0	0	0
Sutterella	0.005273623	0.007921704	0.005638611	0.000692101	0.002308631	0.002191255	0.010781637	0.003022167	0.04465013
Synergistes	0	0	0	0	0	0	0	0	0

Taeseokella	0	0	0	0	0	0	0	0	0
Tepidimicro	0	0	0	0.000187444	0	0	0	0	0
Terrisporob	0.000144088	0.001598876	0.000685777	0.0002307	0	0	0	0	0
Thalassotal	0	0	0	0	0	0	0	0	0
Thiopseudo	0	0	0	0	0	0	0	0	0
Tissierella	0	0	0	0.001240015	0	0	0	0	0
Turicibacter	0.000144088	0.000520846	0.000655298	0.000331632	8.46E-05	0	0	0	0.000473899
Tyzzerella	0.00024495	0.000169578	0.000259071	0.000216282	0.002248196	0	0	0	0.000148093
Tyzzerella 3	0.001195931	0.000230141	0.000365748	0.000158607	0.000229654	4.30E-05	0.000228586	0.000758552	0.00087375
Tyzzerella 4	0.000432264	0.000266479	0.001082004	0.004195865	0.005028223	0.000128897	0.000190488	0.002287696	0.00022214
UBA1819	0.00108066	0.00107803	0.001036285	0.000533495	0.000966966	0.000315082	0.000126992	0.000180608	0.000503517
uncultured	0.010460794	0.005050994	0.016031942	0.007526603	0.009536702	0.004353866	0.011797574	0.00500885	0.007138097
uncultured b	0.000619579	0.001150706	0.00179826	0.000663264	0.000471396	8.59E-05	0.001371516	0.000770592	0.002073306
uncultured b	0	0.000678311	0	0	0	0	0	0.000132446	8.89E-05
uncultured E	0	0	0	0	0	0	0	0	0
uncultured E	0	0	0	0	0	0	0	0	0
uncultured C	0	0	0	0	0	0	0	0	0
uncultured C	0	0	0	0	0	0	0	0	0
uncultured g	0	0	0	0	0	0	0	0	0
uncultured r	0	0	0	0	0	0	0	0	0
uncultured c	0.001786692	0.001756341	0.000396227	0.000692101	0	0.00118872	0.000177789	0.000638147	0.00154017
uncultured F	0	0.000593522	0.000975327	0	0	0.000214829	0	0	0.000459089
uncultured p	0	0	0	0	0	0	0	0	0
uncultured r	0	0.000290705	0.000396227	0.00011535	0.000157132	0	0.000520668	0	0.000858941
uncultured S	0	0	0	0	0	0	0	0	0
uncultured T	0	0	0	0	0	0	0	0	0
uncultured V	0	0	0	0	0	0	0	0	0
unidentified	0	0	0	0	0	0	0	0.000565904	0
Veillonella	0.000187314	0.002580004	0.007284476	0.001586066	0.001535059	0.000229151	0.00097784	0.001131807	0.000118475
Vibrio	0	0	0	0	0	0	0	0	0
Victivallis	0.000129679	0.00013324	0	5.77E-05	0	8.59E-05	0	0	0.000148093
W5053	0	0	0	4.33E-05	0	5.73E-05	0	4.82E-05	0
Weissella	8.65E-05	0.000230141	9.14E-05	4.33E-05	4.83E-05	0.000214829	0	0	0.000177712
Wenyingzhu	0	0	0	0	0	0	0	0	0
Woeseia	0	0	0	0	0	0	0	0	0

Genus	W9
[Caedibacte	0
[Clostridium	0.000161198
[Eubacteriu	0
[Eubacteriu	0.002872258
[Eubacteriu	0.002535207
[Eubacteriu	0.003707557
[Eubacteriu	0.000117235
[Eubacteriu	0.000131889
[Eubacteriu	0.000381014
[Eubacteriu	0.000146544
[Polaribacte	0
[Ruminococ	0
[Ruminococ	0.003766175
[Ruminococ	0.00927622
Abiotrophia	0
Acetanaerol	0
Acetitomacu	0
Acetobacter	0
Acidaminoc	0.000381014
Acinetobact	0
Actinobacillu	0
Actinomyce	0
Adlercreutzi	0
Aeromonas	0
Aerosphaera	0
Aestuariicel	0
Agathobacte	0.002374009
Aggregatiba	0
Akkermansi	0.000527558
Alcaligenes	0
Alcanivorax	0
Alistipes	0.003868755
Allisonella	0
Allobaculum	0
Alloprevotel	0.000644793
Allorhizobiu	0
Alloscardov	0
Amphritea	0
Anaerofilum	0
Anaeroglobu	0
Anaerospor	0
Anaerostipe	0.000337051
Anaerotrunc	0
Angelakisell	0

Anoxybacillu	0								
Anseongella	0								
Arenibacter	0								
Atopobium	7.33E-05								
Atopostipes	0								
Aureimarina	0								
Azospirillum	0								
Bacillus	0								
Bacteroides	0.635648236								
Balneola	0								
Barnesiella	0.000864608								
Bifidobacter	0.000542212								
Bilophila	0.000791336								
Blautia	0.007708202								
Bosea	0								
Brevibacillus	0								
Brevundimo	0								
Butyricicocc	0.005011797								
Butyricimon	0.000175853								
Butyrivibrio	0								
C1-B045	0								
CAG-352	0								
CAG-56	0.000322396								
CAG-873	0								
Campylobac	0								
Candidatus	0								
Candidatus	0								
Candidatus	0								
Candidatus	0								
Candidatus	0								
Candidatus	0								
Candidatus	0.000117235								
Caproicipro	0								
Catabacter	0								
Catenibacte	0								
Cellulosilytic	0								
Cellvibrio	0								
Cephalotico	0								
Christensen	0								
Christensen	0.001099078								
Chryseoline	0								
Chthoniobac	0								
Clavibacter	0								
Cloacibacillu	0								
Clostridiales	0								
Clostridioide	0								
Clostridium	0.000249124								
Collinsella	0.000381014								
Colwellia	0								
Comamona	0								
Coprobacillu	0								
Coprobacte	0.000454286								
Coprococcu	0								
Coprococcu	0.000439631								
Coprococcu	0.000161198								
Coriobacter	0								
Coxiella	0								
Crocinitomix	0								
Cyclobacter	0								
Defluviitalea	0								
Deinococcu	0								
Delftia	0								
Desulfovibri	0.000190507								
Dialister	0.001714562								
Dielma	8.79E-05								
Dolosicoccu	0								
Donghicola	0								
Dorea	0.003722212								
DTU089	0								
Dubosiella	0								
Dysgonomo	0								
Eggerthella	0.000102581								
Eisenbergie	0								
Enhydrobac	0								
Enterococcu	0								
Enterorhabd	0								
Epulopisciu	0								
Erysipelatoc	0.000967189								
Erysipelotric	0.001157696								
Erysipelotric	0								
Escherichia-	0.007590967								

Eubacterium	0								
Ezakiella	0								
Fabibacter	0								
Faecalibacte	0.039259075								
Faecalibacu	0								
Faecalicocc	0								
Faecalitalea	0								
Family XIII A	0								
Family XIII U	0								
Flavonifract	0.000498249								
Formosa	0								
Fournierella	0								
Fusicateniba	0.004440276								
Fusobacteri	0.004835944								
GCA-90006	0								
GCA-90006	7.33E-05								
Gemella	0								
Gimesia	0								
Gluconobac	0								
Gordonibact	0								
Gottschalkia	0								
Granulicatel	0								
Haemophilu	7.33E-05								
Harryflintia	0								
hgcI clade	0								
Hoeflea	0								
Holdemanel	0.000527558								
Holdemania	0								
Howardella	5.86E-05								
Hungatella	0.000556866								
Hydrogenoa	0								
Ileibacterium	0								
Intestinibact	0.000571521								
Intestinimon	0								
Jeotgalibaca	0								
Kordiimonas	0								
Lachnoclost	0.009613271								
Lachnoclost	0								
Lachnoclost	0								
Lachnospira	0.091267457								
Lachnospira	7.33E-05								
Lachnospira	0.000893917								
Lachnospira	0.003355852								
Lachnospira	0.001597327								
Lachnospira	0								
Lachnospira	0.006110875								
Lachnospira	0								
Lachnospira	0.000381014								
Lacticigeniu	0								
Lactobacillu	0								
Lactococcus	0								
Lentibacter	0								
Leuconostoc	0								
Leucothrix	0								
Litoricola	0								
Mailhella	0								
Mannheimia	0								
Maribacter	0								
Marinicella	0								
Marinobacte	0								
Marivita	0								
Marvinbryan	0								
Massilia	0								
Megamonas	0.000644793								
Megasphae	0.001362857								
Merdibacter	0								
Methanobre	0								
Methylobact	0								
Methylotene	0								
Mf105b01	0								
Micrococcus	0								
Mitsuokella	0								
Mogibacteri	0								
Morganella	0								
Moryella	0								
Murimonas	0								
Mycobacter	0								
Negativibac	0.000131889								
Nitrosomona	0								
Nosocomiic	0								
NS3a marin	0								

Oblitimonas	0								
Oceanicocc	0								
Oceanobact	0								
Oceanospiri	0								
Odoribacter	0.000439631								
Olsenella	0								
OM27 clade	0								
Oribacteriur	0								
Oscillibacter	0.000278433								
Oscillospira	0								
Ostreobium	0								
Oxalobacter	5.86E-05								
Paenalcalig	0								
Paeniclostri	0								
Parabacterc	0.009085713								
Paraclostrid	0								
Paracoccus	0								
Parahaliea	0								
Paralcaliger	0								
Paraprevote	0.000307742								
Parasuttere	0.00224212								
Patulibacter	0								
Pediococcu	0								
Pedobacter	0								
Peptoclostri	0								
Peptococcu	0								
Phascolarct	0.003898064								
Phocea	0								
Planctomicr	0								
Polynucleob	0								
Porticoccus	0								
Prevotella	0								
Prevotella 2	0.000908571								
Prevotella 6	0								
Prevotella 7	0								
Prevotella 9	0.003267926								
Prevotellace	0								
Prevotellace	0.000410323								
Prevotellace	0								
Prevotellace	5.86E-05								
Proteocatell	0								
Proteus	0								
Providencia	0								
Pseudoalter	0								
Pseudochro	0								
Pseudoflavo	0								
Pseudohong	0								
Pseudomon	0								
Pseudophae	0								
Pseudovibri	0								
Pyramidoba	0.000337051								
Raoultibacte	0								
Reichenbac	0								
Rheinheime	0								
Rhodococcu	0								
Rhodoferax	0								
Rikenellacea	0.000190507								
Romboutsia	0.000307742								
Roseburia	0.00716599								
Roseibacillu	0								
Rothia	0								
Ruegeria	0								
Ruminiclost	0								
Ruminiclost	0								
Ruminiclost	0.000981843								
Ruminiclost	8.79E-05								
Ruminiclost	0.000278433								
Ruminococc	0.00140682								
Ruminococc	0.001509401								
Ruminococc	0								
Ruminococc	0.000102581								
Ruminococc	0.001348203								
Ruminococc	0								
Ruminococc	0.000146544								
Ruminococc	0.00200765								
Ruminococc	0.001655945								
Ruminococc	0.000600829								
Ruminococc	0.001348203								
Sanguibacte	0								
Sarcina	0								
Sediminibac	0								

Selenomona	0									
Sellimonas	0									
Senegalima	0									
Serratia	0									
Shuttleworth	0									
Slackia	0									
Sneathiella	0									
Solobacteriu	0									
Sphingobac	0									
Sphingomon	0									
Staphylococ	0									
Stenotropho	0									
Streptococc	0.003736866									
Subdoligran	0.003385161									
Succinatimo	0									
Succinivibric	0									
Sulfitobacte	0									
Sutterella	0.067395478									
Synergistes	0									
Taeseokella	0									
Tepidimicro	0									
Terrisporob	0.000249124									
Thalassotal	0									
Thiopseudo	0									
Tissierella	0									
Turicibacter	8.79E-05									
Tyzzerella	0.001143041									
Tyzzerella 3	0.000117235									
Tyzzerella 4	0.00375152									
UBA1819	0.000747373									
uncultured	0.00880728									
uncultured b	0.000146544									
uncultured b	0.000102581									
uncultured E	0									
uncultured E	0									
uncultured C	0									
uncultured C	0									
uncultured g	0									
uncultured r	0									
uncultured o	0.000981843									
uncultured F	0.000307742									
uncultured p	0									
uncultured r	0									
uncultured S	0									
uncultured T	0									
uncultured V	0									
unidentified	0									
Veillonella	0.001480092									
Vibrio	0									
Victivallis	0									
W5053	0									
Weissella	0									
Wenyingzhu	0									
Woeseia	0									

Table S5. ASVs shared by at least 20% of the samples which were clustered into 46 guilds

asv_name	guild_name	Genus	Phylum	mean_relative	asv_number
99deb3c5ecb022ec05609ebd1112a557	Guild1	Bacteroides	Bacteroidetes	0.095367861	ASV1
d900eb33f6b4bc449aab64df16ae18b2	Guild1	Bacteroides	Bacteroidetes	0.060629721	ASV2
9d5054752ed33d6a22bd0f96b5618b1c	Guild1	Bacteroides	Bacteroidetes	0.011916	ASV3
836cd5ea357ad392c00a7e48d921be1d	Guild1	Bacteroides	Bacteroidetes	0.011058216	ASV4
bf611493002f5918a68778bde2def567	Guild1	Bacteroides	Bacteroidetes	0.009171928	ASV5
1569f7ae988e26c905ab8fab427c8a0d	Guild1	Phascolarctobacterium	Firmicutes	0.0055364	ASV6
a663a01aeb4dbb88bbe0a9840e8f64dc	Guild1	Erysipelatoclostridium	Firmicutes	0.002952408	ASV7
c67445ed68d61f49e41b7bdd1de019fb	Guild1	Lachnoclostridium	Firmicutes	0.002452024	ASV8
f479c23321346918723839e33a3544f4	Guild1	UBA1819	Firmicutes	0.000841233	ASV9
149465e40c1bdbc6230588a58a4d6eb0	Guild1	Ruminiclostridium 5	Firmicutes	0.00080802	ASV10
5fbc483f79cd97d3a7253931f37c33e8	Guild1	Flavonifractor	Firmicutes	0.000747456	ASV11
67e8859e108281ee7971084bfa759522	Guild1	Lachnoclostridium	Firmicutes	0.000327013	ASV12
47f770032218f91b352999ff1f663807	Guild1	Lachnoclostridium	Firmicutes	0.000323484	ASV13
a248d70e41d75f4773ef7c94e831a21a	Guild1	unclassified	Firmicutes	0.000288449	ASV14
452e8489334d0ba0cf7eeccea28f1364	Guild1	[Ruminococcus] torques group	Firmicutes	0.000257182	ASV15
629f41c0de31ba20f5d2b3f4c69e09c1	Guild1	Eggerthella	Actinobacteria	0.000223079	ASV16
d49783e7800974b5f2bcce554e26072e	Guild1	Blautia	Firmicutes	0.000167622	ASV17
5149bdbf03de13bb38557d48d5141b87	Guild1	Lachnoclostridium	Firmicutes	8.61E-05	ASV18
69a11f927915e2a3ba1f7b9c84486527	Guild2	Parasutterella	Proteobacteria	0.008141665	ASV19
76b39e86c95e0c7dd8b9c57b974f6532	Guild2	Bacteroides	Bacteroidetes	0.004547425	ASV20
1b8dec58597978264025b9eba656ffb5	Guild2	Bacteroides	Bacteroidetes	0.004466785	ASV21
dc9ddb6b3c51c6926b26dc6d65ff66c3	Guild2	Bacteroides	Bacteroidetes	0.001433267	ASV22
a12a086985ef700cc8f5a0bd3c7b45c1	Guild2	Barnesiella	Bacteroidetes	0.001303935	ASV23
fbd67349783ebeacc69814257b694d56	Guild2	Bacteroides	Bacteroidetes	0.000816485	ASV24
c45f923d6512559cb3c2e622d54fd7f6	Guild2	Megasphaera	Firmicutes	0.000417367	ASV25
ee6f1ad3007b7380824da57876e6c8c3	Guild2	Ruminococcaceae UCG-005	Firmicutes	8.12E-05	ASV26
3916a456bb0a3bfd02c15fd537ff5923	Guild2	Megasphaera	Firmicutes	7.47E-05	ASV27
506ee17cb5d882636086031fa2232f11	Guild2	uncultured organism	Bacteroidetes	6.64E-05	ASV28
b15193fce14759d1c06728933e044af6	Guild3	Bacteroides	Bacteroidetes	0.024696503	ASV29
742e1f191223f58590661de80b9d6e3b	Guild3	Alistipes	Bacteroidetes	0.013086433	ASV30
332a6411ff00b4010ac2397f504c4695	Guild3	Alistipes	Bacteroidetes	0.0114668	ASV31
506efac48dc5d5be2c0122c381639a3f	Guild3	Alistipes	Bacteroidetes	0.005655513	ASV32
a730ef0685ea600be854be1842a6b362	Guild3	Odoribacter	Bacteroidetes	0.002772037	ASV33
b2062ae94fb00a931014b67b499295a8	Guild3	Bacteroides	Bacteroidetes	0.002458463	ASV34
8001826dafd338cf078dd28c2eae05ed	Guild3	Bacteroides	Bacteroidetes	0.002011572	ASV35
8be9dac75adfd36649c004c8b72de91d	Guild3	Bacteroides	Bacteroidetes	0.001620063	ASV36
efa231614a5eb97a385a5cae7b440348	Guild3	Alistipes	Bacteroidetes	0.000201071	ASV37
b0e56c25ca193e27096e8fb4eb560eee	Guild3	Eisenbergiella	Firmicutes	0.00019141	ASV38
7a6d4a1bfc7606e2b28d1aec9abe8368	Guild3	unclassified	Proteobacteria	7.76E-05	ASV39
da1d26d90a7443d34675778207d2c227	Guild3	Ruminiclostridium 9	Firmicutes	3.97E-05	ASV40
44158349d8858abc6c04aada0c131da5	Guild4	Megasphaera	Firmicutes	0.013010861	ASV41
748c5f14c648b743ad9e4b5ebfd761ff	Guild4	Bacteroides	Bacteroidetes	0.008982659	ASV42
173c825a18291f2ba18b5e46d9db8408	Guild4	Parabacteroides	Bacteroidetes	0.002899449	ASV43
27f098662c8f87b4aaff903d9c0f28d3	Guild4	Bilophila	Proteobacteria	0.00241745	ASV44
8624322ae2483faffff421f46f814fc2	Guild4	Pyramidobacter	Synergistetes	0.001151401	ASV45
ed66913c64e880ea0b4cf78c59280664	Guild4	Desulfovibrio	Proteobacteria	0.000993797	ASV46
66063c3a2e5cc0fd8b0b301cdbb7c0ce	Guild4	Tyzzerella	Firmicutes	0.000328586	ASV47
24cccadb274fef1f88113e8f019e2a63	Guild4	[Eubacterium] nodatum group	Firmicutes	0.000124718	ASV48
ef1243f7818d1e35c1e968e8539173cf	Guild4	Anaerotruncus	Firmicutes	0.000102676	ASV49
5f8d35126b0a7321413d21487ec72b73	Guild4	[Clostridium] innocuum group	Firmicutes	8.03E-05	ASV50
824b9c5e131ccef5ab3286cfde31640c	Guild5	Bacteroides	Bacteroidetes	0.00278838	ASV51
d611e03044e800bf023cfc92bec2106a	Guild5	Butyricimonas	Bacteroidetes	0.000201487	ASV52
0e946c1f69e5e0d9ad68a03b2bf71d81	Guild5	Parasutterella	Proteobacteria	0.000200868	ASV53
7823f3affa1ac6029e82f4561502cb4e	Guild5	Prevotellaceae Ga6A1 group	Bacteroidetes	0.000144461	ASV54
d56fd4c21346f6d694c7e638f6bdcb70	Guild5	uncultured	Firmicutes	0.000120194	ASV55
596db8ce20ae5cbfdc338a62f1bed1d9	Guild5	Ruminiclostridium 5	Firmicutes	6.92E-05	ASV56
f98dab4619ff896a9cee45a9b9ddc792	Guild5	uncultured	Firmicutes	4.91E-05	ASV57
12acef5e0eba782d45fe9f038a99bf7b	Guild5	Candidatus Soleaferrea	Firmicutes	3.22E-05	ASV58
ff376ce6067337dd934fdebe0a3cf2eb	Guild5	Acetanaerobacterium	Firmicutes	1.62E-05	ASV59
47c305878bfc73bfeda34b2ae1a82ee1	Guild6	Bacteroides	Bacteroidetes	0.024890979	ASV60
df37b62e507e32409df4885c1ad2ce14	Guild6	Bacteroides	Bacteroidetes	0.011524776	ASV61
edf42f357f8934fc004c334c5c7b5e6e	Guild6	Paraprevotella	Bacteroidetes	0.000770336	ASV62
df8456a1abbfb4c8a2c450b44378d4cb	Guild6	Actinomyces	Actinobacteria	0.000233544	ASV63
e02967778c804350d971534895b0dfae	Guild6	uncultured	Firmicutes	0.000172101	ASV64
8e493df449e84acb4e24f5811ff3599b	Guild6	Solobacterium	Firmicutes	4.52E-05	ASV65
7a3090aa682623800304aca0da51c22c	Guild6	Faecalitalea	Firmicutes	4.20E-05	ASV66
d0833ce681acbdd482a03911d447756d	Guild6	Ruminococcaceae UCG-003	Firmicutes	3.62E-05	ASV67
a72bc4bbcb65e31aac628de5b9a9b0af	Guild6	Christensenellaceae R-7 group	Firmicutes	3.56E-05	ASV68
f9612c98d79c37a803a973724113cef6	Guild6	Atopobium	Actinobacteria	2.58E-05	ASV69
6616dc3bd6aeb3e26b9b2218290ea574	Guild7	Bacteroides	Bacteroidetes	0.023606067	ASV70
878fc53ff6f6333a92673b700951fa1b	Guild7	Parabacteroides	Bacteroidetes	0.004913825	ASV71
ef6a209f17415ca40777d4f3e0459647	Guild7	Bacteroides	Bacteroidetes	0.004018954	ASV72
d9eea834805833ac21a86fdabedd8556	Guild7	Ruminococcus 1	Firmicutes	0.002666811	ASV73

bc7f3f3f3af5b386336ef880486cf17d	Guild7	Paraprevotella	Bacteroidetes	0.001310748	ASV74
03c51b5e257b8e286445b07fe1e7d80b	Guild7	Dialister	Firmicutes	0.000791739	ASV75
647405386797efbb1366386613a6cac8	Guild7	Bacteroides	Bacteroidetes	0.000353281	ASV76
a22adf9cea194889c66a0e9b28768b1b	Guild7	Paraprevotella	Bacteroidetes	0.000311682	ASV77
712e740d6178a192f16d1f3413bd953e	Guild7	Bacteroides	Bacteroidetes	0.000226677	ASV78
d64daeafe88fb6fb606e8c7b4f14dbdc	Guild7	unclassified	Bacteroidetes	0.000132779	ASV79
52573485a4521e007e4c5217fc5d7c68	Guild7	uncultured	Firmicutes	7.52E-05	ASV80
47d2f0adc9e8063c45ed211dfd73fd63	Guild8	Fusobacterium	Fusobacteria	0.002505669	ASV81
b9bd4ddc71b5261571e53f606451241d	Guild8	Fusobacterium	Fusobacteria	0.001587956	ASV82
6dcaf5dafe4d20b4f82131ef8c1e5b19	Guild8	Mitsuokella	Firmicutes	0.001721031	ASV83
6e57b32b545e72b802822e1554c70868	Guild8	Lachnoclostridium	Firmicutes	0.000891326	ASV84
cd9401a6bce4a63af516d06d2a843f9d	Guild8	Veillonella	Firmicutes	0.000812889	ASV85
4d72007c70f4abc0bf9e8fe8b826d07d	Guild8	unclassified	Proteobacteria	0.000755028	ASV86
2740cf2417c92847cc298cbd71dd1fcd	Guild8	Veillonella	Firmicutes	0.000483931	ASV87
15701fcbecf8b280718656e482ad0021	Guild8	Selenomonas	Firmicutes	0.000288457	ASV88
03633857f8307d261e79828c0f213201	Guild8	Acidaminococcus	Firmicutes	0.000270588	ASV89
bb7e5c6df18c100b97f890fc35eb2911	Guild8	Proteus	Proteobacteria	0.00015423	ASV90
ba64f4fa502258a89d2b7468af3cbb28	Guild8	Candidatus Stoquefichus	Firmicutes	5.29E-05	ASV91
945184b6386c192c0066e0a98a154780	Guild9	unclassified	Proteobacteria	0.030160453	ASV92
e2cfe099d4a73a03ac5f80d4f8369dbc	Guild9	Ruminococcaceae NK4A214 group	Firmicutes	0.003894184	ASV93
3b94c19ef326eea0918e1c3d244c999c	Guild9	unclassified	Proteobacteria	0.001546794	ASV94
6db169339ee5897780e713c44784d74a	Guild9	Ruminococcaceae UCG-014	Firmicutes	0.00047865	ASV95
822a0ee849989f75cc532cb3540de19a	Guild9	Victivallis	Lentisphaerae	0.000485847	ASV96
3aad083a4c822d27071d1ff6163cbfb2	Guild9	uncultured organism	Tenericutes	0.000138627	ASV97
67da37a27427f41e9a28d4bcd418445b	Guild9	Comamonas	Proteobacteria	0.000140433	ASV98
35195a80f6fb69ef8bd6827d1b045aad	Guild9	Senegalimassilia	Actinobacteria	0.000129917	ASV99
b07ebea2f57176a62ecdc63193d8ffdf	Guild9	Victivallis	Lentisphaerae	6.62E-05	ASV100
ef2a1531066bf60397c9df449d532d2f	Guild9	uncultured	Firmicutes	3.81E-05	ASV101
523cbcc11a4bf59158f07d36cc967a60	Guild10	Prevotella 2	Bacteroidetes	0.002155031	ASV102
9fec7bdd6bd88e710bd69b15692e54a0	Guild10	Streptococcus	Firmicutes	0.001366919	ASV103
d2ed5fe0443294c72f4a4dd352e80e8c	Guild10	Bacteroides	Bacteroidetes	0.001210204	ASV104
1ab75f81f06d896defe1a2a976e99bcd	Guild10	Bacteroides	Bacteroidetes	0.001125706	ASV105
8538d07b8c1e8676f9b9c8c242aab14a	Guild10	[Eubacterium] coprostanoligenes group	Firmicutes	0.000760213	ASV106
7b28c20e72c6c95b3e604f0849245770	Guild11	Akkermansia	Verrucomicrobia	0.008158696	ASV107
609d1ae0045de753ac19f31803e3e19d	Guild11	Ruminococcaceae UCG-002	Firmicutes	0.002325249	ASV108
ab9899b443255cd34e51705c7b8ea201	Guild11	Roseburia	Firmicutes	0.001732372	ASV109
4bd01f90398375480101204bbcdd2ceb	Guild11	Ruminococcaceae UCG-005	Firmicutes	0.001722058	ASV110
55beed6d1a19ce34788b66488ffe3917	Guild11	uncultured	Firmicutes	0.00140943	ASV111
3219af75ee8056ed06ba81e873b0d0cb	Guild11	Ruminococcaceae UCG-002	Firmicutes	0.001139236	ASV112
51d4f17b8dda9f7d972bf3dd0ecc4e59	Guild11	Alistipes	Bacteroidetes	0.001080746	ASV113
b1f0b65b560701f74c4f43db4234d6c9	Guild11	[Eubacterium] coprostanoligenes group	Firmicutes	0.00108709	ASV114
26736c7ee02864e6bbab37424d69a4ed	Guild11	Christensenellaceae R-7 group	Firmicutes	0.00099236	ASV115
bea2245c01baedd6d4a6d6e4e857f9f6	Guild11	Butyricimonas	Bacteroidetes	0.000933266	ASV116
177db53ded58fcf2915778bff9a04f2c	Guild11	unclassified	Tenericutes	0.000830692	ASV117
43119e5f1eb41e9ee1776d7a6504126f	Guild11	Christensenellaceae R-7 group	Firmicutes	0.000607059	ASV118
1eaa3cb7baaac3220b0606e3621afd16	Guild11	Alistipes	Bacteroidetes	0.00028974	ASV119
0f603a9018080b298f6f858569d3b3e2	Guild11	Ruminococcaceae UCG-005	Firmicutes	0.000256437	ASV120
34b791a0a48d68bbf6278d510ee36430	Guild11	Ruminococcaceae UCG-010	Firmicutes	8.89E-05	ASV121
b1162bcb5e03ffb9e3911aec18b6f761	Guild11	Christensenellaceae R-7 group	Firmicutes	7.31E-05	ASV122
9d88acce7b2f13b58bf95d2f17ec8c2f	Guild12	Bacteroides	Bacteroidetes	0.002124354	ASV123
8795c5a86013864dc11f24b89e8cdd8c	Guild12	Alistipes	Bacteroidetes	0.001608469	ASV124
90af3675c266d09bb7986147ac5885ec	Guild12	Christensenellaceae R-7 group	Firmicutes	0.001447886	ASV125
4102c0c9284b080a36ae312a7b497915	Guild12	Desulfovibrio	Proteobacteria	0.001337947	ASV126
c2da7cc0247443dccd2e3d6a62f7be45	Guild12	Ruminococcaceae UCG-005	Firmicutes	0.000881285	ASV127
df14354ec277348c54d0c9dd49eb4da0	Guild12	Ruminiclostridium 6	Firmicutes	0.000649228	ASV128
84d5a713b1d46f0e0e0f7ba784e9925d	Guild12	Prevotellaceae NK3B31 group	Bacteroidetes	0.000327033	ASV129
d46e2205f0c6ecf67b51f83d111c509c	Guild13	Escherichia-Shigella	Proteobacteria	0.023074445	ASV130
44396f15b10f6577d61a11c6047c939f	Guild13	Methanobrevibacter	Euryarchaeota	0.000284505	ASV131
3525e8f55cd3355cc02bb9df648623e2	Guild13	Blautia	Firmicutes	0.000233381	ASV132
664667183a91ac47de4771fa5456a698	Guild13	[Ruminococcus] torques group	Firmicutes	0.000135099	ASV133
edd418053a744b62aa296a5a85039b3a	Guild13	Hydrogenoanaerobacterium	Firmicutes	0.00010619	ASV134
feb1c288cd46f6fd9bc3c2c35970cbdf	Guild13	Lactobacillus	Firmicutes	9.89E-05	ASV135
90542f89e5caf72027f06114dc95a815	Guild13	Family XIII AD3011 group	Firmicutes	5.53E-05	ASV136
45d4fe3f6c01f77c2dfc11dd91d3359a	Guild14	Lactobacillus	Firmicutes	0.000112626	ASV137
39c746e6698d4935484f50bf45508965	Guild14	Ruminococcaceae NK4A214 group	Firmicutes	8.57E-05	ASV138
1a7a9d14747af9f14fcdabdef2549a77	Guild14	Ruminiclostridium 9	Firmicutes	7.16E-05	ASV139
d515ae0a0b58aa13869a5966a80c98a9	Guild14	unclassified	Firmicutes	5.49E-05	ASV140
1900e8aeb6400849e6294cf82af2180d	Guild14	uncultured	Bacteroidetes	4.79E-05	ASV141
59777186ad2e0947e97615b5d6225136	Guild15	Faecalibacterium	Firmicutes	0.008324291	ASV142
f5f5e0da89730462abaf6301a9557193	Guild15	Faecalibacterium	Firmicutes	0.006284361	ASV143
c48070f3061b086b60ff32f77e0002fa	Guild15	Subdoligranulum	Firmicutes	0.003340406	ASV144
f540f77a6570daea199d86095c3e8c49	Guild15	[Eubacterium] eligens group	Firmicutes	0.002219275	ASV145
6851a4ee264b56be2fff4686ce269907	Guild15	[Eubacterium] hallii group	Firmicutes	0.000719167	ASV146
fec2da4ccc40b9ad85a020553063366b	Guild15	Oxalobacter	Proteobacteria	0.000142743	ASV147
fd44d4cb468fd7dc9b3227867714ed87	Guild16	Bacteroides	Bacteroidetes	0.008432599	ASV148

ab4040fe8717efa3f115680ae82cd72a	Guild16	Roseburia	Firmicutes	0.004182117	ASV149
45c80843dac191f771b11018ff90d94c	Guild16	Ruminococcus 1	Firmicutes	0.00071739	ASV150
547ed9d63f007845c48db64ef3e5aab6	Guild16	Bacteroides	Bacteroidetes	0.000574804	ASV151
ab149525cad479f6599c7923acc49691	Guild16	Lachnospiraceae UCG-010	Firmicutes	0.000377355	ASV152
65990c74559e4adfaa672a884ad7d464	Guild16	Coprococcus 1	Firmicutes	0.000302211	ASV153
a5a4b4d58bdd795871acf39629c9e584	Guild16	uncultured	Firmicutes	0.000297015	ASV154
62c87f13735bb22f5f7a98cfaa429f6c	Guild16	Ruminococcaceae UCG-014	Firmicutes	0.000152762	ASV155
9f1c7313b24bc98241173ae7db699bd0	Guild16	Moryella	Firmicutes	0.000142105	ASV156
b204a17b1a7f29074d81303d5ca68464	Guild16	Phocea	Firmicutes	7.21E-05	ASV157
c3337d877a02fb500fc37be8aa4fd234	Guild16	Family XIII AD3011 group	Firmicutes	6.64E-05	ASV158
9e1f282914b07cd79711ce21e7cf4aff	Guild17	[Eubacterium] eligens group	Firmicutes	0.000586039	ASV159
b621d4b3cca1ec89ceca12efca8a94fb	Guild17	Christensenellaceae R-7 group	Firmicutes	0.000287735	ASV160
ad139328ba8ac39c2e5ccef543df2deb	Guild17	Ruminiclostridium 5	Firmicutes	0.00016488	ASV161
218c409a1b9b9671bbd802d1f2ccb662	Guild17	Parasutterella	Proteobacteria	0.000142592	ASV162
53bf77df9214c54ded7332ff0a9cac2d	Guild17	Coprococcus 3	Firmicutes	9.78E-05	ASV163
a1642ee93cbc5a4d6b2816c0a116cf42	Guild17	Ruminococcaceae UCG-010	Firmicutes	4.83E-05	ASV164
2102f51f3591341a5e15fb5958ee321c	Guild17	Family XIII UCG-001	Firmicutes	3.18E-05	ASV165
3723ea6e099f53017ca85c153c80969f	Guild17	uncultured	Firmicutes	2.52E-05	ASV166
d8e7f9181e4d5a4a6453e9ca9996a43f	Guild18	Parabacteroides	Bacteroidetes	0.009526419	ASV167
77c2dc197e6b3dbebc4ee240c6a1c559	Guild18	Oscillibacter	Firmicutes	0.000568277	ASV168
662a40a2523f7669fa865700fc74a1cf	Guild18	uncultured	Firmicutes	0.000545648	ASV169
5ca4ec70e0319aa4e6e75595df17d8dc	Guild18	Ruminococcaceae UCG-005	Firmicutes	0.000349171	ASV170
8646bffa210e3f061cc57f1b1cf2f751	Guild18	Hungatella	Firmicutes	0.000240514	ASV171
7c3d7711fd15460714ece118b35c0576	Guild18	Anaerotruncus	Firmicutes	0.000158996	ASV172
6f980c6f3f2ffefd2013c721461cc1c6	Guild18	Holdemania	Firmicutes	0.000117673	ASV173
5eeda5ba5e347d3ee4cc5d928bc3a708	Guild18	Coprobacter	Bacteroidetes	8.83E-05	ASV174
3120660c883750285d85c12f7d898f67	Guild18	Oscillibacter	Firmicutes	7.86E-05	ASV175
08bd43071a06abf56df235fca6655bea	Guild19	[Ruminococcus] torques group	Firmicutes	0.003326741	ASV176
11b7d5a0dd0446562be236a4a9144c75	Guild19	Ruminiclostridium 9	Firmicutes	0.00050617	ASV177
64cdad777808dde2fad9b1be7bc91b85	Guild19	uncultured	Firmicutes	0.000452271	ASV178
a5af97c864a48da5a31864d5382cea20	Guild19	uncultured	Firmicutes	0.000116903	ASV179
f7870a4e6cccb8a029cf6f0091c106f5	Guild19	Faecalitalea	Firmicutes	8.93E-05	ASV180
a1a2a4326524ddcb86d54831586052d2	Guild19	Slackia	Actinobacteria	3.38E-05	ASV181
6928971040e6116b9a3f5c9c9bf41adb	Guild20	Bacteroides	Bacteroidetes	0.014869406	ASV182
25394a604bbcd078c80186b300dab8ee	Guild20	Holdemanella	Firmicutes	0.003033723	ASV183
8af49bc8a804f6d85ca5fa00a7c9dda8	Guild20	Dialister	Firmicutes	0.002671251	ASV184
243a590b7a6c17e5a16dff4bc7033ac1	Guild20	Bacteroides	Bacteroidetes	0.002255181	ASV185
8135beec331a8d558070c38b580e4b8e	Guild20	Ruminococcus 2	Firmicutes	0.002044124	ASV186
0dcf5023e96fb8175d7bf02d900c9dd1	Guild20	[Eubacterium] coprostanoligenes group	Firmicutes	0.001740776	ASV187
6bbff26a22040581ff0e24f9b5b40830	Guild20	Acidaminococcus	Firmicutes	0.001423184	ASV188
a63c438fdf54dab3760eef280c5b4fc3	Guild20	unclassified	Proteobacteria	0.001388713	ASV189
a1580b985a39db6e693626a2e4f28d6d	Guild20	Dialister	Firmicutes	0.001363949	ASV190
fd444765f4a6858dfc7607a449a30c31	Guild20	Bacteroides	Bacteroidetes	0.000491521	ASV191
69b90707f6f3da7941026fbe6a1597ce	Guild20	Lachnospiraceae FCS020 group	Firmicutes	0.000140385	ASV192
7b2963eb465aa949c1fc339505286dff	Guild20	Parasutterella	Proteobacteria	0.000128322	ASV193
3b010dbd9eae72a79f11af5d24eb3ca3	Guild21	Akkermansia	Verrucomicrobia	0.006099036	ASV194
3f49f2e33639f81618c76b5807f6498c	Guild21	[Eubacterium] coprostanoligenes group	Firmicutes	0.002023251	ASV195
e603a72ebbc7a4fcb5a657b52c3e8c50	Guild21	Azospirillum sp. 47_25	Proteobacteria	0.001196139	ASV196
241a0c90cc40da29bc738d34b607f25f	Guild21	Lachnospira	Firmicutes	0.000956144	ASV197
620e0644e80653b6c5ae2d4eec1e8d73	Guild21	Lachnospira	Firmicutes	0.000840895	ASV198
9c148227a7c018a242f0a0a892280592	Guild21	uncultured bacterium	Tenericutes	0.000862443	ASV199
cc310148d41118668670367fc570ab7b	Guild21	Subdoligranulum	Firmicutes	0.0004793	ASV200
fee421dd182111e1d718ac10decab1e9	Guild21	Alistipes	Bacteroidetes	0.000453207	ASV201
13e5e67a2599db5c3cdfa82c4c9ea6fd	Guild21	unclassified	Firmicutes	0.000458373	ASV202
b98ddb5dd95e2e10787a8f4f6ecb511c	Guild21	Sutterella	Proteobacteria	0.000337891	ASV203
e6a9c4991e5e11cd234438b7f57a351d	Guild21	Ruminococcus 2	Firmicutes	0.000240184	ASV204
73cd1c10175eb846badd2f34bd86dd08	Guild21	Intestinimonas	Firmicutes	0.000179645	ASV205
a760b9db6c42c5ae5f26045515bd9575	Guild21	Alistipes	Bacteroidetes	0.000177547	ASV206
c95c5a9f7b563d35d8b87a1429acffcc	Guild21	Alistipes	Bacteroidetes	0.000165767	ASV207
7c104699e795bbf45f90fceac906e6b4	Guild21	Christensenellaceae R-7 group	Firmicutes	0.000148229	ASV208
7fd072aff74564746d11ca0c54ee6ae2	Guild21	gut metagenome	Tenericutes	7.21E-05	ASV209
d46f7bf5556893b1a52f835991191722	Guild22	uncultured bacterium	Tenericutes	0.001833779	ASV210
8321090e9dde2fd78476c7c70ac50a32	Guild22	gut metagenome	Bacteroidetes	0.001565087	ASV211
be22632d5e3f6175707a3213d7a90ad2	Guild22	Dialister	Firmicutes	0.001578941	ASV212
a2d300d1e9c621dbc5239cc092d433c3	Guild22	Ruminococcaceae UCG-014	Firmicutes	0.000617652	ASV213
4d3f1d8f02b45e845e419a273f5b2386	Guild22	Ruminococcus 1	Firmicutes	0.000260209	ASV214
26c6e3ed14694582e207478c1f23c21f	Guild22	Turicibacter	Firmicutes	0.000156257	ASV215
2489471b3729e885a2c1ddaa10774d8e	Guild22	Negativibacillus	Firmicutes	0.00012834	ASV216
d084475ce17d67557615ecfb105797ab	Guild22	Ruminococcaceae UCG-010	Firmicutes	0.000124201	ASV217
e147f17187ee6e0d8e84bdd370bfb053	Guild23	Lactobacillus	Firmicutes	0.000664543	ASV218
18812bd61aeb2cea2081498cfa9ee3c8	Guild23	Parabacteroides	Bacteroidetes	0.000559983	ASV219
63878a679588dceb5219d4130965ef79	Guild23	Tissierella	Firmicutes	0.000377837	ASV220
0e4ad48a378cee2414e2d8543e734ffd	Guild23	Parabacteroides	Bacteroidetes	4.81E-05	ASV221
be44bdb8fa2c3b45178a5579d3ed9684	Guild24	Lachnospiraceae NK4A136 group	Firmicutes	0.001920522	ASV222
02db09efa7c46a21be5fadc51cb17dd8	Guild24	Ruminococcaceae UCG-014	Firmicutes	0.001117741	ASV223

67e01a89cf3eabe01c14f7f937534cf4	Guild24	[Eubacterium] coprostanoligenes group	Firmicutes	0.000882618	ASV224
da03da1eef1147967c5046fa9f330630	Guild24	Ruminococcaceae UCG-014	Firmicutes	0.000845442	ASV225
45ddc69a3fbf19ff4953211c157cb9ac	Guild24	Prevotella 7	Bacteroidetes	0.000314919	ASV226
dcd74b6107061fda983a7765c1f93191	Guild24	Sutterella	Proteobacteria	0.000285661	ASV227
c97ea25ba069877eac769cb05968724c	Guild24	Ruminococcaceae UCG-014	Firmicutes	0.000237176	ASV228
81b0af5710b83eca45bef65a75f7794f	Guild24	Christensenellaceae R-7 group	Firmicutes	0.00023005	ASV229
5eff6071b9b7dd4c9a9edaeba059f650	Guild24	Ruminococcus 1	Firmicutes	0.000100166	ASV230
3114d42cc9f862a2a18d7e5efde7fa9b	Guild24	Slackia	Actinobacteria	4.93E-05	ASV231
43a39fa4815bda79d6470002c8172dcb	Guild25	Barnesiella	Bacteroidetes	0.001748893	ASV232
3aff0c61231473669fac5d530978d704	Guild25	gut metagenome	Proteobacteria	0.00160175	ASV233
b6aa2186be5e7c7aca2bca54ff8390fc	Guild25	metagenome	Tenericutes	0.000504338	ASV234
a2217cc8df6a4c209aee7f31a304c2b0	Guild25	Ruminococcaceae NK4A214 group	Firmicutes	0.000502256	ASV235
6f532f9a41acff6f812a4827a62219ac	Guild25	Ruminococcaceae UCG-014	Firmicutes	0.000367998	ASV236
56c77f7e4161cea89f967b42a4dd55ad	Guild25	Ruminococcaceae UCG-014	Firmicutes	0.000278292	ASV237
74a6b2d8faae4662532b0e4123964dac	Guild25	uncultured bacterium	Tenericutes	0.000242601	ASV238
65a80de0631a0b02eedb8c80c61c7df7	Guild25	Eisenbergiella	Firmicutes	0.000174717	ASV239
9db6065dfa5f306778c1a370f3267d73	Guild26	Bacteroides	Bacteroidetes	0.009898815	ASV240
4b7712254e4dd8d5bc585d4a6c5c2e88	Guild26	Fusobacterium	Fusobacteria	0.008800915	ASV241
08a0cf376add32f3f4b57ec1e82a7a27	Guild26	Megamonas	Firmicutes	0.008500721	ASV242
b20abc2c16ea643f1fe7ceb2e5db9d45	Guild26	Sutterella	Proteobacteria	0.003481351	ASV243
66ae7eb5844f42c118ee1090c85c83b5	Guild26	Megasphaera	Firmicutes	0.002260199	ASV244
96cf4779be686036f92ee15e58b72d52	Guild26	Phascolarctobacterium	Firmicutes	0.001583021	ASV245
6fe33250c45ddbda2700682431270ddc	Guild26	Sutterella	Proteobacteria	0.001174153	ASV246
1619b48906d097d8c3a8af893250385b	Guild26	uncultured organism	Firmicutes	0.000801506	ASV247
16530e5b50b780018210dac05a471715	Guild26	Ruminococcaceae UCG-002	Firmicutes	0.000711364	ASV248
412b0b2c43975195a230e493a7a2b763	Guild26	Barnesiella	Bacteroidetes	0.000383767	ASV249
9484afed9f64641667938e294c07fe7c	Guild26	Roseburia	Firmicutes	0.000355257	ASV250
0b886492b5eaa91693cc62f74710b18b	Guild26	Lachnoclostridium 12	Firmicutes	0.000348346	ASV251
b6ecd20f301035741915d6c32bda2410	Guild26	Allisonella	Firmicutes	0.000229418	ASV252
656fc22d077cc5d6ef83285bcd081cbe	Guild26	gut metagenome	Proteobacteria	0.000183298	ASV253
f1eb372635240322c36cc8d43ad0b70f	Guild27	Prevotellaceae NK3B31 group	Bacteroidetes	0.000976453	ASV254
4c974b834afe158c4265402ee0bfcbad	Guild27	uncultured Porphyromonadaceae bacter	Bacteroidetes	0.000407983	ASV255
465b59fcd7a05f0ca2cc7c41e8816fbf	Guild27	Coprococcus 2	Firmicutes	0.000359601	ASV256
e51fc3bc6d08ae82b3299bff3739b1f6	Guild27	metagenome	Bacteroidetes	0.00030586	ASV257
fa7c29d4c4e6a6262f4e41394dfbd4b2	Guild27	Ruminococcaceae UCG-014	Firmicutes	0.000255206	ASV258
86ff0efd7fd88b82809500c90c4a7832	Guild27	Faecalibacterium	Firmicutes	0.000168777	ASV259
afa3e197ab31aa17a760b9a076eb42f3	Guild27	Ruminococcaceae UCG-005	Firmicutes	0.000123422	ASV260
caee94af7a4562c61da6712807157dc9	Guild27	[Eubacterium] coprostanoligenes group	Firmicutes	0.000119389	ASV261
4876553b9fed8291dbf1c63b0ac0886a	Guild27	gut metagenome	Firmicutes	4.54E-05	ASV262
66b90e64bc0857f1f29cb63d373fcff0	Guild28	unclassified	Firmicutes	0.000707786	ASV263
605cc047d6200f294e043f8ce6cb5af7	Guild28	Coprobacter	Bacteroidetes	0.000704842	ASV264
078e0fd51a7d5e5b908328591895ba58	Guild28	gut metagenome	Proteobacteria	0.000546298	ASV265
f4bace87750c8c5b7697f61826634acb	Guild28	unclassified	Firmicutes	0.000267877	ASV266
6b5c5532185593c41966b2173ea9df1c	Guild28	unclassified	Firmicutes	0.000260122	ASV267
a63f65ad0f7a1ffc9e5ea828e95cdd6b	Guild28	Blautia	Firmicutes	0.000136178	ASV268
9a76c2c2904fcae623e236b6818a712e	Guild29	Parabacteroides	Bacteroidetes	0.001063516	ASV269
fd070ebbe12a220527ab068265c06df8	Guild29	Lachnospiraceae NK4A136 group	Firmicutes	0.000531253	ASV270
a2d06d806140a44fbc74faa7452852b8	Guild29	Acidaminococcus	Firmicutes	0.00048893	ASV271
1595757c017000381cfdfdf769029685	Guild29	Prevotella 2	Bacteroidetes	0.000379498	ASV272
afef966c3ddc1ba9f8f1452439b724db	Guild29	Rikenellaceae RC9 gut group	Bacteroidetes	0.000388312	ASV273
f8d1b6567bca78638227d413f6170e06	Guild29	Ruminococcaceae UCG-002	Firmicutes	0.000248265	ASV274
f646c254e568a1126ec17324cc80ad69	Guild29	uncultured bacterium adhufec202	Tenericutes	0.000242911	ASV275
deba3ff7f7e05cdf6e1c4c8e5aa22d6d	Guild29	Ruminococcaceae UCG-014	Firmicutes	0.000232634	ASV276
a8b4b0d58275413b83f3fe9986834bb9	Guild29	Ruminococcaceae UCG-005	Firmicutes	0.000222301	ASV277
4f480409cf34da6fb985eaa1cf33a65b	Guild29	Lachnospiraceae NK4A136 group	Firmicutes	0.000136219	ASV278
274d44a105684512d6df0a983a5b6663	Guild29	Ruminococcaceae NK4A214 group	Firmicutes	0.000134877	ASV279
9e818341877554614cc206109df99d5d	Guild29	Ruminococcaceae UCG-010	Firmicutes	0.000114695	ASV280
305add6c79f7766e1c01a4060f3fe428	Guild29	Ruminococcaceae UCG-010	Firmicutes	0.000108814	ASV281
a333ee090a52b582e1a0e2dd776eb937	Guild29	Coprobacillus	Firmicutes	7.54E-05	ASV282
675c847bccbc53942ebb7b8cbb4efc4d	Guild30	Prevotella 9	Bacteroidetes	0.029940351	ASV283
333bbf9224442fc10cd497377d2b1d01	Guild30	Bacteroides	Bacteroidetes	0.011446325	ASV284
263e41ea2c25dc87bd2ff5910cf30d40	Guild30	Agathobacter	Firmicutes	0.010612603	ASV285
da6a7cba87e0895b9cbe6037b9bd8b3b	Guild30	Prevotella 9	Bacteroidetes	0.00613934	ASV286
b4d07c2a22af0c99c73cf9722200d878	Guild30	Lachnospiraceae NK4A136 group	Firmicutes	0.001852283	ASV287
fc00ed40b15cd5fa384b672502a3adc3	Guild30	Agathobacter	Firmicutes	0.001204117	ASV288
5322567d7de1d94e6cf65390deb29b1c	Guild31	[Eubacterium] eligens group	Firmicutes	0.004168197	ASV289
84f9cf6d08ccc7adb4e945c4fd8be494	Guild31	Ruminococcaceae UCG-002	Firmicutes	0.004278677	ASV290
dbcdaca3ee9e9fe75d8829e87b8551dc	Guild31	Sutterella	Proteobacteria	0.003084825	ASV291
9e3226efaf559eb14a478f880462cb8a	Guild31	[Ruminococcus] torques group	Firmicutes	0.001719669	ASV292
208c0c43c7c5d4a6bb549e0c8365cd21	Guild31	[Eubacterium] eligens group	Firmicutes	0.001663115	ASV293
b726627e18f29e0da5289f81ca294f92	Guild31	Subdoligranulum	Firmicutes	0.000869265	ASV294
396ffa76286336a3eeb1ea420e60f86b	Guild31	Lachnospiraceae UCG-001	Firmicutes	0.0007593	ASV295
c6b90711837508687841b1d5cbff2f65	Guild31	uncultured	Firmicutes	0.00041171	ASV296
6c324279c1fc2965e594486ac26b0548	Guild31	Coprococcus 2	Firmicutes	0.000344182	ASV297
bdb6ddef008a6d31c019642fb28d7c1d	Guild31	Ruminiclostridium 9	Firmicutes	0.000188263	ASV298

ee72ee97db064e5d745356f479b9e576	Guild32	Ruminococcus 2	Firmicutes	0.004174206	ASV299
5669f9055e393154386d3ad1196d3f03	Guild32	Alistipes	Bacteroidetes	0.004019053	ASV300
9a91e2e1c9d7c179afb558a3fd4664fd	Guild32	Ruminococcus 1	Firmicutes	0.002463711	ASV301
9192b3275c36e56540678451adfa601f	Guild32	Ruminococcaceae UCG-002	Firmicutes	0.002064527	ASV302
782a79a0725adc6ac22fc1fd15933a12	Guild32	Holdemanella	Firmicutes	0.001332578	ASV303
07fd92a29ce27a31f4d95c6821595530	Guild32	Lachnospiraceae UCG-001	Firmicutes	0.001038485	ASV304
5f25904e073fe5c114cadfde74349518	Guild32	Lachnospiraceae UCG-004	Firmicutes	0.000988448	ASV305
ed0b2c436e0aa37c7501df9927eca476	Guild32	Sutterella	Proteobacteria	0.000884434	ASV306
315ca0a729f126b941ba111a16d4d97a	Guild32	Bifidobacterium	Actinobacteria	0.000774013	ASV307
acf339d6620e975d91dc57db41e28cae	Guild32	Paraprevotella	Bacteroidetes	0.00054377	ASV308
0b53415990b1e4435ba981cc617f6be0	Guild32	Lachnospiraceae UCG-003	Firmicutes	0.000472082	ASV309
23d6e67ff377b63cc8506920f662b5a5	Guild32	Anaerosporobacter	Firmicutes	0.000409902	ASV310
fcffedae608fe4871be42936a1cb523a	Guild32	[Ruminococcus] torques group	Firmicutes	0.000188053	ASV311
1b5d50e4ae6a2cb153f3b2ca04699419	Guild32	[Eubacterium] ventriosum group	Firmicutes	0.000149152	ASV312
74d519816d4cfb9cf4ec9605f76301d6	Guild32	Family XIII UCG-001	Firmicutes	0.000100539	ASV313
21401b105dc53b367b8fdc0ec97a9f02	Guild32	Sutterella	Proteobacteria	0.000104234	ASV314
1b9ab7ba8573cbfe5f1a9f59f0b38ff4	Guild32	Adlercreutzia	Actinobacteria	9.04E-05	ASV315
69310a7cff9f6b902cdfd08243784ace	Guild32	unclassified	Firmicutes	4.36E-05	ASV316
410e1eaa1468a3d40595898f671ba0b3	Guild33	[Eubacterium] coprostanoligenes group	Firmicutes	0.007063397	ASV317
c66ec67719e54d2cb0afc3154b3c23af	Guild33	Christensenellaceae R-7 group	Firmicutes	0.002057393	ASV318
b1d8e09561b600103cc3d8b388c02478	Guild33	unclassified	Firmicutes	0.001609757	ASV319
d0043999672f950cf76f8077a5f4824a	Guild33	unclassified	Tenericutes	0.000421939	ASV320
a55a010c9525ce2943a553dca1421b1c	Guild33	[Eubacterium] ventriosum group	Firmicutes	0.000361642	ASV321
d969ff7a11c25dc9b04c6eb48a6ec8c3	Guild33	[Eubacterium] coprostanoligenes group	Firmicutes	0.000301574	ASV322
69659d7edd60cd37bbdc9e3bb7f77431	Guild33	Collinsella	Actinobacteria	0.00022987	ASV323
c0a8f5074b73b0648d1a21b1f3d8ffaf	Guild33	Ruminococcaceae NK4A214 group	Firmicutes	0.00019634	ASV324
2f9bd2fa5649f173fc8c80d1cdb2d633	Guild33	Ruminococcus 1	Firmicutes	7.11E-05	ASV325
2c982937754e6321f861027032db80f7	Guild34	Bacteroides	Bacteroidetes	0.006709393	ASV326
aae4c4ed528ae16ec417df816699cfdb	Guild34	Ruminococcaceae UCG-005	Firmicutes	0.003313781	ASV327
0b4aeb45916a24fd10e3655fe6481966	Guild34	Christensenellaceae R-7 group	Firmicutes	0.00145379	ASV328
7ea6aefb9b9611299846da49e41ff0ea	Guild34	Bacteroides	Bacteroidetes	0.001221535	ASV329
35ffcc3b809d667286737d79670b8de5	Guild34	Bifidobacterium	Actinobacteria	0.001078692	ASV330
787ab1672520289680427986b59e3417	Guild34	uncultured organism	Firmicutes	0.000626528	ASV331
41b9fa65a5137bfae1255b4de63be427	Guild34	[Eubacterium] ventriosum group	Firmicutes	0.000614531	ASV332
29872bd95f441262d1a646a94a47c8d7	Guild34	Paraprevotella	Bacteroidetes	0.000432787	ASV333
df1e3db8a1faf4439c34564e9698099a	Guild34	Coprobacter	Bacteroidetes	0.000177993	ASV334
ab38d51270cc78210521f35d22c4ebe1	Guild34	Sutterella	Proteobacteria	0.000150121	ASV335
4552af3e905206840cf8b67bcaf2693f	Guild34	unclassified	Proteobacteria	0.000128353	ASV336
5e50a007ea9a7f83275157c158916fcc	Guild34	Alistipes	Bacteroidetes	8.32E-05	ASV337
75bf5a0b24ffb76d3ba75ad1d75b7d23	Guild34	Ruminiclostridium 1	Firmicutes	5.86E-05	ASV338
9639a3291729a3758207b47715d9205f	Guild35	Subdoligranulum	Firmicutes	0.004396776	ASV339
e335f74033bc634af43ee6baa84fa247	Guild35	Romboutsia	Firmicutes	0.002497324	ASV340
2e4f2b53b856c4def6d021d01f5abb70	Guild35	Dorea	Firmicutes	0.001687321	ASV341
6420671c26eef32065e0275c8cd6701e	Guild35	Intestinibacter	Firmicutes	0.001216984	ASV342
35cbb315e019e0a8768d4b73862df415	Guild35	Collinsella	Actinobacteria	0.001173039	ASV343
00a96fbd0ac34bfd245f9c24f8737f7d	Guild35	Blautia	Firmicutes	0.00105455	ASV344
821130ba72e86c335c55c269f31db0d0	Guild35	Clostridium sensu stricto 1	Firmicutes	0.000138925	ASV345
394eda29c886632f514dd94b58381186	Guild36	Haemophilus	Proteobacteria	0.002197945	ASV346
421cbd1d71d34704c6d53377261e213c	Guild36	[Eubacterium] ventriosum group	Firmicutes	0.00089212	ASV347
9580aa8a52ec18a4e92e701cdb595faa	Guild36	Clostridium sensu stricto 1	Firmicutes	0.000735954	ASV348
7814c2b485cc601b885f117c70bbdf5d	Guild36	Ruminococcus 1	Firmicutes	0.000262209	ASV349
8d703908c09c7cb0da2a4398dd74f9f8	Guild36	Terrisporobacter	Firmicutes	0.000241888	ASV350
33ba975cdb88b5073a2355ffdf01e9cb	Guild36	Bifidobacterium	Actinobacteria	0.000214612	ASV351
22424eb53c259749d0d5cf4f45d18613	Guild36	Clostridium sensu stricto 1	Firmicutes	0.000132743	ASV352
fd2eb1fe2266eed160a855f715f7c77e	Guild36	Streptococcus	Firmicutes	9.03E-05	ASV353
9acb555d3c5dd77e9035a661e7ad1bb6	Guild37	Clostridium sensu stricto 1	Firmicutes	0.002748282	ASV354
dd918c333e5c4fef9c92068fddda1c3e	Guild37	Clostridium sensu stricto 1	Firmicutes	0.001642454	ASV355
40874d61006874f7c87d904707d320d9	Guild37	Clostridium sensu stricto 1	Firmicutes	0.001207652	ASV356
33518e48b174ac428cb7be1a216c6c6e	Guild37	Anaerostipes	Firmicutes	0.000807783	ASV357
caea322863a57bb2b14c7317fa120dc4	Guild37	Lachnospiraceae NK4A136 group	Firmicutes	0.000627532	ASV358
c83c84c8f82639c3bcb16c5630f257b2	Guild37	Dialister	Firmicutes	0.000585765	ASV359
be5cc85bd2ac16b6ae446fd24b1e2308	Guild37	Turicibacter	Firmicutes	0.000373881	ASV360
a82250af6ba5d2b64912c3ad6d84a5b0	Guild37	Cellulosilyticum	Firmicutes	0.000143713	ASV361
9e75d15ba3717ca20d8aa45e3c4a6bab	Guild37	Leuconostoc	Firmicutes	9.43E-05	ASV362
4d334598e144b1d31c0b92a301b5cc68	Guild37	Lachnospiraceae FCS020 group	Firmicutes	6.94E-05	ASV363
e220e84c750738e230e8ecfe97decc4c	Guild37	uncultured	Firmicutes	5.46E-05	ASV364
7e02d8db5f57fe608ac0d706fefb07c1	Guild37	Peptococcus	Firmicutes	4.82E-05	ASV365
6dfb118aa8b73ede1bdb71dedc23838c	Guild38	Holdemanella	Firmicutes	0.001943685	ASV366
bd5ef8f372e09bcf6ec35825be4f053c	Guild38	Coprococcus 3	Firmicutes	0.001295763	ASV367
0a68ec564e278b0246c920ad7de41880	Guild38	Blautia	Firmicutes	0.000843687	ASV368
aac0099991c8518a5268b1a19a84550b	Guild38	[Eubacterium] hallii group	Firmicutes	0.000492859	ASV369
7717c98118439762317647199c6005f7	Guild38	Ruminococcaceae UCG-005	Firmicutes	0.00036698	ASV370
d71feb1923fa288790187009e8efa6d3	Guild38	Blautia	Firmicutes	0.00021392	ASV371
ca94cbcccd3ab51b3784a6aed45a4a04	Guild38	Ruminococcus 2	Firmicutes	0.000168386	ASV372
b7e5e417da183df1d5cb33a51a10991d	Guild39	Bacteroides	Bacteroidetes	0.002574422	ASV373

8343cf4c26bb977e05e4d9c5125441ef	Guild39	Parabacteroides	Bacteroidetes	0.002074775	ASV374
1b92901eaf73176b86069084ac8c13e3	Guild39	Lachnospiraceae UCG-010	Firmicutes	0.001591868	ASV375
e4ef1c7b616eb842c0ff7a327e83bd76	Guild39	Parabacteroides	Bacteroidetes	0.001406916	ASV376
9282e20164736c7ada9bd98d04654b5e	Guild39	Sutterella	Proteobacteria	0.001252207	ASV377
36d9ad396b85196c9b4cf68b81789585	Guild40	Lachnospira	Firmicutes	0.017689732	ASV378
72ec78ecd4c30c1ddc64900630f35387	Guild40	Lachnospira	Firmicutes	0.005982381	ASV379
ec6732c2e0d4cf64b3d0350e7fe3defb	Guild40	Roseburia	Firmicutes	0.005655469	ASV380
601101d33cdf43a40bd674a50711af52	Guild40	Roseburia	Firmicutes	0.002931697	ASV381
1a9df3fbbc7fc8cecec4239cf801d2fc	Guild40	Ruminococcaceae UCG-013	Firmicutes	0.002819718	ASV382
4e2e6735331ad01a30c94d2f4c285dc2	Guild40	CAG-56	Firmicutes	0.00096005	ASV383
0243b68c76049aa1b8c51a20333da950	Guild40	Lachnospiraceae ND3007 group	Firmicutes	0.000878148	ASV384
2de0f958e30d26f04cba8d2a920bbe46	Guild40	Blautia	Firmicutes	0.000790493	ASV385
4ada3d8098e9706fcebb85f1e427fea6	Guild40	uncultured	Firmicutes	0.000492571	ASV386
6ec1023583647a401269990c4e8ad716	Guild41	Lachnospira	Firmicutes	0.012260939	ASV387
8e175abe6a746b8f33bae9cd7c8192bb	Guild41	Faecalibacterium	Firmicutes	0.009827043	ASV388
2c0c62ea09b2efe01bacdcbcf558d2b8	Guild41	Lachnoclostridium	Firmicutes	0.005969191	ASV389
c6c3ab4e828fb40d6e05967b7aac9338	Guild41	Blautia	Firmicutes	0.004840234	ASV390
119eea33cb9f505d6a20297d132e792a	Guild41	unclassified	Firmicutes	0.004569796	ASV391
bed671b4676144142cebd192b690f499	Guild41	Blautia	Firmicutes	0.004400591	ASV392
e865f29a716e8d51f83931befa951240	Guild41	Erysipelotrichaceae UCG-003	Firmicutes	0.003228018	ASV393
d76d59ec71de0e3b22da0c9cd564d41a	Guild41	Fusicatenibacter	Firmicutes	0.003179672	ASV394
a891fb44241e433fa8acf251ef4d328f	Guild41	unclassified	Firmicutes	0.002233087	ASV395
3f6dce06719bb11b6b9bbb8f2c8a5644	Guild41	Butyricicoccus	Firmicutes	0.001908869	ASV396
19715b1593c19aa6d35a4d1e3cd09374	Guild41	Lachnospiraceae UCG-004	Firmicutes	0.001693855	ASV397
f4cc6e4932f507b33d1335d801f54563	Guild41	Dorea	Firmicutes	0.001256197	ASV398
df16a09f3e448ddd3b1ec54066d3081d	Guild41	Butyricicoccus	Firmicutes	0.001192297	ASV399
a18c0c17fce3d52dc60506e8730bc996	Guild41	Butyricicoccus	Firmicutes	0.000199304	ASV400
96040114b5274bdb987f28c63b6b6b87	Guild41	Agathobacter	Firmicutes	0.000192799	ASV401
cf315874f2a721ae340f6dbf4ec0c2d6	Guild41	unclassified	Firmicutes	6.72E-05	ASV402
708b000c48dbefb1454b79108d2a1f70	Guild42	uncultured	Firmicutes	0.006080689	ASV403
c24e0e391aa836b5eae25567c7eb89ee	Guild42	[Ruminococcus] gnavus group	Firmicutes	0.004976167	ASV404
fd496fd32dc8c08ade2e8b6c9d8ee13d	Guild42	Streptococcus	Firmicutes	0.004547791	ASV405
7af6fc12809fdafab918881f836460f8	Guild42	Tyzzerella 4	Firmicutes	0.001075166	ASV406
edc9e5c16e40aff1eadce6597940f08f	Guild42	Streptococcus	Firmicutes	0.000898851	ASV407
da8644094f1d480f3b3a6a03a7027ab4	Guild42	Hungatella	Firmicutes	0.000368902	ASV408
6eaef2dd2e8097421a196c104a2b76c8	Guild42	unclassified	Firmicutes	0.000329192	ASV409
06f825b512d903b9230e1a55d87359ee	Guild42	Streptococcus	Firmicutes	0.000157847	ASV410
de68af3f62b2b04e42f894e18839bb57	Guild42	Dielma	Firmicutes	8.91E-05	ASV411
2bd9309f2f97cae51d18d06ea1ca519a	Guild42	Granulicatella	Firmicutes	8.24E-05	ASV412
e9d23f8839a91794229045bb0395ec02	Guild43	Bacteroides	Bacteroidetes	0.004869623	ASV413
0a035a7ec1f4a194dd7fbda3c5660db0	Guild43	Lachnoclostridium	Firmicutes	0.00340693	ASV414
db7f42d2a4ded289e177bb4c00edff61	Guild43	Lachnospiraceae NK4A136 group	Firmicutes	0.002684894	ASV415
91587a85e342f8dba27f54e15ac0ea77	Guild43	Tyzzerella 3	Firmicutes	0.000816969	ASV416
428c03e963c4173df9cfaee5ce693f6a	Guild43	uncultured	Bacteroidetes	0.00074863	ASV417
107273e73071282e4d0f4a0b46548da5	Guild43	Aeromonas	Proteobacteria	0.000544939	ASV418
e5524af3c73aa22f010506f232a774b1	Guild43	Roseburia	Firmicutes	0.000404815	ASV419
9624ce4cefb03530aaafca65b645cfcc	Guild43	Lachnoclostridium	Firmicutes	0.000371232	ASV420
959035a199d6135cc1731d5602c20f54	Guild43	uncultured	Proteobacteria	0.000374472	ASV421
0a3cf58d4ca062c13d42c9db4ebcbc53	Guild43	unclassified	Cyanobacteria	0.000203217	ASV422
85c44c83eddc5d3028261a1000b7d0e1	Guild43	Gemella	Firmicutes	0.000131745	ASV423
37f004671c936285c26471120596b2f5	Guild43	Sarcina	Firmicutes	9.22E-05	ASV424
d20b46e3c9d79a8e49a48f112fc03d4f	Guild43	Romboutsia	Firmicutes	8.81E-05	ASV425
db2c5f635cc676adc6fa4b1e7394fa74	Guild43	Taeseokella	Bacteroidetes	7.92E-05	ASV426
53d2351a74c69dd99f19a791c81a9370	Guild43	Epulopiscium	Firmicutes	3.28E-05	ASV427
4fba6fef450158c1bd1cbcb5a0e8f5e5	Guild44	Prevotella 9	Bacteroidetes	0.003893926	ASV428
818434292143daca42a3168f3728da82	Guild44	Dialister	Firmicutes	0.001739946	ASV429
5edd946e0a26ae1c85ed8c498c328387	Guild44	Faecalibacterium	Firmicutes	0.000639773	ASV430
54a2bfb0a96adc52c4ccca239ad01466	Guild44	Ruminiclostridium 6	Firmicutes	0.000626513	ASV431
c0044f57ec04cd70905d690c5b7bd147	Guild44	Coprococcus 2	Firmicutes	0.000596263	ASV432
2d0447008c3204f3ac97998db1edbbf2	Guild44	Lachnospiraceae UCG-001	Firmicutes	0.000535885	ASV433
923f521b9cf313f1f95c9367e09bbc1c	Guild44	Veillonella	Firmicutes	0.000513987	ASV434
1f3d20f8f9bfb29a4ae7eaf2962cd4b4	Guild44	Ruminococcaceae UCG-014	Firmicutes	0.000496076	ASV435
bd6cf3f5a66cefd7b96fb6cf75d23cd2	Guild44	gut metagenome	Tenericutes	0.000347374	ASV436
fa2bd2f1500f2aadfa41c605e68f78c0	Guild44	Roseburia	Firmicutes	0.000283695	ASV437
b3d2cc3b9f0184830bec2ef73bcc6166	Guild44	Lactococcus	Firmicutes	0.00026563	ASV438
9908fffab7ed4f3bec44cda2f5084d49	Guild44	Enterococcus	Firmicutes	0.000145952	ASV439
088ffa18748594532a4b153f8dc5092b	Guild44	Butyrivibrio	Firmicutes	0.000134877	ASV440
41a4c7e6cc5a80ba83637525e13a12e6	Guild44	Ruminococcus 1	Firmicutes	0.000133648	ASV441
6acfe69f470ceebec0cce442b1190fa9	Guild44	CAG-352	Firmicutes	0.000114512	ASV442
5c4563e3dd7e12dcdf279783af929eaa	Guild45	unclassified	Firmicutes	0.00446162	ASV443
f50508546ae13143015f8c4cb976d0e4	Guild45	[Eubacterium] ruminantium group	Firmicutes	0.003798046	ASV444
31654b0fabcee256ee93ddfc89c8b048	Guild45	Ruminococcaceae NK4A214 group	Firmicutes	0.001510234	ASV445
973ca62579b1246f54829278d51475ea	Guild45	[Eubacterium] xylanophilum group	Firmicutes	0.001320604	ASV446
9e9c4270966cf507485654eb33a69880	Guild45	Weissella	Firmicutes	0.000211393	ASV447
3265d4af36c9111eeab8366f034a548b	Guild46	Fusobacterium	Fusobacteria	0.002674828	ASV448

5d08d1eb4b8a0672652dd0fc0af2dcf0	Guild46	Ruminococcaceae UCG-003	Firmicutes	0.001501635	ASV449
68d79c3035521e82b504b081a197d2ed	Guild46	Ruminococcaceae UCG-002	Firmicutes	0.000949632	ASV450
497aa38580aba714ec227939456c1c8c	Guild46	Barnesiella	Bacteroidetes	0.000576277	ASV451
e0cdaf99e96ca363991154bd1da46a59	Guild46	unclassified	Firmicutes	0.000514331	ASV452
7ee3e4343242684ba3ce459672348ff7	Guild46	Bacteroides	Bacteroidetes	0.000457425	ASV453
8d5153b41e59c5ffb5a1fc080eb784cf	Guild46	Ruminococcaceae UCG-004	Firmicutes	0.000307637	ASV454
517b36b986012394a442af4336b89612	Guild46	Faecalitalea	Firmicutes	0.000202397	ASV455
b1956a37445160b890fb5a06a818c385	Guild46	Parabacteroides	Bacteroidetes	0.000185351	ASV456
27a7197b6f932b622896bc3dc90f3200	Guild46	[Ruminococcus] gauvreauii group	Firmicutes	0.000114837	ASV457
a62fa6e25e8805dafe5b628529c90023	Guild46	uncultured	Firmicutes	9.38E-05	ASV458
7b25fe2a8e4d57a05ef403d1d0e831e2	Guild46	Intestinimonas	Firmicutes	6.07E-05	ASV459
0df6c802966e8670279671824da4f10a	Guild46	Lactobacillus	Firmicutes	5.85E-05	ASV460
2a8dc8ea97ba5174f1da58a6fea547e9	Guild46	Peptococcus	Firmicutes	5.43E-05	ASV461
997720e304cb420e94f46a9957346b14	Guild46	Ruminococcaceae UCG-005	Firmicutes	3.71E-05	ASV462

Table S6. Relative abundance of guilds based on 16S rRNA data							
guild_name	W1	W10	W11	W12	W13	W14	W15
Guild8	0.000654004	0.000214758	0.007599425	0.00160888	0.001495821	0.00517578	0.004418601
Guild9	0.007792389	0.009835925	0.043431511	0.008797178	0.011555947	0.072958843	0.030184741
Guild11	0.037152995	0.01145377	0.07612502	0.033565071	0.008784279	0.028211277	0.109584028
Guild15	0.12220135	0.007101337	0.026939452	0.01455372	0.015295498	0.029272639	0.008796541
Guild20	0.022277882	0.005426223	0.01099955	0.019837931	0.012831794	0.048154409	0.012862739
Guild21	0.002908231	0.003622255	0.025035963	0.006258395	0.042440241	0.006473001	0.002873446
Guild22	0	0.000157489	0.001031662	0	0.005924622	0.168062162	0.030415159
Guild23	0.001071453	0	0	2.95E-05	0.001510485	0.001926176	0.016278345
Guild24	0.003256105	0.002161899	0.004402726	0.010952191	0.004634111	0.032037423	0.004649019
Guild25	0.000765324	0.00087335	0.035614129	0.010686505	0.003739551	0.004848199	0.003266512
Guild26	0.045028874	0.093920912	0.012859447	0.034244048	0.200117319	0.016365947	0.014204584
Guild27	0.002198567	0.001932824	0.003167638	0.003955778	0.000454612	0.000759988	0.000243972
Guild28	0.057204481	0.002591415	0.003690734	0.06380906	0.005176712	0	0
Guild29	0.003757044	0.029980242	0.006175441	0.014066628	0.000879894	0.003590288	0.001206305
Guild30	0.029485842	0.013801793	0.034902137	0.078156135	0.075626925	0.056645308	0.034372925
Guild34	0.036011967	0.003708158	0.012191046	0.008265805	0.003416923	0.011163961	0.001653587
Guild37	0.00335351	0.000658592	0.002441115	0.002774949	0.004267488	0.006237142	0.001721357
Guild41	0.015181243	0.012728002	0.040510891	0.055941785	0.024915677	0.04460343	0.018352106
Guild42	0.007569749	0.003393179	0.00389416	0.017431992	0.007405778	0.005595084	0.007929086
Guild43	0.002435121	0.003378862	0.002731724	0.02059071	0.001774454	0.007730912	0.001640033
Guild44	0	0.000143172	0.001409454	0.000649456	0.000337293	0.012303943	0.000189756
Guild46	0.003645725	0.002849125	0.003908691	0.001254631	0.005308696	0.007744015	0.002629475
guild_name	W16	W18	W19	W2	W20	W21	W22
Guild8	0.001977201	0.004157715	0	0	0	0.00439912	0.005523
Guild9	0.014399476	0.061670669	0.303185856	0.04851067	0.020977624	0.072042734	0.01265838
Guild11	0.016199411	0.038379881	0.02323669	0.012669582	0.014704315	0.010969235	0.00350151
Guild15	0.037634995	0.035005687	0.007836932	0.035679137	0.035282365	0.010040849	0.006112602
Guild20	0.027271736	0.02145836	0.014714494	0.059286666	0.030127864	0.003713543	0.01162357
Guild21	0.003627141	0.003323645	0.013468559	0.002628596	0.007858284	0.00619876	0.001720673
Guild22	0.007772445	0.118501201	0.007263802	0.015410298	0.007645178	0.006155912	0.004139242
Guild23	0.002059016	0.001996714	0.001420366	0	0.001691529	0.06147342	0.001082941
Guild24	0.039557652	0.035410085	0.005407358	0.007449764	0.005234417	0.005213243	0.004199406
Guild25	0.005127086	0.012094022	0.004161423	0.000635348	0.003542888	0.003927786	0.003176628
Guild26	0.18456147	0.020611652	0.025703642	0.03413437	0.030327651	0.015125546	0.007953602
Guild27	0.001949929	0.008163781	0.003563374	0.00362522	0.000399574	0.00019996	0.000433177
Guild28	0.000381804	0	0	0.002790547	0.000199787	0.000257091	0
Guild29	0.016949384	0.000821433	0.000261646	0.018088724	0.000253063	0.000442769	0
Guild30	0.008972401	0.028156199	0.019835287	0.155585454	0.023894513	0.04293427	0.004861203
Guild34	0.010158722	0.004334639	0.002965326	0.006017117	0.001851359	0.004999	0.001504085
Guild37	0.006027054	0.011032478	0.003501078	0.003475726	0.008457645	0.002013883	0.000818222
Guild41	0.036380495	0.031227095	0.02594037	0.046392844	0.080713905	0.02363813	0.206528933
Guild42	0.002263554	0.004549476	0.002442033	0.005294565	0.005594033	0.004927586	0.010119485
Guild43	0.001090869	0.003475294	0.003114838	0.00424811	0.007072456	0.001885337	0.007568556
Guild44	0.000422712	0.001781878	0.000921992	0.003201654	0.002677144	0.000657011	0.001492052
Guild46	0.005372532	0.007911032	0.004298476	0.003214112	0.014984017	0.00669866	0.00080619
guild_name	W24	W25	W26	W27	W28	W29	W3
Guild8	0.000596742	0.000236522	0.000194544	0.001158961	0.00137592	0.001300715	0.001995057
Guild9	0.024077826	0.021668003	0.02618012	0.028663096	0.038455206	0.066454698	0.013250752
Guild11	0.00581476	0.091533842	0.054402957	0.01003491	0.014876402	0.005045197	0.009915731
Guild15	0.006938855	0.012916705	0.028778678	0.019871949	0.079485853	0.007226193	0.046868952
Guild20	0.002567376	0.017463175	0.017217181	0.212895566	0.082731613	0.168751314	0.016689992
Guild21	0.006467013	0.142556798	0.030237761	0.005964411	0.009266881	0.005413075	0.001503737
Guild22	0.004038414	0.01668791	0.007851257	0.000141337	0.013500482	0.000959113	0.001310187
Guild23	0.001845735	0.001931593	0.001695316	0	0	0.000867143	0.001488849
Guild24	0.003871881	0.008803858	0.080096716	0	0.00250488	0	0.002769258
Guild25	0.003358406	0.179914064	0.05120687	5.65E-05	0	0	0.000923086
Guild26	0.109758805	0.01516366	0.021497158	0.011730951	0.009643202	0.013874291	0.011985231
Guild27	0	0.001629371	0.00721204	8.48E-05	0.00039984	0.000170801	0.003409463
Guild28	0	0	0.002820894	0.004084632	0.00028224	0	0.002054611
Guild29	0.004773932	0.007358448	0.013312397	0.00111656	0.00049392	0.000972251	0.01749397
Guild30	0.050126287	0.00913236	0.163375624	0.037581445	0.027988804	0.012731238	0.21417086
Guild34	0.007327431	0.035675335	0.012381363	0.007589784	0.005150881	0.001326992	0.003290355
Guild37	0.001373893	0.00310106	0.004168809	0.00480545	0.004892161	0.000499264	0.001533514
Guild41	0.022426379	0.022311867	0.03717188	0.036196345	0.045017287	0.01370349	0.023121818

Guild42	0.007077632	0.003403282	0.006809055	0.014077142	0.008102641	0.00438827	0.004168776
Guild43	0.003427794	0.00147169	0.004168809	0.02814015	0.004021921	0.001129914	0.001235744
Guild44	0.000277554	0.001064347	0.003988161	0.00080562	0.00226968	0.001274438	0.000401989
Guild46	0.027935829	0.003285022	0.004780234	0.005512134	0.011148482	0.002562014	0.002292827
guild_name	W30	W31	W32	W33	W34	W35	W36
Guild8	0.002378948	0.006777138	0	0	0.005480576	0.007144899	0.001958189
Guild9	0.011809343	0.055572535	0.012397364	0.038434479	0.019958676	0.005584929	0.00699156
Guild11	0.027217607	0.008783703	0.079234046	0.018005576	0.007151126	0.003274745	0.00467483
Guild15	0.018677793	0.025367759	0.015478279	0.014214243	0.030055245	0.004239306	0.003488885
Guild20	0.074808769	0.130891792	0.020740894	0.109935639	0.01433156	0.132859388	0.014838105
Guild21	0.010211177	0.008610952	0.052758819	0.080907835	0.042818833	0.004334572	0.29805836
Guild22	0.000524589	0.002657701	0.000147412	0.00019543	0.000337041	8.34E-05	0.000220641
Guild23	0	0	0	0.000208458	0.003370408	0	0
Guild24	0.009015603	0.00240522	5.90E-05	0	0	0	0
Guild25	0.00093938	0.000544829	0.000132671	3.91E-05	0	0	0.001903028
Guild26	0.01651844	0.044436767	0.031605171	0.024819553	0.020295717	0.00389397	0.009046279
Guild27	0.000890581	0.006391772	0.004672966	3.91E-05	0.000205155	0.000119082	4.14E-05
Guild28	0.00046359	0.000199328	0.000206377	0	8.79E-05	0	0.000496442
Guild29	0.008893606	0.000159462	0.001238262	9.12E-05	0.000102578	0.001333714	0.001379006
Guild30	0.015322866	0.009953092	0.043412887	0.003296245	0.024076435	0.000226255	0.006260687
Guild34	0.012736522	0.002059719	0.009950322	0.000912005	0.003604871	0.011205583	0.001847868
Guild37	0.001341971	0.003401858	0.003478927	0.002814186	0.001538664	0.000702582	0.000620553
Guild41	0.03254889	0.041353833	0.046523284	0.066250619	0.076112601	0.043548157	0.010728667
Guild42	0.006246264	0.030576854	0.005056238	0.011712745	0.015430606	0.02361389	0.005860776
Guild43	0.003757518	0.013036025	0.008815248	0.011478229	0.005099574	0.005954082	0.001406586
Guild44	0.002269151	0.003043068	0.008196117	0.000925033	0.001509357	0.012051062	0.000441282
Guild46	0.006343862	0.003866955	0.010923242	0.010813769	0.001611934	0.000964561	0.003599206
guild_name	W37	W38	W39	W4	W40	W41	W45
Guild8	0.000496553	0.000665648	0.006039841	0.001596259	0.006367277	0.023405054	0.035791146
Guild9	0.020770082	0.039778611	0.006608616	0.031183052	0.008347882	0.009160187	0.018025565
Guild11	0.155747241	0.033763128	0.001557358	0.066412759	0.003726471	0.001319688	0.006817361
Guild15	0.023706835	0.007580987	0.024348957	0.062310094	0.008817359	0.004062569	0.015743482
Guild20	0.065814488	0.299640057	0.011808838	0.01845499	0.043925412	0.015810379	0.084393731
Guild21	0.108901058	0.015852276	0.002031337	0.003192517	0.003506404	0.0002717	0.000519968
Guild22	0.000893795	6.16E-05	0.000108338	0.000266043	0.014539106	0.001035049	0.000346645
Guild23	0	0	0	0	0	0.001112678	0
Guild24	0.000127685	0.00467186	0	0.097413781	0.001995276	0	0.008680581
Guild25	0.008654201	0.003352892	0	0.006441043	0	0	0.00040442
Guild26	0.019776977	0.010514768	0.045352979	0.033535433	0.069350508	0.067304085	0.034231241
Guild27	7.09E-05	0.000739609	0	0.011719899	7.34E-05	0	0.000534412
Guild28	0	0.00231744	0.005714827	0.003374547	0.000454806	0.000401082	0.000245541
Guild29	0.003319808	0.007741236	0	0.092849042	0.000806913	0.001022111	0.008276161
Guild30	0.011080215	0.02375376	0.0606828	0.022515648	0.135664089	0.038115693	0.01642233
Guild34	0.032701529	0.008172674	0.002369893	0.057143257	0.001863236	0.001229121	0.011569293
Guild37	0.001872712	0.001380603	0.019866473	0.002856463	0.004063908	0.001267935	0.003668665
Guild41	0.032091479	0.01915586	0.04632802	0.024153913	0.050263347	0.020157586	0.026720589
Guild42	0.00422779	0.045116119	0.015641293	0.003570578	0.015932865	0.016910119	0.004881924
Guild43	0.002653009	0.003266604	0.003615779	0.001960318	0.00908144	0.00020701	0.001747671
Guild44	0.001021479	0.001627139	0	0.006090987	0.000880269	0	0.005661876
Guild46	0.0043271	0.003032395	0.002275097	0.005586905	0.004870821	0.000672782	0.003119809
guild_name	W46	W47	W48	W49	W5	W50	W51
Guild8	0.019871582	0.005796583	0.00538052	0.135936216	0.002152124	0.269595154	0.004292166
Guild9	0.010060334	0.005617591	0.003144952	0.01748273	0.054811535	0.024192042	0.021999222
Guild11	0.006808369	0.006374864	0.002216623	0.025927391	0.019615077	0.000440341	0.001779679
Guild15	0.014267132	0.005741508	0.022090446	0.017634886	0.083158089	0.002762136	0.003499536
Guild20	0.013699768	0.004309573	0.013546028	0.00401692	0.071438234	0.008606656	0.002572309
Guild21	0.001065537	0.003497226	0.000189455	0.005873224	0.006751522	0.00040031	0.006894386
Guild22	0.003653271	0.000289141	0.001401967	0.000289096	0.005497141	0.000226842	0
Guild23	0.000138381	0	0	0.00502115	0	0.000106749	0
Guild24	0.002421676	0	0	0	0.015667466	0	0
Guild25	0.000871803	0	0	0	0.0057308	0	0
Guild26	0.144650172	0.377246004	0.000625201	0.024466693	0.098444321	0.010341331	0.096850417

Guild27	0.003874682	0.001996448	9.47E-05	0.000304312	0.085408596	0	0
Guild28	0.000622717	0	0	6.09E-05	0.004402632	0.00040031	8.97E-05
Guild29	0.000636555	0.000137686	0	0	0.005447949	0	0.001031915
Guild30	0.313987601	0.177904143	0.00231135	0.015078665	0.049486565	0.009193777	0.050264709
Guild34	0.00164674	0.001900067	0.001458803	0.04248197	0.004968333	0.001414427	0.006266264
Guild37	0.003155098	0.002519655	0.068923706	0.001780226	0.004882248	0.001174241	0.000837496
Guild41	0.045264585	0.099836154	0.18780668	0.021423572	0.041751214	0.04084492	0.022941405
Guild42	0.014668438	0.014553415	0.088986984	0.006238398	0.006800713	0.058845507	0.003469626
Guild43	0.003016716	0.001803687	0.008563363	0.005766714	0.004058292	0.099757146	0.011291239
Guild44	0.004691133	0.000261603	0	0.005127659	0.000885445	0.002321796	0
Guild46	0.004137607	0.001390629	0.00075782	0.004853778	0.00486995	0.000467028	0.009317142
guild_name	W52	W53	W54	W55	W57	W59	W6
Guild8	0.005718861	0.004983962	0.002878008	0.000532148	0.001598611	0.000897525	0
Guild9	0.043755639	0.004576857	0.015328245	0.056908533	0.131040468	0.03246835	0.035929403
Guild11	0.011120007	0.006822107	0.014478789	0.009625618	0.137648062	0.061535555	0.013263237
Guild15	0.052524559	0.006661732	0.017813221	0.015056658	0.014692001	0.031602318	0.039594442
Guild20	0.039409305	0.016111522	0.010535791	0.022084142	0.002649128	0.010360899	0.017213669
Guild21	0.008374954	0.003047126	0.001863732	0.000829525	0.002847051	0.01067582	0.005812993
Guild22	0.000940435	0.000209721	0.000595887	0	0.000304497	0.000425143	0.000540744
Guild23	0.000114377	0.000172712	0	0.000156514	0	0	0
Guild24	0	0.004046385	0.000126784	0.000516497	0.002085807	0.001401398	0.00322944
Guild25	0.008489331	0.003466568	0.000988919	0.000673011	0.00121799	0.000582604	0.000165227
Guild26	0.048686568	0.022452504	0.004272637	0.017169599	0.00943942	0.016706557	0.11792715
Guild27	0.000152503	0.000542808	5.07E-05	0.000375634	0.000137024	0.001165208	0.02275629
Guild28	0	0.002257587	0	0.001878169	0.000228373	0.00015746	0.002898986
Guild29	7.63E-05	0.001110289	0.002015873	0.000923433	0.007856034	0.000771556	0.004551258
Guild30	0.012721288	0.103552924	0.08592185	0.06762975	0.029612375	0.033633558	0.174855426
Guild34	0.019113704	0.002331606	0.027372772	0.013788894	0.018406869	0.00848712	0.005843034
Guild37	0.002401922	0.002356279	0.005832087	0.006996181	0.004658811	0.014297411	0.02069846
Guild41	0.055371281	0.033691093	0.05910693	0.056470294	0.036448342	0.106884172	0.043169358
Guild42	0.005566358	0.01597582	0.011537389	0.012568084	0.011296855	0.024059961	0.003860308
Guild43	0.003355065	0.004453491	0.109947511	0.040286734	0.025334186	0.0342634	0.004581299
Guild44	0.000101669	9.87E-05	0.011169714	0.017952169	0.022837307	0.016549096	0.009553136
Guild46	0.001664824	0.002072539	0.007733854	0.006166656	0.006775068	0.035523084	0.005242208
guild_name	W60	W61	W62	W63	W64	W65	W66
Guild8	0.003053619	0.00194045	0.000572337	0.000441191	0.00221009	0.000508047	0.006033332
Guild9	0.33509257	0.013634553	0.057806041	0.06815017	0.031599027	0.127077256	0.121940062
Guild11	0.007658091	0.046249534	0.010709062	0.032399939	0.019075183	0.070602117	0.014180802
Guild15	0.003125751	0.003225516	0.014702703	0.007693262	0.029928304	0.005736012	0.022896988
Guild20	0.001514787	0.0009638	0.002162162	0.002178379	0.008682497	0.014848078	0.006379506
Guild21	0.001094013	0.00116941	0.006041335	0.000675573	0.002052227	0.005113245	0.001223975
Guild22	0	0.00019276	0.000419714	0.000482552	5.26E-05	0.000426104	0
Guild23	0	0.000873845	0.00017806	0	0.000118398	0.00399882	0
Guild24	0.00022842	0.000154208	0.000763116	0.000193021	0.000381504	0.004769085	0.000234904
Guild25	0.000336619	0.001760541	0.000419714	0.005197778	0.006196145	0	0.000642896
Guild26	0.00419572	0.008841256	0.01091256	0.00552867	0.011629284	0.008751516	0.004673359
Guild27	2.40E-05	0	8.90E-05	0	0.000197329	0.003687436	4.95E-05
Guild28	0.001695119	0.000308416	0.000165342	0.000606637	0.00089456	0.00047527	6.18E-05
Guild29	0.000144265	6.43E-05	0.001348172	0.000454978	0.000828784	0.000671933	9.89E-05
Guild30	0.015292138	0.064548877	0.046499205	0.013607975	0.030914951	0.049575535	0.064771772
Guild34	0.002596778	0.005114564	0.002976153	0.067309151	0.002775768	0.003277721	0.020139954
Guild37	0.002163982	0.002390223	0.008165342	0.003667397	0.022061435	0.011619522	0.00551407
Guild41	0.040754989	0.030661681	0.04136089	0.031669217	0.071130698	0.021518241	0.161428713
Guild42	0.206419812	0.025739877	0.008127186	0.029559774	0.021429981	0.033154151	0.025901291
Guild43	0.110110604	0.027616074	0.016267091	0.026554163	0.033993291	0.016552493	0.015058602
Guild44	0.008619861	0.014341339	0.045532591	0.044463747	0.024442544	0.026369268	0.137357203
Guild46	0.004243809	0.167456982	0.008	0.007665688	0.007195948	0.012652004	0.006923495
guild_name	W67	W68	W69	W7	W70	W71	W72
Guild8	0.032067094	0.009147578	0.000527804	9.79E-05	0.002549342	0.000742179	0.000896788
Guild9	0.015329635	0.00183252	0.014155987	0.11703091	0.009073465	0.002369262	0.018139573
Guild11	0.005137955	0.021464514	0.004060034	0.00483346	0.017584978	0.00476707	0.005570955
Guild15	0.023102747	0.017453999	0.019420498	0.014341303	0.012445175	0.039934917	0.004076308

Guild20	0.002250111	0.00253849	0.004587839	0.055150388	0.000246711	0.005709066	0
Guild21	0.00018049	0.006413819	0.000663139	0.005482	0.001288377	0.002098082	0.003342573
Guild22	0	0.000841157	0	0.000269205	0	0	5.44E-05
Guild23	0	0	0.000108268	0	0	0	0
Guild24	0.000144392	0	0	0.003438487	0.000561952	0.000313999	0.000163052
Guild25	0.000216588	0.001517086	0	0.000770906	0.000328947	0.000171272	8.15E-05
Guild26	0.093530028	0.000495682	0.003234494	0.049117741	0.007963268	0.002825988	0.001970216
Guild27	3.61E-05	0.000570785	0.000175935	0.001713125	0	0.000171272	0
Guild28	4.81E-05	0.000180248	0.001935283	0.003438487	8.22E-05	8.56E-05	0.001127779
Guild29	0.000649765	0.000781074	0.001042075	0.00414821	0.002535636	0	0
Guild30	0.261397957	0.039279009	0.035498234	0.006265143	0.10276864	0.01601393	0.057761291
Guild34	0.002105719	0.124100638	0.059547171	0.002471795	0.035594846	0.025448162	0.009158106
Guild37	0.007267739	0.010259106	0.011679366	0.003157045	0.012815241	0.046000799	0.007160715
Guild41	0.080065458	0.047254976	0.074988835	0.026480018	0.066680373	0.278017241	0.084977444
Guild42	0.013031393	0.011746151	0.007944134	0.059775825	0.009279057	0.033840489	0.051266373
Guild43	0.031706114	0.028088622	0.019339297	0.004968062	0.014857456	0.012174583	0.01550356
Guild44	0.057071005	0.004040556	0.006347187	0.000354862	0.007168311	0.003739438	0.002540899
Guild46	0.002996138	0.007795719	0.003031492	0.009789286	0.001589912	0.006351336	0.003165933
guild_name	W73	W74	W75	W76	W77	W78	W79
Guild8	0.000591928	0.000689089	0	0.00407735	0.001895297	0.001773574	0.006801611
Guild9	0.001679423	0.009819512	0.010947483	0.005458602	0.010145412	0.013678016	0.001577974
Guild11	0.003207422	0.006665607	0.008661888	0.003094536	0.007931578	0.003479967	0.000734574
Guild15	0.003111062	0.013039676	0.012505844	0.019257843	0.012964467	0.019845215	0.015017956
Guild20	0	0.001417932	0.000285699	0.010691423	0.008950897	0.002109478	0.000163239
Guild21	0.000743351	0.003949007	0.002064828	0.00083672	0.0021342	0.000550883	0.000435303
Guild22	9.64E-05	0.000198776	0.000155836	0.001115627	0.000127415	0	0
Guild23	0.000261549	0	0	0	0.0010671	0	0
Guild24	0	0.001563701	0	0	0.001274149	0	0.000122429
Guild25	0.000578162	0	0.001610306	0.001288283	0.000127415	8.06E-05	0
Guild26	0.002574197	0.029776576	0.00092203	0.002576567	0.001736028	0.000927095	0.016813581
Guild27	0	0	0	0	0.000637075	0	0
Guild28	0.000523099	0	0.00053244	0.000491407	0.000907831	0.000309032	0.000136032
Guild29	0	0.000596327	6.49E-05	0.000292188	0.000318537	0	0.001401132
Guild30	0.292412312	0.171105987	0.014233027	0.018846123	0.365234842	0.056525945	0.055746001
Guild34	0.038186223	0.015199703	0.048503974	0.012245332	0.013139663	0.160145648	0.01051529
Guild37	0.008782556	0.018247595	0.010012467	0.084256382	0.038112985	0.011434176	0.016283056
Guild41	0.076413744	0.058042458	0.068593839	0.205700321	0.042110628	0.122040685	0.122714659
Guild42	0.026884533	0.007169171	0.020492442	0.060217282	0.022807269	0.017090802	0.011141038
Guild43	0.019478553	0.016180329	0.008557997	0.01672112	0.008425311	0.009029103	0.050005441
Guild44	0.069888773	0.046261695	0.012077295	0.006135947	0.007278577	0.05444334	0.011236261
Guild46	0.020841363	0.002239538	0.00315568	0.001700003	0.002341249	0.004501115	0.000176842
guild_name	W8	W80	W9				
Guild8	0.009400672	0.000791538	0.006627192				
Guild9	0.034338954	0.001971649	0.005244126				
Guild11	0.005844038	0.001208894	0.002405959				
Guild15	0.016583679	0.005900554	0.019867168				
Guild20	0.004742179	0.002173131	0.071732146				
Guild21	0.003849533	0.000474923	0.000561871				
Guild22	0	0.000115133	0				
Guild23	0.005104816	0	0				
Guild24	0.003542687	0.000172699	0.00230511				
Guild25	0.000599746	0.000575664	0.00070594				
Guild26	0.137118708	0	0.071098241				
Guild27	0.006457732	0	0.001512729				
Guild28	0.002398985	0.000489314	0.002290703				
Guild29	0.002761622	0.003324459	0.003544107				
Guild30	0.018173703	0.006519393	0.004970394				
Guild34	0.007113268	0.007800245	0.00230511				
Guild37	0.003500844	0.001798949	0.000561871				
Guild41	0.048188906	0.04176441	0.069888058				
Guild42	0.006625103	0.009066705	0.019895982				
Guild43	0.015523662	0.019946751	0.006483122				
Guild44	0.001380811	0.000474923	0.001066113				

Guild46	0.001283178	0.00225948	0.000100849				

Table S7. Relative abundance at species level based on the metagenomic sequencing data

species	W9	W71	W70	W67	W66	W64	W63	W6	W62	W61
Absiella_do	0	0	0	0	0	0	0	0	0	0
Acidaminoc	0	0	0	0	0	0	0	0	0	0
Acidaminoc	0	0	0	0	0	0	0	0	0	0
Acidaminoc	0	0	0	0	0	0	0	0	0	0
Actinobacul	0	0	1.73E-05	0	0	0	0	0	0	0
Actinomyce	0	0	5.18E-05	0	0	0	0	0	0	0
Actinomyce	0	0	3.16E-05	0	0	0	0	0	0	0
Actinomyce	4.98E-05	0	0.000112346	0.001120943	3.20E-06	6.94E-05	0.000124232	5.28E-05	0.000502109	0.000631322
Actinomyce	0	0	5.31E-05	0	0	0	0	0	0	0
Actinomyce	0	0	0	0.000266334	0	0	0	0	5.60E-05	0.000176134
Actinomyce	0	0	0	0	0.000468618	0	0	0	0	0
Actinomyce	3.71E-05	0.00021302	0.000383058	0.000338743	0	0.000209502	0.000121331	0.00017	0.000220748	4.19E-05
Actinomyce	0	0	8.63E-05	0	0	0	0	0	0	0
Actinomyce	0	3.69E-05	0	0.000118215	0.000130833	0	4.48E-05	0	0	6.49E-05
Actinomyce	0	0	6.90E-05	0	0	0	0	0	0	0
Actinomyce	0	0	0	0	0	0.000107501	0	4.27E-05	0	0
Actinomyce	0	0	0	0	0	0	0	0	0	0
Actinomyce	0	0	0	0	0	0	0	0	0	0
Adlercreutz	0	0	0	0	6.48E-05	0.001531817	0.004777216	8.82E-05	0.002336605	0
Aeromonas	0	0	0	0.067022579	4.94E-05	0	0.001463373	0	0	0
Aeromonas	0	0	0	0.004187336	0	0	8.10E-05	0	0	0
Aeromonas	0	0	0	0	0	0	0	0	0	0
Agathobacu	0.003784599	0	0.013367709	0.01273173	0.000605853	0.000477505	0.000172144	0.002611398	0.003027455	0.000267852
Aggregatiba	0	0	0	0.000222328	0	0	2.11E-05	0	0	0
Akkermansi	0	0	0.000520114	2.05E-05	0	0	0.00058825	0.017936189	0	0.106234967
Alistipes_fir	0	0	0	0	0	0.00823159	0.009864011	0.014684991	0.012828974	0.001683426
Alistipes_in	0	0	0.007178458	0	0.002524936	0.000368404	0.000309879	0	0	0.001918071
Alistipes_in	0	0	0	0	0	0	0.004536255	0	0	0
Alistipes_or	0	0	0	0	0	0	0	0	0	0.000504698
Alistipes_pu	0	0	0	0.004304451	0.123121102	0.147819811	0.213257395	0.138479017	0.074412088	0.267368663
Alistipes_sh	0	0	0	0.000680187	0.018685605	0.014475458	0.009834704	0.002801398	0.031362881	0.004438859
Allisonella_	0	0	2.99E-05	0	0	0	0	0	0	0
Anaeroglob	0	0	0.000625858	0	0	0	0	0	0	0
Anaeromas	0	0	0	0	0	0	0	0	0	0
Anaerostipe	0	0	0.000920779	5.57E-05	0	0.00092261	2.77E-05	0.0001074	0	0
Anaerotrun	0	0	0.000227594	0	0	0	9.81E-05	6.42E-05	0	0.002784739
Asaccharob	0	0	0	0	1.02E-05	0.000492705	0.00145487	2.09E-05	0.000702052	0
Atlantibacte	0	0	0	0.000649783	0	0	0	0	0	0
Atopobium	0	0	0.000258707	0	0	0	0	0	0	0
Atopobium	0	0	0	0	0	0	0	0	0	0
Bacteroides	0	0.027774039	0.047066896	0.000201826	0.009856882	0.0091618	0.002271078	0.006021096	0.000391485	0.060499013
Bacteroides	0.005152899	0	0	0	0	0.001245014	0	0	0	0
Bacteroides	0	0	0.002076756	0	0.000275769	0	0.001978704	0	0.000706153	0
Bacteroides	0	0	0	0.004211339	0	0.160787953	0	0	0.044279073	0
Bacteroides	0	0	0	0	0.009232225	0	0	0	0	0
Bacteroides	0	0	0	0	0.001483774	0.020558924	0	0.005541397	0	0.004454362
Bacteroides	0	0	0	0	0	0.021170731	0	0	0	0
Bacteroides	0	0	0	0	0	0	0	0	0.000253555	0
Bacteroides	0	0	0	0	0	0	0	0	0.004399151	0.000253649
Bacteroides	0.026323995	0.005554928	0	0	0.000169943	0	0.005350662	0	0.003460048	0.003998674
Bacteroides	0	0.000940289	0.013168027	4.11E-05	0.000185347	0	0.115336265	0	0	0
Bacteroides	0	0	0	0	0	0	0	0	0	0
Bacteroides	0	0	0	0	2.00E-06	3.08E-05	0.002392209	0	1.78E-05	0.027263078
Bacteroides	0.019408496	0	0	0	0	0.004178446	0.000280271	0.009595094	0.012068009	0.003747325
Bacteroides	0	0	0.001955206	0	0	0	0	0	0	0
Bacteroides	0	9.52E-05	0.023623735	9.07E-05	0.000602352	0.009264001	0.00094134	0.004402897	0.001798189	0.022928539
Bacteroides	0	0	0	0.076024431	0.016120759	0.315969144	0	0	0.311030845	0.013692451
Bacteroides	0	0.000266225	0	0	0	0.000463105	0.001105882	0	0	0
Bacteroides	0	0	0	0	0	0	0	0	0	0
Bacteroides	0	0	0	0	0	0	0	0	0	0
Bacteroides	0.544097891	0	0.168473328	0.002927675	0.00639561	0.002218324	0.116270302	0.023513486	0.023250727	0.050656807
Bacteroides	0	0.173537186	0.019907304	0.000410753	0.001264618	0.005422259	0.011934939	0.0005093	0.000827179	0.004829635
Bacteroides	0.174630065	0.009865637	0.139473277	0.001189252	0.011869289	0.042114259	0.069706847	0.013106192	0.102445249	0.133859815
Bacteroides	0.128320974	0.265561428	0.20628541	0.004858622	0.007780259	0.037030304	0.094490957	0.045858672	0.051132755	0.06740905
Bacteroides	0.034571493	0.002554543	0.015810816	0.00031134	0.000123531	0.003732841	0.006424736	0.001488299	0.000187641	0.011445916
Barnesiella	0	0	0	0	0	0.009070199	0.001041565	0	0.008528944	0.008733191
Bifidobacter	0	0.000586456	0.024646857	0	9.32E-05	9.99E-05	0	0	0.000427492	0
Bifidobacter	0	0.054811207	0	0	0.000405802	0	0	0.005662197	0.000171937	0
Bifidobacter	0.002729399	0	0	0	0	0.013665849	0	0.003843298	0	0
Bilophila_w	0.0007394	0	0.000185777	2.70E-06	0.000167142	0.001234213	0.000358291	0.000789	0.000198043	0.001314655
Blautia_han	0	0	8.39E-05	0	0	0	0	0	0	0
Blautia_obe	0	0	2.30E-06	0.000172222	1.70E-06	0.000106201	2.70E-05	7.52E-05	0.000282861	0
Blautia_sp	0.0001147	0	0	0	0	0	0	0	0	0
Blautia_wex	0.0013562	0.016615278	0.002289844	0.000339543	0	0.000233003	0	0.000209	3.69E-05	0
Butyricicocc	0	0	0	0	0	0	0	0	0	0
Butyricimon	0	0	0	0	0	0.000480005	1.80E-06	0	0.000939303	0
Butyricimon	0	0	0	0	0.000473519	0.000128201	0.000602253	0	0.000404888	0.002493183
Butyrivibrio	0	0	0	0	0	0	0	0	0.00869398	0
Candidatus	0	0	0	0	0	0	0	0	0	0
Catabacter	0	0	0	0	0	0	0	0	0	0
Christenser	0	0	0	0	0	0	0	0	0	0
Citrobacter	0	0	0	0	0.001361443	0	0	0	0	0
Citrobacter	0	0	0	0	0.000177045	0	2.70E-05	0	0	0
Citrobacter	0	0	0	0	0	0	0	0	0.000166036	0
Citrobacter	0	0	0	0	9.30E-05	0	0.000244262	0	0	0
Citrobacter	0	0	0	0	0.000104226	0	0	0	0	0
Citrobacter	0	0	0	0	0.001034861	0	0.000251564	0	0	0
Cloacibacill	0	0	0	0	0	0	0	0	0	0
Clostridium	0	0	0	0	0	0	0	0	0	7.32E-05
Clostridium	0	0	0	0	0	0	0	0	0	0
Clostridium	0	2.00E-05	0	0	4.70E-06	0	0	7.77E-05	0	0.00025685
Clostridium	0.0002955	0	0	0	0	0	0	0	0	0
Clostridium	0.0001135	0	4.69E-05	0	0	0	1.60E-05	0	0	0.000266552
Clostridium	0	0	0	0	0	0	0	0	0	9.21E-05
Clostridium	0	0	0	5.59E-05	0	0	5.50E-06	0	0.000339173	0
Clostridium	0.0001945	0.00041974	0	0	0	0	0	0	0	0
Clostridium	0	0	0	0	0	0	0	0	0	0.000342666
Clostridium	0	0	0	0	0	0	0	0	0	0

Clostridium	0	0	0	0	0	0	0.000647065	0	0	0
Clostridium	0	0	0	0	0	0	0	0	0	0
Clostridium	0	0	0	0	0	0	0	1.46E-05	0	0
Clostridium	0	0.000302629	0	5.29E-05	0	0	0	0.0001111	0	0
Clostridium	0	0	0	0	0	0	0	0	0	0
Clostridium	0	0	0	0	0	0	0	0	0	0
Clostridium	0	0	0	0.00039325	0.003443267	0	0.001340341	0	0.000160935	0.000905975
Clostridium	0	0	0	0	0	0	0	0	0	0
Clostridium	0.0013819	0	0.00029072	0	0	0	0	0	0	0.001970982
Clostridium	0	0	0	0	0	0.000177802	0	0	0	0
Collinsella_	0.0016863	0	0.006323206	0.009829558	0.002665771	0.009760006	0.000719183	0	0.001217863	0.004263125
Collinsella_	0	0	0	0	0	0	5.10E-06	0	0	0.001436778
Collinsella_	0	0	3.70E-06	5.80E-06	0	0	0	0	0	0.000134326
Comamona	0	0	0	0	0	0	0	0	0.000199543	0
Coprobacillu	0	0	7.68E-05	0	0	0	0	0	0	0
Coprobacte	0	0	0	0	0	0.002467527	0.000338486	0.003040398	0	0
Coprobacte	0	0	0	0	0	0	0	0	0	0
Coprococcu	0	0	0.000758012	0	0	0.000303803	1.70E-06	0	0	0.000189137
Coprococcu	0	0	0.00082464	0	0.000391399	0.000655207	0.000123131	0	0.000241052	0
Coprococcu	0	0	0	0	0.000688173	0.002551528	0.000632761	0	0.006734656	0
Corynebact	0	0	0	0	0	0	0.000338386	0	0	0
Corynebact	0	0	0.000118449	0	0	0	0	0	0	0
Desulfovibri	0	0	0	0	0	0	0	0	0	0
Desulfovibri	0	0	0	0	0.002538339	0	0.023907587	0	0	0.008071563
Desulfovibri	0	0	0	0	0	0.007074977	0	0	0	0
Dialister_inv	0	0	0	0	0.002803006	0	0	0	0.000863587	0
Dialister_su	0	0	0	0	0	0	0	0	0	0
Dielma_fast	0	0	0.000330436	0	0	0	0	0	0	0.000123024
Dorea_form	0.0008688	0	0.002261932	0.001454486	5.31E-05	0.000403904	0.000140836	0	0.000251754	0
Dorea_long	0.0007628	0	0.000694486	0.001443685	5.55E-05	0.000488505	2.07E-05	0.0003045	2.15E-05	0
Eggerthella	0	0	0.001187189	0	0	1.83E-05	0.000520332	0.001107899	0	0.002584
Eisenbergie	0	0	0	0	0	0	0	0	0	0
Eisenbergie	0	0	0	0	0	0	0	0	0	0.00030946
Enterobacte	0	0	0	0	0	0	0	0	0.053828638	0
Enterobacte	0	0	0	0.00226459	0.001818058	0	0.004218674	0	0.001212962	9.44E-05
Enterobacte	0	0	0	0	0	0	0.000425608	0	0.000127528	0
Enterococcu	0	0	0	0	0	0	0.00015874	0	0	0
Erysipelatoc	0.0002999	0.016723989	7.10E-05	9.00E-06	0	0	0	0	0	0.001129519
Escherichia	0.0006653	5.01E-05	0.075608358	0.002923574	0.011096994	0	0	8.80E-05	0.003669593	0.012776273
Eubacteriur	0	0	0	0	0	0	0	0	0	0
Eubacteriur	0	0	0.008326131	0.009351397	0.002814209	0.00365034	0.000172344	0.000952499	0.11142579	0
Eubacteriur	0.0010418	0	0.001113759	7.57E-05	4.59E-05	0.000603207	0	0.0004896	0.000117125	0
Eubacteriur	0	0	0.004096988	0.000653184	6.42E-05	0	0	0.0003383	0.000260156	0
Eubacteriur	0	0	0.090662462	0	0.001024258	0.00087671	0.000728285	0.002034499	0.001241668	0.000239446
Eubacteriur	0	0	0	0	3.45E-05	0.001166413	0.005441085	0	0.005430874	0
Eubacteriur	0	0	0	0	0.028700927	4.50E-05	0.023946297	0.069287158	0.000431693	0
Eubacteriur	0	0	0.004187526	0	0	0	0.000310679	0	0	0
Eubacteriur	0	0	0	0	0	8.59E-05	0	0.0007838	0.000117425	0
Eubacteriur	0	0.170141463	0	0.00023593	0.067492595	0.000252703	4.12E-05	8.01E-05	0.001918015	0
Eubacteriur	0	0	0	0	0	2.33E-05	0	0	0	0.00020894
Eubacteriur	0	0	0.001156176	0	0	0	0	0	0	0
Faecalibact	0.027726594	0.218064816	0.019979333	0.066499612	0.01493096	0.026718291	0.016579121	0.010132394	0.017652116	0.003705217
Firmicutes	0	0	0	0	0	0	0.001227813	0	0	0
Firmicutes	0	0	0	0	0	0	0	0	0	0.000219643
Firmicutes	0	0	0	0	0.000376295	0	0	0	0	0
Firmicutes	0	0	0	0	0	0	0	0	0	0
Firmicutes	0	0	0	0.000162921	0.001378047	0.00732008	0.009916225	0.001781999	0.002950738	0.027070141
Firmicutes	0	0	0	0	0.000347087	8.40E-05	0.000151739	0	0	0
Flavonifract	0.00016	0.000193818	0.002388784	8.79E-05	7.22E-05	4.70E-06	0.000231559	0.000877699	0	0.005350036
Fusicatenib	0.005266999	0	0.007469078	0.009097665	0.000305277	0.003091234	0.002972057	0.0002172	0.002044442	0
Fusobacteri	0	0	0.004272361	0	0	0	0	9.62E-05	0	0
Fusobacteri	0	0	0	0	0	0	0	0	0	0
Fusobacteri	0	1.51E-05	0	0	0	0	0	0	0	0
Fusobacteri	0	0	0	0	0	0	0	0	0	0.012264674
Gardnerella	0	0	0	0	0	0	0	0	0	0
Gemella_sa	0	0	0	1.63E-05	1.17E-05	0	0	0	0	0
Gemmiger	0	0	0	0.000375248	0.000697376	0.002865731	0.00035339	0.002767798	0.000664544	0
Gordonibac	0	0	0.000106544	0	0.000122131	0.002463327	0.000341187	0.0003927	0	0.000428183
Haemophilu	0	0	0	0.003148803	0	0.001225213	0	0	5.67E-05	0
Holdemane	0	0	0	0	0	0	0.000271469	0	0	0
Holdemania	0	0	0	0	0	0	8.70E-06	0	0	0.000203439
Hungatella	0.0002055	0	0.000333838	0	0	0	3.45E-05	0	0	0.000237046
Intestinibac	1.08E-05	0	0	0	0	4.08E-05	0.000194149	0	0.000108323	0
Intestinimor	0	0	0	0	0.000273969	6.65E-05	0.000174244	0.0002673	0	0.000767449
Klebsiella_a	0	0	0	0	0	0	0.001704534	0	5.37E-05	0
Klebsiella_r	0	0	0	0	0	0	0	0	0	0
Klebsiella_c	0	0	0	0	2.56E-05	0	0.000133134	0	0	0
Klebsiella_p	3.89E-05	0	0	0.024474733	0.089519641	0.002397226	0.118416149	0.01678309	0.016952965	0.000733342
Klebsiella_c	0	0	0	0.002295994	0.00980907	0.000767808	0.0341706	0.002451699	0.005856666	0.000127825
Klebsiella_v	0	0	0	0.008340568	0.026774042	0.000122001	0.020899421	0.013884392	0.004170002	0.000332264
Kluyvera_as	0	0	0	0	0	0	0	0	0	0
Kluyvera_cr	0	0	0	0	0	0	0	0	0	0
Kluyvera_ge	0	0	0	0	1.55E-05	0	0	0	0.000225949	0
Lachnospira	0	0	0	0.026367275	0	0	0	0	0	0
Lachnospira	0.0009441	0	0	0	0	0	0	0	0	0.001580006
Lactobacillu	0	0	0	0	0	0	0	0	0	0
Lactobacillu	0	0	6.18E-05	0	0	0	0	0	0	0
Lactobacillu	0	0	0	0.000205726	0	0	0	0	0	0
Lactococcus	0	0	0	0	0	0	0.000953043	0	0	0
Lactococcus	0	0	0	0	0	0	0.001519387	0	0	0
Lawsonibac	0	0	0.000234997	0	0	0	0	0	0	0
Leclercia_a	0	0	0	0	0.000775995	0	6.65E-05	0	0	0
Megamonas	0	0	0	0.000473761	0	0.002430926	0	0.078698053	0	0
Megamonas	0	0	0	0.000129017	0	0.000243603	0	0.007559095	0	0
Megamonas	0	0	0	0	0	0	0	0	0	0
Megasphae	0	0.007667428	0.002591468	0	0.001162893	2.59E-05	0	0.007386896	0.000469702	0
Megasphae	0	5.56E-05	0.001077444	0	0	0	0	0	0	0
Methanobre	0	0	0	0	0	0	0	0	0	0
Mitsuokella	0	0	0	0	0	0	0	0	0.001470618	0
Mitsuokella	0	0	0	0	0	0	0	0	9.21E-05	0

Mixta_calida	0	0	0	0.000436256	0	0	0	0	0	0
Monoglobus	0	0	0	0	0	0	0	0	0	9.77E-05
Morganella	0	0	0	0	0.000101025	0	0	0	0	0
Odoribacter	0	0	0.000144259	0	0.006165953	0.009867608	0.002322991	0	0.004630101	0.009287498
Oscillibacte	0	0	0	0.000180223	0.004729591	0.013102843	0.02211493	0	0.001728074	0.027186763
Oscillibacte	0	0	0	0	0	0	0.001381452	0	0	0
Oxalobacter	0	0	0	0	0.000670769	0	0	0	0	0
Paeniclostri	0	0	0	0	0	0	0	0	0	0
Parabacter	0	0	0	0	0	0	0	0	0	0
Parabacter	0.006631699	0.00252174	0.01339592	0.001034932	0.004194556	0.003824542	0.005560016	0.005495997	0.003485554	0.022755405
Parabacter	0	0	0	0	5.50E-06	0	0	0	0	0
Parabacter	0	0	0	0	0	0	0	0	0	0.000194238
Parabacter	0	0	0	0	0	0	0	0	0	0
Parabacter	0	0	0	0.00250232	0.00707028	0.009822607	0.00400522	0.005628597	0.008821707	0.014507409
Parabacter	0	0	0	0	0	0	0	0	0	0
Paraprevote	0	0	0	9.53E-05	9.90E-05	0.00090961	0.000474521	0	0	0
Paraprevote	0	0	0.022738671	0.000557471	0.000140935	0.002144123	0.001073373	0	0.005395166	0
Parasuttere	0	0.007712233	0.009420682	3.81E-05	2.30E-06	0	0.000163242	0.000840299	7.68E-05	0
Pediococcu	0	0	0	0	0	0	0.000249163	0	0	0
Phascolarct	0.0006731	0	0.005672738	0.001311368	0.000525332	0.002903532	0.001679928	0.001542599	0.00596619	0.004793228
Phascolarct	0	0	0	0	0	0	0	0	0	0
Plesiomona	0	0	0	0	0	0	0	0	0	0
Pluralibacte	0	0	0	0	0.000344387	0	0	0	0	0
Prevotella_	0	0.000328131	0	0	0	0	0	0	0	0
Prevotella_	0	0	0	0	0	0	0	0	0	0
Prevotella_	0	0	0	0	0	0	0	0	0	0
Prevotella_	0	0	0	0.375613379	0.292742613	0	0	0.451164729	0.009320815	0
Prevotella_	0	0	0.000527818	0	0	0	0	0	0	0
Prevotella_	0	0	0	0	0	0	0	0	0.004702217	0
Prevotella_	0	0	0	0	0	0	0	0	0	0
Prevotella_	0	0	0	0	0	0	0	0	0	0
Prevotella_	0	0	0	0	0	0	0	0	0	0
Prevotella_	0	0	0	0.24906628	0.15798148	0	0	0	0	0
Prevotella_	0	0	0	0	0	0	0	0	0	0
Proteobacte	0	0.000262925	0.008460487	0	0	0	0	2.18E-05	0.000329371	0
Proteus_mi	0	0	0	0	0	0	0	0	0	0
Proteus_pe	0	0	0	0	0.00047782	0	0	0	0	0
Pseudocitro	0	0	0	0	0	0	0	0	0	0
Pyramidoba	0	0	0	0	0.000148637	0	0	0	7.56E-05	0
Pyramidoba	0	0	0	0	0	0	0	0	0	0
Raoultella_	0	0	0	0	0.00134884	0	0	0	0	0
Raoultella_	0	0	0	0	0.002035112	0	0	0	0	0
Roseburia	0	0	0.001211999	0.002008457	0.005947798	0.006685873	0	0.005501097	0.000947805	0
Roseburia	0	0	0	0.000350545	0.006543448	0.002145023	0.001509384	0.0004065	0.000717055	0.00377323
Roseburia	0	0	7.66E-05	0.000432855	0.000480121	0.004162345	0.000122331	1.63E-05	0.000799773	0
Roseburia	0	0	0.003860891	0.004398163	0.000245762	0.000311703	0.000180946	0.001188799	0.000947405	0.00118943
Roseburia	0	0	0	0	0.004466925	0.000408504	0	0	0.003173186	0
Roseburia	0	0	0.000560331	0	0	0	0	0	0.001083834	0
Roseburia	0	0	0	0	0	4.94E-05	0	0	5.34E-05	0
Rothia_muc	0	4.40E-06	0	0.000530368	0	0	0.000167243	0	5.29E-05	8.95E-05
Ruminococ	0	0	0	0	5.30E-06	0	0.000144037	0	0	0.000966587
Ruminococ	0	0	0	0	2.31E-05	0.025805481	5.07E-05	0.0005656	0.001072132	0
Ruminococ	0	0	0	0	0	5.73E-05	0	0.004585297	0	0
Ruminococ	0	0	0	0	0	0	0	0	1.42E-05	0
Ruminococ	0	0	0	0	0	0	0	0	0	0
Ruminococ	0.0024579	0.015837805	0.001009216	7.59E-05	0	0	0.000398501	0	0	0.005762216
Ruminococ	0	0	0	0	0	0.00087191	0	0.0005887	0.000948005	0
Ruminococ	0	0	0	0	0	0	0	0	0	0
Ruminococ	0	0	0	0	0.006305588	0	0	0	0	0
Ruminococ	0	0	0	0	0.002339289	0	0	0	0	0
Ruminococ	0	0	0	0	0	0	0	0	0	0
Ruminococ	0.0018684	0	0.002727624	0.005769238	0.001195701	0.001187113	0.000130033	0	0.000109924	0
Ruthenibac	0.0022074	0	0.001437793	0	0.000372194	0.000289803	0.00102296	0	0.000373281	0.016417879
Sanguibact	0	0	0	0	0	0	0	0	0	0
Scardovia_	0	0	0.000563232	0	0	0	0	0	0	0
Sellimonas	0	0	0	0	0	0	0	0	0	0
Slackia_iso	0	0	0	0	0	0	0	0	0	0
Streptococ	0	0	0	0.000116415	0	0	0	0	0	0
Streptococ	0	0	0	3.21E-05	0	0	0	0	0	0
Streptococ	0	0	0	0	0	0	0.002431519	0.0005274	0	0
Streptococ	0	0	0	0	0	0	0	0	0	0
Streptococ	0	0	0	0	0	0	0	0	0	0
Streptococ	0	0	0	1.93E-05	0	0	0	0	0	0
Streptococ	0	0.000496247	0.000159766	9.23E-05	0.000730184	1.33E-05	0.000258666	2.08E-05	2.34E-05	0
Streptococ	0.0002065	0.000550252	0.000768817	0.000717692	0.001873672	1.00E-05	0.001197005	0.0002028	0.000153033	7.26E-05
Sutterella_	0.002800199	0	0	0.001570501	7.93E-05	0	0	0.001975899	0.000214146	0.000495696
Turicimonas	0	0.000131813	0.000566934	0	0	0	0	4.58E-05	0.00013883	0
Tyzzerella_	0.0001746	1.09E-05	0	0	0	0	0.000125232	0	0	0.000221143
Veillonella_	0	7.65E-05	0.000272912	0	0.00139165	0.001149013	8.57E-05	0	0.00018444	0
Veillonella_	0	0	0	0	8.95E-05	3.81E-05	0	0	0.000121926	0
Veillonella_	0	0	0	0	0.000502727	0.000119501	0	0	0	0
Veillonella_	0	0	0.000785424	0.000125616	2.07E-05	0.000555706	0	0	0.000177038	6.90E-06
Veillonella_	0	0	0	0	4.68E-05	2.08E-05	0	0	0	0
Victivallis_v	0	0	0	0	0.001254216	0	0	0	0	0
Weissella_c	0	0	0	0	0	0	0.000817508	0	0	0
Weissella_c	0	0	0	0	3.01E-05	0	0	0	3.53E-05	0

species	W53	W50	W49	W47	W41	W40	W34	W33	W3	W32
Absiella_do	0	0.000251454	0	0	0	0	2.22E-05	0	0	0
Acidaminoc	0	0	0.000163225	0	0	0	0	0	0	0
Acidaminoc	0	0	0	0	0	0	0	0	0	0
Acidaminoc	0	0	0.010446281	0	0	0	0	0	0	0
Actinobacul	0	0	0	0	0	0	5.38E-05	0	0	0
Actinomyce	0	0	0	0	0	0	0	0	0	0
Actinomyce	0	0	0	0	0	0	0	0	0	0
Actinomyce	0.000117804	0	0.000173726	0.0002042	0	0	0.000264721	0.00050181	1.00E-05	0
Actinomyce	0	0	0	0	0	0	0	0	0	0
Actinomyce	0	0	0	0.0001046	0	0	0.000310725	9.97E-05	0	0
Actinomyce	0	0	0	0	0	3.15E-05	4.93E-05	3.12E-05	0	0

Actinomyce	2.00E-06	0.000415855	0	1.10E-05	0	0	0.001847546	0	0	0
Actinomyce	0	0	0	0	0	0	0	0	0	0
Actinomyce	0	0	0	0	0	0	0	0	0	0
Actinomyce	0	0	0	0	0	0	0	0	0	0
Actinomyce	0	0	0	0	0	0	0	0	0	0
Actinomyce	0	0	0	0	0	0	0	0	0	0
Actinomyce	0	0	0	0	0	0	0	0	0	0
Adlercreutz	0	0	3.48E-05	0	0	0	0	0.000354107	0.000165004	0.001271099
Aeromonas	0	0	0	0	0	0	0	0	0	0
Aeromonas	0	0	0	0	0	0	0	0	0	0
Aeromonas	0	0	0	0	0	0	0	0	0	0
Agathobacu	0.010030742	0	0.000109917	0.0005104	0	0.001217557	0.003834103	0.003159661	0.000207504	0.000436934
Aggregatiba	0	0	0	0	0	0	0	0	0	0
Akkermansi	0	0	0.014203949	0	0	0	0	0.070453867	0	0
Alistipes_fi	0.006103208	0	0.048286506	0.0004529	0.010215101	0.045337317	0.007420487	0.007932654	0	0.014428621
Alistipes_in	0.000113704	0	9.96E-05	0	0	0	0	0	0.003520575	0
Alistipes_in	0	0	0	0	0	0	0	0	0	0
Alistipes_or	0	0	0	0	0	0	0	0	0	0
Alistipes_pu	0.001346746	0	0.369239466	0.004163501	0	0	0.062565149	0.17423628	0.214990779	0.11802387
Alistipes_sh	0.025121357	0	0.042547237	0.0010786	0	0.011150921	0.015178701	0.011034714	0.008615984	0.005204104
Allisonella_	0	0	0	2.64E-05	0	0	0	0	0	0
Anaeroglob	0	0	0	0	2.80E-06	0	0	0	0	0
Anaeromas	0	0	0	0	0	0	0	0	0	0
Anaerostipe	0.000409114	1.41E-05	0	0	0.0001535	0.000485523	0.000221918	0.000250605	0.000205204	0
Anaerotrun	0	0	0	0	0	0.000410419	0.000109209	0	7.97E-05	6.18E-05
Asaccharob	0	0	0	0	0	0	0	0.000115002	4.70E-05	0.000289522
Atlantibacte	0	0	0	0	0	0	0	0	0	0
Atopobium_	0	0	0	0	0	0	3.95E-05	0	0	0
Atopobium_	0	0	0	0	0	0	0	0	0	0
Bacteroides	0.002661391	0.001406063	0.011437631	0.007765902	0.0026089	0.011537939	0	0.005454106	0.021154051	0.005872356
Bacteroides	0	0	8.73E-05	0.0001155	0	0	5.10E-05	0.000566311	0	0.011172468
Bacteroides	0.001997668	0	0	2.12E-05	0	0.005767369	0	0	0	0
Bacteroides	0	0	0	0.008278502	0	0	0	0	0	0
Bacteroides	0	0	0	0	0	0	0	0.02369376	0	0
Bacteroides	0.019864177	0	0	0.0004323	0	0.023400393	0.000152312	0.004884895	0	0
Bacteroides	0	0	0.000970647	0	0	0	0	0	8.10E-06	0
Bacteroides	0	0	3.20E-06	0	0	0	0	0	0	0
Bacteroides	0	0	0	0	0	0	0	0	0	0
Bacteroides	0	0	4.96E-05	0.0010058	0.075565808	0	0.000219117	0.001456528	0.00092182	8.70E-06
Bacteroides	0	0	0	0	0	0	0	0	0	0
Bacteroides	0	0	0	0	0	0	0	0	0	0
Bacteroides	0	0	0.00185128	0	0	0	0.000158413	0.001097021	7.99E-05	0
Bacteroides	0.009266716	0.034562904	0.00707037	0.003437501	0.0037102	0.026380832	0	0	0.004184289	0.04452636
Bacteroides	0	0	0	0	0	0	0	0	0	0
Bacteroides	0.010948673	0.001161913	0	0.0001121	0.0021672	0	8.91E-05	0.001379027	0.002995964	0.005576333
Bacteroides	0.24398692	0.253734866	0.036715455	0.015743403	0	0.078248454	0	0	0.009186296	0
Bacteroides	0.004134341	0	0	0.000526	0	0	0	0	0.006244633	0
Bacteroides	0	0	0	0	0	0	0	0	0	0
Bacteroides	0	0	0.002793223	0	0	0	0	0	0	0
Bacteroides	0.157100757	0.07209473	0.029389447	0.002659001	0.480467748	0.042023763	0.184611803	0.140576327	0.010950233	0.005585234
Bacteroides	0.005400084	0	0.011047371	0.0011871	0.009405201	0.03233051	0.012729207	0.000679413	0.013900496	0.00682573
Bacteroides	0.204507474	1.79E-05	0.091311315	0.0009408	0.049576205	0.370254291	0.066849488	0.169422587	0.035248851	0.229067399
Bacteroides	0.098029143	0.478104317	0.01943394	0.023608205	0.237013624	0.096496506	0.361634705	0.051066791	0.083237673	0.198280706
Bacteroides	0.004606357	0	0.009578849	0.0003488	0.005875901	0.003410459	0.020424316	0.002682552	0.000767616	0.000611448
Barnesiella_	0.005631292	0	0.005368112	0.0004839	0	0	0.006913247	0.005398405	0.014228203	0.002800718
Bifidobacter	0	0	0	0	0	0	0.009031114	0	0	0
Bifidobacter	0.006493621	0	0	0.0009889	0	0	0	0	0.000108402	0
Bifidobacter	0.023099788	0	0	6.60E-05	0	0	0	0	0.000239205	0
Bilophila_wa	0.000928732	0	1.59E-05	0.0007937	0	0.002068197	0.001376809	0.000405508	0.005000807	0.001738235
Blautia_han	0	0.000430464	0	0	0	0	0	0	0	0
Blautia_obe	0	0	0.000241537	1.24E-05	0	0	0.00012771	0	0	0.000861767
Blautia_sp_	0	3.68E-05	0	0	2.14E-05	0	0	0	0	0
Blautia_wex	0.000443415	0	0	3.73E-05	0.0002659	0.001748182	0.00012351	0.000198604	1.90E-05	0.000319125
Butyricicocc	0	0.00192318	0	0	0	0	0	0	0	0
Butyricimon	0	0	0	0	0	0	0	0	0	1.39E-05
Butyricimon	0.001368047	0	0.009182289	0.0002355	0	0.001694679	0.003942912	0.00103982	0.002183947	0.008265242
Butyrivibrio_	0	0	0	0	0	0	0	0	0	0
Candidatus	0	0.000698028	0	0	9.20E-06	0	0	0	0	0
Catabacter_	0	0	0	0	0	0.000154607	0	0	0	0
Christenser	0	0	0	0	0	0	0	0	0	0
Citrobacter_	0	0.011917411	0	0	0	0	0	0	0	0
Citrobacter_	0	0.000990107	6.84E-05	0	0	0	0	0	0	0
Citrobacter_	0	0	0	0	0	0	0	0	0	0
Citrobacter_	0	0.003233184	0.000105116	0	0	0	0	0	0	0
Citrobacter_	0	0.001008719	0	0	0	0	0	0	0	0
Citrobacter_	0	0.009271188	0.000351553	0	0	0	0	0	0	0
Cloacibacill	0	0	7.31E-05	0	0	0	0	0	0	0
Clostridium_	7.13E-05	9.34E-05	0	0	0	0	0	0	0	0
Clostridium_	0.00058662	0	0	0	0	0.000530325	0	0	0	0
Clostridium_	0.000610921	0	0	0.0002156	0.0005375	0.003834779	7.25E-05	3.16E-05	0	0.000447135
Clostridium_	0	0	0	0	0	0	0	0	0	0
Clostridium_	5.90E-06	0	0	0	0.0003265	0.000142407	2.69E-05	0.000272005	0	0
Clostridium_	0	0.00011347	0	0	0.0008507	0.00128796	0	0	0	0
Clostridium_	0	0	0	0	0	0.00021861	0	0	0	0
Clostridium_	0	0	0	0	0	0	0	0	0	0
Clostridium_	0	0	0	0	0	0	5.12E-05	9.30E-06	0	0.00025732
Clostridium_	0	0	0	0	0	7.26E-05	0	9.40E-06	0	0
Clostridium_	0	0	4.22E-05	0	0	0	0	0	0	0
Clostridium_	0	0	0	0	0	0	0	0	0	0
Clostridium_	0	0	0	0	0	0	0.000401732	0.001458528	0	0.000148712

Clostridium_	0	0.008782888	0	0	0.013008001	0	0.000276422	0	4.50E-06	0
Clostridium_	0	0	0	0	0	0	0	0	0	0.00012361
Clostridium_	0	0	0	0	0	0	0	0	0	0
Clostridium_	0.000551919	0	0.000422864	0.0003078	0	0.015711334	0	0.000398408	0.000103702	0.000364428
Clostridium_	0	0.000115071	0	0	0	0	0	0	0	0
Clostridium_	0	0	0	0	0.0007461	0.000293214	0	0	0	0
Clostridium_	0	0	0	0	0	0	0	0	0	0
Collinsella_	0	0	0.000139921	0.002216	0	0	0	0	0	0
Collinsella_	0.003122106	0	0	0.0010741	0	0	0	0	0	0
Collinsella_	4.52E-05	0	0	4.11E-05	0	0	0	0	0	0
Comamona	0	0	0.000814823	0	0	0	0	0	0	0
Coprobacillu	0	0	0	0	0	0	0	0	0	0
Coprobacte	0	0	0	4.71E-05	0	0	0	0	0.000873219	0
Coprobacte	0.000157605	0	0.000189729	0	0	0.002502117	0	0.000352107	0	0
Coprococcu	0	0	0	0	0	0	0	0.000284806	0	0
Coprococcu	0.000247208	0	0	0	0	0	0	0.000347907	9.30E-06	0
Coprococcu	0	0	0	0	0	0	0	0	0	0.00115779
Corynebact	0	0	0	0	0	0	0	0	0	0
Corynebact	0	0	0	0	0	0	0	0	0	0
Desulfovibri	0	0	0	0	0	0	0	0	0	0
Desulfovibri	0	0	0.003342506	0	0	0	0	0	0	0
Desulfovibri	0	0	0	0.0006834	0	0	0	0	0	0.012649383
Dialister_inv	0	0	0	0	0	0	0	0	0	0
Dialister_su	0	0	0	0	0	0	0	0	0.000888219	0
Dielma_fast	0	4.82E-05	0	0	0	0	0	0	0	0
Dorea_form	0	0	0	0.0001092	0	0	0.000418433	0.00052371	6.82E-05	0.000146311
Dorea_long	2.56E-05	0	0	3.22E-05	0	0	0.000194015	0.000136803	8.76E-05	0.000295523
Eggerthella	0.001321445	0	0	0	0.0004608	0.002269406	0.001253999	0.000126502	0.000173604	0.00231688
Eisenbergie	0	0	0	0	0.0008021	0.000886541	0	0	0	0
Eisenbergie	0	0	0	0	0	0	0	0	0	0
Enterobacte	0	0	0	0	0	0	0	0	0	0
Enterobacte	0	0.023236356	0.00098905	0	0	0	0	5.82E-05	0	0
Enterobacte	0	0	0	0	0	0	0	0	0	0
Enterococcu	0	0	0	0	0	0	0	0	0	0
Erysipelatoc	0	0.001093771	0	0	0	0.000597228	0	0	0	0
Escherichia	9.48E-05	0.000901953	0.003447122	0.0003938	0.017529602	0.011922357	0.041754403	0.02476378	2.54E-05	0
Eubacterium	0	0.000295982	0	0	0	0	2.11E-05	0	0	0
Eubacterium	0	0	0.003167479	0	0	0	0.0075802	0.003315064	0.001777738	0.001687131
Eubacterium	4.76E-05	0	0	0	7.50E-06	0	0.000178214	1.78E-05	0	0.000100808
Eubacterium	0	0	0	8.77E-05	0	0	0.000452736	6.38E-05	0	0
Eubacterium	0.010870671	0	0.000109217	0.014222803	0	0	0.000155412	0	3.00E-06	0.000393031
Eubacterium	0	0	0.002341454	0	0	0	0.00215477	0	0	0
Eubacterium	0	0	4.12E-05	0	0	0	0	0	0	0
Eubacterium	0	0	0	0	0	0	0	0	0	0.006459802
Eubacterium	7.96E-05	0	0	0	0	0	0	0.00153203	0	0
Eubacterium	0	0	4.57E-05	0	0	0.010962012	0.01226907	0	0	0.000484838
Eubacterium	0	0	0	0	0	0	0	0.005065998	0	0.000320025
Eubacterium	0	0	0	0	0	0.000595928	0.000898771	1.65E-05	0	0
Faecalibact	0.010405755	0	0.018088837	7.34E-05	0	0.017602222	0.042180236	0.039333963	0.015988541	0.014791549
Firmicutes_	0	0	0	0	0	0	0	0	0	0.007098552
Firmicutes_	0	0	0	0	0	0.001541872	0	0	0	0
Firmicutes_	0	0	0.000354754	0	0	0	0	0	0	0
Firmicutes_	0	0	0	0	0	0	0	0	0	0
Firmicutes_	0.002080171	0	6.34E-05	0	0	0.00727304	0.001247399	0.000401008	0.000870319	0.007925816
Firmicutes_	0	0	0	0	0	0	0	0.000653613	0.000294606	0.000213717
Flavonifract	0.002962401	0.00034271	0	9.03E-05	0.010488101	0.010455988	0.0012591	0.000783415	0.000230605	0.010953451
Fusicatenib	0.003870932	0	0	0	0	0	0.00214967	0.003179062	0	0.000175914
Fusobacteri	0	0	0	0.006665401	0	0	0	0	0	0
Fusobacteri	0	0	0	0	0	0	0	0	0	0.002801818
Fusobacteri	0.000310511	0	0	0	0	0	0	0	0	0
Fusobacteri	0	0	0	0	0	0.001545372	0.000118909	0	0	0
Gardnerella	0	0	0	0	0	0	0.000166013	0	0	0
Gemella_sa	0	0	0	0	0	0	0	0	0	0
Gemmiger_	0	0	0	0.0001408	0	0	2.58E-05	0.000138803	3.87E-05	0.002085562
Gordonibac	0.00029951	0	0	0	9.54E-05	0.000826339	0.00038253	0.000176403	8.01E-05	0.003970108
Haemophilu	0	0	0	0	0	1.38E-05	0.003022239	0	0	0
Holdemane	0	0	0	0	0	0	0	0	0	0
Holdemania	0	0	0	0	0	0.000464822	0.000369129	5.15E-05	0	0.00051424
Hungatella_	0	4.81E-05	0	0	4.67E-05	0.000794137	0.00012141	0	0	0
Intestinibact	0	0	0	0	0	0.000335716	0	0	0	0.000461336
Intestinimor	0.001095037	0	0	0	0	0.012106765	0	0	0.001024922	0.000295023
Klebsiella_a	0	8.36E-05	0	0	0	0	0	0	0	0
Klebsiella_r	0	0.00014699	0	0	0	0	0	0	0	0
Klebsiella_c	0	9.00E-05	9.86E-05	0	0	0	0	0	0	0
Klebsiella_p	0	0.029980893	0.01070502	0	0.007693401	0	0.025948953	0.104089419	4.58E-05	0.001732635
Klebsiella_c	0	0.012836175	0.00211772	0	0.0018955	0	0.002167571	0.010853611	0	0.00038933
Klebsiella_v	0	0.007891842	0.003113171	0	0.0021729	0	0.008539475	0.025312191	0	0.000292823
Kluyvera_as	0	0.002211657	0.000113717	0	0	0	0	0	0	0
Kluyvera_cr	0	3.25E-05	0.000206031	0	0	0	0	0	0	0
Kluyvera_ge	0	0	7.30E-06	0	0	0	0	2.34E-05	0	0
Lachnospira	0	0.002306415	0.000162125	0.0005148	0	0	0	0	0	0
Lachnospira	0	0.000112369	0	0	0	0	0.001782541	0	0	0
Lactobacillu	0	0	0.000373056	0	0	0	0	0	0	0
Lactobacillu	0	0	4.19E-05	0	0	0	0	0	0	0
Lactobacillu	0	0	0	0	0	0	0	0	0	0
Lactococcus	0	0	0	0	0	0	0	0	0	0
Lactococcus	0	0	0	0	0	0	0	0	0	0
Lawsonibac	0	0	7.57E-05	0	0	0	1.43E-05	0	2.57E-05	0
Leclercia_a	0	0.00027677	0	0	0	0	0	0	0	0
Megamonas	0	0	0	0.017016203	0	0	0.002301782	0.002856255	0	0

Megamonas	0	0	0	0.005747301	2.86E-05	0	0.000646251	0.001801035	0	0
Megamonas	0	0	0	0	0	0	0	0	0	0
Megasphae	0	0	0.002940945	0.052568611	0	0	0	0.000475609	0	0
Megasphae	0	0	0	0	0.0001771	0	0.000455536	0	0	0
Methanobre	0	0	7.48E-05	0	0	0	0	0	0	0
Mitsuokella_	0	0	0	0	0	0	0	0	0	0
Mitsuokella_	0	0	0.176517507	0	0	0	0	0	0	0
Mixta_calida	0	0	0	0	0	0	0	0	0	0
Monoglobus	0	0	0	0	0	0.000400219	0	0	0	0
Morganella_	0	0	0	0	0	0	0	0	0	0
Odoribacter	0.001232442	0	0.015250107	0.0003204	0	0.004013987	0.005849763	0.004362785	0.004825503	0.001256198
Oscillibacte	0.01027395	0	0.005608149	0.0001015	0	0.000949144	0	0.008654568	0.002634156	0.137700499
Oscillibacte	2.20E-06	0	0.000104816	0	0	0	0	0.00103732	0	0.020044357
Oxalobacte	0	0	0	0	0	0	0	0	0.000131703	0
Paeniclostri	0	0	0	0	0	0	0	0	0	0
Parabactero	0	0	0	0	0	0	0	0	0	0
Parabactero	0.010914572	0.001624697	0.005214189	0.006283101	0.0035046	0.03447411	0.007797317	0.002166242	0.011837452	0.026268541
Parabactero	0	0	0.00237966	0	0	0.000914343	0.000693855	0.000167803	0	0.00218227
Parabactero	0	0	0	0	0	0	0	0	0	0
Parabactero	0	0	0	7.50E-06	0	0	0	0.000224204	0	0
Parabactero	0.01262263	0	0.003684457	0.003773301	0	0.005635163	0.010863759	0.001237224	0.007473359	0.001189492
Parabactero	0	0	0	0	0	0	0	0	0	0
Paraprevote	0.000242908	0	0	0.0013645	0	0	0.001567224	0	0	0.001969653
Paraprevote	0.006931136	0	0.000301446	0.005421501	0	0.03041272	0.004448752	0	0	0.006617614
Parasuttere	0.024585638	3.87E-05	0	0	0.0027712	0.000503924	0.00089077	0.001301725	0.006250133	0
Pediococcu	0	0	0	0	0	0	0	0	0	0
Phascolarct	0.001252743	0	0	0	0.0049277	0.017711427	0.002581904	0.004423586	0.001850139	0
Phascolarct	0	0	0.004459375	0.05172081	0	0	0	0	0	0.044123928
Plesiomona	0	0	0	0	0	0	0	0	0	0
Pluralibacte	0	0	0	0	0	0	0	0	0	0
Prevotella_l	0	0	0	0	0	0	1.91E-05	0	0	0
Prevotella_l	0	0	0.000144422	0	0	0	0	0	0	0
Prevotella_c	0	0	0	0	0	0	0	0	0	0
Prevotella_c	0	0	0	0.752644451	0	0	0	0	0.499189433	0
Prevotella_c	0	0	0	0	0	0	0	0	0	0
Prevotella_s	0	0	0	0	0	0	0	0	0	0
Prevotella_s	0	0	0	0	0	0	0	0	0	0
Prevotella_s	0	0	0	0	0	0	0	0	0	0
Prevotella_s	0	0	0	0	0	0	0	0	0	0.002119165
Prevotella_s	0	0	0	0	0	0	0	0	0	0
Prevotella_t	0	0	0	0	0	0	0	0	0	0
Proteobacte	0.002401782	0.001212944	0	0	0.050945605	0	0.006689329	0.00414258	0.003338171	0
Proteus_mi	0	0	0.002339254	0	0	0	0	0	0	0
Proteus_pe	0	0	0	0	0	0	0	0	0	0
Pseudocitro	0	0.000640393	0	0	0	0	0	0	0	0
Pyramidoba	0	0	0.003220287	0	0	0	0	0	0	0
Pyramidoba	0	0	9.10E-05	0	0	0	0	0	0	0
Raoultella_	0	0.000118172	0	0	0	0	0	0	0	0
Raoultella_	0	0	0	0	0	0	0	0	0	0
Roseburia_	0	0	0.000349553	2.65E-05	0	0.000959745	0.00416633	0.045761588	0	0.003808196
Roseburia_	0.044354913	0	0.000698506	0	0	0.031401866	0.00531032	0	0.000741716	0
Roseburia_	0	0	0	0	0	0	0.019016304	0.010655407	0	0
Roseburia_	0	0	9.33E-05	0	0	0	0	0.003330765	0	0.000470437
Roseburia_	0	0	0	0	0	0	0	0	0	0
Roseburia_	0	0	0	0	0	0	0.002329084	0	0	0
Roseburia_	0.000130504	0	0	0	0	0	0	9.59E-05	0	0
Rothia_muc	4.59E-05	1.71E-05	0	0	0	0	0.000166113	5.43E-05	0	0
Ruminococ	0	0	0.000880633	0	0	0.000236911	0	3.00E-06	0	0
Ruminococ	0.001254743	0	0	0	0	0	0	0	0	0
Ruminococ	0	0	0.000312147	0	0	0.003606268	0	0	0	0
Ruminococ	0.000101403	0	0	0	0	0	0	0.000919918	0	0
Ruminococ	0	0	0	0	0	0	0	0	0	0
Ruminococ	0.000337312	0.002651227	0	0	0.0034252	0.003746475	0.001826344	0.001379527	0	0
Ruminococ	0	0	0.000374257	0.0002164	0	0	0	0.000412308	4.92E-05	0
Ruminococ	0	0	0	0	0	0	0	0	0	0
Ruminococ	0	0	0	0	0	0	0	0	0	0
Ruminococ	0	0	0	0	0	0	0	0	0	0
Ruminococ	0	0	0	0	0	0	0	0	0	0
Ruminococ	0.000352012	0.00076627	6.45E-05	3.50E-06	0	0	0	0.000338607	0	0.000105308
Ruthenibac	0.000215407	2.47E-05	0.000239736	0.0001087	0	0.005748268	0.000767861	0.001247524	0.000183204	0.008461057
Sanguibacte	0	0	0.000243837	0	0	0	0	0	0	0
Scardovia_	0	0	0	0	0	0	0	0	0	0
Sellimonas_	0	0	0	0	0	0	0	0	0	0
Slackia_iso	0	0	0	0	0	0	0	0	0	0
Streptococc	0	0	0	0	0	0	0	0	0	0
Streptococc	0	0.000137284	0	0	0	0	0	0	0	0
Streptococc	0	0	0	0	0	0	0	0	0	0
Streptococc	0	0	0	0	0	0	0	0	0	0
Streptococc	0	0	0	0	0	0	0	0	0	0
Streptococc	0	2.83E-05	0	0	0	0	0	0	0	0
Streptococc	0	0.000254156	0	0.0001	0	1.23E-05	0.000292123	9.81E-05	0	0
Streptococc	0	0.00435167	0	0.0003008	0	0.000250212	0.001428613	0.000483109	0	0
Sutterella_	0	0	8.90E-06	8.00E-06	0	0	0	0	0	4.46E-05
Turicimonas	0.000171706	3.81E-05	0	0	2.46E-05	0	0.000189715	0.000168403	0.000380808	0
Tyzzerella_	0	0	0	0	0	8.66E-05	0.000152812	3.88E-05	0	0
Veillonella_	0	0.001131894	7.73E-05	0	0	0	0.000471537	2.92E-05	0	0
Veillonella_	0	0	0	0	0	0	3.53E-05	0	0	0
Veillonella_	0	9.81E-05	0	0	0	0	0.000220417	0	0	0
Veillonella_	0	0.02451484	0.000183428	0	0.0004759	0	0.000795363	0	0	0
Veillonella_	0	0	0	0	0	0	1.91E-05	0	0	0

Victivallis_v	0	0	0.001078363	0	0	0	0	0	0	0.001342404
Weissella_c	0	2.34E-05	0	0	0	0	0	0	0	0
Weissella_c	0	1.37E-05	0	0	0	0	0	0.000180704	0	0
species	W31	W30	W29	W28	W27	W26	W25	W16	W13	W12
Absiella_do	0.000283242	0.0005244	0	0	0	0	0	0	0	2.05E-05
Acidaminoc	0	0	0	0	0	0	0	0	0	0
Acidaminoc	0	0.005617001	0.06617137	0	0	0	0	0	0	0
Acidaminoc	0	0	0	0	0	0	0	0	0	0
Actinobacul	0.000172748	0	8.22E-05	0	0	0	0	0	0	2.08E-05
Actinomyce	0	0	0	8.22E-05	0	0	0	0	3.81E-05	0.00012343
Actinomyce	0	0	0.000150709	3.47E-05	0	0	0	0	0	0.000213352
Actinomyce	0.000530754	0.0001219	4.51E-05	0.000517275	0.000347416	0.000193232	9.64E-05	0	0.000157429	2.21E-05
Actinomyce	0.000135716	0	1.19E-05	3.53E-05	0	0	0	0	0	4.58E-05
Actinomyce	0.000211381	2.51E-05	0	0.000101534	2.71E-05	0	1.11E-05	0	0.000136525	0
Actinomyce	1.78E-05	0	0	7.28E-05	0	0	0	0	0	0
Actinomyce	0.001293307	4.00E-06	4.86E-05	0	5.05E-05	0.000109918	0.00039198	7.40E-06	0.000262148	0
Actinomyce	0	0	7.97E-05	0.000548486	0	2.43E-05	0	0	0	0.000457112
Actinomyce	0	0	0	3.92E-05	0	0	0	0	9.18E-05	9.29E-05
Actinomyce	1.26E-05	0	0	0.000173059	0	0	0	0	0	0
Actinomyce	0.000708707	0	0.000174011	0	0.000136906	0.000545591	0	0	0	0.001935774
Actinomyce	0.000124507	0	0	9.20E-06	0	0	0	0	0	0
Actinomyce	5.85E-05	0	0	0	9.44E-05	0	0.000111623	0	0	0
Adlercreutzi	0.000777766	0.0006466	0.000185411	0.001025547	0.000502023	0.001632271	0.017892764	0.0005988	0	0
Aeromonas	0	0	0	0.00026659	0	0	0	0	0	0
Aeromonas	0	0	0	0	0	0	0	0	0	0
Aeromonas	0.002046151	0	0	0	0	0	0	0	0	0
Agathobacu	0.002101098	0	0.001064765	0.000624811	0.001628775	0.003019501	0.000104621	0.0012375	0.00065732	0.009672668
Aggregatiba	0	0	0	0	0	0	0	0	0	0
Akkermansi	0	0.0028499	0.000389124	0	0.006469898	0.003399564	0.127288669	6.73E-05	0.069585371	0.148431136
Alistipes_fin	0.001582955	0.076599608	0.003505216	0.024148472	0.012136158	0.019752577	0.029690881	0.0016265	0.047575463	0.010815748
Alistipes_in	0	0.005577601	0.000347221	0	0.000685932	0.013694772	5.20E-06	0.0008802	0.00708419	0
Alistipes_in	0	0	0	0	0	0.000802333	0	0	0	0
Alistipes_or	0	0	0	0	0	0	0	0	0	0
Alistipes_pu	0.114514902	0.020844502	0.154397295	0.035930059	0	0.066077062	0.10332066	0.1469426	0.272830282	0.108624191
Alistipes_sh	0.003300425	0	0.015019424	0.075897584	0.004848423	0.038065115	0.035917056	0.0273083	0.029830032	0.002944021
Allisonella_	1.57E-05	0	0	0	0	0	0.000109322	0	0	0.000125831
Anaeroglob	6.26E-05	0.0002757	0	0	0	0	0	0	0	0
Anaeromas	0	0	0	0	0	0	0.00024385	0	0	0
Anaerostipe	0.000367114	0	0	0	0.001258958	0	0.000656734	0.0015683	0	0.000905822
Anaerotrun	0	0.0024629	0	0.000337814	0	0	0	3.72E-05	0.003712876	0.000999145
Asaccharob	0.000192164	0.0001757	3.77E-05	0.000330112	0.000127206	0.000464377	0.004368295	0.0001529	0	0
Atlantibacte	0	0	0	0	0	0	0	0	0	0
Atopobium_	0.000122905	0	6.75E-05	0	0	0	0	0	0.000139525	1.64E-05
Atopobium_	0.000237203	0	0	0	0	0	0	0	0	0
Bacteroides	0.003931264	0.051677205	0.003218298	0.0132993	0	0.011411993	0.005966722	0.0113768	0.002729097	0.001031853
Bacteroides	0	0	0	0	0	0	0.011435042	1.46E-05	0.000671422	0.002437597
Bacteroides	0	0	0	0	0.0021673	0.000403867	0	0.0016283	0	0.000437607
Bacteroides	0.050743026	0	0.084973526	0.009805818	0	0.002396198	0	0	0	0
Bacteroides	0	0	0	0	0	0	0	0	0	0
Bacteroides	0	0	0	0	0.001108751	0.004693579	0	0.0302968	0	0.00224475
Bacteroides	0	0	0	0.009826525	0.141328201	0.000779929	0.02036067	4.10E-06	0	2.27E-05
Bacteroides	0	0	0	0	0	0	0	0	0	0
Bacteroides	0	0	0	0	0	0	0	0.0052275	0	0
Bacteroides	0.010247069	0	0	0.001108075	0.018860368	1.18E-05	0.002369785	0.000718	0.00148507	0.000964036
Bacteroides	0	0.009465901	0	0.0002967	0.002581319	0	0.03188403	0.0025121	0	0.003397632
Bacteroides	0	0	0	0	0	0	0.000110723	0.000524	0	0
Bacteroides	0	0.194889819	0.000105006	0.007043784	0.000611428	0.000521987	0.002201251	4.36E-05	0.000236143	0
Bacteroides	0.014689671	0	0	0.006791498	0.028946032	0.003263241	0.007968032	0.009219	0.009630054	0
Bacteroides	0	0	0	0	0	0	0	0	0	0
Bacteroides	0.000626736	0.011981301	0.039697841	0.001246822	0.013435318	0.00669011	0	0.0036854	0.000158729	0.007583256
Bacteroides	0.133359529	0	0.040787208	0	0	0.007025366	0	0.2020629	0	0
Bacteroides	0	0	0	0	0	0.008414796	0.000981601	0	0.00285442	0
Bacteroides	0	0	0	0	0	0	0	0	0	0.003190981
Bacteroides	0.000381727	0	0	0	0	0	0.00083037	0	0	0
Bacteroides	0.024874988	0	0.060588926	0	0	0	0.001767862	0.0920317	0.135767423	0.243826489
Bacteroides	0.002179665	0.008208901	0.001979022	0.00780054	0.005117035	0.004682677	0.000940393	0.0003345	0.022389877	0.001972283
Bacteroides	0.011397354	0.368802837	0.044215719	0.059469124	0.139035196	0.037028543	0.025369896	0.0755475	0.241567089	0.057610403
Bacteroides	0.072868461	0.10065481	0.082981703	0.235621234	0.471657796	0.005881576	0.041002697	0.0901402	0.021194359	0.126160684
Bacteroides	0.002312479	0	0.00259606	0.001310143	0.006166284	0	0.00126956	0.000125	0	4.51E-05
Barnesiella_	0.001625891	0	0	0	0	0.010160186	0.024485715	0.0153256	0.001073996	0.00559637
Bifidobacter	0.001052701	0	0	0.001514112	0	2.07E-05	0	0	0	0.000352486
Bifidobacter	0.001452143	0.0011766	0.001641701	0.006071455	0	0	0.065031318	9.83E-05	0	0
Bifidobacter	0.002721629	0.0003059	0.005719852	0	0	0	0	0.0113929	2.06E-05	0
Bilophila_wa	0.001719472	0.0010938	0.007653771	0.002782242	0.002280305	0.000495082	0.000637931	0.0023178	0.002607475	0.000957834
Blautia_han	0	0	0	0	0	0	0	0	0	0
Blautia_obe	0.000337289	0	0	0	3.60E-06	0	0.002326176	0.0005677	0	0
Blautia_sp_	0	0	0	0	0	0	0	0	0	0.001244105
Blautia_wex	0.001154988	0	0	2.60E-06	0.000167008	0	0	0.0002062	0	0.002253852
Butyricicocc	0	0	0	0	0	0	0	0	0	0
Butyricimon	0.000294052	0	0	0	0	0.001661276	0.000638831	0.0001453	0	0
Butyricimon	0.0003509	0	0	0.000966227	0	0.006575691	0.001988907	0.0003602	0.000184234	0.000789793
Butyrivibrio_	0	0	0	0	0	0.002753057	0	0	0	0
Candidatus_	0	0	0	0	0	0	0	0	0	0
Catabacter_	0	0	0	0	0	0	0	0	3.94E-05	0
Christenser	0	0	0	0	0	0	0.000205342	0	0	0
Citrobacter_	1.17E-05	0	2.36E-05	0	0	5.25E-05	0	0	0	0
Citrobacter_	8.47E-05	0	0	0	0	0	0	0	0	0
Citrobacter_	8.94E-05	0	0	0	0	0	0	0	0	0
Citrobacter_	5.97E-05	0	0	0	0	0	0	0	0	0

Citrobacter_	2.27E-05	0	0	0.000116539	5.00E-05	0	2.58E-05	0	0	0
Citrobacter_	6.14E-05	0	0	0	0	0	0	0	0	0
Cloacibacillu	0	0	0	0	0	0.000477579	0	0	0	0
Clostridium_	0	0.0001149	0	0	0	0	0	0	0	5.50E-06
Clostridium_	0	0.0006262	0	0.000298801	0.000114605	0	1.83E-05	0	2.14E-05	0.00171762
Clostridium_	0.000111195	0	0	0	0.000148207	0	0.001747258	0	0.000374768	0.00094153
Clostridium_	0	0	0	0	0	0	9.73E-05	0	8.83E-05	0
Clostridium_	0.0001166	0.0001971	0	0	5.39E-05	0	0	0	5.28E-05	0.000320278
Clostridium_	0	0.0007316	0	0	0	0	0	0	0	0.000218153
Clostridium_	6.01E-06	0	0	0	0	0	0.000119624	0	0	0
Clostridium_	0	0	0	0	0	0	0.000165934	0	0	0
Clostridium_	0	3.80E-06	0	2.20E-06	0	0	0	0	6.09E-05	0.000198949
Clostridium_	0	0	0	0	0	0	0	0	0.000315657	1.83E-05
Clostridium_	0.00143683	0	0	0	0	0	0	0	0	0
Clostridium_	0	0	0	0	0	0.000203934	0	7.27E-05	0	0
Clostridium_	0	0	0	0	0	0	0	0	0	0
Clostridium_	0	0.0003364	0	0	0	0	0	0	0.001510075	0.000461713
Clostridium_	0	0	0	0	0	0.011840764	0.000209443	0	0	0
Clostridium_	0	0	0	0	0	0.064018221	0.002084127	0	0	0
Clostridium_	0.008184404	0	0	0.000211572	0.000950244	0.001337222	0.001201546	0.0003597	0.005842264	0
Clostridium_	0	0	0	0	0	0	0	0	0	0
Clostridium_	2.90E-05	3.52E-05	0	0.001318146	0	0	0.000988302	0	0	0.000267465
Clostridium_	0	0	0	0	0	0	0	0	0	0
Collinsella_	0.013821528	0.017006102	0.012639377	0.021644925	0	0.005986593	0.063385281	0.0250352	0	0
Collinsella_	5.37E-05	0	0	0	0	0	0	0.0015574	0.012162815	0
Collinsella_	0.000331584	6.97E-05	1.52E-05	1.99E-05	0	4.70E-06	0.000168935	8.51E-05	0.000364966	0
Comamona	0	0	0	0	0	0.000589298	0	0	0	0
Coprobacillu	0	0	0	0	2.08E-05	0	0.000428988	0	0	0
Coprobacte	7.55E-05	0	0.000640339	0	0	0	9.32E-05	0.0004524	0	0.035755753
Coprobacte	0	0	0	0	0	0.000708017	0.001598027	0	0	0
Coprococcu	0	7.71E-05	0	0	0	0	0.000792062	5.44E-05	0	0
Coprococcu	0.006380961	0	6.83E-05	0	0	0.000149325	0.000410684	0	0	0
Coprococcu	0	0	0	0	0	0	0	0	0	0
Corynebact	0	0	0	0	0	0	0	0	0	0
Corynebact	0	0	0	0	0	0	0	0	0	0
Desulfovibri	0	0	0	0	0	0	0.001449197	0	0	0
Desulfovibri	0	0	0	0	0	0.008785858	0	0	0.011579809	0.022480503
Desulfovibri	0.003752511	0	0	0	0	0	0	0	0	0
Dialister_inv	0	0	0	8.72E-05	0	0	0.000128826	0	0	0
Dialister_su	0.004318296	0.0043067	0.014904117	0.00115299	0	0	0	0	0	0
Dielma_fast	1.98E-05	0	0	1.36E-05	0	0	0	0	0	0
Dorea_form	0.0030385	0	0	6.72E-05	5.77E-05	2.81E-05	0	0.0004993	0	0
Dorea_long	0.003000268	0	5.80E-06	0.000113438	0.000197909	0.000174329	0.00112183	0.0010992	0	0.000112227
Eggerthella	2.97E-05	0.0005102	0	0.001573933	0.000277913	0	0.001750859	0	0.00027625	0.001942676
Eisenbergie	0	0	0	0	0	0	0	0	0	0
Eisenbergie	0	0	0	2.44E-05	0	0	0.000756555	0	0	0.000453511
Enterobacte	0.000487717	0	7.42E-05	0.000130844	0	0	0.000276157	0	0	0
Enterobacte	0.000416657	0.0001266	0.019063972	0.006100865	0	0.000149825	0.000344971	0.0009545	0.000173332	0
Enterobacte	0.000230097	0	0.000209413	7.12E-05	0	0	0	0	0	0
Enterococci	0	0	0	0	0	0	0	0	0	0
Erysipelatoc	0.000513339	1.86E-05	0	0	0	0	0.001103926	0	0	0.002929717
Escherichia	0.010758807	0.0037129	0.002507254	0.061757699	0.015362807	0.00012302	0.077544281	0.0263059	0.003814695	0.000100325
Eubacteriur	0.000207978	0.0004339	0	0	0	0	0	0	0	5.60E-06
Eubacteriur	0.010194424	0.002802	0.001889416	0.000663725	0.00022781	0.001059576	0.005545836	0.0016986	0.000336661	0
Eubacteriur	0.001342949	5.52E-05	2.59E-05	0	0.000146007	0	0.0004901	0.0005824	6.10E-06	0.000171842
Eubacteriur	0	0	0.000286018	0	7.21E-05	0	3.11E-05	0.0001251	0	0
Eubacteriur	0	0	1.80E-06	0.001539121	0.006069279	0.003356457	0	0	0.000614912	0.07663386
Eubacteriur	0.000213483	0	0	0.001376466	0	0.000141023	0.004316084	1.99E-05	0.00334761	0.000923626
Eubacteriur	0	0	0	0	0	0	0.000491401	0.0133584	0	0
Eubacteriur	0.003194734	0	7.58E-05	0	0	0	0	0.000455	0	0
Eubacteriur	0	0	0	0	0.000157807	0	0	8.40E-06	0	0
Eubacteriur	0.00317822	3.00E-06	0	0	0.000351416	2.81E-05	0	0.0009755	0.000552101	0.005703396
Eubacteriur	0	0.010858901	0	0	0	0.000190232	0.003882695	0	0	0.00016164
Eubacteriur	6.11E-05	0	1.20E-05	7.49E-05	3.33E-05	0	0	1.93E-05	0	0
Faecalibact	0.033492263	0.0026877	0.003707528	0.083057207	0.004298198	0.01675758	0.005411308	0.0479476	0.000703328	0.013457794
Firmicutes_	0	0	0	0	0	0.009346851	0.006258082	0	0	0
Firmicutes_	0	0	0	0	0	0	0	0	0	0
Firmicutes_	0	0	0	0	0	0.004927718	0	0	0	0
Firmicutes_	0	0	0	0.000168057	0	0	5.25E-05	0	0	0
Firmicutes_	0.009496327	0.010256401	0.005458336	0.031712131	0.002482714	0.001348024	0.001724453	0.0036064	0.013145794	0.003862446
Firmicutes_	0	0	0	0	0	0.001490047	0.000678039	0	0.000139825	0
Flavonifract	0.003076333	0.0005588	0.000879154	0.049454535	0.000473422	0	0.004536729	0.0001924	0.003030352	0.008713533
Fusicatenib	0.006926928	0.0002688	4.37E-05	0.000955223	0.000496623	5.41E-05	0.006891511	0.0081288	9.91E-05	0
Fusobacteri	0	0	0	0	0	0	0	0	0.005752448	0
Fusobacteri	0	0	0	0	0	0	0	0	0	0
Fusobacteri	0	0	0	0	0	0	0	0	0	0.000625453
Fusobacteri	0	0	0	0	0	0	0	0	0	0
Gardnerella	7.21E-05	0	0	0	0	0	0	0	0	0
Gemella_sa	0.000556276	0	0	0	0	0	0	0	0	0
Gemmiger_	0.000260023	0	0.000103506	3.78E-05	0	0.001334521	0.001613831	0.0003469	3.79E-05	0
Gordonibac	4.83E-05	0.0004448	4.99E-05	0.00171508	9.32E-05	2.90E-05	0.001800569	0	0.000818449	0.000961935
Haemophilu	0.002409362	0	0	0	0	1.47E-05	0	0	0	5.56E-05
Holdemane	0	0	0.000125308	0	0	1.55E-05	0	0	0	0
Holdemania	9.64E-05	0	0	0.000311705	0	5.55E-05	0.001028111	4.47E-05	0	0
Hungatella_	0	0.0001761	0	0.000612407	9.73E-05	0	0	0	0.000213039	0.000295672
Intestinibact	0.000257721	0	0	4.86E-05	0.000130006	0	0.000104821	0	0	0
Intestinimor	0	0.0003137	0	0.00274873	4.41E-05	0.000116419	0.001051215	0	0	0.002315567
Klebsiella_a	0.000331484	0	4.41E-05	0	0	0	0	0	0	0
Klebsiella_r	0	0	0	0	0	0	0	0	0	0
Klebsiella_c	0	0	0.000144409	0	0	0	0	0	0	0

Klebsiella_p	0.050507624	0.0030569	0.157793704	0.051186622	0.017130388	0.025647155	0.0170901	4.78E-05	0.006183926	0.000349386
Klebsiella_q	0.004562905	0.0004007	0.019304687	0.015224352	0.00194899	0.004126685	0.001398786	0	0.000784043	0
Klebsiella_v	0.011704317	0.0004117	0.036863167	0.011157576	0.024428924	0.005735051	0.002520116	0	0.000978078	7.06E-05
Kluyvera_as	0	0	0	0	0	0	0	0	0	0
Kluyvera_cr	0	0	0	0	0	0	0	0	0	0
Kluyvera_ge	0	0	0	0	0	0	0	0	0	0
Lachnospira	0	0	0	0	0.007488644	0	0.003333783	0.008483	0.0005494	0.000412001
Lachnospira	0.00142532	0	0	0	0	0	0	0	0	0
Lactobacillu	0	0	0	0	0	0	0	0	0	0
Lactobacillu	0	0	0	0	0	0	0.002795973	0	0	0
Lactobacillu	0	0	0	0	0	0	0	0	0	0
Lactococcus	0	0	0	0	0	0	0	0	0	0
Lactococcus	0	0	1.85E-05	0	0	0	0	0	0	0
Lawsonibac	0	0	0	0.000242982	0	0	0	0	0	0.00036789
Leclercia_a	0	0	0	3.51E-05	0	0	0	0	0	0
Megamonas	0.018132618	0.0008283	0.002257539	0	0	0	0	0.0012451	5.83E-05	0
Megamonas	0.000434172	0	6.90E-05	0	0	0	0	0.0006711	5.63E-05	0
Megamonas	0	0	0	0	0	0	0	0.0024307	0	0
Megasphae	0.003495992	0.0033105	0.016838736	0.00159574	0	0	0	0.0221073	3.02E-05	0
Megasphae	0.00061933	0	0	0	0	0	0	0	0	0
Methanobre	0	0	0	0	0	0.000202334	0	0	0	0
Mitsuokella_	0	0	0	0	0	0	0	0	0	0
Mitsuokella_	0	0	0	0	0	0	0	0	0	0
Mixta_calida	0	0	0	0	0	0	0	0	0	0
Monoglobus	0	0	0	0	0	0	0	0	0	0
Morganella_	0	0	0	0	0	0	0	0	0	0.000127331
Odoribacter	0.002111307	0.0003982	0.000546034	0.003684047	0	0.003803231	0.007250585	0.0033394	0.00153488	0.002186935
Oscillibacte	0.015177389	0.008660701	0.003430311	0.036632396	0.004296998	0.017064331	0.003168149	0.0145014	0	0
Oscillibacte	0	0	4.20E-06	0	1.90E-06	0.001934021	0.095383935	0.0008952	0	0
Oxalobacter	0	0	0	0	0	0	0	0	0.000199236	0
Paeniclostri	0.0001173	0	0	0	0	0	0	0	0	0
Parabactero	0	0	0	0	0	0.000126721	0	0	0	0
Parabactero	0.007859426	2.84E-05	0.011452804	0.013294299	0.006181384	0.008634232	0.022477703	0.022368	0.017494586	0.021052654
Parabactero	0	0	7.32E-05	0	0	0.001401432	0	0	0	0
Parabactero	0	0	0	0	0	0	0	0	0	0
Parabactero	0	0	0	0	0	2.40E-05	0	0	0.001020586	0.002267855
Parabactero	0.010547226	0	0.013140808	0.038352078	0.006252588	0.005494612	0.029994343	0.0109382	0.009176471	0.004782871
Parabactero	0	0	0	0	0	0	0	0.0006866	0	0
Paraprevote	0	0	0	0	0.001509469	0	0	0	1.61E-05	0
Paraprevote	0.014660346	0	0	0.001278933	0.014414763	0.001409534	0.000152331	0	0.000846054	0
Parasuttere	0.044400098	0.008839601	0.007397555	0.012952383	0	0.000152625	0.003533724	0	0	0
Pediococcu	0	0	0	0	0	0	0	0	0	0
Phascolarct	2.21E-05	0.0003733	0	0.000193766	0.001620675	0	0.002155942	0.0031132	0.003784789	0
Phascolarct	0	0	0	0	0	0.010782289	0	0	0	0
Plesiomona	0.038766576	0	0	0	0	0	0	0	0	0
Pluralibacte	0	0	0	0	0	0	0	0	0	0
Prevotella_	0.000221389	0	0	0	0	0	0	0	0	0
Prevotella_	0	0	0	0	0	0	0	0	0	0
Prevotella_	0	0	0	0	0	0	0.000264054	0	0	0
Prevotella_	0	0	0	0	0	0.475494885	0	0	0	0
Prevotella_	0	0	0	0	0	0	0	0	0	0
Prevotella_	0	0	0	0	0	0	0	0.0088244	0	0
Prevotella_	0.028145987	0	0	0	0	0	0	0	0	0
Prevotella_	0	0	0	0	0	0	0	0.0019769	0	0
Prevotella_	0	0	0	0	0	0	0	0	0	0
Prevotella_	0	0	0	0	0	0	0	0	0	0
Prevotella_	6.67E-05	0	0	0	0	0	0.000456093	0	0	0
Proteobacte	0.002445193	0.014278201	0.03950913	0.009560135	0	0	0.000127826	0	0	0
Proteus_mi	0	0	0	0	0.000280913	0	0	0	0	0
Proteus_pe	0	0	0	0	0	0	0	0	0	0
Pseudocitro	0	0	0	0	0	0	0	0	0	0
Pyramidoba	0	0.013776901	0	0.001149589	4.87E-05	6.49E-05	0	0	0	0
Pyramidoba	0	0.0003389	0	1.18E-05	0	0	0	0	0	0
Raoultella_	0	0	0	0.000120841	0	0	0	0	0	0
Raoultella_	0	0	0	0	0	0	0	0	0	0
Roseburia_	0.018771665	0.0025112	0.004588782	0.002222652	0.004553209	5.90E-05	0.003675453	0.0080921	0	0.002822591
Roseburia_	0.006199105	0.0005748	0.000290018	0.000232579	0.000136606	0.005522916	0.002324176	0.0024883	0.003420823	0
Roseburia_	9.90E-05	0	0	0	0.001407265	0	0	6.21E-05	8.69E-05	0
Roseburia_	0.084184045	2.64E-05	0.000456928	0.000182362	0.00413909	0.000107218	0	0.0063169	0.001013485	0.011872206
Roseburia_	0	0	0	0	0	9.04E-05	0.000125026	0	0	0
Roseburia_	0	0	0	0	0	0	0	0	0	0
Roseburia_	0	0	0	0	0	0	0	0	0	0
Rothia_muc	0.00079488	0	0	7.13E-05	0	0	0	0	0	0
Ruminococ	0	0.0014013	0	0.00750624	6.75E-05	0.004655472	0.009265398	0	0	0
Ruminococ	0	0.017584602	4.90E-05	0.000553087	0	0	0	0.0034609	6.05E-05	0
Ruminococ	0.005323856	0	0.001626	0.010985618	0.00260902	0	0.002983611	0.0052036	0.001181515	0.006486688
Ruminococ	0	0	0	0	0	0	0	8.70E-05	0	0
Ruminococ	0	0	0	0	0	0.007102378	0	0	0	0
Ruminococ	0.002438787	0	0	0	0.00022791	0	0.000302262	7.21E-05	5.93E-05	0.002036198
Ruminococ	0.00276927	0	0	0	0	0	6.05E-05	0.0007894	0	0
Ruminococ	0	0	0	0	0	0	0.000241049	0	0	0
Ruminococ	0	0	0	0	0	0	0	0	0	0
Ruminococ	0	0	0	0	0	0.004712582	0.000159833	0	0	0
Ruminococ	0	0	0	7.49E-05	0	0	0.001181442	0	0	0
Ruminococ	0	0	3.16E-05	0	9.88E-05	0.001316218	0.001640836	0	0	0.008733038
Ruthenibac	0.00209189	0.0012746	0.000432127	0.005698528	0.003653268	0.000535689	0.005641055	0.0004733	0.001765622	0.003536666
Sanguibacte	0	0	0	0	0	0.001918618	0	0	0	0
Scardovia_	2.64E-05	0	0	0	0	0	0	0	0	0.000169742
Sellimonas_	6.31E-06	1.16E-05	0	0	0	0	0	0	0	0.000191447
Slackia_iso	0	0	0	0	0	0	0.014265167	0	0	0

Streptococc	0.000110595	0	0	2.41E-05	0	0	0	0	0	0.000231357
Streptococc	0.00012861	0	0	0	0	0	0	0	0	0
Streptococc	0	0	0	0	0	0	0	0	0	0
Streptococc	0.000353703	0	0	0	0	0	0	0	0	0
Streptococc	0.00015243	0	0	0	0	0	0	0	0	0
Streptococc	0.000265827	0	0	0	0	0	0	0	0	0
Streptococc	0.003126275	0	8.45E-05	0.00011924	1.77E-05	0	0	0	0	0
Streptococc	0.00862308	0	0.000446427	5.60E-06	0.00042532	0	0.00024535	1.28E-05	0	0
Sutterella_p	0	0	0	0	0	0	0	0.0009091	0.00955264	0.000429505
Turicimonas	0.001972488	0.0011988	0.001911918	0.001206708	0.002360409	0	0.000212544	0	0	0
Tyzzerella_	1.21E-05	0	0	0	0	0	0	0	0	0.001071662
Veillonella_	0.001162495	0	3.63E-05	5.60E-05	0	0	0	0	0	0
Veillonella_	0.000974034	0	0	0	5.86E-05	0	0	0	0	0
Veillonella_	0.000603216	0	0	0	1.95E-05	0	0	0	0	0
Veillonella_	0.004082294	0	0	7.60E-05	0.00043442	0	0	0	0	0.000787593
Veillonella_	0.000254218	0	0	0	0	0	0	0	0	0
Victivallis_v	0	0	0.000371323	0	0	6.93E-05	0.005936116	0	0	0.000614951
Weissella_c	0	0	0	0	0	0	0	0	0	0
Weissella_c	0	0	4.13E-05	0	0	0	0	8.59E-05	0	0

Table S8. Relative abundance of Metacyc pathways based on the metagenomic sequencing data

	W12	W13	W16	W25	W26	W27	W28	W29	W30	W31
N10-formyl-	0.010464161	0.010685134	0.00927008	0.008417198	0.010175997	0.009674621	0.006566547	0.006326161	0.010694809	0.007156116
4-hydroxyph	0	2.64E-05	5.04E-05	0.000316752	9.74E-05	0.000254303	0.000695902	0.000703368	3.96E-05	0.000370792
aerobactin b	0	1.27E-05	0	0.000106227	7.97E-05	0	0	0	0	0
superpathw	0	2.49E-05	0	3.94E-05	3.48E-05	0	0.000111788	4.53E-05	0	7.56E-05
homolactic f	0.005132237	0.00452941	0.004533932	0.004251222	0.004139687	0.003273024	0.003956773	0.004456939	0.00436402	0.005090189
glycolysis III	0.00858621	0.006509109	0.006799383	0.006644786	0.006541253	0.005302239	0.005589655	0.006102543	0.00841428	0.006405144
superpathw	0.000344807	0.000827077	0.001998139	0.002251066	0.001836377	0.001705035	0.003202174	0.003198593	0.001316285	0.002836497
superpathw	0	9.86E-05	0.000918769	0.001060394	0.000279207	0.000669988	0.001481926	0.001522077	0.000193246	0.00096656
L-ornithine (	0.006726325	0.006479061	0.005951628	0.001197823	0.00155436	0.00763015	0.002707464	0.00381737	0.007354349	0.003627414
arginine, orn	0	4.44E-05	0.000130045	0.000168729	0.000169827	0	2.07E-05	6.57E-05	0	0.000302192
L-arginine b	0.006179026	0.005343963	0.004928712	0.006919326	0.0092329	0.002784944	0.006535348	0.004575546	0.005825759	0.005960544
L-arginine b	0.006399125	0.004533676	0.004641859	0.006884734	0.008746646	0.002474107	0.006212437	0.004164105	0.003516145	0.005813114
chorismate	0.010112233	0.008505475	0.007824998	0.009388659	0.011397977	0.007009262	0.007304921	0.007802422	0.010076164	0.008391263
superpathw	0.006611285	0.005735559	0.006659257	0.00324008	0.00645498	0.007980629	0.004604125	0.004867956	0.005601664	0.004749915
L-arginine d	8.71E-06	0.000148986	0.000407137	0.000558745	0.000306883	0.000607216	0.00112344	0.001526295	6.94E-05	0.001062294
biotin biosyn	0.003297511	0.004898042	0.004020729	0.003098158	0.002605935	0.006418243	0.004172441	0.004730066	0.005695369	0.003754228
superpathw	0.009449998	0.010148303	0.008070372	0.006661797	0.0091907	0.006404401	0.005496693	0.006754709	0.007917733	0.006556628
Calvin-Bens	0.010067647	0.007117473	0.006586064	0.008575019	0.00738372	0.005875782	0.006570125	0.007057976	0.005144759	0.008106308
catechol de	1.31E-05	3.00E-05	0	8.20E-05	0.000205997	0.000271585	0.000315977	0.00066718	4.29E-05	0.000315025
pyruvate fer	0.000333445	0.000182728	0.000254154	0.00056625	0.000373551	0	0.000764791	0.000194823	0.000440053	0.000302378
L-citrulline b	0.003555627	0.002819998	0.003227553	0.00176738	0.002456751	0.003466835	0.004110322	0.003168763	0.003958352	0.002771107
coenzyme A	0.01159148	0.011655718	0.01012792	0.011944156	0.012901576	0.008434964	0.006966648	0.006552624	0.01091758	0.008808467
coenzyme A	0.010805521	0.011908188	0.009925821	0.008430994	0.010148571	0.008102788	0.006445601	0.006502365	0.010899131	0.008156617
adenosylcol	0.004106342	0.003525985	0.003306228	0.001696528	0.001936863	0.005376283	0.003393455	0.002443804	0.005621944	0.003003044
colanic acid	0.001614505	0.002192626	0.002547061	0.002158633	0.001352098	0.001688521	0.002625575	0.002321858	0.002953296	0.001806528
superpathw	0.00987397	0.008634214	0.007777347	0.007803648	0.009933748	0.007162642	0.006342663	0.00766931	0.009814434	0.007957982
creatinine d	0	0	0	0	0	0	0	0	0	4.29E-05
L-lysine bios	0.000117939	0.000887688	0.002168385	0.002443396	0.001242906	0.002114552	0.002498873	0.003296978	0.000437321	0.003308861
superpathw	0.006887849	0.007169011	0.005985271	0.005925634	0.004439266	0.005899058	0.005334454	0.005011488	0.005747683	0.005767713
dTDP-L-rha	0.01358249	0.013427848	0.011857212	0.010230451	0.01543612	0.013605285	0.006857785	0.00564079	0.014444289	0.007251184
enterobacte	0	8.19E-05	0.000265701	0.000256245	0.00021895	0.000364568	0.000520293	0.001120118	6.53E-05	0.000466284
enterobactir	0	0.000537817	0.00132977	0.002340904	0.000901676	0.000884984	0.002442435	0.002324697	0.000393997	0.001716938
fatty acid &b	0.000438995	0.000544644	0.001426779	0.002016832	0.000628351	0.001647164	0.003523017	0.002455259	0.000769589	0.002119221
fatty acid el	0.003076129	0.004513391	0.003877111	0.004840138	0.004050533	0.006250794	0.005436989	0.004980443	0.00632904	0.004222785
superpathw	0.002235297	0.00334325	0.003257594	0.004096518	0.003020109	0.00583412	0.005295307	0.004469472	0.005069973	0.003721397
mixed acid f	9.68E-05	0.000486876	0.001321243	0.00219943	0.001013914	0.001373151	0.002663714	0.002965407	0.000263837	0.002541855
superpathw	0.000285391	0.000302673	0.000484513	0.000845845	7.60E-05	0.000361488	0.001021279	4.09E-05	8.96E-05	0.001016593
superpathw	0.001089528	0.000806154	0.001042777	0.001326306	0.000757743	0.001787308	0.001540209	0.002006173	0.000603265	0.001529082
fucose degr	0.000673586	0.000702445	0.000606676	0.001439923	0.000528977	0.001403216	0.001066484	0.001538299	0.000290345	0.001159
superpathw	0.001325013	0.001559484	0.002617237	0.001667913	0.002358986	0.001209556	0.002864585	0.001272183	0.000490821	0.001231921
D-galactara	0.000127067	0.000301888	0.000735014	0.001175121	0.000254075	0.000651605	0.002023648	0.001774848	0.000323434	0.000970323
D-galacturo	0.001771104	0.001320068	0.002279983	0.001653903	0.002008547	0.000943054	0.003040169	0.001293601	0.000318746	0.001368849
superpathw	0.001560451	0.000897442	0.00231521	0.001676248	0.000953664	0.00154779	0.002568461	0.000826379	0.000483177	0.002389439
D-glucarate	9.95E-05	0.000214886	0.000567865	0.000747078	0.000196844	0.000483568	0.001193388	0.001375945	0.000184035	0.000811802
superpathw	0.000127067	0.000301888	0.000735014	0.001175121	0.000254075	0.000651605	0.002023648	0.001774848	0.000323434	0.000970323
gluconeoge	0.002694551	0.002404381	0.005097451	0.003599707	0.004043312	0.002912188	0.003766517	0.004178113	0.001517929	0.003774556
glucose and	0.000246761	0.001017133	0.000969649	0.002142973	0.000542483	0.001000181	0.001471714	0.001809709	0.000290952	0.001100939
superpathw	0.002116111	0.001679725	0.002543734	0.001573389	0.002366042	0.00099192	0.002665054	0.001018683	0.000516829	0.001062563
GABA shun	0.000109609	0.000383651	0.002464912	0.001003442	0.00100364	0.001857061	0.002198461	0.002588107	0.001281184	0.001812463
L-ornithine l	0.005301722	0.00411037	0.004191128	0.005171033	0.007982887	0.003007791	0.005433141	0.00371372	0.005844125	0.005467948
glycogen de	0.000482182	0.001818811	0.000558916	0.003615064	0.001036123	0.00071541	0.001260785	0.002310307	0.000559096	0.001670893
glycogen bi	0.001567336	0.001429359	0.002729906	0.00414823	0.00244409	0.00192177	0.003679721	0.003382139	0.002481818	0.003834663
superpathw	6.27E-05	0.000170189	6.77E-05	7.28E-05	0.000138339	5.15E-05	0.000169676	0.000188998	1.79E-05	7.96E-05
superpathw	0.001679772	0.001650925	0.003285366	0.001812541	0.001830444	0.001551336	0.002796408	0.00209513	0.000880611	0.00238921
superpathw	0.000123734	0.00057771	0.001852074	0.001992294	0.001054417	0.001744849	0.002630157	0.0030746	0.000408735	0.002259142
glycolysis I (	0.003394666	0.008767594	0.00454505	0.003565828	0.002948244	0.004751253	0.003876099	0.005693521	0.006595435	0.005099459
glyoxylate c	5.13E-05	0.000256511	0.000769379	0.000938399	0.000486198	0.000810905	0.001387584	0.001703407	0.000139429	0.00113974
superpathw	0.000274383	0.000223964	0	0.000410651	0.000522463	0.000382199	0.001001719	0.001917473	0.000183317	0.001124572
3-phenylpro	0	6.54E-05	0.000410968	0.000613531	0.000300293	0.000392852	0.001529763	0.000816035	0.000155228	0.00073051
heme biosy	0.000136956	0.000144172	0.000485171	0.000651532	0.000232147	0.000450598	0.001153517	0.001228667	0.000186827	0.000806533
heme biosy	0.000158688	0.000154968	0.000540029	0.000510953	0.000250888	0.000450496	0.001037056	0.001080712	0.000209334	0.001083397
superpathw	0.001415868	0.000905529	0.002520745	0.00200039	0.001564006	0.001485942	0.002675495	0.002751245	0.000757866	0.002420543
L-histidine d	0.008164622	0.011520875	0.006745469	0.007369479	0.006172832	0.008252399	0.003913666	0.004394192	0.007967176	0.002365793
L-histidine b	0.007721912	0.007182608	0.007158507	0.004803776	0.009832849	0.00848451	0.00604506	0.005555957	0.011339339	0.006743663
L-methionin	0.000413922	0.000257719	0.00135597	0.001841301	0.000333438	0.001290422	0.00258533	0.001814095	0.000315436	0.002202079
L-methionin	0.005238271	0.003875797	0.003123626	0.004100985	0.002985239	0.002489374	0.003989966	0.004115949	0.003012652	0.003617633
L-isoleucine	0.010779118	0.01059161	0.010528577	0.008454459	0.012431342	0.010359778	0.008887679	0.008171537	0.011911428	0.009159668
superpathw	0	3.45E-05	0.00019037	0.00017003	6.72E-05	0.000341514	0.000428828	0.000526738	2.87E-05	0.000443299
ketoglucona	0	8.40E-05	0.000173218	0.000251935	0.000219063	0.000253571	0.000305763	0.000337798	4.52E-05	0.000335017
lactose and	6.96E-05	0.000101888	0.000423888	0.000574108	0.000125719	3.69E-05	0.00025986	0.000124534	0.000214124	0.000354619
L-leucine de	0	0	0	0	0	0	0	0	0	0
superpathw	0	2.64E-05	0.000201821	0.000208801	0	0.00017295	0.000324628	0	4.29E-05	0.000120616
superpathw	0.000894828	0.000524308	0.002367922	0.003077434	0.000721484	0.00216522	0.00372452	0.003064941	0.000645139	0.003522108
methanoger	0.000111343	0	0.000133558	0.000118453	4.24E-05	2.31E-05	2.11E-05	0	0	0.000109762
superpathw	0.000103879	5.14E-05	0.000374194	0.000408098	0.000124874	0.000637236	0.000924366	0	0.000113991	0.000651409
L-homoserir	0.000794476	0.000502016	0.002343374	0.003073147	0.000649789	0.002274878	0.003885856	0.002932182	0.000607084	0.003516367
NAD salvag	0.0007211	0.000278023	0.001194763	0.000377314	0.000252637	0.001811668	0.001814576	0.00131017	0.001363801	0.000905605
lipid IVA bio	0.003286209	0.006710595	0.003414519	0.001720537	0.001588035	0.003519059	0.002893783	0.003951854	0.004287678	0.002807036
methyleryth	0	0	0.000577998	0.001602195	0	0	0.000536674	0.000336036	0.000110554	0.000634942
pentose pho	0.006250523	0.003925143	0.003300592	0.005668236	0.003285917	0.003180715	0.005149812	0.005940677	0.00286401	0.005634094
O-antigen b	0.002266659	0.003199307	0.003888935	0.003108833	0.004407508	0.001974586	0.002870993	0.004328007	0.003313954	0.005138713
superpathw	0	9.86E-05	0.000918769	0.001060394	0.000279207	0.000669988	0.001481926	0.001522077	0.000193246	0.00096656
superpathw	0	0.00018766	0.001216715	0.000960947	0.000352929	0.000816237	0.001273071	0.001074261	0.000236328	0.000739117
TCA cycle IV	8.49E-05	0.000478729	0.000726715	0.001489792	0.000836863	0.000939114	0.001769528	0.002546695	0.000123581	0.001902097
pyruvate fer	0.001376736	0.002511472	0.000580077	0.000981147	0.001209242	0.000483482	0.000614672	0.001178266	0.000826025	0.001078647
heterolactic	0	3.04E-05	0.000290657	0.000172164	5.17E-05	0.00024828	0.000293404	0.000655876	1.63E-05	0.000368327
Bifidobacter	1.76E-05	0	0.000594893	0.000795865	0.000142267	0	0.00025644	7.41E-05	0.000613702	7.08E-05
acetylene d	0.000250685	0.000219499	0.000835631	0.001045075	0.000353635	0.000858424	0.002174299	0.001612374	0.000424458	0.001188
L-glutamate	5.41E-05	5.24E-05	0.000157187	8.34E-05	4.13E-05	2.30E-05	0.00011143	0.000984578	0.000141154	2.46E-05
L-lysine ferr	5.84E-05	0	0	8.23E-05	0	0	0.000122138	0	0	0
purine nucle	0.000932336	0.000452979	0.000976585	0.000946521	0.000515893	0.000793026	0.001336778	0.001485549	0.00084136	0.000807786
formaldehyd	0.000129315	0.000123673	3.42E-05	0.000359122	0.000592343	0.000579874	0.001147379	0.002345817	6.48E-05	0.00175232
octane oxid	1.70E-05	6.04E-05	3.65E-05	0.000201793	0.000174507	0.000202068	0.000475364	0.001024732	4.10E-05	0.000486795
reductive TC	0	0.000113899	0.000125523	0.000203002	0.000250213	7.99E-05	0.000364706	0.000996814	8.30E-05	6.37E-05
superpathw	5.91E-05	0.000670398	0.001446228	0.001992198	0.000892185	0.001671475	0.002460322	0.002823999	0.000434578	0.002606374
incomplete	0.000394152	0.000661453	0.000313415	0.00043691	0.000561873	0.00041928	0.000679491	0.001095828	0.00080999	0.000934203
superpathw	0.000775217	0.000634159	0.002377489	0.002087558	0.000953921	0.001739856	0.002956906	0.001758543	0.000752868	0.002405459
hexitol ferm	0.000374864	0.000753332	0.001236206	0.002336213	0.001217718	0.001184627	0.002398128	0.002184851	0.000816961	0.001808525

myo-inosito	0	0	0	0	0	0	0.000118284	0.000150697	1.89E-05	5.88E-05
phosphopa	0.009905664	0.010685584	0.009486061	0.006744691	0.008869066	0.008568637	0.005262032	0.006454645	0.009532278	0.006952295
pantothena	0.010136245	0.0108675	0.008820045	0.00675704	0.009011606	0.008168594	0.005454711	0.006396803	0.009910741	0.007257595
pentose ph	0.003963975	0.003964589	0.003293476	0.002440374	0.001850821	0.003648094	0.002995171	0.003186244	0.003173278	0.002427863
peptidoglyc	0.014032606	0.012643122	0.009853294	0.014182837	0.015194785	0.008967382	0.007495848	0.007527621	0.010394262	0.008852892
superpathw	0.005374704	0.00180955	0.00373462	0.003964473	0.003187617	0.003743301	0.004319712	0.003735331	0.002884428	0.004427313
superpathw	6.90E-05	0.000186008	6.35E-05	0.000100038	0.000398327	0.00021514	2.68E-05	0	2.45E-05	0.000240467
superpathw	0.000178091	0.000448935	0.001280899	0.001365907	0.001030859	0.001237315	0.002154979	0.002467655	0.000752934	0.00190159
polyisopren	0.001188546	0.000501954	0.001259922	0.000531737	0.000409926	0.001873032	0.001291731	0.001448536	0.000929899	0.001092231
ppGpp bios	3.25E-05	0.000412841	0.000359281	0.000481314	0.000475416	0.00051637	0.000723991	0.001188968	8.78E-05	0.000660182
protocatech	1.82E-05	5.12E-05	8.00E-06	0.000136421	0.000234756	0.00033456	0.000340803	0.000893762	2.55E-05	0.0004448
superpathw	0.00226196	0.002390409	0.00409802	0.005187605	0.002557668	0.003707044	0.004641284	0.004144702	0.003169933	0.004199287
photosynthe	0	0	8.12E-06	1.04E-05	0	0	0	0	0	4.80E-06
glycolysis IV	0.013363032	0.011996642	0.01180628	0.010973475	0.01402506	0.011470751	0.008240761	0.009240539	0.008009241	0.010349357
CMP-3-deo	0.002463037	0.003260793	0.002836126	0.003049542	0.006065845	0.003293551	0.003760216	0.003649649	0.005580569	0.002448177
superpathw	0	6.59E-06	0	1.46E-05	6.75E-05	1.63E-05	0.000134	0.000464032	0	0.000189151
formaldehy	8.91E-05	0.000131351	0	2.75E-05	0.000438091	1.74E-05	0.000137906	0.000371036	0.000100007	0.000237461
Entner-Dou	3.12E-05	0	0	3.58E-05	0	0	1.71E-05	0	0	0
C4 photosy	8.09E-05	0.000546018	0.00181934	0.002057093	0.000861599	0.001605866	0.0026847	0.002800437	0.000619245	0.002408533
trehalose d	0.000284258	0.001151748	0.000300995	0.002209664	0.000620164	0.000391579	0.000698379	0.001390613	0.000315128	0.000971054
L-lysine bio	0.00145289	0.001507864	0.000598915	0.004482124	0.000680075	0.000952012	0.000619244	0.000997348	0.003164375	0.001204923
L-lysine bio	0.00857645	0.008824678	0.007652011	0.007121323	0.009868832	0.009321795	0.005780387	0.005908514	0.00915392	0.007044352
superpathw	0.008439181	0.008588042	0.007694627	0.005940074	0.008435393	0.007037754	0.005745265	0.005859946	0.007087237	0.00592489
aerobic resp	0	0	8.82E-05	1.19E-05	3.74E-05	0	8.48E-06	0	0	0.000525014
sucrose deg	0	0.000263014	0	0.000334105	0.000585565	0	0.000507933	0.001727691	0.000158719	0.000701606
folate transf	0.012247536	0.012260585	0.010240589	0.011406135	0.011879992	0.009436805	0.006850547	0.006443985	0.011582641	0.007671686
γ-g	0.001165746	0.001522365	0.001856191	0.001780134	0.001045077	0.001572683	0.001978437	0.001982567	0.000492308	0.001845465
pantothena	0.010873586	0.011192421	0.009443502	0.007665757	0.009810458	0.008263348	0.006501951	0.00644296	0.010435702	0.008167816
L-glutamate	0	5.48E-05	1.75E-05	4.05E-05	0.000110549	2.96E-05	0.000135148	0.000495836	0	0.000127561
phytate deg	1.34E-05	5.53E-05	0.00101916	0.000912709	0	0.000314781	0.000792754	2.80E-05	5.92E-05	0.000148643
L-proline bi	0.002773243	0.000900396	0.002603849	0.006980578	0.002916001	0.002920978	0.002723778	0.001736924	0.001466742	0.001105694
urea cycle	0.002657323	0.002475489	0.002768927	0.001464144	0.002075659	0.002502532	0.003567875	0.002752086	0.003413776	0.002393031
superpathw	0	0	0	0.000252933	0.000368439	0	9.31E-06	0	0	0
biotin biosy	0.000103421	5.93E-05	0.000131729	0.00012765	0	0.000103689	0.000164164	0.001613375	0.000955452	0.001273691
4-aminobut	0.00017701	0.000372633	0.002434351	0.001196687	0.000790877	0.001842555	0.002015521	0.002657084	0.001266442	0.001995723
L-histidine	0	0	0	0	0	0	9.20E-06	0.00065187	0	4.46E-05
L-histidine	0.008183727	0.01067627	0.005633933	0.002945011	0.003707647	0.007343925	0.003062417	0.003359733	0.005298636	0.002278322
NAD/NADH	0.000374155	0.00024695	0.001345027	0.001664271	0.000521745	0.001477662	0.001995224	0.002639549	0.000733493	0.001404216
L-glutamate	0	0.000102424	0	0	0	0	0	0	0	1.59E-05
L-lysine bio	0.008050522	0.008961094	0.008239965	0.007343945	0.010529126	0.009259401	0.005876153	0.006252752	0.009334674	0.007318188
pyruvate fer	0.002548934	0.001233427	0.00195072	0.002618765	0.002144815	0.001063702	0.002075505	0.00190492	0.000809903	0.003541973
L-isoleucine	0.008916474	0.009621901	0.007502144	0.006173907	0.008304719	0.005565957	0.00479644	0.006293424	0.007059467	0.005955375
L-isoleucine	0.002590709	0.001640683	0.001360209	0.002015733	0.000680841	0.000877978	0.002311616	0.001855307	0.000871041	0.001536909
superpathw	0	0	0.000428759	0.0005732	0	0	0.000403096	0.00024668	8.53E-05	0.000444956
fatty acid &b	0.000469911	0.000567154	0.001223082	0.001687434	0.000637041	0.00167287	0.003267963	0.00231347	0.000776296	0.002075066
unsaturated	0.000475729	0.000377461	0.000282862	0.000408452	0.000411774	0.000943416	0.001557771	0.001935921	0.000173931	0.001202969
L-arginine b	0.005732861	0.00561414	0.004998012	0.003775679	0.008160935	0.003578632	0.004771782	0.003739815	0.006992951	0.004349054
superpathw	8.21E-06	0.00025038	0.000709114	0.000778733	0.000351432	0.000620465	0.00131603	0.001910441	0.000119809	0.001327082
glutaryl-Co	0.000588384	0.000456737	0.002321588	0.00208208	0.00069469	0.001745742	0.003478355	0.002089495	0.000664639	0.00219539
toluene deg	0	0	0	0	0	0	0	0	0	0.000663892
toluene deg	0	4.47E-05	0	0.000128384	0.000193227	0.000236634	0.00034796	0.000976096	2.26E-05	0.000490652
toluene deg	0	0	0	0	0	0	0	0	0	0.000663892
tetrapyrrole	0.002840665	0.003411926	0.001709163	0.003121559	0.000847847	0.00104659	0.002037503	0.003263269	0.001933416	0.003156473
tetrapyrrole	4.58E-05	6.76E-05	0.001242801	0.000726182	0.000152521	0.000282173	0.000989629	0.001184235	0.000132072	0.001358036
peptidoglyc	0.000318779	0.000289474	0	0.00016283	0	0	0	0	0.00042555	0
superpathw	0.001239944	0.000328319	0.000116474	5.49E-05	0.000364974	3.52E-05	6.49E-05	0.000672334	0.000258728	0.000147842
superpathw	0.000150928	0.001385453	0.002598037	0.002864891	0.001896487	0.001906152	0.003845843	0.003928146	0.001154612	0.003539026
superpathw	0.000912076	0.00057964	0.002622265	0.003026508	0.000743572	0.00257328	0.003910801	0.003183375	0.000696317	0.003650765
petroselina	0.000193342	5.97E-05	0.000318773	0.000382715	0	0.000212254	0.000693949	0.000133739	4.91E-05	0.000107306
sucrose deg	0.000254187	0.000978553	0.000584728	0.002121384	0.000617336	0.00039165	0.000689688	0.001392858	0.000283376	0.000883237
reductive T	0	4.15E-05	0.000113035	0.000153758	0.000184573	6.54E-05	0.000253756	0.000243995	0	5.78E-05
catechol de	0	3.98E-05	0	0.000103745	0.000251831	0.000335901	0.000407244	0.000851538	5.27E-05	0.000396727
aromatic co	0	3.98E-05	0	0.000103745	0.000251831	0.000335901	0.000407244	0.000851538	5.27E-05	0.000396727
superpathw	4.18E-05	9.77E-05	0.000865501	0.000287161	0.000126689	0.000232683	0.000776604	0.00264971	0.000208531	0.001439299
glycolysis II	0.003304336	0.008529871	0.004421183	0.003349419	0.002803622	0.004768529	0.003759207	0.005510893	0.006566943	0.00479469
L-glutamate	0.00094167	0.000404243	0.001400818	0.000614695	0.001506117	0.000412944	0.001656898	0.000250633	0.000477637	0.001308293
superpathw	8.07E-05	0.000360131	0.000741299	0.001352592	0.000502947	0.000971155	0.001844358	0.002244393	0.000141802	0.00168867
2-amino-3-	0	1.83E-05	0	4.50E-05	4.47E-05	2.44E-05	0.000125911	1.84E-05	1.46E-05	0.000109704
mannosylgl	0	3.16E-05	0.000538528	0.000352494	0	0.000273777	0.000340081	1.68E-05	6.47E-05	0.000109543
GDP-mann	0.00290569	0.002609017	0.002483485	0.002060444	0.002773279	0.005091068	0.002940666	0.002033106	0.004542957	0.001552797
CDP-diacyl	0.008874652	0.008141027	0.008163837	0.006920391	0.008684044	0.00851008	0.006110172	0.005697485	0.010237789	0.007126684
nitrate redu	0	0.00016979	0.001292569	0.00111186	0.000516507	0.000905127	0.001641792	0.001167849	0.000236748	0.000781869
acetyl-CoA	0.000714516	0.000375356	0.00135271	0.00092908	0.000167486	0.000431529	0.002100032	0.000556374	0.000119896	0.000715291
succinate f	0.000118439	3.00E-05	0	4.39E-05	0	0	0.000407352	6.98E-06	1.65E-05	5.30E-05
UMP biosy	0.011933598	0.012439852	0.01065299	0.010251849	0.014122648	0.00952147	0.007760999	0.008010496	0.010847627	0.009218004
TCA cycle I	0.000260639	0.000476277	0.000652623	0.001319476	0.001014159	0.000773965	0.001542402	0.002498336	0.000891605	0.001764887
allantoin de	0	0	0	8.88E-05	0	3.32E-05	0.000146485	0	5.80E-06	3.80E-05
urate biosy	0.012075167	0.010147262	0.009854821	0.007844295	0.012249856	0.00962569	0.006954998	0.006897254	0.010502529	0.008671916
allantoin de	0	0	3.27E-05	0.000196607	7.27E-05	0.000151806	0.000238664	1.77E-05	1.94E-05	5.61E-05
Rubisco sh	8.87E-05	0.000491811	0.001501562	0.001310682	0.000384192	0.00094408	0.001680246	0.002188344	0.000141174	0.001655003
2-methylcitr	1.23E-05	1.13E-05	0.000371931	0.00054244	6.89E-05	0.000275178	0.000811596	0.000163884	8.74E-05	0.000325308
1,4-dihydro	3.08E-05	0.00018512	0.000803377	0.000852029	0.000429661	0.000742051	0.001505297	0.001823269	0.000157269	0.001641794
1,4-dihydro	3.08E-05	0.00018512	0.000803377	0.000852029	0.000429661	0.000742051	0.001505297	0.001823269	0.000157269	0.001641794
superpathw	0.000104789	0.000611348	0.002212571	0.002084927	0.001234953	0.002123028	0.002980768	0.003440297	0.000519298	0.003023047
superpathw	0.000102075	0.000407995	0.001459631	0.001847471	0.00089446	0.001308883	0.002151437	0.002870949	0.000357015	0.001951104
superpathw	0.000104751	0.000606337	0.002090969	0.001847471	0.00089446	0.001992884	0.002151437	0.002870949	0.000395152	0.001951104
superpathw	0	0.000249188	0.001459631	0.001847471	0.00089446	0.001308883	0.002151437	0.002870949	0.000357015	0.001951104
ubiquinol-7	0	1.99E-05	0.000214562	0.000187528	9.30E-05	0.000351454	0.000434516	0.000567037	4.76E-05	0.000594193
ubiquinol-9	0	1.99E-05	0.000214562	0.000187528	9.30E-05	0.000351454	0.000434516	0.000567037	4.76E-05	0.000594193
ubiquinol-1	0	1.99E-05	0.000214562	0.000187528	9.30E-05	0.000351454	0.000434516	0.000567037	4.76E-05	0.000594193
superpathw	0	0.000167211	0.001028921	0.001294974	0.000612654	0.000921192	0.001664003	0.002278727	0.000241066	0.001472986
superpathw	7.01E-05	0.000414166	0.001607255	0.001471139	0.000854793	0.001547812	0.002457136	0.002837885	0.000352703	0.002461321
superpathw	7.01E-05	0.000410717	0.001511475	0.001294974	0.000612654	0.001444642	0.001664003	0.002278727	0.000267184	0.001472986
superpathw	3.52E-05	0.000188704	0.000846034	0.000727794	0.000381776	0.000820456	0.001463915	0.001380372	0.000175776	0.001340127
superpathw	0	0.000249188	0.001459631	0.001847471	0.00089446	0.001308883	0.002151437	0.002870949	0.000357015	0.001951104
superpathw	9.19E-05	0.000541488	0.002056164	0.002107489	0.0011697	0.001926073	0.002944002	0.003452349	0.000457982	0.003138321
superpathw	9.19E-05	0.000541488	0.002056164	0.002107489	0.0011697	0.001926073	0.002944002	0.003452349	0.000457982	0.003138321
superpathw	9.19E-05	0.000541488	0.002056164	0.002107489	0.0011697	0.001926073	0.002944002	0.003452349	0.000457982	0.003138321
TCA cycle V	6.51E-05	0.000455646	0.001794546	0.002028699	0.000805399	0.001487989	0.002669332	0.003193904	0.000513784	0.002519323
superpatha	9.37E-05	0.000208378	0.000838072	0.000948113	0.000310828	0.00050775	0.001288604	0.001547294	0.000232085	0.001314656

superpathw	2.41E-05	8.80E-05	0.000493033	0.000551575	0.000176334	0.000320412	0.000792613	0.000975517	0.000139504	0.000812545
glycogen de	0.00062167	0.000647135	0.000354238	8.43E-05	0.000252816	0.001115891	0.000266584	0.000790619	0.000687244	0.000445418
palmitate bi	0.002942141	0.002710114	0.00370896	0.00446286	0	0.005005577	0.005129465	0.003721944	0.002848385	0.003161328
cis-vaccena	0.01084781	0.010001861	0.007315654	0.006405068	0.007098423	0.00869506	0.00559244	0.005748995	0.010507266	0.006168116
stearate bio	0.002458087	0.003752675	0.003364405	0.004282533	0.003454797	0.006041492	0.005171292	0.00435338	0.005388622	0.00354436
palmitate bi	0	0	0	0.000513115	0	0	0.001005696	0.000609289	0	0.000247355
superpathw	0	0	1.04E-05	0.000131597	0	8.51E-05	0.000219574	7.88E-06	2.19E-05	6.64E-05
superpathw	0.001719158	0.000841057	0.002432785	0.002879025	0	0.002358693	0.003893679	0.001583008	0.000738988	0.001302508
5-aminoimid	0.011005186	0.011528863	0.008600559	0.007600957	0.010313736	0.009441933	0.007462999	0.00671733	0.010658261	0.007430721
5-aminoimid	0.011003299	0.012746043	0.009286168	0.007519137	0.010792207	0.009825242	0.007894564	0.006753377	0.010445602	0.007764584
inosine-5'-p	0.010258851	0.012045423	0.008424374	0.007116104	0.00784662	0.009427025	0.006529166	0.005871552	0.010643708	0.006464326
inosine-5'-p	0.009648337	0.011732881	0.007977938	0.006669744	0.007192681	0.009281622	0.006231172	0.005582706	0.010522897	0.006053856
superpathw	0.006008292	0.005580967	0.005242298	0.005197043	0.002883815	0.008362132	0.004356811	0.004439826	0.006854235	0.005250991
superpathw	0.007809039	0.008112579	0.006710181	0.007234456	0.006983817	0.008326374	0.006083483	0.005091679	0.007106487	0.005931378
CMP-pseud	0	1.10E-05	0	6.30E-05	0.000164332	0	0	0	0.00168212	0
6-hydroxym	0.005224244	0.007420358	0.004127537	0.00213609	0.00655472	0.006239223	0.003397968	0.004905685	0.00682641	0.004214959
S-adenosyl-	0.008979421	0.008024952	0.008127469	0.007538371	0.008613621	0.007563837	0.004873822	0.004050097	0.005235501	0.00580938
chorismate	0.011965075	0.010067028	0.00900506	0.009727607	0.012723838	0.008823348	0.007162166	0.007973116	0.011132148	0.008706899
flavin biosyn	0.006346664	0.004588282	0.00561035	0.003943504	0.004866208	0.006307416	0.004093841	0.003945127	0.003763907	0.004686057
superpathw	0	2.43E-05	0	9.17E-05	0.000218369	0.000265922	0.000315229	0.000744468	2.18E-05	0.000312521
4-methylcat	0	1.41E-05	0	9.62E-05	0.000229062	0.000385947	0.000295077	0.000754634	1.60E-05	0.00030382
2-aminophe	0	1.83E-05	0	4.50E-05	4.47E-05	2.44E-05	0.000125911	1.84E-05	1.46E-05	0.000109704
4-chlorober	0	4.59E-06	0	0.000110669	0	6.19E-05	0.000118371	2.92E-06	0	3.36E-05
sucrose deg	0.001336472	0.000673284	0.001329969	0.00096972	0.001629039	0.002042405	0.001974963	0.002728588	0.001769114	0.002479258
starch biosy	0.000152712	0	0.000169826	0.001013389	0	0	0.000133001	0.000144115	8.95E-05	0.000210313
superpathw	0.00055176	0.000318207	0	0.000163147	0.000134862	6.24E-05	0.000773217	5.98E-05	5.84E-05	0.000291636
isoprene bio	0	0	0.000237633	0.000750157	0	0	0.000221495	0.000132927	4.29E-05	0.000260214
superpathw	0.011003299	0.012746043	0.009286168	0.007519137	0.010792207	0.009825242	0.007894564	0.006753377	0.010445602	0.007764584
palmitoleate	0.002496554	0.003822538	0.003373625	0.004496404	0.003567574	0.005799359	0.005179899	0.004687983	0.005591209	0.003792397
superpathw	0.0005691	0.000196325	0.000899574	0.001078107	0	0.000726837	0.001762371	0.000575644	0.000180283	0.000466095
superpathw	0.000137523	0.000383582	0.001375122	0.0015148	0	0.00132494	0.002403733	0.001038	0.000355511	0.000848485
putrescine b	0.002424974	0.003319083	0.003094095	0.002703111	0.005002727	0.003004607	0.004185942	0.003613813	0.005064382	0.00277595
galactose d	0.002761689	0.000763603	0.003102264	0.002459494	0.003171619	0.001319782	0.003205414	0.001900087	0.001555398	0.003802713
L-phenylala	0.000184973	4.83E-05	1.86E-05	0.000236505	0.000296599	0.000428854	0.000697942	0.001042338	2.34E-05	0.000592809
purine nucle	0.001377802	0.0004475	0.001591344	0.001634069	0.00051164	0.001069965	0.002955975	0.000267109	0.000559448	0.001452994
peptidoglyca	0.013375436	0.012428206	0.010073798	0.013818447	0.015194785	0.009281207	0.007748183	0.007873363	0.010772552	0.008922169
UDP-N-ace	0.013559453	0.011934775	0.009975558	0.013964602	0.015812516	0.008917233	0.007495432	0.007682483	0.010445376	0.008869865
UDP-N-ace	0.013673823	0.012422998	0.009840625	0.014357708	0.015407631	0.009039618	0.007579879	0.007654714	0.010532421	0.008858959
4-hydroxybe	0	0	7.81E-05	0	0	0	0	1.74E-05	0	1.14E-05
peptidoglyca	0.001239145	0.000731894	0.000779167	0.000142786	0.000146954	0.000179871	0.000787775	0.000567041	0.000953872	0.001510233
peptidoglyca	0.001315577	0.001240156	0.000836765	0.001800552	0.000158192	0.000183467	0.002267624	0.00062467	0.001009571	0.001963838
GDP-D-glyc	2.26E-05	5.20E-05	0	1.04E-05	5.19E-05	2.53E-05	2.51E-05	5.77E-06	4.62E-05	8.96E-06
4-deoxy-L-t	0.001823757	0.001295209	0.002116926	0.001352404	0.002008907	0.000696948	0.002181905	0.001184481	0.000259668	0.001157762
8-amino-7-c	0.002822193	0.004278585	0.003600642	0.003142213	0.00278659	0.006104432	0.004598029	0.004508171	0.005787443	0.003606906
stachyose c	0.002231055	0.000811067	0.002462852	0.001847247	0.002454382	0.001252205	0.002143115	0.001785816	0.001016779	0.003599675
mannitol cyc	0.000204718	0.000117621	0.000898353	0.000550516	0.000447109	0.00062381	0.000957604	0.001115046	0.000636724	0.000570141
pyrimidine c	0.001899298	0.000963726	0.002425794	0.002645202	0.00210908	0.001069941	0.002836383	0.001833372	0.002698694	0.002389144
L-glutamine	0.000129952	0.000382763	0.000596778	0.000991542	0.001110534	0.000379704	0.001414445	0.001898574	0.000408779	0.001604001
norspermidi	0	6.68E-05	0	0.000199739	0.000464608	0.000158367	0.000160886	0.00027607	3.46E-05	0.000223962
chondroitin	0.000117992	0.000475498	0.00014522	0	0.000104788	3.88E-05	6.48E-05	3.13E-05	0.00011046	4.05E-05
pyruvate fer	0.000281234	0.000288169	0.000278545	0.000251243	7.16E-05	9.65E-05	0.00063383	0.000201628	4.90E-05	0.000187828
superpathw	0.000414367	0.00022805	0.000321236	0.000694371	0.000459707	0	0.000923172	0.000248087	0.00050659	0.000382224
superpathw	0.000499482	0.000494482	0.000704997	0.00111106	0.000801198	0.000458174	0.001275459	0.000683194	4.06E-05	0.00028628
guanosine r	0.000664769	0.000474335	0.000812321	0.001201373	0.000716671	0.000572729	0.001709058	0.000778062	0.000297667	0.000705157
guanosine r	0.001285067	0.000582736	0.003364269	0.002105091	0.000803789	0.001245889	0.002867592	0.002482359	0.000942143	0.001500491
adenine and	0.010258429	0.008506679	0.008939741	0.010954715	0.013780847	0.009155487	0.007001037	0.006571114	0.00956364	0.008564502
superpathw	0.000191637	0.000203374	0.000328685	0.000580636	5.08E-05	0.0002439	0.000715544	2.73E-05	5.99E-05	0.000711018
superpathw	0	0.000629077	0.002173012	0.002070557	0.001568547	0.00186049	0.002695963	0.003185477	0.000569983	0.002407898
superpathw	0	0.000550754	0.001788895	0.001455129	0.001670686	0.002996357	0.002794551	0.004053731	0.000775775	0.003163207
superpathw	0	0.000640295	0.001171581	0.001161668	0.001555572	0.001216344	0.00233814	0.002853713	0.000546303	0.001865175
cinnamate a	0	6.54E-05	0.000410968	0.000613531	0.000300293	0.000392852	0.001529763	0.000816035	0.000155228	0.00073051
queuosine b	0.0117483	0.009939128	0.009468122	0.010163354	0.010214292	0.009273836	0.00565526	0.006725532	0.009455782	0.00682355
preQ0 biosy	0.00815816	0.008307239	0.006356954	0.003928424	0.004295263	0.007619143	0.003336175	0.005627429	0.006177713	0.00687799
ubiquinol-8	0	1.99E-05	0.000214562	0.000187528	9.30E-05	0.000351454	0.000434516	0.000567037	4.76E-05	0.000594193
starch degra	0.000326152	0.001354417	0.000361108	0.001440363	0.000636	0.000478665	0.000749758	0.001199509	0.000374336	0.000946591
starch degra	0.014786343	0.010571445	0.009245317	0.009237057	0.013280391	0.00761418	0.008949335	0.005541684	0.011798649	0.006833171
CMP-legion	2.04E-05	0.000105162	0	0.000686138	7.43E-05	0	0	2.12E-05	6.09E-05	1.78E-05
hydrogen pr	0	0	1.25E-05	1.95E-05	0	0	0	0	0	8.16E-06
phosphatidy	5.00E-06	0.000347818	0.000369391	0.000694713	0.000341024	0.000662032	0.001383334	0.001900841	0.000139856	0.000838482
molybdenur	0	7.43E-05	0	0.000299432	0	0.000138569	0.000363364	0	5.97E-05	0.000113452
fatty acid be	1.28E-05	3.03E-05	8.53E-05	0.00010709	0.000109038	0.000276035	0.000360211	0.00051221	4.09E-05	0.000222613
all-trans-far	0.000739592	0.000322146	0.000948758	0.000323878	0.000276426	0.001207945	0.001049439	0.000897733	0.000629513	0.000897967
isopropanol	0	0	0	4.09E-05	1.31E-05	7.40E-06	0.000187854	0	0	1.67E-05
thiazole bio	0.000214064	0.000496475	0.001783687	0.002553977	0.000596	0.001275302	0.002554448	0.002376061	0.000354982	0.001722829
thiazole bio	0.002910495	0.006845003	0.003244277	0.003148868	0.0025706	0.004346578	0.003028566	0.00371303	0.00595904	0.003167077
superpathw	0.000978475	0.002076134	0.004504305	0.003934807	0.002258553	0.003473822	0.004062035	0.004516607	0.001519452	0.004303914
thiamin salv	0.00657842	0.005825218	0.005295094	0.00404571	0.00656936	0.004138587	0.004498224	0.005386338	0.006917593	0.005739565
superpathw	0.002143299	0.001705245	0.002934275	0.002685955	0.002236063	0.002268499	0.002834899	0.0031247	0.001208043	0.002637603
seleno-amir	0.00519749	0.002273858	0.003744639	0.002586349	0.002013935	0.008539523	0.004659041	0.003736512	0.00249957	0.004156039
dTDP-3-ace	0	0	0	1.18E-05	0	0	0	0	0	0
erredoxin ox	0.000206438	0.000674219	0.000751947	0.001640854	0.001251115	0.001018081	0.001872979	0.002732189	0.000393844	0.002079171
glycerol deg	0.000275967	0.000257117	0	0.000518581	0.000193266	0.000250757	0.001235485	0.000539636	0.000101323	0.000493963
L-1,2-propa	0.000399679	0.000304672	5.62E-05	0.000763495	0.001061007	0.000925489	0.002058429	0.00354406	0.000338561	0.002107972
phosphatida	0	0	0	0.000113402	0	0	1.22E-05	0	2.30E-05	9.00E-06
4-coumarat	0.000140682	3.16E-05	0.000314776	0.000306997	4.74E-05	0.000189335	0.000349615	1.46E-05	0	0.000119749
fatty acid sa	0	0.000114457	0.000287941	0.000207177	6.67E-05	0.000416496	0.000557024	0.000533005	3.25E-05	0.000144871
pyruvate fer	0.010779118	0.009605298	0.010528577	0.008454459	0.012431342	0.010359778	0.008887679	0.008171537	0.011911428	0.009159668
C4 photosy	0.000137712	0.000158915	0.000480419	0.000707026	0.000706776	0.000772304	0.000960983	0.001378758	0.00027639	0.001029553
C4 photosy	9.66E-05	0.00062654	0.002005047	0.002280053	0.000886502	0.001950031	0.002922088	0.003226005	0.000718903	0.002562035
L-ascorbate	0	4.77E-05	0	8.21E-05	0	0.000276308	0.000391198	0.000622443	4.29E-05	0.000279457
pyrimidine c	0.005098179	0.004348583	0.0039302	0.004649491	0.002144899	0.003695028	0.003742103	0.004008226	0.005804017	0.004398542
pyrimidine c	0.005960367	0.004983933	0.004109825	0.005431863	0.003294193	0.003370579	0.003928278	0.003942915	0.003837349	0.004293468
superpathw	0.000429473	0.000214798	0.000330073	0.000367812	5.12E-05	0.000478442	0.001297281	0.000317639	6.70E-05	0.000602964
pyrimidine c	0.003689339	0.003101227	0.002587794	0.003323188	0.001422501	0.002055257	0.002694874	0.002922147	0.004179305	0.003230126
pyrimidine c	0.000529627	0.000199612	0.000569885	0.000789366	0.000344369	0.000440955	0.000908545	0.002439445	0.001379024	0.001114724
pyrimidine c	0.009428738	0.008115497	0.008574275	0.010271361	0.010749979	0.00909587	0.005842845	0.005502968	0.008084488	0.006048351
superpathw	0	0.000274561	0	0.000318458	0.000291115	0	2.10E-05	0	0	0
pyridoxal 5'-	0	6.53E-05	0.00051074	0.000454563	0	0.000237597	0.000547183	4.64E-05	6.19E-05	0.000232648
superpathw	0.004311194	0.003984175	0.003494445	0.00440324	0.002074793	0.002508307	0.003347615	0.00376642	0.005113812	0.004149004
superpathw	2.98E-05	8.97E-06	0.000219558	0.000118018	5.52E-05	3.76E-05	0.000114421	5.69E-05	0	6.52E-05

pyrimidine d	0.000427224	0.0001058	0.000170874	0.000116948	0.000449174	0.000589395	0.000950161	0.001157683	0.000671936	0.001103707
superpathw	0.000740944	0.000317903	0.000304364	0.000537684	0.000784417	0.000995092	0.00171652	0.00185503	0.001157247	0.001803811
adenosine r	0.01111021	0.011406884	0.009405695	0.01214325	0.014785542	0.009672371	0.007991775	0.007554769	0.010724777	0.008896766
adenosine d	0.00646132	0.006529101	0.005316962	0.005246497	0.004231842	0.007936748	0.004700713	0.004029004	0.005451076	0.00466057
guanosine r	0.012048444	0.011428055	0.010272542	0.00963082	0.013379916	0.009583369	0.006745118	0.006817995	0.011854182	0.008452057
guanosine d	0.00646132	0.006529101	0.005316962	0.005246497	0.004231842	0.007936748	0.004700713	0.004029004	0.005451076	0.00466057
superpathw	0.006185418	0.005642003	0.005469383	0.005447068	0.002797561	0.008814473	0.004491145	0.004780586	0.00759248	0.005620113
superpathw	0.008554759	0.008927951	0.007454206	0.008308156	0.008336784	0.008768165	0.006818652	0.005696761	0.007957511	0.006559695
inosine-5'-p	0.003875551	0.007437088	0.003738686	0.004806619	0.006371651	0.00300644	0.005066502	0.005270494	0.006045543	0.004911652
myo-, chiro-	0.00296515	0.006037327	0.00237408	0.001014361	0.004566377	0.003623301	0.002225344	0.003813599	0.004634034	0.003820377
D-fructuron	0.00184067	0.001286116	0.002190573	0.001379358	0.00198974	0.00072472	0.002456728	0.000841121	0.000353213	0.000983023
superpathw	0	3.53E-05	0	0	6.81E-05	0.00038809	0.000207224	0.000209172	2.31E-05	0.000166852
superpathw	0.007230276	0.007579637	0.007347943	0.00607183	0.009015424	0.007356237	0.005554304	0.005279948	0.007442384	0.006025137
TCA cycle V	3.16E-05	0.000226094	0.00051582	0.00111691	0.000595701	0.000692515	0.001422715	0.002187661	8.25E-05	0.001354666
NAD/NADP	0	0.000142736	0	0	6.72E-05	0.000648854	0.000182453	0.000175321	2.01E-05	0.000149983
NAD/NADP	0	9.61E-05	0.000227602	0.000332053	0.000141312	0.000448799	0.000660998	0.000939527	8.32E-05	0.000488434
aerobic resp	0	0	7.38E-05	1.16E-05	3.15E-05	0	8.41E-06	0	0	0.000268641
4-amino-2-r	0.004317541	0.004654641	0.00447942	0.001900539	0.003057126	0.003512926	0.002474292	0.003544852	0.005839976	0.003384435
fatty acid &b	0.000333502	0.000263438	0	5.11E-05	0	0	0.001143307	1.62E-05	0	0.000123934
xylose degr	0	0	0	3.90E-05	0	0.0001135	0.000128334	0.000190635	0	3.21E-05
dTDP-D-&b	0.003436788	0.002291729	0.001316479	0	1.47E-05	7.19E-05	0	0.000482784	0	0.000229076
dTDP-N-ace	0.000858905	0.000141069	0.000517346	0.000561596	0.000488684	0.00076309	0.000999206	0.001995296	0.004980427	0.001705122
dTDP-N-ace	0.000198291	0	0	6.22E-05	0	3.55E-05	0	0	0	0
superpathw	0.002416576	0.001886468	0.002336361	0.001678755	0.001164811	0.002500992	0.002553938	0.001833854	0.003590228	0.001382569
superpathw	0.000303778	0.001250594	0.00035665	0.001750791	0.000672823	0.0004217	0.000790408	0.001649807	0.00036185	0.001121134
superpathw	0.001424177	0.002041585	0.001972455	0.001032701	0.000233607	0.001269622	0.0006686	0.002140616	0.000260362	0
superpathw	0	0.000248226	0	0.000314201	0.000551334	0	0.000477838	0.001651229	0.000148881	0.000664037
thiamin form	0.005732563	0.004471872	0.004445664	0.003977341	0.005391515	0.003109117	0.004083633	0.0048009	0.005534952	0.005420881
1,4-dihydro	0.000361709	0.000370975	4.91E-05	0.000400125	0.000621453	0.000103223	0	4.81E-05	0.000857863	0.000215386
1,4-dihydro	0	0	0	0.000123007	0	0	0	0	0	0
anaerobic e	0.004261735	0.00322754	0.000533068	0.004211255	0.004906982	0.000513312	0.001667405	0.001024418	0.000330938	0.000573592
1,3-propane	0	0.000111521	0	0.000586935	0	0.000330753	0.00104287	0	8.16E-05	0.000299041
octanoyl-[ac	0.002089111	0.003260953	0.003057642	0.004106898	0.002936859	0.005456331	0.005549247	0.004644468	0.004857269	0.003615164
superpathw	0	0	0	0	0	0	0	0	0	0
taxadiene b	0	0	0.000254183	0.000470848	0	0	0.000238267	0.000142952	4.75E-05	0.000270022
L-arginine b	0.006192606	0.005374681	0.004962086	0.006910498	0.009287842	0.002797769	0.006562328	0.004622766	0.005829477	0.005980844
phospholipi	0	0	0.000175201	0.000289135	0	0.000149622	0.000417956	1.71E-05	4.49E-05	7.61E-05
sulfoglycoly	0	1.02E-05	0.000158623	0.000269682	1.04E-05	8.71E-05	0.000376912	0.000263875	2.31E-05	6.59E-05
mannan deg	0.001683127	0.002384528	0.001675468	0.001391952	0.00214925	0.002014015	0.001666222	0.00159489	0.003329074	0.001092959
6-hydroxym	0.005234831	0.007021837	0.004132301	0.002183521	0.006518867	0.006237437	0.003326483	0.004715123	0.006957723	0.004024343
methyleryth	0	0	0.000435884	0.000970217	0	0	0.000461896	0.000306372	9.29E-05	0.000469995
methanol ox	0	0	5.41E-05	4.08E-05	7.63E-06	4.65E-05	0.000159243	9.80E-05	7.22E-06	7.65E-05
gondoate bi	0.009887465	0.00940802	0.006589632	0.006057859	0.00647682	0.008052813	0.005793918	0.005388351	0.010012851	0.005695505
oleate biosy	0.002803199	0.004204459	0.003666274	0.004673267	0.003796112	0.006041736	0.005308731	0.004879083	0.005945274	0.004066495
superpathw	8.11E-05	0.000198348	0.000833795	0.000994724	0.000875456	0.00088361	0.001762574	0.001729606	0.000186688	0.001883676
superpathw	0.007193058	0.007147292	0.00624454	0.006196856	0.004100069	0.006445996	0.005399882	0.005188101	0.008021486	0.006062155
superpathw	0.001614315	0.000172878	0.000509933	0.000788317	0.000948357	0.000829263	0.001014285	0.001533404	0.000312348	0.001513842
ADP-L-glyce	0.001743836	0.002633307	0.001004677	0.001389873	0.000526651	0.000825432	0.000887922	0.002296329	0.001387416	0.001285953
anhydromur	0.002573463	0.003522283	0.00353633	0.003499475	0.003355207	0.002240722	0.003304298	0.004490076	0.003614652	0.004752413
3-phenylpro	0	7.97E-05	0.000505602	0.000681575	0.000228475	0.000495561	0.001564052	0.000888798	0.00020237	0.00069684
purine ribon	0.009383446	0.001440801	0.003721512	0.00518276	0.006534985	0.001855694	0.004641983	0.00355806	0.001569433	0.004836642
superpathw	0.000381368	0.000307771	0.001219954	0.001582369	0.000554107	0.00130224	0.003094064	0.002053719	0.000593326	0.001906834
superpathw	0.000317292	0.000501642	0.000885684	0.001294552	0.000653852	0.000981492	0.00248834	0.001754466	0.000340915	0.001997991
CDP-diacylg	0.008874652	0.008141027	0.008163837	0.006920391	0.008684044	0.00851008	0.006110172	0.005697485	0.010237789	0.007126684
polymyxin re	4.76E-06	5.20E-05	0.000175844	0.000231862	0.000146796	0.000525044	0.000516851	0.00070338	8.75E-05	0.000368869
superpathw	6.51E-05	0.000123031	0.000411285	0.000456947	0.000186635	0.000342342	0.000790872	0.000818502	0.000126122	0.000744407
tRNA proce	0.000741975	0.000696471	0.00135776	0.002666479	0.001106921	0.001110313	0.002621259	0.002979581	0.000974852	0.003234531
methylphos	7.82E-06	1.09E-05	0.000129807	0.000170335	0	0	0.000344845	2.77E-05	3.20E-05	7.86E-05
peptidoglyca	0.00860358	0.008042751	0.009394788	0.005253563	0.007767697	0.009113932	0.00803618	0.008747382	0.006911448	0.008624405
superpathw	0.006258837	0.006711129	0.005814596	0.005903927	0.003751322	0.003003721	0.004463383	0.005111627	0.007528453	0.005768459
superpathw	0.00642281	0.005281235	0.004380976	0.005964561	0.003452192	0.003886122	0.004216397	0.004317585	0.006067197	0.004594838
phenylaceta	0	0	1.68E-05	0.000125931	0	7.43E-05	0.000198902	6.46E-06	2.00E-05	5.65E-05
allantoin de	0	0	0	0.000150739	0	9.52E-05	0.000236457	9.32E-06	1.97E-05	5.35E-05
2-methylcitr	8.83E-06	0	0.000372132	0.000584838	5.14E-05	0.000272581	0.000843728	0.000126098	6.88E-05	0.000325652
aspartate su	6.28E-05	0.000672853	0.001483546	0.002035061	0.000923814	0.00159662	0.002485893	0.002879716	0.000449922	0.002579151
superpathw	0.004481839	0.004751684	0.005257632	0.001786977	0.001763925	0.004786041	0.002033949	0.003557712	0.006264651	0.002405725
(5Z)-dodec-	0.002506661	0.003852835	0.003418417	0.004467885	0.00350276	0.005783815	0.005146846	0.004750185	0.005500566	0.003875259
superpathw	0	0.000155044	0.000618512	0.00066521	0.000338307	0.000518308	0.001111921	0.001902634	0.000248865	0.000962987
L-ascorbate	3.14E-05	0.000165584	0	6.23E-05	0.000168435	0.000282819	0.000484991	0.00132526	6.07E-05	0.000702598
stearate bio	0	8.62E-05	0.000429612	0.000391545	0.000198953	0.000308491	0.000829122	0.000605182	0.00012009	0.000271382
nitrate redu	1.05E-05	0	3.10E-05	0	2.66E-05	0	2.88E-05	0	3.54E-05	5.30E-05
phosphatidy	0.003703507	0.001141199	0.002220704	0.002309467	0.002227895	0.002400199	0.003054928	0.002902831	0.001874777	0.003697016
phosphatidy	0.003703507	0.001141199	0.002220704	0.002309467	0.002227895	0.002400199	0.003054928	0.002902831	0.001874777	0.003697016
superpathw	0.000303959	0.000252553	0.000783485	0.001092455	0.000428131	0.000601433	0.002092292	0.001583222	0.00047346	0.001241833
biphenyl de	0	0	0	0	0	0	2.00E-05	5.87E-05	0	5.21E-05
ketogenesis	0	0	0	0	0	0	0.000197487	0	0	3.66E-05
fatty acid &a	0	7.23E-05	1.69E-05	4.67E-05	0.000159137	0	0	1.57E-05	5.01E-05	0
phytol degra	5.52E-05	7.59E-05	3.94E-05	0.000301469	0.000233789	0.000391629	0.000695084	0.001316827	5.03E-05	0.000671259
fatty acid &b	0.000412476	0.00037847	0	9.97E-05	0	0	0.001728307	3.22E-05	0	0.000235075
TCA cycle II	9.68E-05	6.72E-05	0.000571191	0.000132926	8.26E-05	0.000154885	0.00053511	0.001949759	0.000186812	0.000986807
gluconeoge	0.002021973	0.00156448	0.001207401	0.002192821	0.002474076	0.000909882	0.002242763	0.001616747	0.000611935	0.000931801
glycolysis V	0.001928093	0.001675646	0.004779648	0.003755772	0.003197721	0.00228795	0.003639407	0.004242227	0.001030678	0.003618258
superpathw	0.001011633	0.000498071	0.001649089	0.001663791	0.001178231	0.001520594	0.002245922	0.003152531	0.001043858	0.003721958
D-galactose	0.002671621	0.000763603	0.003004086	0.003060463	0.002791267	0.001319782	0.003594784	0.002142162	0.001555398	0.004067982
mycolate bi	0.002864723	0.004295297	0.003738642	0.004725874	0.003872611	0.005978916	0.005619698	0.005007275	0.006094595	0.004098303
NAD salvag	0.000464964	0.00040752	0.001659821	0.001310554	0.000649432	0.001146407	0.001990894	0.002007898	0.000200116	0.001689062
NAD biosyn	0.007648777	0.008420432	0.006688942	0.005817279	0.008281976	0.008109209	0.004486682	0.004708355	0.008344404	0.00522075
pyridoxal 5'-	0.005921529	0.007063966	0.004991019	0.001481081	0.001544407	0.00485818	0.001821377	0.003210772	0.007616368	0.002172106
TCA cycle V	3.07E-05	0.000173199	0.000113751	0.00028458	0.000414609	0.000393214	0.000568973	0.001072405	7.69E-05	0.00076481
L-rhamnose	0.002007722	0.002388471	0.002304346	0.001900514	0.002884682	0.002641241	0.002292601	0.002716474	0.006211029	0.002437695
flavin biosyn	0.008591249	0.006379917	0.007223929	0.005539562	0.008593132	0.007969176	0.004468841	0.005263444	0.008215452	0.006723705
adenosine r	0.001089734	0.000240707	0.002963144	0.001827912	0.0002673	0.000603758	0.002544665	0.000125523	0.000291107	0.00086321
superpathw	0.006872508	0.007337097	0.005568018	0.004815838	0.006146444	0.007195805	0.005176547	0.005161837	0.008003849	0.004541958
sulfate redu	5.56E-05	0.000594379	0.001820554	0.001649863	0.000913574	0.002153447	0.003134437	0.003699775	0.00050069	0.003574576
superpathw	0.000123426	0.001196284	0.002801436	0.002550899	0.001715925	0.002803528	0.003924663	0.003683271	0.001018687	0.004101052
superpathw	6.36E-05	0.000324581	0.001165738	0.001526869	0.000652592	0.001246473	0.002072197	0.002324555	0.000232851	0.001578221
TCA cycle I	0.00026649	0.000881161	0.002241065	0.00329699	0.001621083	0.00248159	0.003549991	0.003335623	0.00059522	0.002482842
teichoic aci	0.000389731	0.000329386	0.000543295	0.000406938	0.000256427	0.001553316	0.00066923	0.00027624	0.000730765	0.000356571
superpathw	0.006393254	0.009255568	0.005829899	0.004854197	0.00540559	0.005511974	0.004272872	0.005232956	0.007770884	0.00557388
superpathw	0.006476221	0.006148285	0.006710834	0.001816541	0.002785963	0.006833634	0.00320676	0.003678241	0.005359053	0.00464809

superpathw	0	3.97E-05	1.92E-05	0.000414483	0.000372398	0.000571522	0.000941538	0.001282789	0.000105642	0.000693591
superpathw	0.007775721	0.007818989	0.007414228	0.005399941	0.007856933	0.009234526	0.005490457	0.005248271	0.006669204	0.005370541
tRNA charg	0.011700816	0.010981687	0.009446354	0.011354333	0.01309119	0.008573906	0.006766843	0.006822741	0.009683386	0.007940531
L-tryptopha	0.003147569	0.002242247	0.003806502	0.002470948	0.001880504	0.004159475	0.002711641	0.004860485	0.003006344	0.005055894
L-tyrosine d	0	0	0	0	0	0	9.63E-06	0	0	3.69E-05
superpathw	0	2.31E-05	0.000231577	0.000210523	0.000108157	0.000390958	0.000484032	0.000635781	5.50E-05	0.000646157
UDP-N-ace	0.001281002	0.001986944	0.002556537	0.002874931	0.002992674	0.00110863	0.002486064	0.004165286	0.002439642	0.004393708
superpathw	0	0	0	8.88E-05	0	3.32E-05	0.000146485	0	5.80E-06	3.80E-05
ureide biosy	0	0	0	6.18E-05	0.000160059	0	8.88E-05	0.000157529	3.06E-05	0.000138009
L-valine bio	0.010779118	0.01059161	0.010528577	0.008454459	0.012431342	0.010359778	0.008887679	0.008171537	0.011911428	0.009159668
	W32	W33	W34	W3	W40	W41	W47	W49	W50	W53
N10-formyl-	0.011911715	0.006898	0.007339902	0.011193041	0.01048922	0.010227427	0.007328439	0.010132182	0.008579039	0.010899877
4-hydroxyp	0	0.000693172	0.000529346	0	0.00012338	6.57E-05	0	0.000146945	0.000405192	0
aerobactin	0	0	2.39E-05	0	0	0	0	4.06E-05	0	0
superpathw	0	8.58E-05	0	0	0	2.13E-05	0	1.75E-05	5.69E-05	0
homolactic	0.005762871	0.004812039	0.003834069	0.003033475	0.004366253	0.002928454	0.003124023	0.003288031	0.002761567	0.005426951
glycolysis III	0.008717142	0.006061613	0.005821561	0.007435012	0.007373831	0.005123375	0.004137898	0.005948845	0.004775313	0.007252993
superpathw	0.001161336	0.002874054	0.002512061	0.000127509	0.001407773	0.001539761	0.001548449	0.003315542	0.001295607	0.000176981
superpathw	5.51E-05	0.001338611	0.000882819	0	0.000235042	0.000463688	0	0.000363259	0.000698305	0
L-ornithine	0.005838811	0.003804366	0.005803732	0.00458183	0.006638637	0.008730745	0.002650098	0.002439047	0.008463282	0.01018583
arginine, or	8.89E-05	8.16E-05	0.00014449	0.000152643	9.27E-05	3.92E-05	0	0.000194478	0	2.95E-05
L-arginine b	0.00735729	0.005127197	0.004767145	0.003639592	0.005937748	0.002611335	0.008217663	0.006730642	0.002258933	0.004145767
L-arginine b	0.008015601	0.005173931	0.00460342	0.003422123	0.004473188	0.002626851	0.007945774	0.005702226	0.002017447	0.003751762
chorismate	0.009078024	0.007915024	0.007733058	0.006201125	0.008773531	0.007818848	0.007593616	0.009183612	0.006343066	0.009980132
superpathw	0.007054375	0.004994218	0.006221559	0.01109733	0.006501919	0.009029464	0.00939822	0.004214095	0.007934491	0.00858643
L-arginine d	2.39E-05	0.001224869	0.000549359	0	0.00010529	0.000372156	0	0.000247639	0.000860003	0
biotin biosy	0.003169619	0.004719571	0.005597713	0.001421554	0.00553035	0.004704223	0.000753104	0.003501552	0.005846252	0.002392592
superpathw	0.007222357	0.007185951	0.006255181	0.013889723	0.007379658	0.009298909	0.014511902	0.008788966	0.00386593	0.008372509
Calvin-Bens	0.005372843	0.007035349	0.006467112	0.004657879	0.005857644	0.005901309	0.010844928	0.007288411	0.006766786	0.004801966
catechol de	0	0.000526624	0.000153288	0	0	6.43E-05	0	0.000138775	0.00025119	0
pyruvate fer	0.000308613	0.000200043	0.000472109	0.000160505	0.000430541	0.000421197	0.000199242	0.000435332	1.77E-05	0.000425039
L-citrulline	0.005075716	0.0033441	0.004672912	0.003475337	0.005440566	0.003263665	0.001738706	0.004163873	0.003797975	0.003322771
coenzyme A	0.013953341	0.007217585	0.008312826	0.012423172	0.010333392	0.009767241	0.010044239	0.012063343	0.008329521	0.01228571
coenzyme A	0.010682457	0.006989047	0.007766101	0.012423172	0.010198628	0.008799855	0.008988736	0.010782231	0.008612199	0.01189694
adenosylco	0.004069884	0.003518484	0.003704085	0.002645695	0.004642097	0.001265112	0.002007505	0.001470184	0.001046775	0.004937949
colanic acid	0.001408732	0.002208719	0.002694381	0.001424086	0.001986714	0.001788796	0.000764305	0.002231563	0.001754401	0.00170786
superpathw	0.009024884	0.00759564	0.007434013	0.006582339	0.008827717	0.007857476	0.008203477	0.008020509	0.006573616	0.010388299
creatinine d	0	0.000218862	1.44E-05	0	0	0	0	4.18E-05	6.40E-05	0
L-lysine bio	0.000397782	0.002571036	0.002208119	0.000644778	0.000934919	0.001702561	0.000199435	0.001008708	0.003219935	0.001298649
superpathw	0.007270503	0.004488403	0.00596136	0.007303409	0.006071579	0.00566226	0.004963127	0.006297302	0.005034875	0.005591872
dTDP-L-rha	0.013347749	0.007920053	0.007671111	0.016349851	0.014551624	0.011603278	0.037839656	0.013664793	0.011849863	0.015441128
enterobacte	1.93E-05	0.000558901	0.00040493	0	5.06E-05	0.000245992	0	0.000160655	0.000821665	0
enterobacti	0	0.00173109	0.001683238	0	0.000622817	0.001101773	0	0.000791956	0.00101391	0
fatty acid &b	0.000229814	0.002486003	0.002375302	0.000177404	0.001458895	0.001565824	0.000203627	0.000704246	0.002109699	0.000311857
fatty acid el	0.002979206	0.004735092	0.005692719	0.001316996	0.005677494	0.004281915	0.000672499	0.005786427	0.005490322	0.002055793
superpathw	0.002368363	0.004433224	0.006084514	0.000878125	0.004704133	0.003275093	0.000449015	0.004763673	0.0055162	0.001442979
mixed acid	0.000177419	0.002848128	0.001959545	2.54E-05	0.000517761	0.001336955	0.000151672	0.000776452	0.002117453	0.000447799
superpathw	0	0.000633078	0.001253132	4.58E-05	0.000246331	0.000552286	1.61E-05	0.000119763	0.000852236	0
superpathw	0.000412548	0.001697128	0.001394328	0.000301893	0.000701801	0.001239261	7.60E-05	0.000560282	0.001375658	0.000416565
fucose degr	0.000210456	0.00143148	0.000858548	0.000144022	0.000370544	0.000695102	3.44E-05	0.000312108	0.000904259	0.000199771
superpathw	0.000771847	0.001892405	0.002592547	0.001552807	0.002423656	0.001799648	0.000246341	0.001736044	0.000761565	0.002009839
D-galactara	0.000110115	0.001676987	0.001127737	6.02E-05	0.000388829	0.000905443	5.19E-05	0.000407094	0.001319318	9.23E-05
D-galacturo	0.000685784	0.001950209	0.002385963	0.001490498	0.002297362	0.00192207	0.000373968	0.00147297	0.000911763	0.002465549
superpathw	0.000912322	0.001825834	0.002081508	0.000790069	0.001321563	0.000992351	0.000493309	0.000879795	0.002261337	0.000344799
D-glucarate	4.75E-05	0.001139763	0.000579178	4.30E-05	0.000249096	0.000523694	3.14E-05	0.000269919	0.001090409	8.54E-05
superpathw	0.000110115	0.001676987	0.001127737	6.02E-05	0.000388829	0.000905443	5.19E-05	0.000407094	0.001319318	9.23E-05
gluconeoge	0.002747226	0.004322421	0.003725219	0.001848678	0.002456546	0.002744709	0.000911176	0.002755118	0.003257201	0.002196502
glucose and	8.91E-05	0.001642072	0.001107858	0	0.000338081	0.000658597	8.59E-05	0.000324049	0.000671875	0.000152542
superpathw	0.000821227	0.001728341	0.002510421	0.001768045	0.002762412	0.001656393	0.000376065	0.001732924	0.000631028	0.002805406
GABA shun	0.000124926	0.002584396	0.0027269	3.34E-05	0.000783637	0.002523114	0.00097031	0.001147131	0.001885459	9.32E-06
L-ornithine	0.004447079	0.005036891	0.004363499	0.004204045	0.005796254	0.002689892	0.009501159	0.004954999	0.001965101	0.003547306
glycogen de	0.000177163	0.001719315	0.001036558	5.35E-05	0.000391042	0.000716939	0.000169682	0.000445753	0.001157229	0.000299739
glycogen bi	0.001358538	0.003054389	0.002871521	0.001549563	0.001669443	0.00192532	0.002159673	0.005380822	0.002284262	0.00238366
superpathw	1.61E-05	0.000125528	0.000124573	0	2.61E-05	4.92E-05	0	6.65E-05	0.00041522	0
superpathw	0.001929974	0.002696968	0.002566344	0.001344899	0.001866869	0.001241678	0.000537212	0.001661674	0.001607291	0.00167623
superpathw	0.000184739	0.002667005	0.001855613	0	0.000715618	0.001466132	8.45E-05	0.00088053	0.001861851	0
glycolysis I	0.007076598	0.005023447	0.003396745	0.004377606	0.006726469	0.004194373	0.005231902	0.004027818	0.002880305	0.00780265
glyoxylate c	5.21E-05	0.001343745	0.000850841	0	0.000244334	0.000666245	3.06E-05	0.000299969	0.000942275	0
superpathw	0.000101443	0.00153664	0.000547064	2.47E-05	0.000252587	0.00048427	5.97E-05	0.000404565	0.001025374	4.30E-05
3-phenylpro	1.24E-05	0.000817885	0.000471178	0	0.000137424	0.000849275	1.27E-05	0.000185923	0.000640843	0
heme biosy	5.71E-05	0.001021997	0.000735484	0.000204746	0.000217425	0.000602536	0.000165885	0.000181895	0.000981406	0.000188054
heme biosy	8.18E-05	0.000899331	0.000945761	0.000215189	0.000264674	0.000470477	0.000162807	0.000138752	0.00064573	0.000294468
superpathw	0.00126309	0.002679879	0.002491524	0.00045356	0.001736799	0.001567239	0.000165108	0.001387738	0.001693493	0.001262936
L-histidine	0.010571951	0.004937244	0.006004029	0.007043326	0.008386362	0.008513376	0.00112457	0.007939048	0.006755398	0.010724815
L-histidine	0.00743957	0.004860057	0.006100766	0.010256244	0.009411094	0.009376971	0.015078363	0.007046076	0.007421475	0.010580153
L-methionin	0.000313386	0.001650414	0.001755745	9.29E-05	0.000792113	0.001166861	2.89E-05	0.000869663	0.002726997	0.000338838
L-methionin	0.004214086	0.004142086	0.003651486	0.003920817	0.002333063	0.001997081	0.002218302	0.003640204	0.00318917	0.002281915
L-isoleucine	0.009820743	0.008521657	0.008669922	0.014874169	0.011698655	0.011491735	0.015651524	0.009131097	0.010708387	0.013379439
superpathw	0	0.000412623	0.000258658	0	1.83E-05	0.000231737	0	0.00012215	0.000889956	0
ketoglucon	0	0.000272188	0.000220524	0	5.72E-05	0.000425577	6.53E-06	0.000131423	0.000552415	1.82E-05
lactose and	0	1.16E-05	1.47E-05	0	1.29E-05	4.88E-05	3.70E-05	0.000224684	4.02E-05	2.87E-05
L-leucine de	0	0	0	0	0	0	0	0	0	0
superpathw	0	4.93E-05	0.000365195	0	7.28E-05	0.000178238	0	1.84E-05	7.80E-06	0
superpathw	0.000639408	0.00289212	0.002944665	0.000212701	0.001344575	0.001995321	6.64E-05	0.001732685	0.003701107	0.000673253
methanoge	0.000159792	0.000108019	0.000164603	0	8.50E-05	1.57E-05	2.96E-05	0	0	4.78E-05
superpathw	6.92E-05	0.000569339	0.000602298	0	0.000470962	0.000544406	0	0.000143934	0	0.000294557
L-homoseri	0.000610026	0.002754492	0.002918715	0.000184108	0.001451302	0.002107692	5.76E-05	0.001613246	0.004162032	0.000654929
NAD salvag	0.001761097	0.000899827	0.001123878	0.001089675	0.001691455	0.002356362	0.000153703	0.000203443	0.001363273	0.000216225
lipid IVA bio	0.005134955	0.004180767	0.003078688	0.002850564	0.005533983	0.004477734	0.00072531	0.001807023	0.002207612	0.006973091
methyleryth	0	0	0.000378846	0	0.000160217	0	0.000268973	6.30E-05	0	0.001104217
pentose ph	0.002302531	0.006309161	0.004103007	0.001665125	0.003167287	0.00310138	0.00750436	0.003947599	0.00371448	0.002037151

O-antigen b	0.004339776	0.003105907	0.002568989	0.003224123	0.002182716	0.003519594	0.010218888	0.004947217	0.002994243	0.002589806
superpathw	5.51E-05	0.001338611	0.000882819	0	0.000235042	0.000463688	0	0.000363259	0.000698305	0
superpathw	0.000192001	0.001213649	0.000917485	0	0.000281637	0.000515154	0.000368191	0.000450912	0.000787135	0
TCA cycle I'	0.000165902	0.002384599	0.001290956	0	0.000269791	0.000760247	4.00E-05	0.000575153	0.001044832	0
pyruvate fer	0.001037686	0.001161795	0.00062251	0.000287775	0.001079026	0.000290243	0.000480481	0.003578517	0.000515388	0.000294013
heterolactic	0	0.000521295	0.000252892	0	4.81E-05	0.0001391	0	0.00011774	0.000690425	0
Bifidobacter	0	0	0.000176792	3.12E-05	0	0	2.71E-05	3.05E-05	0	0.000475838
acetylene d	8.24E-05	0.001460343	0.001299894	0	0.000331658	0.001118197	4.20E-05	0.000437646	0.001576138	8.89E-05
L-glutamate	0.000267076	8.36E-06	0	0	0.000117903	0.000346348	0	0.000374394	0.000232917	2.17E-05
L-lysine ferr	7.17E-05	0	0	0	0.000227663	0	0	0	4.99E-05	2.14E-05
purine nucle	0.000423579	0.001414689	0.001392341	0.000363305	0.001107358	0.001132429	0.000113395	0.000590317	0.000708173	0.000315717
formaldehyd	9.83E-05	0.002003933	0.000668409	9.78E-05	4.74E-05	0.000226237	7.16E-05	0.00033845	0.001346132	2.08E-05
octane oxid	1.90E-05	0.000849597	0.000274699	0	1.08E-05	0.000154126	0	0.000120475	0.000238001	0
reductive T	0	0.000254941	0.000250906	0	4.08E-05	0.000305947	0	0.000280542	0.000188313	0
superpathw	0.000272659	0.002240922	0.001758611	9.12E-05	0.000553497	0.001355374	9.08E-05	0.000964412	0.002401585	0.000904257
incomplete	0.00024622	0.000549897	0.000497524	0.00026928	0.000350519	0.000608657	2.93E-05	0.000753575	0.000842455	8.60E-05
superpathw	0.000564475	0.002488282	0.002298359	0	0.00137622	0.00154328	0.000565885	0.000997148	0.002466406	0.000617048
hexitol ferm	0.000485121	0.002030596	0.001790967	0.00012268	0.000889023	0.001173338	8.45E-05	0.0011153	0.001674338	0.000162825
myo-inosito	0	8.92E-05	5.70E-05	0	0	0	0	3.40E-05	0.000138419	0
phosphopa	0.008998991	0.006886735	0.007967771	0.010955274	0.009147528	0.010715722	0.009837516	0.007853483	0.008593122	0.012937267
pantothena	0.008706691	0.006622228	0.007275614	0.011666842	0.009340482	0.009468611	0.009399072	0.008725477	0.008370111	0.012100434
pentose ph	0.0025456	0.003462049	0.004056345	0.001882069	0.003322686	0.003552929	0.001332077	0.002094836	0.003912069	0.002475305
peptidoglyc	0.01530296	0.008256274	0.008417462	0.016287335	0.010797767	0.009668415	0.017234177	0.01217969	0.008515036	0.012600261
superpathw	0.003909199	0.004532953	0.004286544	0.001742926	0.001941486	0.003691631	0.002719488	0.002186548	0.003756971	0.002342496
superpathw	0.000546669	0.000414816	0.000526877	2.84E-05	0.00053674	0	0.001616586	0.000165959	0	1.72E-05
superpathw	0.000632199	0.002008984	0.00174009	6.50E-05	0.000804644	0.001097388	0.001582051	0.002207687	0.00090947	9.06E-05
polyisopren	0.001773879	0.001372639	0.002469186	0.001031449	0.001540989	0.001501927	0.000656243	0.000340297	0.00221197	0.001042205
ppGpp bios	8.98E-05	0.000789531	0.000654349	0.000161077	0.000320194	0.000226691	2.25E-05	0.000370223	0.000905829	0.000232719
protocatech	1.79E-05	0.000636705	0.000167728	0	0	9.28E-05	0	0.000170944	0.000441798	0
superpathw	0.00129549	0.003602284	0.004182615	0.003222584	0.003229021	0.003641784	0.001030978	0.003170458	0.003855703	0.001596925
photosynthe	5.52E-06	3.19E-05	0	0	0	8.84E-06	0	0	0	0
glycolysis IV	0.012991302	0.009351857	0.009802946	0.018697373	0.009954736	0.008868655	0.015317698	0.013066274	0.012259961	0.011113577
CMP-3-deo	0.002342725	0.003007066	0.002390539	0.00751858	0.004287134	0.003026373	0.011397652	0.005337382	0.00262707	0.003429788
superpathw	0	0.000220655	0.00013148	0	0	2.10E-05	0	4.90E-05	9.09E-05	0
formaldehyd	0	0.000357754	0.000102419	0	3.97E-05	0.000101098	0	4.52E-05	7.98E-05	0
Entner-Dou	0	0	0	0	0	0	0	0	0	0
C4 photosy	0.000132035	0.003116859	0.002324978	1.62E-05	0.000865271	0.001857576	0.000260952	0.00071669	0.001770565	0.000785574
trehalose de	9.53E-05	0.001033907	0.000582818	0	0.00021938	0.000377397	8.73E-05	0.000222483	0.000636154	0.000158126
L-lysine bios	0.000990331	0.000244803	0.001475788	0.002381114	0.001864892	0.001151378	0.000848594	0.001179018	0.00040318	0.001356861
L-lysine bios	0.008167844	0.006337515	0.00691238	0.011558107	0.008780336	0.009862403	0.011692375	0.009092985	0.008206197	0.010054011
superpathw	0.007705172	0.00653921	0.006469961	0.008318609	0.007097635	0.009458799	0.008205157	0.007771125	0.004701419	0.008472503
aerobic resp	1.44E-05	3.74E-05	5.78E-05	0	0	0	0	8.38E-06	0.000268069	1.75E-05
sucrose deg	0.000118424	0.001190006	0.000424854	0	0	0.000309586	0	0.000257162	0.000606042	0
folate transf	0.013943517	0.007516992	0.007681199	0.01230394	0.010801263	0.010673895	0.009797337	0.011346332	0.008180472	0.011845683
γ-g	0.000633683	0.001882575	0.001612425	0.000861573	0.000649617	0.001267299	0.00012656	0.000838768	0.003568992	0.000656433
pantothena	0.010605446	0.007105027	0.007766749	0.011260201	0.009923357	0.008852965	0.007778767	0.010801106	0.008136472	0.011713686
L-glutamate	0	0.000432378	0.000117649	0	0	5.18E-05	0	9.59E-05	0.000151843	0
phytate deg	0	0.000320408	0.00053215	0	0.000312763	0.000471208	1.30E-05	9.38E-05	1.37E-05	6.36E-06
L-proline bio	0.004771201	0.001182423	0.003984476	0.002186068	0.002425441	0.002510271	0.000978327	0.001038271	0.003045128	0.002715055
urea cycle	0.004017946	0.002854069	0.003900296	0.002684462	0.004696612	0.002355391	0.001470242	0.00353887	0.002118943	0.002366142
superpathw	4.14E-05	0	0	0	0	0	0.000102009	0.000390888	2.96E-05	0
biotin biosy	0	0.000172624	0.000204213	0.000307911	0.000302298	0.000307954	0.002842759	1.74E-05	0.000366833	1.30E-05
4-aminobut	0.000207041	0.002173823	0.002542224	3.34E-05	0.000772016	0.002538457	0.000963867	0.000985704	0.001911663	6.38E-05
L-histidine c	0	1.78E-05	0	0	0	0	0	0.000162727	3.91E-05	0
L-histidine c	0.006476578	0.004804573	0.005202375	0.006679648	0.006936351	0.008884257	0.001140547	0.006271865	0.006350866	0.011131038
NAD/NADH	0.000155821	0.002257913	0.001889064	0.000264476	0.000549825	0.001414039	0.00011167	0.000622502	0.001151828	0.000236553
L-glutamate	0	0	0	0	3.47E-05	0	0	7.83E-05	0.000417605	0
L-lysine bios	0.009683713	0.006741054	0.00741374	0.012215271	0.008972631	0.0065679	0.013841559	0.009301508	0.007765577	0.010661753
pyruvate fer	0.001761002	0.003128736	0.001867887	0.000726912	0.001661978	0.001322829	0.001189967	0.001608201	0.001259943	0.002327891
L-isoleucine	0.006553333	0.006780733	0.00558292	0.013464391	0.006475069	0.008591379	0.01427042	0.008352889	0.00315794	0.007409185
L-isoleucine	0.001165952	0.001625717	0.001550157	0.000768907	0.002381908	0.002007115	0.000460048	0.007436543	0.000795307	0.000965639
superpathw	0	0	0.000296949	0	0.000124207	0	0.000191382	4.63E-05	0	0.000807135
fatty acid &b	0.000236354	0.002523779	0.002280952	0.00015545	0.001542271	0.001573097	0.000201785	0.000716429	0.002147182	0.000318826
unsaturated	0.000316785	0.001073086	0.000605493	0.000147611	0.000376116	0.000833514	0	0.00041314	0.001751047	0.000111567
L-arginine b	0.005525293	0.004482834	0.004714117	0.003982088	0.006366027	0.003144888	0.009438733	0.005611098	0.00242432	0.004260824
superpathw	0.000113521	0.001348677	0.001053662	0	0.000218337	0.000542969	1.39E-05	0.000245986	0.001150845	1.83E-05
glutaryl-Co	0.000243973	0.002513494	0.002415071	0.000855991	0.001498884	0.001111068	0.000218146	0.00100105	0.002111519	0.000471904
toluene deg	0	0	0	0	0	0	0	0	0.00061098	0
toluene deg	0	0.000687624	0.000177598	0	0	0.000105196	0	0.000154999	0.000397957	0
toluene deg	0	0	0	0	0	0	0	0	0.00061098	0
tetrapyrrole	0.000949967	0.002463576	0.001565268	0.000950532	0.000822282	0.001820486	0.005291022	0.00287416	0.001437543	0.000830308
tetrapyrrole	1.37E-05	0.000741479	0.000682754	2.64E-05	0.000311455	0.000552321	1.64E-05	0.00017547	0.000513116	0
peptidoglyc	0	0	0	0	0	0	0	0	0	0
superpathw	1.50E-05	1.76E-05	0	2.45E-05	0	0	0.000820811	0.000343401	0	9.10E-05
superpathw	0.000598231	0.002885816	0.00212542	2.07E-05	0.001621472	0.001075138	0.000183008	0.001895703	0.002805637	9.89E-05
superpathw	0.000701293	0.002971169	0.003217995	0.000214212	0.001634614	0.0023996	6.71E-05	0.001711236	0.004632363	0.000756215
petroselinat	0	0.000253781	0.000303287	0	0.000111352	0.000231262	0	0.000140138	0.000424604	9.03E-06
sucrose deg	8.99E-05	0.000959435	0.00055342	0	0.000213702	0.000353684	7.38E-05	0.000215279	0.000673181	0.000464346
reductive T	0	0.000149253	0.00018907	0	3.35E-05	0.000158584	0	0.000122475	0.000120313	0
catechol de	0	0.000668761	0.000195137	0	0	8.81E-05	0	0.000161084	0.000322281	0
aromatic co	0	0.000668761	0.000195137	0	0	8.81E-05	0	0.000161084	0.000322281	0
superpathw	0.000268647	0.000488365	0.000331263	0	0.000225337	0	5.75E-05	0.000631063	0.000108023	8.67E-05
glycolysis II	0.006739964	0.004919996	0.003399416	0.004284865	0.006699584	0.004131099	0.003854395	0.003929662	0.002885019	0.007399712
L-glutamate	0.000638737	0.001013659	0.001183332	0.002050947	0.000705423	0.000266216	0.000387643	0.001345823	0.000191853	0.001065666
superpathw	9.45E-05	0.001966981	0.001229056	0	0.000307599	0.000789325	3.64E-05	0.000499493	0.00109373	0
2-amino-3-c	0	0.000317894	2.21E-05	0	0	1.10E-05	0	1.44E-05	2.91E-05	0
mannosylgl	0	0.000181009	0.000273255	0	0.000148258	0.000370227	0	5.17E-05	4.12E-05	0
GDP-mann	0.002234926	0.002380277	0.004164882	0.001373008	0.004627595	0.001933216	0.0005983	0.003970513	0.001528204	0.003415815
CDP-diacyl	0.008234801	0.006019404	0.006738521	0.00871669	0.008612977	0.009522343	0.008593453	0.00923494	0.007284525	0.009869045
nitrate redu	6.20E-05	0.001146246	0.000877574	1.11E-05	0.000390254	0.000773046	0	0.000401498	0.001069648	0

acetyl-CoA	0.000697154	0.000682487	0.000497196	6.56E-05	0.001174925	0.001028438	0.000230147	0.000172692	0.000626728	0.000339895
succinate fe	6.92E-05	0	1.88E-05	0	0.000124878	0.000192132	0	0	0	0
UMP biosyn	0.015574975	0.008615815	0.00870981	0.01647682	0.011040778	0.009835853	0.016512824	0.012307719	0.009104924	0.01282546
TCA cycle II	0.000408945	0.002212493	0.001079353	0.000420411	0.000210139	0.000639589	0.001375558	0.000705036	0.000829091	0.000257875
allantoin de	0	3.41E-05	5.05E-05	0	0	5.56E-05	0	0	3.07E-06	0
urate biosyn	0.012270902	0.007251979	0.008312938	0.014679713	0.011034788	0.009428734	0.012504401	0.011607856	0.0094746	0.011445572
allantoin de	0	0.000103352	9.71E-05	0	2.71E-05	0.000150064	4.88E-06	4.83E-06	0.000131445	0
Rubisco shu	2.77E-05	0.001980511	0.001296977	0	0.000419295	0.000853358	3.83E-05	0.000578962	0.001740743	0
2-methylcitr	1.43E-05	0.000346288	0.000386643	0	7.28E-05	0.000335794	0	8.80E-05	0.000377726	0
1,4-dihydrox	0.000143488	0.001579996	0.001001521	0	0.000426976	0.00073932	8.01E-05	0.000439617	0.002073492	7.98E-05
1,4-dihydrox	0.000143488	0.001579996	0.001001521	0	0.000426976	0.00073932	8.01E-05	0.000439617	0.002073492	7.98E-05
superpathw	0.000475482	0.003491501	0.002489407	0	0.001336253	0.002105914	0.000257183	0.001272331	0.004327682	0.00026944
superpathw	0.000377112	0.002839249	0.001486288	0	0.001093143	0.001462888	0.000148185	0.001292078	0.002883069	0.000232526
superpathw	0.00028515	0.003106856	0.002326991	0	0.001130242	0.002120939	0.000215594	0.000938744	0.002883069	0.000267968
superpathw	0.000186788	0.002839249	0.001486288	0	0.000210106	0.001462888	0	0.000842326	0.002883069	0
ubiquinol-7	0	0.000248077	0.000225258	0	4.92E-05	0.000162432	0	0.000117532	0.000725503	0
ubiquinol-9	0	0.000248077	0.000225258	0	4.92E-05	0.000162432	0	0.000117532	0.000725503	0
ubiquinol-10	0	0.000248077	0.000225258	0	4.92E-05	0.000162432	0	0.000117532	0.000725503	0
superpathw	0.000125377	0.002207041	0.001076468	0	0.00014107	0.001025768	0	0.000583221	0.002206486	0
superpathw	0.000322564	0.00282166	0.00191464	0	0.000932868	0.001511052	0.000172813	0.000898658	0.003577355	0.00018094
superpathw	0.000192092	0.002453447	0.001771957	0	0.000783349	0.001522662	0.000144682	0.000652864	0.002206486	0.000179944
superpathw	0.000162363	0.001478315	0.00109773	0	0.000471681	0.000802626	8.92E-05	0.000302137	0.002221881	9.06E-05
superpathw	0.000186788	0.002839249	0.001486288	0	0.000210106	0.001462888	0	0.000842326	0.002883069	0
superpathw	0.00041929	0.003370877	0.002311906	0	0.00119318	0.001932187	0.000230545	0.001187407	0.004121273	0.000236697
superpathw	0.00041929	0.003370877	0.002311906	0	0.00119318	0.001932187	0.000230545	0.001187407	0.004121273	0.000236697
superpathw	0.00041929	0.003370877	0.002311906	0	0.00119318	0.001932187	0.000230545	0.001187407	0.004121273	0.000236697
TCA cycle V	0.000106654	0.002909831	0.002221364	1.30E-05	0.000724303	0.00178763	0.000227332	0.000600684	0.001926165	0.000657802
superpatha	4.70E-05	0.001088217	0.000916348	5.94E-05	0.000348168	0.000770791	3.86E-05	0.000259981	0.001162844	0
superpathw	1.90E-05	0.000559068	0.000395613	0	0.000146909	0.000244768	1.82E-05	0.000163591	0.000666839	0
glycogen de	0.00030383	0.000182275	0.001233325	0.001033818	0.000679759	0.002127559	0.000218831	0.0004736	6.41E-05	0.000670923
palmitate bi	0	0.00412332	0.004918009	0	0.003933785	0.00390838	0	0.004236682	0.005052989	0.000651947
cis-vaccena	0.007394476	0.005593127	0.006797638	0.009476447	0.009519029	0.009526352	0.004850761	0.007326694	0.007176439	0.009160399
stearate bio	0.002486171	0.004378906	0.005581723	0.001017291	0.005044679	0.003652889	0.000508078	0.005257187	0.005318703	0.001637895
palmitate bi	0	0	0	0	0	0	0	0	0	0.000524994
superpathw	0	4.26E-05	5.13E-05	0	2.77E-05	0.000124023	0	8.78E-06	0	0
superpathw	0	0.002343163	0.002820944	0	0.001448249	0.002174968	0	0.001718597	0.003303434	0.00014305
5-aminoimid	0.011717024	0.007960418	0.008726214	0.012126016	0.010282313	0.009458983	0.008637935	0.009946487	0.00806393	0.012304798
5-aminoimid	0.011561552	0.008485442	0.008880485	0.015670858	0.010489837	0.009474849	0.01331998	0.009824649	0.008859216	0.013184823
inosine-5'-p	0.011390645	0.0062219	0.00747281	0.01082273	0.00945855	0.009753325	0.00442587	0.009256884	0.007791731	0.009076599
inosine-5'-p	0.010773127	0.005893109	0.007128498	0.009895773	0.008993172	0.009710159	0.003842555	0.008912473	0.007478735	0.008517215
superpathw	0.00584199	0.003390957	0.005427332	0.005949616	0.005316484	0.007177822	0.00582316	0.004782261	0.007858922	0.00473687
superpathw	0.007195575	0.005837674	0.006530021	0.008328298	0.006046925	0.007134467	0.007020483	0.007716775	0.005939734	0.005622561
CMP-pseud	0	2.48E-05	3.28E-05	0	0	3.07E-05	0	0	0	0
6-hydroxym	0.006883883	0.00470237	0.005435813	0.012627313	0.00572968	0.004778045	0.013578961	0.006210033	0.006392535	0.00785514
S-adenosyl-	0.009672071	0.007045064	0.007814537	0.012257497	0.007929357	0.009084519	0.013610673	0.003757485	0.007782471	0.012256568
chorismate	0.010996665	0.008272842	0.008388279	0.008987003	0.009600482	0.010016438	0.009571931	0.00980465	0.007658746	0.013166764
flavin biosyn	0.006643981	0.004024207	0.005592684	0.009056414	0.004636494	0.005020372	0.007044156	0.006245644	0.005678732	0.005067329
superpathw	0	0.000521688	0.000140701	0	0	7.85E-05	0	0.000136182	0.000294406	0
4-methylcat	0	0.000619251	0.000125502	0	0	8.25E-05	0	0.000152835	0.000362352	0
2-aminophe	0	0.000317894	2.21E-05	0	0	1.10E-05	0	1.44E-05	2.91E-05	0
4-chlorober	1.80E-05	0.000110272	8.20E-05	0	0	5.72E-05	0	1.67E-05	0	0
sucrose deg	0.000638658	0.002298435	0.00158863	0.00080666	0.002156698	0.001954786	0.00039344	0.002141019	0.001609322	0.000943532
starch biosy	0	0	0	0	0	0	0	5.19E-05	0	0.000324197
superpathw	0.000307948	0.000211669	0.000165034	0.000104271	0.00105724	0.000351573	0.000113659	0.002886833	1.04E-05	0.000314466
isoprene bio	0	0	0.000150583	0	6.21E-05	0	0.000104682	2.43E-05	0	0.000521123
superpathw	0.011561552	0.008485442	0.008880485	0.015670858	0.010489837	0.009474849	0.01331998	0.009824649	0.008859216	0.013184823
palmitoleate	0.002499875	0.004437031	0.005443131	0.001026218	0.00503654	0.003637698	0.000521814	0.005324351	0.005127114	0.001640597
superpathw	0	0.000898638	0.001005803	0	0.000355456	0.000690286	0	0.000521273	0.00146118	2.98E-05
superpathw	0	0.001532304	0.001739816	0	0.000567765	0.001054446	0	0.000967263	0.002353667	0
putrescine b	0.00197821	0.00357509	0.00384783	0.002331427	0.004590179	0.002228011	0.001364769	0.004092502	0.002991563	0.002261016
galactose d	0.003059654	0.002950282	0.003269149	0.001463597	0.002360115	0.001159961	0.001279078	0.004191762	0.001767868	0.002261911
L-phenylala	0.000121785	0.000637398	0.000240622	1.89E-05	0.000158793	0.000370772	0	0.000105609	0.000502938	6.04E-05
purine nucle	0.000822586	0.002350993	0.002263562	0.000910428	0.001997152	0.001746285	0.000117041	0.00078473	0.000976733	0.000916174
peptidoglyca	0.015235146	0.008492731	0.008417462	0.016183095	0.011030119	0.00984511	0.01710605	0.01217969	0.008607006	0.013114322
UDP-N-ace	0.015529949	0.008306587	0.00836909	0.016229392	0.010738815	0.009472688	0.017335059	0.0121944	0.008384034	0.012597905
UDP-N-ace	0.015305173	0.008355695	0.008351316	0.016460085	0.010834079	0.009605588	0.017201898	0.012157264	0.008429393	0.01266235
4-hydroxybe	0	0	0	0	0	0	0	0	0	0.000114709
peptidoglyca	0.001114263	0.00015946	0.000332429	0	0.000926796	0	0	8.57E-05	0	0.000708948
peptidoglyca	0.001484261	0.000190342	0.000421724	0	0.001097197	0.000906931	0.000329741	8.98E-05	0.000474858	0.000728196
GDP-D-glyc	6.95E-05	0	5.95E-05	0.000107393	0.00018552	8.47E-05	2.87E-05	8.17E-05	0	4.99E-05
4-deoxy-L-t	0.000590009	0.001939102	0.002368016	0.001377087	0.002246304	0.000828177	0.000318153	0.001435172	0.001072936	0.002355835
8-amino-7-c	0.002781424	0.004532559	0.005495348	0.001191451	0.005278227	0.004117342	0.000609577	0.003834153	0.005387984	0.001949071
stachyose c	0.001580249	0.002543581	0.002459886	0.001465305	0.00205339	0.001004301	0.001105945	0.00348752	0.001626033	0.001795933
mannitol cy	0.000111329	0.000850206	0.000678694	8.67E-05	0.00022509	0.000790662	6.39E-05	0.000231811	0.000464745	0.000194502
pyrimidine c	0.003576955	0.001683943	0.002812827	0.004412536	0.002038835	0.000735809	0.004953837	0.003126835	0.002211689	0.00199063
L-glutamine	0.000205019	0.000748526	0.000847978	5.04E-05	0.000306532	0.000387497	0.000334153	0.000759339	0.000291219	0.000382052
norspermidi	6.69E-05	4.66E-05	0.000132753	0	0	2.77E-05	0	6.39E-05	3.36E-05	0
chondroitin	0.000171281	2.33E-05	0.000120785	0.000390092	0.000466489	0.000200745	2.48E-05	0.000179949	0	8.52E-05
pyruvate fer	0.000201419	0.000156683	9.86E-05	6.61E-05	0.000692441	0.000218685	0.000191805	1.85E-05	0.000439306	9.17E-05
superpathw	0.000381955	0.000253931	0.000576544	0.00020052	0.000529784	0.00051524	0.00025025	0.000520647	2.27E-05	0.000534885
superpathw	0.000181862	0.000906103	0.00057561	0.000295681	0.000898103	7.00E-05	8.54E-05	0.000585248	6.44E-05	0.000564577
guanosine r	0.000419127	0.001383839	0.001137305	0.000462477	0.001004047	0.000973464	9.54E-05	0.000557087	0.000459445	0.000550565
guanosine r	0.000726146	0.00209942	0.002624921	0.000778535	0.001858362	0.002026973	0.000227671	0.000678311	0.00090265	0.000693383
adenine and	0.013461174	0.006994477	0.007680468	0.014452112	0.010246606	0.008726587	0.014855907	0.011077434	0.008314113	0.011364613
superpathw	0	0.000433112	0.000875692	3.06E-05	0.000165436	0.000374079	1.08E-05	8.01E-05	0.000590652	0
superpathw	3.88E-05	0.002692393	0.002542187	0	0.000852065	0.00163364	0.000209892	0.001285777	0.002156868	0
superpathw	4.68E-05	0.003644597	0.002397723	0	0.000937483	0.001285626	0	0.001370502	0.003595004	0
superpathw	7.12E-05	0.00220581	0.001501177	0	0.000747758	0.000628548	0	0.0011997	0.001679846	0
cinnamate a	1.24E-05	0.000817885	0.000471178	0	0.000137424	0.000849275	1.27E-05	0.000185923	0.000640843	0
queuosine b	0.015142916	0.00693346	0.007538517	0.012822969	0.009329122	0.009966598	0.016203953	0.008412926	0.009273355	0.013014884

preQ0 biosy	0.005841256	0.004918726	0.007394316	0.009301523	0.006599677	0.007838099	0.006730476	0.005164086	0.007311483	0.00944977
ubiquinol-8	0	0.000248077	0.000225258	0	4.92E-05	0.000162432	0	0.000117532	0.000725503	0
starch degra	0.000114521	0.001151985	0.000671393	0	0.000260518	0.000474291	9.72E-05	0.000276357	0.000636583	0.000193182
starch degra	0.015204269	0.007655108	0.00867344	0.005882687	0.010906909	0.01050425	0.007302355	0.01129626	0.00327307	0.012786159
CMP-legion	0	0	7.31E-05	0	0.00019101	0.000160361	0	0.000156945	0	5.42E-05
hydrogen pr	9.61E-06	5.66E-05	0	0	0	1.45E-05	0	0	0	0
phosphatidy	8.16E-05	0.001640288	0.000805087	9.95E-05	0.000208457	0.000558865	0	0.000305877	0.001349524	3.35E-05
molybdenur	0	0.000151938	0.000261713	0	2.36E-05	0.000136519	0	6.58E-05	0	0
fatty acid be	1.47E-05	0.000285642	0.000144445	0	3.53E-05	0.000137077	0	0.000118067	0.000793269	0
all-trans-far	0.001252849	0.000929957	0.001818956	0.00071603	0.001097654	0.00099799	0.000602883	0.000217624	0.001425429	0.000631737
isopropanol	8.92E-05	0	0	0	1.44E-05	5.57E-05	0	0	0	0
thiazole bios	0.000417278	0.002118928	0.002454334	0	0.000812415	0.001526555	0.000396677	0.0009821	0.001559238	0.000163304
thiazole bios	0.004858843	0.005141512	0.003622139	0.003958897	0.005979624	0.00401882	0.000574745	0.004113952	0.001877542	0.007090094
superpathw	0.001708439	0.004501101	0.004423916	0	0.002841795	0.003770908	0.001706243	0.003286058	0.004023005	0.00076764
thiamin salv	0.006201221	0.005401758	0.004992986	0.010882943	0.005469054	0.003872107	0.010789562	0.006149773	0.004789503	0.007158094
superpathw	0.001932173	0.003148763	0.002903614	0.001180152	0.001844654	0.002099287	0.000730248	0.001817177	0.002683252	0.001590801
seleno-amir	0.005831542	0.004263024	0.006347496	0.003810431	0.004001486	0.006491622	0.001812324	0.002977311	0.008466446	0.003859864
dTDP-3-ace	0.000151705	0	0	0	0	0	0	0	0	0
erredoxin ox	0.000473065	0.002541938	0.001390327	0.00050291	0.000429692	0.000822465	6.10E-05	0.000775413	0.001114592	0.000233023
glycerol deg	0.000209727	0.000425003	0.000262661	0	0.000130603	0.000388282	0.000142591	5.33E-05	0.00094297	5.32E-05
L-1,2-propa	0.000110051	0.003112265	0.001324048	3.94E-05	0.000537064	0.000928502	0	0.000570027	0.001614254	0.000131225
phosphatida	0	9.15E-06	0	0	0	0	0	0	0	0
4-coumarat	0	0.000219933	0.000238113	0	0.000106288	0.000135629	0	4.99E-05	6.01E-06	0.000178215
fatty acid sa	0	0.000100719	0.000199892	0	0	0.000216403	0	0.000118268	0.001134859	0
pyruvate fer	0.009820743	0.008521657	0.008669922	0.013540743	0.011698655	0.011491735	0.015651524	0.009131097	0.010708387	0.013379439
C4 photosy	2.97E-05	0.000961415	0.000689945	2.65E-05	0.000106684	0.000358624	0.000292187	0.00066764	0.001632927	0.000223292
C4 photosy	0.000157848	0.003276431	0.002667529	1.94E-05	0.000998803	0.002042554	0.000289451	0.000773247	0.002430188	0.000903959
L-ascorbate	0	0.000230464	0.000229742	0	0	0.000129329	0	0.000101989	0.00050962	0
pyrimidine d	0.005486583	0.00263862	0.003771358	0.004505283	0.00465283	0.003789877	0.00449121	0.003737061	0.003219096	0.003824523
pyrimidine d	0.005875582	0.00330025	0.003743769	0.004923276	0.004379748	0.00342215	0.003841824	0.004811443	0.002832841	0.003709546
superpathw	0.000316382	0.000521406	0.00071153	0.000128281	0.000584306	0.000487352	7.93E-05	5.64E-05	0.00033375	0.000797061
pyrimidine d	0.003929039	0.001788642	0.002436678	0.003166356	0.003270253	0.0021702	0.004562466	0.002641124	0.001762857	0.002517147
pyrimidine d	0.000129418	0.000968471	0.001203629	0.000345197	0.000205249	0.000592464	0.001743015	0.001041717	0.001816694	0.000890363
pyrimidine d	0.013995394	0.005535219	0.006911013	0.013046834	0.009216799	0.009768809	0.009329706	0.008591169	0.008192955	0.010818592
superpathw	9.31E-05	0	0	0.000202702	0	0	0	0	0	8.78E-05
pyridoxal 5'-	0	0.000299607	0.000485338	0	0.000280409	0.000483113	8.21E-06	0.000123444	0.000208087	0
superpathw	0.005193238	0.002338545	0.003028426	0.004699477	0.00398861	0.003050094	0.006973335	0.003568372	0.002719563	0.003154221
superpathw	2.21E-05	5.97E-05	8.43E-05	0	2.16E-05	6.08E-05	0	2.21E-05	6.34E-05	0.000112565
pyrimidine d	6.78E-05	0.001143088	0.001459718	0.00034258	0.000243673	0.00067687	0	0.001292421	0.001959563	0.000445029
superpathw	0.00046487	0.001842745	0.002259791	0.000606883	0.000834524	0.001128658	0.000117421	0.002116729	0.003263584	0.000992521
adenosine r	0.015256933	0.007593466	0.008176493	0.013894888	0.010029717	0.009903965	0.01708122	0.011359163	0.008633683	0.01155446
adenosine d	0.004524426	0.004704553	0.005305448	0.005435239	0.004100931	0.006336204	0.003952904	0.006361727	0.007272702	0.003751069
guanosine r	0.012945059	0.007323336	0.007922863	0.015093089	0.01100004	0.009751073	0.012895361	0.011521019	0.009697504	0.012320691
guanosine d	0.004524426	0.004704553	0.005305448	0.005435239	0.004100931	0.006336204	0.003952904	0.006361727	0.007272702	0.003751069
superpathw	0.006596389	0.003329993	0.005712347	0.006269515	0.006019884	0.007779919	0.006757671	0.00472232	0.008371202	0.005369031
superpathw	0.008769533	0.006406154	0.007206687	0.009662256	0.007162893	0.007720592	0.008232775	0.00840414	0.005972886	0.006781691
inosine-5'-p	0.003845249	0.00492702	0.003295187	0.006601575	0.005106168	0.003101222	0.001283209	0.00808579	0.002884463	0.003768542
myo-, chiro-	0.004400478	0.004041984	0.003303254	0.006508564	0.006002274	0.002855307	0.010516825	0.004100532	0.001806499	0.007140111
D-fructurona	0.000585776	0.001520767	0.002346487	0.001370482	0.002193477	0.001394261	0.000318653	0.001392688	0.000699446	0.002332912
superpathw	0	9.57E-05	0	0	0	4.90E-05	0	2.71E-05	0.000619389	0
superpathw	0.008640899	0.006066581	0.00630608	0.007523606	0.008172616	0.005110489	0.007572423	0.007799051	0.006240337	0.009490863
TCA cycle V	0.000149702	0.001933637	0.000970723	4.10E-05	0.000190702	0.000557496	3.42E-05	0.000432697	0.000782989	0
NAD/NADP	0	7.61E-05	0	0	0	4.01E-05	0	2.20E-05	0.000737344	0
NAD/NADP	2.02E-05	0.000809147	0.000466355	0	5.78E-05	0.000289778	0	0.000182163	0.00079788	0
aerobic resp	1.00E-05	3.62E-05	5.33E-05	0	0	0	0	8.15E-06	0.000245308	0
4-amino-2-m	0.004842534	0.003905202	0.003486526	0.004845294	0.00464905	0.003321519	0.001986885	0.003764935	0.003425781	0.007336721
fatty acid &b	0.000184127	1.20E-05	5.11E-05	7.85E-05	0.00025494	0.000658331	0	9.07E-06	2.92E-05	6.95E-05
xylose degr	0	7.09E-05	0	0	0	0	0	5.96E-05	0.000274395	0
dTDP-D-&b	0.000113596	0.001451852	0.001831523	0.000173718	0.000841532	0.006010574	0.000147915	0.000294982	0.000979563	0.003270847
dTDP-N-ace	0.000962402	0.001197214	0.000620567	0	0.000242332	0.000517028	2.97E-05	0.000310655	0.001512559	7.16E-05
dTDP-N-ace	0.000119405	1.19E-05	0	6.58E-06	0	9.17E-06	0	0	0	0
superpathw	0.001789315	0.002026094	0.003424733	0.001438311	0.003252948	0.001424348	0.000525163	0.001707039	0.001475929	0.003517956
superpathw	0.000112393	0.001081429	0.000644518	0	0.00024793	0.000456542	0.000108907	0.000281035	0.000745755	0.000182145
superpathw	0.00065093	0.001727109	0.001523689	0.001042548	0.001050976	0.002283741	0	0.000574986	0.001399074	0.001805003
superpathw	0.000111205	0.001136615	0.000400178	0	0	0.000291688	0	0.000241727	0.000571581	0
thiamin form	0.006161123	0.005006885	0.004546254	0.009507415	0.004392508	0.002940873	0.010548689	0.004790448	0.003851508	0.006318782
1,4-dihydrox	0.000203404	6.84E-05	0.000100793	0	0.000671422	0	0.001642591	0.004134765	0	0.000272943
1,4-dihydrox	0	0	0	0	0	0	0	0	0	0
anaerobic e	0.005217356	0.001777082	0.000593263	0.000684525	0.000482784	0.000220857	0.00010302	0.002170631	9.01E-05	0.000267281
1,3-propane	0	0.00062317	0.000843086	0	0.00033147	0.000596656	0	0.000156606	9.23E-06	0
octanoyl-[ac	0.002454299	0.004468925	0.005652909	0.001058869	0.004477502	0.00310041	0.000525656	0.004924269	0.005264137	0.001597845
superpathw	0	5.48E-05	0	0	0	0	0	0	0	0
taxadiene b	0	0	0.00016738	0	6.91E-05	0	0.000111241	2.63E-05	0	0.000530022
L-arginine b	0.007367882	0.005134852	0.004791295	0.003696943	0.005967517	0.002625656	0.008303069	0.006796832	0.002289011	0.004200273
phospholipi	0	0.000132101	0.000202962	0	3.08E-05	0.000186523	0	3.60E-05	0.000138831	0
sulfoglycoly	0	0.00019176	0.000241171	0	3.66E-05	0.000126222	0	5.44E-05	0.000497384	0
mannan deg	0.001545769	0.002110829	0.001979034	0.001034483	0.004415543	0.000933281	0.000378268	0.002400121	0.000987881	0.003224939
6-hydroxym	0.006720237	0.004670182	0.005095805	0.012530868	0.00581226	0.004652044	0.014503586	0.005982557	0.006309172	0.007540487
methylerythr	0	0	0.000323301	0	0.000126456	0	9.18E-05	5.74E-05	0	0.000458283
methanol ox	0	0.000106592	0.000104097	0	8.64E-06	4.94E-05	0	1.64E-05	0	0
gondoate bi	0.006643462	0.005141088	0.006190701	0.008527929	0.008930596	0.009080918	0.004090826	0.006677886	0.00637998	0.00834936
oleate biosy	0.002757401	0.004635901	0.005617138	0.001173044	0.005369686	0.003983939	0.000600039	0.005639648	0.005395106	0.001857855
superpathw	0.000136904	0.001743051	0.001230599	0	0.000288124	0.000353352	0	0.000591697	0.000660998	0
superpathw	0.008166857	0.004310988	0.006229078	0.007654688	0.006902924	0.006294685	0.006079357	0.006024035	0.005623984	0.006280625
superpathw	3.17E-05	0.001030158	0.000718563	0.00019154	0.000109922	0.000452093	0.000545025	0.00094646	0.001554619	0.000239927
ADP-L-glyc	0.000951872	0.00156681	0.000750003	0.000592126	0.000661266	0.001031013	0.001438583	0.001366688	0.000888225	0.000781243
anhydromur	0.004729683	0.003465869	0.002527565	0.002924237	0.002437433	0.003656851	0.003150485	0.003662153	0.00288777	0.002866427
3-phenylpro	2.69E-05	0.000945362	0.000669679	0	0.000165432	0.000822543	2.62E-05	0.000252961	0.000660524	0
purine ribon	0.006520653	0.003861191	0.00411346	0.00266514	0.003169007	0.002222497	0.007872754	0.005574379	0.002319518	0.002601751
superpathw	0.000125442	0.001937554	0.001897723	5.20E-05	0.000512055	0.001716592	6.36E-05	0.000639544	0.002068518	0.000144669

superpathw	0.000101605	0.001783264	0.001487845	0	0.000476737	0.001138211	5.12E-05	0.001107114	0.001667165	0.000138452
CDP-diacylg	0.008234801	0.006019404	0.006738521	0.00871669	0.008612977	0.009522343	0.008593453	0.00923494	0.007284525	0.009869045
polymyxin r	5.48E-06	0.000499792	0.000205311	0	4.83E-05	0.000191248	0	0.000146596	0.000570726	0
superpathw	3.59E-05	0.000819153	0.00063794	0	0.000134187	0.000389745	1.84E-05	0.000130838	0.000711716	0
tRNA proce	7.22E-05	0.002727387	0.00196964	0.000559239	0.00056332	0.001828893	0.000667874	0.000780867	0.001812843	0.001081183
methylphos	0	0.000123301	0.000184446	0	1.19E-05	0.000166526	0	2.83E-05	3.08E-05	0
peptidoglyc	0.006939977	0.008794277	0.008712656	0.010914939	0.008633651	0.010971683	0.016423908	0.004256023	0.00902119	0.011128218
superpathw	0.006996436	0.0039013	0.003871736	0.007351726	0.005388396	0.00299746	0.003323972	0.005778892	0.005029054	0.005745038
superpathw	0.006670549	0.003471947	0.003970892	0.005196109	0.005015165	0.003990444	0.005014815	0.005075096	0.003384202	0.004141176
phenylaceta	0	3.53E-05	4.50E-05	0	2.62E-05	0.000115164	0	7.29E-06	0	0
allantoin de	0	9.49E-05	0.000108287	0	2.75E-05	0.0001002	0	6.15E-06	4.58E-06	0
2-methylcitr	0	0.000352655	0.000407014	0	8.45E-05	0.00034262	0	8.47E-05	0.000363036	0
aspartate su	0.000284313	0.002264477	0.001804753	9.67E-05	0.000567064	0.001230865	9.58E-05	0.001006083	0.002390687	0.000898042
superpathw	0.004101027	0.003640032	0.003735345	0.003043185	0.004984204	0.00556706	0.00089084	0.002470531	0.003050752	0.007742001
(5Z)-dodec-	0.002508398	0.004509934	0.00551943	0.001023833	0.005007693	0.003645672	0.000524663	0.005455146	0.005273174	0.001646485
superpathw	0	0.001457782	0.001179818	0	0.000198171	0.000272542	0	0.000414085	0.001024759	0
L-ascorbate	4.95E-05	0.000997439	0.000237877	0	0	0.000147524	0	0.000169447	0.000512974	0
stearate bio	3.29E-05	0.000534413	0.000477632	0	0.000161678	0.000323471	0	0.000183378	0.000579555	1.35E-05
nitrate redu	5.20E-05	2.22E-05	2.82E-05	2.68E-05	0	0	0	0	2.42E-05	0.000125563
phosphatidy	0.002585334	0.003720155	0.002954163	0.001118839	0.001238737	0.002229139	0.001807297	0.001215995	0.002143213	0.001486376
phosphatidy	0.002585334	0.003720155	0.002954163	0.001118839	0.001238737	0.002229139	0.001807297	0.001215995	0.002143213	0.001486376
superpathw	0.000101601	0.001393521	0.00143414	0	0.000404112	0.001373986	5.18E-05	0.000512598	0.001062351	0.00011042
biphenyl de	0	4.00E-05	0	0	0	0	0	1.08E-05	2.14E-05	0
ketogenesis	3.93E-05	0	1.42E-05	0	0	2.02E-05	0	0	0	0
fatty acid &a	2.59E-05	0	0.000211313	0	0	0	0	0	0	0
phytol degra	2.61E-05	0.00152032	0.000523935	0	3.36E-05	0.000204837	0	0.000206965	0.000308204	0
fatty acid &b	0.000227617	2.39E-05	0.000100059	0.00011682	0.000445272	0.000966438	0	1.79E-05	5.77E-05	0.000118527
TCA cycle II	0.00010391	0.000335476	0.000222312	3.03E-05	0.000135979	0	0	0.000442057	7.19E-05	0
gluconeoge	0.002483855	0.001492762	0.001219766	0.000946451	0.000945286	0.000454133	0.000152692	0.002081771	0.000143011	0.000596547
glycolysis V	0.002068711	0.003941185	0.003188692	0.001238746	0.001761669	0.002007221	0.000611694	0.002075519	0.003208446	0.001513388
superpathw	0.000153194	0.00215219	0.001663932	0.000155114	0.000577944	0.001902292	0.000153282	0.000766123	0.003030823	0.000423358
D-galactose	0.001997043	0.003301169	0.00314482	0.001463597	0.002194645	0.001159961	0.002567817	0.00555892	0.001996737	0.002261911
mycolate bi	0.002831248	0.00476381	0.005670306	0.001208514	0.005450287	0.004016375	0.000609727	0.005949492	0.005243716	0.001899238
NAD salvag	5.08E-05	0.001855288	0.001473636	0	0.000821374	0.001116549	8.05E-05	0.000685163	0.001179303	0.001203724
NAD biosyn	0.007924273	0.005067295	0.006047177	0.011168412	0.007752741	0.006942345	0.008837199	0.006965994	0.006856217	0.00843753
pyridoxal 5'-	0.003586653	0.003942321	0.003814168	0.003031022	0.00522761	0.007286872	0.000720456	0.002288499	0.002608443	0.007664076
TCA cycle V	7.23E-05	0.000620623	0.00038171	2.40E-05	0.000135594	0.000297689	0	0.000284457	0.000640595	0
L-rhamnose	0.002009833	0.001969612	0.00264669	0.003045285	0.00259928	0.003765446	0.005623414	0.002755282	0.002177096	0.002825055
flavin biosyn	0.008292259	0.00543655	0.006566873	0.013012285	0.007668017	0.008448826	0.019070136	0.006193239	0.006451169	0.008108971
adenosine r	0.00048505	0.001566981	0.002294879	0.000540688	0.00159064	0.001018872	5.89E-05	0.000570741	0.000578169	0.000583343
superpathw	0.006171272	0.004899252	0.005593726	0.005395953	0.007303452	0.00892532	0.006783545	0.007552716	0.005449734	0.006880027
sulfate redu	0.000231657	0.003175941	0.002711081	7.57E-06	0.000799508	0.002130413	6.86E-05	0.00098594	0.003637521	3.65E-05
superpathw	0.000489835	0.00382799	0.003302438	1.70E-05	0.001528508	0.002020361	0.000144991	0.001833555	0.002686491	8.11E-05
superpathw	9.53E-05	0.001959591	0.001316404	0	0.000414164	0.001032379	4.41E-05	0.000530907	0.001395296	0
TCA cycle I	0.000740875	0.003292756	0.002590376	0.000780423	0.001093248	0.002120414	0.000112874	0.001607168	0.002520713	0.000481863
teichoic acid	0.001097198	0.000462898	0.000596454	0.000678209	0.001261358	0.001767258	0.000408023	0.000217879	0.00017583	0.000288044
superpathw	0.006793699	0.005999534	0.005005717	0.008257049	0.007083466	0.00529505	0.002326258	0.006698565	0.004408686	0.009416895
superpathw	0.005792425	0.005014681	0.005967616	0.00598963	0.006517174	0.00821412	0.003281667	0.003398821	0.006388986	0.009167364
superpathw	0	0.001044168	0.000358397	0	0.000110784	0.00039976	0	0.000329956	0.000534242	0
superpathw	0.008813393	0.005992508	0.007199078	0.00652202	0.007625132	0.009928459	0.006296611	0.007187662	0.008870273	0.009269179
tRNA charg	0.013562908	0.007086823	0.007545715	0.013676475	0.009532735	0.008971078	0.01356405	0.011044868	0.008347371	0.010768867
L-tryptopha	0.004458778	0.004545766	0.003718608	0.002720609	0.002589243	0.003227185	0.008301145	0.001901094	0.006993914	0.005147498
L-tyrosine d	0	0	0	0	0	0	0	0	6.18E-05	0
superpathw	0	0.000288635	0.00025639	0	5.57E-05	0.000183596	0	0.000132009	0.000795415	0
UDP-N-ace	0.002919922	0.002172302	0.001601613	0.001901009	0.001224177	0.002135815	0.007475342	0.003627179	0.001755531	0.00147237
superpathw	0	3.41E-05	5.05E-05	0	0	5.56E-05	0	0	3.07E-06	0
ureide biosy	2.83E-05	0.000384867	0.000234162	0	0	0	0	0	1.64E-05	0
L-valine bio	0.009820743	0.008521657	0.008669922	0.014874169	0.011698655	0.011491735	0.015651524	0.009131097	0.010708387	0.013379439
	W61	W62	W63	W64	W66	W67	W6	W70	W71	W9
N10-formyl-	0.010300529	0.008984633	0.006522936	0.011881878	0.006864502	0.00793602	0.007514365	0.006197935	0.008185339	0.011098172
4-hydroxyph	1.23E-05	0.000319227	0.000842414	1.43E-05	0.000664402	0.00022178	0.000241008	0	0	0
aerobactin b	7.01E-05	0	0	0	6.04E-06	2.74E-05	0	0.000269556	0	0
superpathw	7.84E-05	0.0001011	0.000171214	2.50E-05	0.000139011	0	4.34E-05	3.99E-05	0	0
homolactic f	0.004282726	0.004934937	0.004203195	0.003667127	0.004278462	0.002982258	0.003450103	0.004716305	0.005632084	0.004133983
glycolysis III	0.007389918	0.006492722	0.006599538	0.008434535	0.005525085	0.003080051	0.004960763	0.006284183	0.007509041	0.008061902
superpathw	0.001170589	0.001368487	0.002281392	0.000735996	0.003088324	0.002155021	0.001696827	0.002977929	0.000787557	0.000423531
superpathw	0.000134694	0.000711263	0.00145132	0.000115894	0.001701641	0.000733681	0.000372706	0.000162965	0	0
L-ornithine d	0.004923708	0.005639172	0.003694846	0.009027579	0.001295825	0.001362028	0.002412554	0.004615015	0.00530583	0.011526984
arginine, orn	0.000285775	0.000114335	2.86E-05	0.000200956	6.18E-05	9.03E-05	0	0.000817489	0	4.37E-05
L-arginine b	0.00449819	0.005789882	0.004545677	0.007547815	0.005771676	0.008304867	0.00844398	0.003727589	0.010322991	0.003284419
L-arginine b	0.004456945	0.005478165	0.004506231	0.007287814	0.005687028	0.008142724	0.008204773	0.003834331	0.010155511	0.002978461
chorismate	0.008328983	0.008599669	0.007218174	0.010678816	0.006857943	0.006189503	0.008183681	0.005628946	0.011331856	0.008341816
superpathw	0.006054031	0.005359747	0.005391864	0.005712209	0.005095383	0.003901611	0.005902706	0.005981681	0.004233879	0.010507901
L-arginine d	0.000162138	0.000726531	0.001668105	3.57E-05	0.001452301	0.000950973	0.000396276	0.001131441	5.66E-06	0
biotin biosyn	0.003651538	0.004419035	0.004422015	0.002690474	0.003245637	0.002751663	0.003011469	0.005434607	0.002526464	0.002271608
superpathw	0.009507898	0.007493668	0.006773757	0.008153002	0.00692112	0.007727607	0.010517457	0.007750954	0.006899903	0.011636902
Calvin-Bens	0.007468121	0.007697802	0.007364945	0.006173052	0.007920074	0.006911126	0.009459258	0.006453003	0.006784329	0.00518048
catechol de	1.21E-05	0.000158599	0.000699404	2.99E-05	0.000659891	0.000291032	0.000163177	0	0	0
pyruvate fer	0.000481112	0.000320913	0.000161015	0.000543518	0.000297244	0.000317371	0.000507237	0.000311987	0.000226004	3.05E-05
L-citrulline b	0.003648649	0.002078962	0.001779542	0.00196821	0.000906807	0.000470626	0.003584083	0.003332281	0.005038822	0.004854205
coenzyme A	0.011131355	0.009849855	0.007629108	0.01541956	0.008025994	0.012262016	0.011133508	0.006029871	0.010092963	0.013693905
coenzyme A	0.010785682	0.009862278	0.006698297	0.014350811	0.006942469	0.01113312	0.009672211	0.005709648	0.008822039	0.012481813
adenosylco	0.0063805	0.002514091	0.00270187	0.002390674	0.002417473	0.003142261	0.001897138	0.003674491	0.006072661	0.005183923
colanic acid	0.002118236	0.002087058	0.002387567	0.001540166	0.001688884	0.001380935	0.001699121	0.003378873	0.003446044	0.001854086
superpathw	0.007994646	0.008531321	0.006494343	0.010297659	0.006459057	0.00618411	0.008509712	0.005757003	0.010394003	0.008582494
creatinine d	0	1.17E-05	0.000138461	0	0.000127187	0	3.22E-05	0	0	0
L-lysine bio	0.001582097	0.002598515	0.003299835	0.000924987	0.003084612	0.002366692	0.001666454	0.003276614	0.001438422	0.000190808
superpathw	0.006686435	0.004719592	0.005501424	0.005981344	0.004946517	0.006835895	0.005986866	0.004715204	0.007379226	0.006628464
dTDP-L-rha	0.013826485	0.011629021	0.00748789	0.01478248	0.010295976	0.013143748	0.013468717	0.006262966	0.013326248	0.014565664

enterobacte	0.000103273	0.000573353	0.0010949	6.48E-05	0.000950175	0.000230615	0.000273245	0.000264048	0	0
enterobactir	0.001105749	0.001287397	0.001914313	8.17E-05	0.002071239	0.001865972	0	0.001996547	0	0.000131854
fatty acid &b	0.001468573	0.0018763	0.002222515	0.00027648	0.002341738	0.002104119	0.000676726	0.00436655	0.000362536	7.30E-05
fatty acid ele	0.003586262	0.004668112	0.004848383	0.002534351	0.00462569	0.003831693	0.003329393	0.005699962	0.002543752	0.001992427
superpathw	0.002669448	0.003791349	0.004572417	0.001835327	0.004080136	0.002880088	0.002638041	0.00652645	0.001975569	0.001348643
mixed acid f	0.001035454	0.00162296	0.003058686	0.000665254	0.003177922	0.00224401	0.000998701	0.003120408	0.001125987	0.000108305
superpathw	0.000214576	0.000113687	0.000240314	0.000334274	0.000457274	0.000513006	7.04E-05	0.000773682	4.23E-05	0.000150166
superpathw	0.001409972	0.000686725	0.001821445	0.000689397	0.001686298	0.0007756	0.001090738	0.002445973	0.000203812	0.000268347
fucose degr	0.000969822	0.000362182	0.001291468	0.000361117	0.001349157	0.000513278	0.000828107	0.002175162	9.39E-05	0.000127223
superpathw	0.001788709	0.001694537	0.001587794	0.001432383	0.001159709	0.001721791	0.001174573	0.002804188	0.005065333	0.001984024
D-galactara	0.000675814	0.000943208	0.001363554	6.22E-05	0.001619014	0.000732868	0.000353515	0.00319896	0.001424631	5.19E-05
D-galacturo	0.001522833	0.001729561	0.001671333	0.00152919	0.001362159	0.002023212	0.001206178	0.002245654	0.004766205	0.001926783
superpathw	0.00165571	0.002024145	0.002178446	0.002140858	0.001421603	0.002137892	0.002249562	0.003740112	0.002296158	0.00053165
D-glucarate	0.000312473	0.000737788	0.001169895	4.67E-05	0.001378677	0.000569326	0.000297321	0.001014163	0.000172107	4.56E-05
superpathw	0.000675814	0.000943208	0.001363554	6.22E-05	0.001619014	0.000732868	0.000353515	0.00319896	0.001424631	5.19E-05
gluconeoge	0.002797101	0.003088937	0.005323763	0.003092378	0.004777096	0.002749649	0.002577778	0.004094842	0.002180405	0.000534241
glucose and	0.000529605	0.000633314	0.001777099	0.000131164	0.001688623	0.001179585	0.000629236	0.0015759	0.000783354	2.35E-05
superpathw	0.001712643	0.001802802	0.00141412	0.001639836	0.000934932	0.001953324	0.001399389	0.002553026	0.004973642	0.002260627
GABA shun	0.000673901	0.001939245	0.002777436	0.000285822	0.001029731	0.00020793	0.001570446	0.002626289	0.001136348	5.53E-05
L-ornithine I	0.003168579	0.005346408	0.00428017	0.006623206	0.005315396	0.007316378	0.008266023	0.004088769	0.009121173	0.00258882
glycogen de	0.000715113	0.001178701	0.002292773	0.000144646	0.002353621	0.0019789	0.001088072	0.001491497	0.001474207	4.69E-05
glycogen bi	0.002295211	0.002870581	0.003858301	0.00220593	0.003762301	0.003459604	0.002873796	0.003731227	0.005142117	0.002257236
superpathw	0.000167802	2.31E-05	0.000293029	1.91E-05	0.000189752	4.68E-05	0	0.00020093	0	0
superpathw	0.001819821	0.001952055	0.002985864	0.001394712	0.002281433	0.001722512	0.001564671	0.002597624	0.001716492	0.00042306
superpathw	0.001234236	0.001436609	0.002751068	0.000172674	0.002937493	0.001637192	0.000989431	0.00284853	2.56E-05	6.50E-05
glycolysis I (	0.005244527	0.004399201	0.004847792	0.002241253	0.0045576	0.003132757	0.003797064	0.004714764	0.005349152	0.007054877
glyoxylate c	0.000634613	0.000606385	0.001396893	4.98E-05	0.00159623	0.000820168	0.00038227	0.001470124	7.29E-06	3.31E-05
superpathw	0.00032748	0.000418647	0.001682825	0.000175669	0.001511487	0.000708936	0.000629532	3.86E-05	0.000139614	0.000112206
3-phenylpro	2.87E-05	0.000302672	0.000929369	2.16E-05	0.000962187	0.000282027	0.000155979	0.001220233	0	0
heme biosy	0.000352558	0.000758755	0.000998787	0.000127933	0.001204292	0.001257817	0.000291599	0.001831294	0	2.78E-05
heme biosy	0.000384125	0.000555821	0.000682271	0.000253269	0.001043896	0.001818986	0.000383131	0.001590073	0.000162777	4.62E-05
superpathw	0.002001408	0.001909115	0.002694508	0.000829211	0.002784487	0.001656682	0.001850784	0.003598017	0.00091134	0.000239183
L-histidine d	0.007801886	0.004079438	0.005606839	0.003986717	0.003924211	0.004107412	0.003792895	0.003288869	0.003985843	0.012783651
L-histidine b	0.00689582	0.007704578	0.00479097	0.010834308	0.005030907	0.007266341	0.009633321	0.005457176	0.007005368	0.01008657
L-methionin	0.000805044	0.000963662	0.001464038	0.000302192	0.001730042	0.001162238	0.00046212	0.002591881	0.000844964	0.000505476
L-methionin	0.005252082	0.003905406	0.004273903	0.003555036	0.004345624	0.004517572	0.002424429	0.003121791	0.003387058	0.001660374
L-isoleucine	0.009604616	0.01048792	0.00706537	0.013300109	0.007541223	0.009049675	0.011907215	0.009840952	0.013274384	0.012639369
superpathw	9.89E-05	0.000439117	0.000575963	3.86E-05	0.00014864	7.90E-05	0.000187462	0.000208079	0	0
ketoglucona	0.0003551	0.000314864	0.000339472	8.14E-05	0.00039058	0.000199183	0.000145259	0.001227685	3.93E-06	0
lactose and	0.000147187	2.06E-05	8.60E-05	0.00022062	8.01E-05	0.000217383	0.000123167	0.000134448	0.000601611	3.56E-05
L-leucine de	0	0	5.09E-06	0	0	0.000493634	0	0	0	0
superpathw	2.29E-05	0	0	0	0.000130349	0	0	0.000385237	0	0
superpathw	0.001593259	0.001862252	0.002645657	0.000660874	0.003077667	0.002282835	0.000949531	0.003675839	0.00167041	0.000997532
methanoge	0	2.08E-05	0	6.64E-05	0	0.000164587	4.49E-05	0.000189826	0.000309906	0.000143846
superpathw	0.0004039	0.000266586	0.000719555	9.48E-05	0.000149251	0.000400057	0	0.000771338	1.78E-05	1.83E-05
L-homoseri	0.001491392	0.001741586	0.002484002	0.000588298	0.002885245	0.002072861	0.000881309	0.003883429	0.001559744	0.000969039
NAD salvag	0.001344309	0.000831798	0.001351597	0.000708168	0.000882379	0.000349782	0.000675749	0.001222463	0.002360372	0.000909439
lipid IVA bio	0.003702623	0.002800809	0.002882457	0.001482305	0.002900901	0.00359205	0.001563713	0.003233318	0.00060161	0.006818843
methyleryth	0.000429938	0	0.000137139	0.000609174	6.95E-05	0	0.000640713	0.000835168	0.003214046	0.000268521
pentose pho	0.003933697	0.004446228	0.00630826	0.002235624	0.006665814	0.003324103	0.006522131	0.005633638	0.003827427	0.002194823
O-antigen b	0.002702487	0.003383678	0.002811733	0.003981701	0.003854581	0.006031418	0.007300603	0.002803545	0.003843229	0.000609511
superpathw	0.000134694	0.000711263	0.00145132	0.000115894	0.001701641	0.000733681	0.000372706	0.000162965	0	0
superpathw	0.000321566	0.000620556	0.00106062	8.75E-05	0.001355728	0.000654838	0.000272999	0.00127485	0	0
TCA cycle I'	0.000854339	0.00104219	0.002514832	0.000202147	0.002653272	0.001418424	0.0006937	0.001959238	0	6.34E-05
pyruvate fer	0.002127475	0.000927674	0.001889642	0.001223565	0.001331774	0.000242563	0.001433047	0.000640634	1.18E-05	6.59E-05
heterolactic	0.000125781	0.000405148	0.000349413	8.69E-05	0.000544506	0.000304782	6.64E-05	0.000195408	0	0
Bifidobacter	9.21E-05	0	1.49E-05	0.000521031	4.86E-05	0.00020399	0.000114623	0.000339713	0.000557713	0.000117059
acetylene d	0.000600973	0.00072986	0.001992322	5.30E-05	0.001900772	0.000519729	0.000632085	0.0020721	4.18E-05	2.94E-05
L-glutamate	0.000304276	0	0	2.26E-05	2.19E-05	1.62E-05	0	0	0	6.15E-05
L-lysine ferr	0.000132829	0	0	0	0	0	0	0	0	0
purine nucle	0.000907693	0	0.001638519	0.000284396	0.001214685	0.000520156	0.000626414	0.001596693	0.000796326	0.000419404
formaldehy	0.000101453	0.000497419	0.002275166	0.000203504	0.002007133	0.000783954	0.000883766	3.89E-05	0	1.99E-05
octane oxid	3.54E-05	0.000214835	0.000925764	5.50E-05	0.000859006	0.000347743	0.000190505	3.27E-06	0	0
reductive T	0.000285603	0.000634002	0.000510658	0.000133661	0.000210763	1.62E-05	0.000132894	2.63E-05	0	0
superpathw	0.000851368	0.001869871	0.002591434	0.000764091	0.002705792	0.001819693	0.001130762	0.002303134	0.001300953	0.000149525
incomplete	0.000449907	0.000900211	0.001019827	0.000356307	0.00104108	0.000189769	0.000412964	0.001098765	0	0
superpathw	0.001505286	0.001726857	0.003058287	0.000548841	0.002475462	0.001240408	0.001468935	0.003653472	0.000167112	0.000156203
hexitol ferm	0.001546279	0.001525615	0.002358743	0.000433361	0.002446227	0.001294212	0.001379054	0.002753265	7.15E-05	3.99E-05
myo-inosito	0	0.000775458	0.000278417	1.90E-05	0.00035757	7.93E-05	0	4.00E-05	0	0
phosphopa	0.009131422	0.008394545	0.006040845	0.011236913	0.00681384	0.006529821	0.009477336	0.006030316	0.008111001	0.013919473
pantothena	0.009636752	0.008611934	0.006196759	0.012253953	0.00663577	0.007500666	0.009234705	0.005720447	0.00806842	0.012131213
pentose pho	0.003633771	0.002177478	0.003721466	0.00176089	0.00243847	0.000915878	0.002440917	0.004303242	0.003550585	0.002851468
peptidoglyca	0.011747538	0.010544086	0.007530726	0.015234229	0.009245911	0.010466432	0.013019425	0.006208244	0.010373386	0.012789434
superpathw	0.002616984	0.00378376	0.003783128	0.003534698	0.004271943	0.005251903	0.00235734	0.005102606	0.004324806	0.002465676
superpathw	0.000143992	0.000377821	0.000192737	0.000100828	0.000257055	0.000762223	0.000199378	0.000887327	2.95E-05	6.31E-05
superpathw	0.000673954	0.00078356	0.001531547	0.000390558	0.002147317	0.001276938	0.000963237	0.002493523	0.00051171	0.000226362
polyisopren	0.001225746	0.001008199	0.001474057	0.000812017	0.001170109	0.000667667	0.000710561	0.002246772	0.003201289	0.001844462
ppGpp bios	0.000282745	0.000795478	0.001309471	0.000213495	0.001302538	0.000470718	0.000432791	0.00038126	0	3.83E-06
protocatech	0	0.000192341	0.000919053	3.14E-05	0.000789112	0.000395753	0.000171849	0	0	0
superpathw	0.003326005	0.00392128	0.00440322	0.001144254	0.004023902	0.003252554	0.00378563	0.003743273	0.000573819	0.000327958
photosynthe	0	1.29E-05	3.70E-06	7.28E-06	0	0.000228189	0	0	5.13E-05	0
glycolysis IV	0.012984752	0.011358683	0.009378932	0.016719399	0.009795463	0.01007577	0.01270796	0.008101478	0.011165677	0.010088518
CMP-3-deo	0.003814775	0.003851656	0.003058818	0.002190076	0.00421323	0.004028604	0.005040461	0.003515235	0.002602188	0.003433234
superpathw	6.23E-06	6.77E-05	0.000296722	0	0.000363419	9.11E-05	9.14E-05	0	0	0
formaldehy	0.000536017	0	0.000237411	0	0.000198493	8.51E-05	0.000145998	0.001608409	4.81E-05	0.000133859
Entner-Dou	3.32E-05	2.87E-05	0.000195476	0	0.000153288	0	0	0	0	0
C4 photosy	0.000998373	0.001439322	0.002785138	0.000655591	0.002797529	0.00230316	0.00100489	0.002946921	0.00137553	0.000150668
trehalose de	0.000395008	0.000688624	0.001440341	7.45E-05	0.001418381	0.00127341	0.000587623	0.000868362	0.000836439	2.36E-05
L-lysine bios	0.00376671	0.000278957	0.002476054	0.00138662	0.000541191	0.000759682	0.000734103	0.003139929	0.003127937	0.003045961
L-lysine bios	0.008618481	0.00865629	0.005895965	0.010762672	0.006199361	0.007654862	0.008306364	0.005134036	0.008590455	0.010796514

superpathw	0.007903821	0.007010742	0.006502487	0.007675876	0.005278589	0.006120254	0.007481218	0.007144057	0.006946033	0.011514447
aerobic resp	1.29E-05	3.97E-05	4.80E-05	0	2.38E-05	0.001911833	0	1.27E-05	0.000143509	0
sucrose deg	0	0.00029227	0.001183634	0.000225635	0.001380176	0.000413768	0.000124784	0	0	0
folate transf	0.01063484	0.009358364	0.006869287	0.012487933	0.007076612	0.008210453	0.009643623	0.006155164	0.008667325	0.012312546
γ-g	0.001139265	0.00126062	0.002663556	0.001135242	0.001704139	0.001357268	0.001052449	0.003135797	0.000891516	0.000468134
pantothenat	0.010593068	0.009631916	0.00670047	0.014424968	0.006544782	0.010369454	0.009199935	0.005757336	0.009400251	0.011270239
L-glutamate	0	7.94E-05	0.000609352	0	0.000328207	0.000108337	8.89E-05	0	0	0
phytate deg	0.000360362	7.30E-05	7.07E-06	0	0.000236588	8.11E-05	0	0.002872618	0	2.10E-05
L-proline bio	0.001856195	0.001026541	0.001573042	0.001152895	0.000649567	0.001359349	0.001400812	0.002905068	0.003866636	0.003102026
urea cycle	0.003262894	0.00174724	0.001506903	0.001659073	0.000483948	0.000287817	0.003080484	0.002686779	0.003426933	0.003356804
superpathw	0	0	0	0	0	0	0	0	0	0
biotin biosyn	3.74E-05	0.000289336	0.000110284	0.000273925	0.000904927	0.000170227	0.002744351	0.000123214	0	9.08E-05
4-aminobuta	0.000651508	0.002071211	0.002443541	0.000288671	0.000853063	0.000884566	0.001317341	0.002413296	0.001133437	5.52E-05
L-histidine d	0	0	0.000139825	0	0.000133846	2.62E-05	2.05E-05	0	0	0
L-histidine d	0.007379239	0.003011195	0.003910181	0.00408316	0.002190942	0.000445666	0.003645418	0.003044851	0.004492645	0.013140005
NAD/NADH	0.001470215	0.000941962	0.001905733	0.000376594	0.002088176	0.000974825	0.000593286	0.003645715	0.00017553	0.000111622
L-glutamate	0.000238394	0	4.82E-05	0	7.40E-05	3.30E-05	0	9.77E-05	0	0
L-lysine bios	0.008959744	0.009276424	0.00611314	0.012835535	0.00701801	0.008892271	0.009653589	0.005190843	0.008828808	0.011043318
pyruvate fer	0.001305466	0.004104025	0.002315922	0.003143182	0.002887351	0.002053469	0.001681493	0.002032558	0.001249304	0.000439894
L-isoleucine	0.009263044	0.006841809	0.006556318	0.007200014	0.006626307	0.007391648	0.010092597	0.007251024	0.005933334	0.01099325
L-isoleucine	0.002070107	0.001394061	0.001627172	0.000694264	0.001694776	0.000460987	0.000547778	0.003103928	0.000209664	2.95E-05
superpathw	0.000314279	0	0.000105561	0.000426783	5.27E-05	0	0.000446627	0.000639265	0.002465563	0.000208661
fatty acid &b	0.001410282	0.001961473	0.002207149	0.000263751	0.002293436	0.002131823	0.000681263	0.003412037	0.00031039	6.18E-05
unsaturated	0.000516398	0.001757322	0.001913683	0.000122143	0.001633934	0.001635165	0.00050746	0.000830725	1.43E-05	3.73E-05
L-arginine b	0.004178836	0.005619329	0.00366824	0.007689917	0.004364193	0.005309308	0.008133283	0.003798578	0.006852399	0.003610398
superpathw	0.000279098	0.00093079	0.001459626	0.000155151	0.001877908	0.00138644	0.000321904	0.001432519	4.98E-06	1.69E-05
glutaryl-CoA	0.00106254	0.00206751	0.00214678	0.000614174	0.002681518	0.002499405	0.000831141	0.003093159	0.00311612	0.00100141
toluene deg	0	0	0	0	0	0	0	0	0	0
toluene deg	0	0.000200682	0.000925072	3.03E-05	0.00087342	0.000300277	0.000199371	0	0	0
toluene deg	0	0	0	0	0	0	0	0	0	0
tetrapyrrole	0.002253828	0.002054793	0.00168331	0.001152883	0.002704567	0.003534338	0.004581222	0.003186001	0.002299237	0.000973673
tetrapyrrole	0.00042403	0.000319182	0.000741888	1.99E-05	0.001042729	0.000531692	9.46E-05	0.001539926	0	0
peptidoglyca	0.000417224	0	0.000402429	0	0	0	0.000282042	0	0	0
superpathw	0.000304303	7.96E-05	0.000597169	6.09E-05	0.000183212	0	0.000731082	0.000436341	0.000496219	0
superpathw	0.002091704	0.003921463	0.003245113	0.000941696	0.003258336	0.002428922	0.002149362	0.002283896	0.00019627	0.000259107
superpathw	0.001668875	0.001945231	0.002729738	0.000677188	0.003098925	0.002196545	0.001001265	0.004159487	0.001765073	0.001118655
petroselinat	0.000202991	0.001057026	0.000231788	0	0.000465484	0.000201736	9.35E-06	0.000673289	0	5.12E-05
sucrose deg	0.000479794	0.000631102	0.001329473	0.000319508	0.00138694	0.001143674	0.000568007	0.001525011	0.000775719	6.46E-05
reductive TC	5.46E-05	0.000148857	0.000337825	1.90E-05	0.000158749	1.53E-05	0.000115656	0	0	0
catechol de	9.08E-06	0.000195789	0.000883371	3.48E-05	0.000826457	0.000330531	0.000207929	0	0	0
aromatic co	9.08E-06	0.000195789	0.000883371	3.48E-05	0.000826457	0.000330531	0.000207929	0	0	0
superpathw	0.00023312	0.000671155	0.000223309	0.00016978	0.000923907	0.001642406	0.000831903	0.001083488	2.72E-05	6.34E-05
glycolysis II	0.005210356	0.004071698	0.004676773	0.002189528	0.004036231	0.002028408	0.003638071	0.004639234	0.005120061	0.007042396
L-glutamate	0.000405377	0.001520262	0.000848982	0.00074785	0.00117669	0.001628467	0.001184688	0.000766987	0.001411391	0.000237562
superpathw	0.000642542	0.001053321	0.001920194	7.91E-05	0.002203737	0.001311742	0.000555981	0.002125372	0	3.97E-05
2-amino-3-c	0	1.30E-05	0.000213496	0	4.91E-05	3.51E-05	1.42E-05	0	0	0
mannosylgly	0.000279682	3.76E-05	8.67E-05	0	0.000184164	3.75E-05	0	0.001362869	0	2.72E-05
GDP-manno	0.002959248	0.002695956	0.002442082	0.002195272	0.001788472	0.00114949	0.001360235	0.003413229	0.003818393	0.002997295
CDP-diacylg	0.008190485	0.008189126	0.005685547	0.011164345	0.005622048	0.005767542	0.00820359	0.006217418	0.009576503	0.010670405
nitrate redu	0.000324465	0.000816641	0.000944449	6.56E-05	0.001432608	0.00039479	0.000266154	0.001542699	1.11E-05	0
acetyl-CoA	0.00096807	0.000118178	0.001072351	7.45E-05	0.000988415	0.000255801	0.000210832	0.001231811	3.90E-05	3.58E-05
succinate fe	0.000134373	0	0	0	0	0	2.84E-05	3.03E-05	0	0
UMP biosyn	0.011890245	0.011509967	0.008311938	0.016056637	0.009391914	0.010825216	0.014019331	0.005926439	0.010290566	0.01292422
TCA cycle II	0.001185633	0.000979582	0.002527887	0.000563921	0.002278132	0.001310388	0.001780198	0.001905566	0.001958299	0.000381249
allantoin de	2.44E-05	5.35E-06	0	0	1.86E-05	1.46E-05	0	0.000144546	0	0
urate biosyn	0.010272873	0.009786917	0.007426915	0.012731872	0.007836565	0.009525015	0.011424541	0.007413897	0.010131768	0.012105547
allantoin de	9.86E-05	1.95E-05	2.01E-05	0	7.52E-05	2.14E-05	0	0.00050506	0	0
Rubisco shu	0.000711259	0.001149867	0.001719256	5.16E-05	0.00211759	0.001675912	0.000569687	0.001900512	0.000123738	4.46E-05
2-methylcitr	0.000162327	0.0001144	0.000187214	2.85E-05	0.000332236	0.000258008	0	0.000837876	0	9.86E-06
1,4-dihydrox	0.000499268	0.001072551	0.001549933	0.000226172	0.001664524	0.001539493	0.000352298	0.001839313	6.76E-06	3.81E-05
1,4-dihydrox	0.000499268	0.001072551	0.001549933	0.000226172	0.001664524	0.001539493	0.000352298	0.001839313	6.76E-06	3.81E-05
superpathw	0.001524072	0.0026673	0.003125535	0.000724636	0.002933907	0.001898902	0.001036469	0.003529804	2.31E-05	0.000129747
superpathw	0.001192559	0.00152112	0.002796497	0.000599344	0.002520834	0.001620025	0.000853591	0.002712114	2.06E-05	0
superpathw	0.001377199	0.00152112	0.002954587	0.000631214	0.002520834	0.001620025	0.000898806	0.002712114	2.29E-05	0.000129753
superpathw	0.001192559	0.00152112	0.002796497	0.000271286	0.002520834	0.001620025	0.000853591	0.002712114	0	0
ubiquinol-7	2.87E-05	0.000367401	0.000587835	1.78E-05	0.00055852	0.000701335	0.000180736	0.00015191	0	0
ubiquinol-9	2.87E-05	0.000367401	0.000587835	1.78E-05	0.00055852	0.000701335	0.000180736	0.00015191	0	0
ubiquinol-10	2.87E-05	0.000367401	0.000587835	1.78E-05	0.00055852	0.000701335	0.000180736	0.00015191	0	0
superpathw	0.000825893	0.001087863	0.00218379	0.000182327	0.001938883	0.001165397	0.000587922	0.002220509	0	0
superpathw	0.00106699	0.002018228	0.002490958	0.000493725	0.002314904	0.001384859	0.000718987	0.003103414	1.54E-05	8.68E-05
superpathw	0.000959529	0.001087863	0.002329811	0.000428855	0.001938883	0.001165397	0.000620153	0.002220509	1.53E-05	8.68E-05
superpathw	0.000526665	0.00091019	0.001343701	0.000250487	0.001234193	0.001428475	0.000380286	0.001819916	7.72E-06	4.35E-05
superpathw	0.001192559	0.00152112	0.002796497	0.000271286	0.002520834	0.001620025	0.000853591	0.002712114	0	0
superpathw	0.001378623	0.002561411	0.003080883	0.000652779	0.003059325	0.003042009	0.000960121	0.003345903	2.02E-05	0.000113748
superpathw	0.001378623	0.002561411	0.003080883	0.000652779	0.003059325	0.003042009	0.000960121	0.003345903	2.02E-05	0.000113748
superpathw	0.001378623	0.002561411	0.003080883	0.000652779	0.003059325	0.003042009	0.000960121	0.003345903	2.02E-05	0.000113748
TCA cycle V	0.000870247	0.001313374	0.002839691	0.000590345	0.002864813	0.001807477	0.000902462	0.003466334	0.001172612	0.000121623
superpatha	0.000574623	0.001045229	0.001237725	4.50E-05	0.001430613	0.001401188	0.000412602	0.002005183	0	2.44E-05
superpathw	0.000381614	0.000443626	0.000839617	2.43E-05	0.001049653	0.000702397	0.000136693	0.000259529	0	0
glycogen de	0.00117729	0.000235696	0.000659213	0.000522741	8.40E-05	2.53E-05	0.000223528	0.002004247	0.003597831	0.002297197
palmitate bi	0.003319874	0.004785625	0.004118217	0	0.004327834	0.003438486	0.000741907	0.005298282	0	0.00158587
cis-vaccena	0.008607268	0.008211664	0.005487015	0.009058582	0.005651753	0.007676591	0.006587669	0.005475981	0.006660452	0.011233701
stearate bio	0.002968844	0.00372161	0.004433833	0.001968253	0.003532582	0.002798758	0.002690571	0.005413208	0.002109764	0.001571489
palmitate bi	0	0.000713092	0	0	0	0	0.000521929	0	0	0
superpathw	0	0	0	0	5.84E-05	0	0	0	0	0
superpathw	0.001876047	0.003993926	0.002240917	0	0.002982001	0.00183703	0.000150852	0.003993236	0	0.000634827
5-aminoimid	0.010662674	0.008092443	0.007447816	0.011512295	0.006313738	0.007887602	0.009280999	0.006739462	0.009713106	0.012678655
5-aminoimid	0.010938135	0.008968152	0.007466487	0.011736547	0.007882813	0.010133652	0.009928712	0.006814043	0.009292408	0.013239218
inosine-5'-p	0.010855655	0.006425494	0.006355965	0.010710384	0.005193788	0.006907646	0.007511733	0.005591613	0.0093242	0.012854275
inosine-5'-p	0.010509985	0.005899776	0.006077234	0.010115104	0.004201818	0.006193224	0.006799016	0.005354549	0.008947668	0.012573034

superpathw	0.005215764	0.003830691	0.004835575	0.004482578	0.004363895	0.00711235	0.006056314	0.004646206	0.007261869	0.006694079
superpathw	0.008284781	0.006147336	0.005877886	0.007765988	0.005135783	0.00737521	0.006994713	0.004976271	0.007201922	0.007053655
CMP-pseud	2.50E-05	0	0.000227281	2.74E-05	0	0	0	0	0	0
6-hydroxym	0.006450149	0.006065283	0.004657055	0.0068761	0.005537864	0.006467692	0.007990595	0.003710518	0.006076361	0.005797958
S-adenosyl-	0.005248922	0.008744604	0.004371886	0.009530119	0.007667531	0.013477337	0.007731687	0.004816218	0.010278223	0.01408964
chorismate	0.009890824	0.009492847	0.007540048	0.012666734	0.007459509	0.006152112	0.009637735	0.005806948	0.011183941	0.012894792
flavin biosyr	0.005881359	0.005085348	0.004783193	0.005640073	0.004352978	0.004547745	0.00553308	0.004149417	0.007250075	0.006121838
superpathw	0	0.000174224	0.00080979	0	0.000697809	0.000261294	0.000172792	0	0	0
4-methylcat	7.63E-06	0.000167585	0.000846896	2.92E-05	0.000780821	0.000284897	0.000172499	0	0	0
2-aminophe	1.46E-06	1.30E-05	0.000213496	0	4.91E-05	3.51E-05	1.42E-05	0	0	0
4-chlorober	0	1.51E-05	0	0	5.67E-05	2.41E-05	0	0	0	0
sucrose deg	0.001237603	0.001939623	0.002966913	0.001629998	0.002715831	0.001933121	0.002045908	0.002273193	0.001342061	0.000795706
starch biosy	0	0	0	0.000130892	0	0	0.000330378	0	0.001156085	0
superpathw	0.000799731	0.000564563	0.00036071	0.000543452	0.000191853	7.66E-05	0.000243479	1.21E-05	0	0
isoprene bic	0.000171447	0	5.30E-05	0.000252179	2.67E-05	0	0.000273355	0.000356893	0.001623625	0.000105331
superpathw	0.010938135	0.008968152	0.007466487	0.011736547	0.007882813	0.010133652	0.009928712	0.006814043	0.009292408	0.013239218
palmitoleate	0.003002883	0.004095974	0.004595282	0.002049669	0.004356496	0.003299712	0.002901091	0.005442396	0.002104653	0.001564958
superpathw	0.000602558	0.002343307	0.000905585	0	0.001460261	0.000788076	4.54E-05	0.0017403	0	0.000162019
superpathw	0.000828867	0.003018528	0.001541681	0	0.002248474	0.001333783	8.97E-05	0.002246841	0	0
putrescine b	0.00278053	0.003522562	0.003333746	0.004259756	0.003681786	0.003794743	0.002622172	0.004033965	0.004666353	0.00280871
galactose d	0.002329154	0.0025355	0.002891538	0.002989074	0.002589268	0.003566548	0.001753373	0.002952571	0.005343564	0.001962179
L-phenylala	0.000205053	0.000249125	0.001345488	8.84E-05	0.000937371	0.000917713	0.000300602	2.27E-05	0	0
purine nucle	0.001855432	0.000972963	0.000679869	0.000575971	0.001259058	0.002259742	0.000607788	0.002565226	0.002043934	0.001643087
peptidoglyca	0.011658573	0.010544086	0.007596397	0.015234229	0.009320621	0.01082589	0.013019425	0.006583991	0.010373386	0.013014169
UDP-N-ace	0.01172838	0.010619365	0.007549454	0.015414158	0.00944402	0.010758332	0.013278235	0.006190191	0.011417568	0.012770696
UDP-N-ace	0.011836395	0.010497873	0.007532937	0.015203227	0.00933835	0.010911045	0.012986688	0.006252817	0.011173952	0.012689797
4-hydroxybe	0	0	0	9.81E-05	0	0	3.36E-05	0	0	2.27E-05
peptidoglyca	0.000819234	0.000539351	0.000518555	0.000709361	0.000236991	0.000883971	0	0.000469837	0	0.000147831
peptidoglyca	0.000958386	0.000621583	0.00054197	0.000828201	0.000348526	0.00109447	0.000440787	0.000682847	0	0.000443095
GDP-D-glyc	5.17E-05	5.81E-06	0.000567315	2.23E-05	0	0	0	5.87E-05	1.10E-05	0
4-deoxy-L-t	0.001316139	0.001528163	0.00176077	0.001309523	0.001286761	0.001953657	0.001122514	0.001592402	0.004764559	0.001770537
8-amino-7-c	0.00325895	0.004153053	0.004532416	0.002302412	0.003453039	0.002653381	0.002758385	0.005425194	0.002255636	0.001860934
stachyose c	0.001972216	0.002265453	0.001852612	0.002503091	0.002442414	0.00368427	0.001441514	0.002708131	0.00461879	0.001589348
mannitol cyc	0.000789154	0.00027158	0.001017467	0.000240439	0.001067654	0.000369152	0.000313881	0.001177234	9.62E-05	4.71E-05
pyrimidine c	0.001482313	0.002306424	0.002124654	0.002838557	0.003215526	0.004853487	0.005063325	0.001406009	0.004407022	0.00166898
L-glutamine	0.000697978	0.001188177	0.000513034	0.000488331	0.002476666	0.001899751	0.001239263	0.000969006	0.001075855	0.000133152
norspermidi	3.07E-05	3.03E-05	0.00102272	6.90E-05	0.001163991	0.000411994	0.000262213	0	0	0
chondroitin	0.000164624	5.83E-05	0.000275179	9.39E-05	2.49E-05	0	3.27E-05	0.000208722	0.001293183	0
pyruvate fer	0.000452466	3.19E-05	0.000399219	0	0.000350119	5.67E-05	2.89E-05	0.001393562	0	0
superpathw	0.000576351	0.00040612	0.000205367	0.000686784	0.000376529	0.000399462	0.000621889	0.000385086	0.000277382	3.86E-05
superpathw	0.000748788	0.000687226	0.000331214	0.000365636	0.000512156	0.000176004	0.000383386	0.00019471	0.000481864	0
guanosine r	0.00100661	0.00075043	0.0003878	0.000348721	0.000633852	0.001246826	0.000429126	0.001331892	0.000945743	0.000890768
guanosine r	0.001630362	0.001376324	0.002611128	0.000428396	0.002634557	0.001791288	0.001064131	0.00318993	0.002960155	0.001876899
adenine and	0.0092797	0.009066717	0.00690586	0.013336845	0.007353785	0.009376214	0.01084701	0.006953332	0.010887546	0.011446458
superpathw	0.000143953	7.62E-05	0.000161875	0.000224941	0.000313925	0.000352121	4.71E-05	0.000536473	2.82E-05	0.000100465
superpathw	0.001443479	0.001439458	0.002944757	0.000557022	0.002872345	0.001155995	0.002163709	0.002344823	8.79E-05	0
superpathw	0.001083292	0.001843493	0.003734672	0.000676259	0.003548804	0.001839392	0.00259049	0.003148679	0.000105657	0
superpathw	0.000844312	0.001376094	0.002585636	0.000533427	0.002870996	0.000945285	0.001366057	0.00035675	8.58E-05	0
cinnamate a	2.87E-05	0.000302672	0.000929369	2.16E-05	0.000962187	0.000282027	0.000155979	0.001220233	0	0
queuosine b	0.007941447	0.009203191	0.006116848	0.01278327	0.006674473	0.008364518	0.01081066	0.005788175	0.009381068	0.013079227
preQ0 biosy	0.007186059	0.010538379	0.003942693	0.008795208	0.004127631	0.00487083	0.006590638	0.004533462	0.006989057	0.009253077
ubiquinol-8	2.87E-05	0.000367401	0.000587835	1.78E-05	0.00055852	0.000701335	0.000180736	0.00015191	0	0
starch degra	0.000474864	0.000757839	0.001506443	9.06E-05	0.000837779	0.000778156	0.000598148	0.000972909	0.000710362	2.95E-05
starch degra	0.008776964	0.008395752	0.00721338	0.012545954	0.00612677	0.00688079	0.010095384	0.00737698	0.015440853	0.014700017
CMP-legion	0.000154868	0	0.001235344	3.20E-05	0	0	0	0.000111533	0.001308663	0
hydrogen pr	0	1.52E-05	5.72E-06	1.29E-05	0	0.000274478	0	0	7.81E-05	0
phosphatidy	0.000371579	0.000944513	0.001744405	0.000118286	0.001450504	0.000598955	0.000436585	0.001405891	4.37E-06	1.98E-05
molybdenur	0.00013712	3.56E-05	0	0	0.000118314	4.28E-05	0	0.000420312	0	0
fatty acid be	4.48E-05	0.000588648	0.000600497	2.91E-05	0.000613306	0.000796556	0.000191427	0.000258341	3.21E-06	2.21E-06
all-trans-far	0.000936668	0.000574368	0.001138414	0.00065649	0.000440477	0.000180099	0.000558336	0.001670938	0.002758282	0.00122425
isopropanol	3.45E-05	9.29E-06	0	0	0	0	0	0	0	0
thiazole bios	0.000524995	0.000635089	0.001592909	0.00022032	0.002045968	0.000941791	0.000473383	0.002252511	0	0.000150993
thiazole bios	0.005123225	0.003664645	0.004411652	0.002344848	0.00324685	0.002830558	0.001961563	0.004577731	0.003229523	0.006604131
superpathw	0.002146504	0.002419536	0.003759863	0.001018604	0.004603063	0.003113711	0.00196726	0.003870386	0	0.000708458
thiamin salv	0.005793252	0.005955512	0.004966589	0.007822882	0.005898318	0.00677262	0.00819539	0.00382474	0.005907592	0.006669468
superpathw	0.001983744	0.002125492	0.003599244	0.001831893	0.002906719	0.001250513	0.001852458	0.003463965	0.001824194	0.000498316
seleno-amir	0.00368418	0.002996273	0.005239149	0.002442419	0.003761214	0.004082031	0.002871807	0.00435248	0.006103256	0.004370074
dTDP-3-ace	0	0	0	0	0	0	0	0	0	0
erredoxin ox	0.001176241	0.001298929	0.00275858	0.000685076	0.002791903	0.001656071	0.000800177	0.002209164	0	7.91E-05
glycerol deg	0.000185552	9.08E-05	0.000984159	0	0.000865613	0.000157276	8.35E-05	0.000112921	0	0
L-1,2-propa	0.000617748	0.000814884	0.004210669	0.000342677	0.003422648	0.001241906	0.00160461	4.74E-05	2.71E-05	6.41E-05
phosphatida	0	0	0	0	2.50E-05	0	0	6.39E-05	0	0
4-coumarat	9.38E-05	4.64E-05	0	9.66E-05	0.000184021	0.000297487	5.94E-05	0.000843029	0	4.13E-05
fatty acid sa	0.00027782	0.000903788	0.000694643	0	0.000679544	0.00020208	0.000176767	0.001548844	0	1.50E-05
pyruvate fer	0.009405629	0.01048792	0.00706537	0.013300109	0.007541223	0.009049675	0.011907215	0.009840952	0.013274384	0.012639369
C4 photosy	0.000296224	0.001444932	0.001652867	6.48E-05	0.001805159	0.00060318	0.000410318	0.00183392	0.000105166	1.83E-05
C4 photosy	0.00108751	0.001594862	0.003079982	0.000733097	0.002838691	0.00236203	0.001069404	0.003303374	0.001591432	0.00017959
L-ascorbate	4.11E-05	0.000254406	0.000455697	0	0.000601918	0.000188837	0.00024184	0	0	0
pyrimidine c	0.004072531	0.002874092	0.004149146	0.003297087	0.003970094	0.005121275	0.004998785	0.002925266	0.004952392	0.00208531
pyrimidine c	0.004994746	0.003556983	0.004180974	0.004556305	0.003975156	0.005036287	0.004150056	0.002707195	0.00548384	0.002140354
superpathw	0.00051265	0.000546575	0.000442249	0.000480011	0.000527046	0.000284842	0.000365428	0.000382616	0.000277687	0.000167232
pyrimidine c	0.002860931	0.001907045	0.003038623	0.002155898	0.002872489	0.003767051	0.00421343	0.00175594	0.003372333	0.001067467
pyrimidine c	0.000755214	0.002183553	0.001152973	0.000657131	0.001509347	0.000484531	0.001632146	0.000709604	0.002158357	0.000943081
pyrimidine c	0.008336384	0.008384602	0.005770276	0.009220212	0.006966024	0.00766892	0.008424349	0.005703561	0.007995361	0.010792464
superpathw	0.000129735	0	0.000302791	0	0	0	0	0	0	0
pyridoxal 5'-	0.00021903	0.00018508	0.000222733	0	0.000342819	4.55E-05	0	0.000782783	0	0
superpathw	0.003552956	0.002677438	0.003687421	0.002977684	0.003876825	0.006573116	0.005873029	0.002881856	0.004236774	0.002635837
superpathw	9.70E-06	1.37E-05	3.68E-05	4.32E-05	8.42E-05	5.69E-06	2.82E-05	0.00021868	0	4.07E-05
pyrimidine c	0.000977167	0.002110034	0.001437171	0.000617353	0.001780789	0.000659007	0.000308641	0.000576394	0.000203713	0.00105172
superpathw	0.00163938	0.003358151	0.002255009	0.001083087	0.002733961	0.001123762	0.000542685	0.000965654	0.00400128	0.001904923

adenosine r	0.01066922	0.009924962	0.007571334	0.015014891	0.008920863	0.01199374	0.012583095	0.006148833	0.010012641	0.012542662
adenosine d	0.006997071	0.004609243	0.005022237	0.006944096	0.003387681	0.005775505	0.004608739	0.003973864	0.009185324	0.00526957
guanosine r	0.010780645	0.010592933	0.007143988	0.013709252	0.008413965	0.010021166	0.011468921	0.00713265	0.010697733	0.012779772
guanosine d	0.006997071	0.004609243	0.005022237	0.006944096	0.003387681	0.005775505	0.004608739	0.003973864	0.009185324	0.00526957
superpathw	0.005139025	0.003836966	0.00501945	0.004316662	0.004898383	0.007463819	0.006606283	0.005141436	0.007074063	0.007533041
superpathw	0.008950225	0.006856411	0.006392161	0.008818237	0.006098747	0.007804371	0.007966398	0.005622931	0.008176002	0.008073369
inosine-5'-p	0.007149841	0.004267626	0.005512531	0.006432611	0.004678662	0.005177439	0.005540252	0.002784527	0.000232455	0.000106277
myo-, chiro-	0.003655145	0.003896157	0.003452178	0.002088436	0.004128917	0.00448589	0.008219295	0.002954266	0.009452395	0.004937948
D-fructuron	0.001332385	0.001526436	0.001170894	0.001297272	0.000783483	0.001943589	0.001126091	0.002218082	0.005344455	0.001756429
superpathw	0	0.000186798	0.000387006	0	0.000462871	3.99E-05	0.000302494	0	0	0
superpathw	0.007385887	0.007561886	0.005506004	0.00900058	0.005096501	0.006821596	0.007334475	0.005205617	0.007438703	0.007843674
TCA cycle V	0.000650665	0.000879254	0.002208676	0.000146501	0.002284033	0.001035151	0.000476081	0.001775823	1.97E-05	1.86E-05
NAD/NADP	0	0.000157983	0.000346844	0	0.000425375	3.17E-05	0.000630434	0	0	2.62E-05
NAD/NADP	0	0.00079266	0.001094698	3.24E-05	0.001178924	0.000354599	0.000435034	0	0	1.75E-05
aerobic res	0	3.88E-05	4.70E-05	0	2.35E-05	0.000491178	0	7.53E-06	0	0
4-amino-2-r	0.004932011	0.003785142	0.003750485	0.003577112	0.003553768	0.003430415	0.003260474	0.003816769	0.004280288	0.005912848
fatty acid &b	0.000379646	0	6.89E-06	0	0	0.000701492	7.34E-05	3.99E-05	0	2.01E-05
xylose degr	0.000152547	0.000474991	0.000203682	0	0.000227126	6.51E-05	0	0.000625839	0	0
dTDP-D-&b	0.000538295	0.000260734	0.001000948	4.71E-05	9.89E-05	5.56E-05	0.00033482	0.001105586	0	0.007228467
dTDP-N-ac	0.00015882	0.001182635	0.00174382	0.000105742	0.001802242	0.00042528	0.000570736	0.000297425	0	4.86E-05
dTDP-N-ac	0	0	0.000588588	5.29E-05	0	0	0	0	2.83E-05	5.63E-05
superpathw	0.003046598	0.001956577	0.002136432	0.00209268	0.00131847	0.000512013	0.001333808	0.003174149	0.003849051	0.002059676
superpathw	0.000438341	0.000727122	0.001442587	8.96E-05	0.001526682	0.001390797	0.000747885	0.000960623	0.00092079	2.93E-05
superpathw	0.001247329	0.000960326	0.001906487	0.000971185	0.000498833	0.000518707	0.000987167	0.001347921	0	0.000385368
superpathw	0	0.000274479	0.001125161	0.000211809	0.001316041	0.000389498	0.000116835	0	0	0
thiamin forn	0.004425077	0.005065045	0.00435814	0.006436938	0.005761231	0.007138589	0.007808572	0.003520296	0.007995186	0.005798798
1,4-dihydro	0.001215116	0.000301125	0.000345519	0.000309615	0.000135459	3.08E-05	0.001595123	8.58E-06	0	0
1,4-dihydro	0	0	0	0	0	0	0	0	0	0
anaerobic e	0.003497414	0.001673247	0.001568375	0.002536088	0.002351498	0.000353977	0.001657324	0.000414347	6.29E-05	2.72E-05
1,3-propane	0.000569748	1.19E-05	0	0	0.000263737	0.000106684	0	7.02E-05	0	3.90E-05
octanoyl-[ac	0.002549849	0.003641189	0.004649859	0.001699862	0.004098868	0.002745823	0.002484881	0.006483285	0.00212717	0.001624791
superpathw	0	0.000131577	0	0	0	0	0	0	0	0
taxadiene b	0.000183539	0	5.88E-05	0.000260832	2.94E-05	0	0.000279266	0.000384106	0.001664179	0.00011696
L-arginine b	0.004516359	0.005837394	0.004554936	0.007577645	0.005784472	0.008317181	0.008491615	0.003690799	0.010331707	0.003318409
phospholipi	0	3.97E-05	0	0	0.000104953	3.79E-05	0	0	0	0
sulfoglycoly	5.06E-05	0.000575288	0.000238546	0	0.000300031	9.81E-05	0	0.000308976	0	0
mannan deg	0.002591476	0.002151089	0.001687864	0.002053238	0.001007252	0.00029543	0.000997332	0.001870215	0.000941678	0.001517612
6-hydroxym	0.006319188	0.005735755	0.004592877	0.00685032	0.005499031	0.006029337	0.008093142	0.003708503	0.006018247	0.005718513
methyleryth	0.000254476	0	0.000132675	0.000221187	6.78E-05	0	0.000240073	0.000744137	0.001460384	9.23E-05
methanol ox	0	2.95E-05	7.25E-05	2.24E-05	0.000132222	3.50E-05	0	0	0	0
gondoate bi	0.007991857	0.007606909	0.00509549	0.008215857	0.005180318	0.007062126	0.005819576	0.006383388	0.005863568	0.010518291
oleate biosy	0.00331554	0.004417586	0.004731757	0.002311051	0.004485251	0.003559105	0.003154921	0.005537681	0.002350835	0.001786226
superpathw	0.000628246	0.001282568	0.001826153	0.000286559	0.001515087	0.001313628	0.000562169	0.001200341	6.04E-05	0
superpathw	0.006513919	0.004735444	0.005601014	0.005719346	0.005184504	0.007249771	0.007344672	0.005255354	0.007377672	0.007318539
superpathw	0.00032406	0.002032927	0.001896669	6.66E-05	0.002241552	0.000668469	0.000471646	0.002109674	0.000106876	1.89E-05
ADP-L-glyc	0.00180067	0.001007305	0.001011778	0.000424625	0.001080681	0.001086218	0.001209872	0.001217596	0.000598745	0.001233149
anhydromu	0.00272109	0.003205005	0.002897457	0.002308442	0.00335209	0.004217628	0.002593323	0.003050964	0.001531086	0.000752208
3-phenylpro	6.61E-05	0.000397454	0.000994808	3.55E-05	0.001070613	0.000329356	0.000209102	0.001633854	0	0
purine ribon	0.002204343	0.004006512	0.003060553	0.004006171	0.004300801	0.005674877	0.006229662	0.004330466	0.009435521	0.002835804
superpathw	0.000788949	0.001511374	0.00231291	7.93E-05	0.002471976	0.002018105	0.000963569	0.004053573	0.000565934	0.000174895
superpathw	0.000556947	0.001307207	0.001893252	0.000181755	0.002205151	0.001807406	0.000685257	0.002696815	6.77E-05	4.00E-05
CDP-diacylg	0.008190485	0.008189126	0.005685547	0.011164345	0.005622048	0.005767542	0.00820359	0.006217418	0.009576503	0.010670405
polymyxin r	7.97E-06	0.000870894	0.000868912	6.00E-05	0.000763776	0.000243345	0.000264195	0	0	0
superpathw	0.000356441	0.000611311	0.000740627	9.13E-05	0.000927604	0.000950555	0.000286979	0.001203892	0	3.20E-05
tRNA proce	0.001075868	0.002313519	0.00269045	0.000504877	0.002942444	0.004356908	0.000708804	0.002797354	0.000909886	4.69E-05
methylphos	6.07E-05	3.89E-06	0	0	2.68E-05	1.52E-05	0	2.39E-05	0	2.89E-06
peptidoglyc	0.006141822	0.007755451	0.007278442	0.005661857	0.008993548	0.011607895	0.007511561	0.009557718	0.007145054	0.013362625
superpathw	0.006033105	0.004659184	0.005378156	0.005697824	0.005169823	0.005499468	0.007639218	0.003091255	0.003114598	0.003503854
superpathw	0.005243264	0.00374335	0.004479551	0.004735147	0.004431857	0.005567204	0.005275218	0.003022437	0.005546813	0.002261037
phenylaceta	0	0	0	0	4.85E-05	0	0	0	0	0
allantoin de	3.55E-05	7.79E-06	0	0	4.77E-05	2.05E-05	0	0.00021516	0	0
2-methylcitr	0.000171498	8.68E-05	3.24E-05	1.49E-05	0.000309197	0.00012545	0	0.000960013	0	1.10E-05
aspartate su	0.000887094	0.001916466	0.002654353	0.000796886	0.002783168	0.001896368	0.001156375	0.002326741	0.000203543	0.000140508
superpathw	0.004765883	0.002643997	0.003642785	0.002223898	0.002529509	0.002007913	0.001844694	0.004216601	0.00315621	0.007444881
(5Z)-dodec-	0.003012342	0.004122587	0.004584711	0.002068093	0.004310747	0.003250758	0.002948878	0.005335154	0.002134951	0.001569633
superpathw	0	0.000388796	0.002079387	0.000167925	0.00186224	0.000919554	0.000417996	0	0	0
L-ascorbate	4.14E-05	0.000262043	0.001237954	6.07E-05	0.001293741	0.000621725	0.000546033	0	0	0
stearate bio	0.000274022	0.000819431	0.000612781	5.05E-05	0.000474975	0.000128899	0.000133511	0.000830219	0	0
nitrate redu	0	2.72E-05	7.90E-06	1.08E-05	1.69E-05	6.05E-05	1.37E-05	1.41E-05	2.65E-05	0
phosphatidy	0.001649454	0.002584272	0.002820296	0.002123787	0.00348694	0.004424473	0.001579269	0.004144541	0.003233878	0.001553846
phosphatidy	0.001649454	0.002584272	0.002820296	0.002123787	0.00348694	0.004424473	0.001579269	0.004144541	0.003233878	0.001553846
superpathw	0.000656984	0.000860694	0.001620112	6.53E-05	0.001652748	0.000512267	0.000567882	0.00271743	5.21E-05	3.26E-05
biphenyl de	0	2.05E-05	5.74E-05	0	0.000106708	1.96E-05	0	0	0	0
ketogenesis	3.20E-05	9.99E-06	1.92E-05	0	1.14E-05	2.40E-05	2.81E-05	0	0	0
fatty acid &a	4.25E-05	0.000150702	0	0	4.28E-05	5.74E-06	0	0	0	9.67E-06
phytol degra	4.31E-05	0.000705336	0.001383111	0.000124607	0.001310203	0.001031837	0.000311088	3.09E-05	0	9.69E-06
fatty acid &b	0.000621676	0	1.37E-05	0	0	0.001061957	0.000133814	7.90E-05	0	3.33E-05
TCA cycle II	0.000179172	0.000153328	0.000113913	0.000101262	0.000627943	0.001018426	0.00054202	0.000783674	0.000160103	4.67E-05
gluconeoge	0.002022336	0.001626137	0.002222897	0.002126848	0.002415384	0.000284705	0.001566269	0.000623758	0.000144048	7.46E-05
glycolysis V	0.002027123	0.002455517	0.005309386	0.002459094	0.00472491	0.002138266	0.001923506	0.003893232	0.001577304	0.000341367
superpathw	0.000891824	0.003033295	0.003106148	0.000126338	0.003041066	0.004262404	0.001176311	0.002437957	0.001481273	0.00051311
D-galactose	0.002329154	0.002722743	0.002891538	0.002989074	0.003229304	0.003566548	0.00347904	0.002952571	0.005447495	0.001962179
mycolate bi	0.003427076	0.004427661	0.004986544	0.002349856	0.004548462	0.003489302	0.003172954	0.00585603	0.002450932	0.001837918
NAD salvag	0.000951192	0.00205949	0.002155653	0.000769699	0.001954587	0.00205605	0.00060278	0.002380172	6.64E-05	0.000166227
NAD biosyn	0.009567827	0.006695982	0.005259173	0.008277364	0.005407659	0.00501794	0.007429409	0.004929549	0.006073345	0.007031752
pyridoxal 5'	0.005589104	0.002381747	0.003795619	0.00202132	0.002283496	0.001711677	0.001564073	0.004005697	0.002722464	0.011140411
TCA cycle V	0.000236022	0.000502996	0.001075358	0.00010701	0.001113426	0.000564911	0.000322115	2.62E-05	0	0
L-rhamnose	0.002380661	0.002365088	0.002666109	0.002289776	0.00313391	0.002422231	0.001643942	0.00302761	0.003226069	0.002351201
flavin biosyn	0.006885337	0.007617749	0.004925121	0.009829533	0.006552161	0.008771834	0.010950291	0.005132515	0.008661121	0.008619718
adenosine r	0.001157017	0.000528687	0.000335776	0.000354056	0.000668088	0.00149218	0.000314927	0.002845743	0.002590474	0.000941752

superpathw	0.005895733	0.004779634	0.00514884	0.004678325	0.004162399	0.002936668	0.006728894	0.00532796	0.004212781	0.01025061
sulfate redu	0.000947927	0.002655205	0.003242544	0.000393802	0.002410922	0.002118567	0.00118306	0.003856245	7.32E-05	9.95E-05
superpathw	0.001765458	0.003482854	0.004053089	0.000796168	0.003038215	0.002238909	0.002047531	0.004062587	0.000158378	0.000208907
superpathw	0.000797124	0.000946586	0.002039411	9.04E-05	0.00220269	0.001140142	0.000623817	0.002176344	1.29E-05	3.44E-05
TCA cycle I	0.001965752	0.001876371	0.003434252	0.000978982	0.003163303	0.001963304	0.00141519	0.003612788	0.001518264	0.000145463
teichoic acid	0.000732326	0.000444615	0.000362863	0.000337663	0.000118459	0.000292545	0.000378419	0.001039285	0.001949523	0.000240893
superpathw	0.008067398	0.006537583	0.005383718	0.006279073	0.005522068	0.005572388	0.005325102	0.004634507	0.00504656	0.008530174
superpathw	0.004652408	0.005504001	0.003942044	0.006952265	0.004208988	0.003972951	0.003821315	0.004673879	0.006334019	0.011382496
superpathw	2.52E-05	0.000348658	0.001353385	6.44E-05	0.001199725	0.000519155	0.000255849	0	0	0
superpathw	0.00710615	0.00662439	0.006219175	0.00743227	0.004467487	0.005236667	0.006181302	0.006606277	0.007706601	0.011530853
tRNA charg	0.010150972	0.009379002	0.007152055	0.013388838	0.007963248	0.009564889	0.010881251	0.006234386	0.009720252	0.010878131
L-tryptophan	0.002410339	0.005890409	0.003511457	0.003550572	0.003244731	0.00326065	0.005928943	0.005682597	0.004522291	0.003199801
L-tyrosine d	0	1.06E-05	1.95E-05	0	0	0.00025565	0	0	0	0
superpathw	3.39E-05	0.000408994	0.000650569	2.10E-05	0.000614221	0.000565433	0.000204215	0.000178736	0	0
UDP-N-ace	0.001583345	0.00210704	0.001922281	0.002369542	0.003028361	0.004647415	0.00620498	0.001963004	0.002388568	0.00031277
superpathw	2.44E-05	5.35E-06	0	0	1.86E-05	1.46E-05	0	0.000144546	0	0
ureide biosy	0	2.88E-05	0.000259133	0	0.000421122	0.000120207	9.98E-05	0	0	0
L-valine bio	0.009604616	0.01048792	0.00706537	0.013300109	0.007541223	0.009049675	0.011907215	0.009840952	0.013274384	0.012639369

Table S9. For each bin, the number of bases, N50, completeness, contamination and taxonomy are reported

genome	completen	contamina	bases	N50	completen	contamination_metric	taxonomy
mixed_bin.107.fa	91.88	2.82	2803257	12506	near	low	Bacteria;Actinobacteria;Coriobacteriia;Eggerthellales;Eggerthellaceae;Eggerthella;Eggerthella lenta
mixed_bin.113.fa	75.21	4.91	4431681	4513	substantial	low	Bacteria;Firmicutes;Clostridia;Eubacteriales;Lachnospiraceae
mixed_bin.18.fa	82.08	3.12	2132952	7832	substantial	low	Bacteria;Firmicutes;Clostridia;Eubacteriales;Lachnospiraceae
mixed_bin.19.fa	79.43	8.72	1597306	3900	substantial	medium	Bacteria;Firmicutes;Clostridia;Eubacteriales;Oscillospiraceae
mixed_bin.30.fa	87.66	8.45	5268918	6918	substantial	medium	Bacteria;Firmicutes;Clostridia;Eubacteriales;Lachnospiraceae;Enterocloster;Enterocloster bolteae
mixed_bin.42.fa	86.42	1.79	2078858	9899	substantial	low	Bacteria
mixed_bin.46.fa	82.35	0.81	1964851	9524	substantial	low	Bacteria;Actinobacteria;Coriobacteriia;Eggerthellales;Eggerthellaceae
mixed_bin.52.fa	83.33	2.75	2091839	10397	substantial	low	Bacteria;Firmicutes;Clostridia;Eubacteriales;Lachnospiraceae
mixed_bin.56.fa	93.21	2.02	1675603	7078	near	low	Bacteria
mixed_bin.61.fa	89.26	3.15	2046527	7747	substantial	low	Bacteria;Firmicutes
mixed_bin.66.fa	86.96	2.15	2070865	8989	substantial	low	Bacteria;Firmicutes;Clostridia;Eubacteriales;Lachnospiraceae
mixed_bin.67.fa	96.55	2.19	2093280	55368	near	low	Bacteria;Firmicutes;Clostridia;Eubacteriales;Lachnospiraceae
mixed_bin.76.fa	81.66	2.89	2853765	4928	substantial	low	Bacteria;Proteobacteria;Deltaproteobacteria;Desulfovibrionales;Desulfovibrionaceae
mixed_bin.79.fa	74.01	4.39	2062082	6618	substantial	low	Bacteria;Firmicutes;Clostridia;Eubacteriales;Lachnospiraceae
mixed_bin.87.fa	81.92	0.67	1914929	15254	substantial	low	Bacteria;Firmicutes;Clostridia;Eubacteriales
mixed_bin.88.fa	92.24	2.4	2577733	7684	near	low	Bacteria;Firmicutes;Clostridia;Eubacteriales;Oscillospiraceae;[Eubacterium] siraeum
mixed_bin.89.fa	83.05	2.85	1912161	4520	substantial	low	Bacteria;Firmicutes;Clostridia;Eubacteriales;Lachnospiraceae;Anaerostipes;Anaerostipes hadrus
mixed_bin.9.fa	79.2	1.27	2042213	6057	substantial	low	Bacteria;Firmicutes;Clostridia;Eubacteriales;Lachnospiraceae
mixed_bin.98.fa	84.78	1.68	1809963	17770	substantial	low	Bacteria;Firmicutes;Clostridia;Eubacteriales
W12_bin.1.fa	98.66	0	2509503	167729	near	none	Bacteria;Firmicutes;Clostridia;Eubacteriales;Oscillospiraceae
W12_bin.13.fa	88.56	0	2179161	25557	substantial	none	Bacteria;Firmicutes;Clostridia;Eubacteriales
W12_bin.18.fa	97.31	0.1	1929994	51012	near	low	Bacteria;Proteobacteria;Alphaproteobacteria
W12_bin.5.fa	99.25	0	3387086	42720	near	none	Bacteria;Bacteroidetes;Bacteroidia;Bacteroidales
W12_bin.6.fa	95.05	0	2009167	137838	near	none	Bacteria
W13_bin.13.fa	94.35	1.78	2501512	9162	near	low	Bacteria;Proteobacteria;Deltaproteobacteria;Desulfovibrionales;Desulfovibrionaceae
W13_bin.15.fa	86.1	2.96	1828973	5870	substantial	low	Bacteria;Firmicutes;Negativicutes;Veillonellales;Veillonellaceae;Megasphaera;Megasphaera stantonii
W13_bin.17.fa	95.42	1.16	2052152	12132	near	low	Bacteria;Proteobacteria;Betaproteobacteria;Burkholderiales;Oxalobacteraceae;Oxalobacter;Oxalobacter formigenes
W13_bin.19.fa	98.31	0	2639529	114759	near	none	Bacteria;Bacteroidetes;Bacteroidia;Bacteroidales;Rikenellaceae
W13_bin.20.fa	90.56	0	1523934	20874	near	none	Bacteria;Proteobacteria;Betaproteobacteria;Burkholderiales
W13_bin.21.fa	75.17	0	4688220	11095	substantial	none	Bacteria;Bacteroidetes;Bacteroidia;Bacteroidales;Bacteroidaceae;Bacteroides;Bacteroides thetaiotaomicron
W13_bin.24.fa	96.56	0	1817070	17188	near	none	Bacteria;Actinobacteria;Coriobacteriia;Coriobacteriales
W13_bin.25.fa	97	0	2331014	12931	near	none	Bacteria;Synergistetes;Synergistia;Synergistales;Synergistaceae
W13_bin.7.fa	70.52	1.97	1663159	8783	substantial	low	Bacteria
W16_bin.14.fa	97.01	0.19	3489085	83602	near	low	Bacteria;Bacteroidetes;Bacteroidia;Bacteroidales
W16_bin.15.fa	97.52	0.16	2682030	105397	near	low	Bacteria;Proteobacteria;Betaproteobacteria;Burkholderiales;Sutterellaceae
W16_bin.20.fa	97.92	0.53	3192387	58910	near	low	Bacteria;Bacteroidetes;Bacteroidia;Bacteroidales
W16_bin.3.fa	83.18	0.38	1893303	3668	substantial	low	Bacteria;Bacteroidetes;Bacteroidia;Bacteroidales;Muribaculaceae
W25_bin.1.fa	100	0	2557008	236648	perfect	none	Bacteria;Bacteroidetes;Bacteroidia;Bacteroidales;Rikenellaceae
W25_bin.11.fa	95.16	1.28	1916432	21448	near	low	Bacteria;Actinobacteria;Coriobacteriia
W25_bin.12.fa	95.47	0.92	2110025	22950	near	low	Bacteria;Firmicutes;Clostridia;Eubacteriales
W25_bin.16.fa	78.72	3.14	2269120	3015	substantial	low	Bacteria
W25_bin.17.fa	82	0	952191	14661	substantial	none	Bacteria
W25_bin.18.fa	90.45	2.25	1191529	46958	near	low	Bacteria
W25_bin.19.fa	95.7	0	1691787	55093	near	none	Bacteria;Proteobacteria
W25_bin.20.fa	92.42	2.25	1344035	64521	near	low	Bacteria
W25_bin.21.fa	76.55	0.1	2356659	3556	substantial	low	Bacteria;Proteobacteria;Deltaproteobacteria;Desulfovibrionales;Desulfovibrionaceae;Desulfovibrio;Desulfovibrio fairfieldensis
W25_bin.22.fa	97.58	0	2063209	21108	near	none	Bacteria
W25_bin.24.fa	89.93	0	1881564	116801	substantial	none	Bacteria;Firmicutes;Clostridia;Eubacteriales
W25_bin.27.fa	99.19	0	2223544	122533	near	none	Bacteria;Actinobacteria;Coriobacteriia;Eggerthellales;Eggerthellaceae
W25_bin.3.fa	71.41	3.85	1172295	34234	substantial	low	Bacteria;Firmicutes;Clostridia;Eubacteriales
W25_bin.36.fa	99.06	0.01	3100371	42879	near	low	Bacteria;Bacteroidetes;Bacteroidia;Bacteroidales
W25_bin.38.fa	98.25	3.86	1284046	37809	near	low	Bacteria;Firmicutes;Clostridia;Eubacteriales
W25_bin.40.fa	100	0	2287829	185109	perfect	none	Bacteria;Actinobacteria;Actinomycetia;Bifidobacteriales;Bifidobacteriaceae;Bifidobacterium;Bifidobacterium longum
W25_bin.41.fa	97.96	0	2979082	103104	near	none	Bacteria;Verrucomicrobia;Verrucomicrobiae;Verrucomicrobiales;Akkermansiaceae
W25_bin.43.fa	95.75	1.01	3637209	36936	near	low	Bacteria;Firmicutes;Clostridia;Eubacteriales
W25_bin.47.fa	94.73	2.42	1478981	10103	near	low	Bacteria
W25_bin.48.fa	71.87	8.99	2195865	5582	substantial	medium	Bacteria;Firmicutes;Clostridia;Eubacteriales;Oscillospiraceae
W25_bin.5.fa	81.55	7.36	5266168	10543	substantial	medium	Bacteria;Firmicutes;Clostridia;Eubacteriales
W25_bin.6.fa	88.26	0	1690899	17356	substantial	none	Bacteria;Firmicutes;Clostridia;Eubacteriales
W25_bin.9.fa	71.95	0.67	1665364	4245	substantial	low	Bacteria
W26_bin.1.fa	73.1	0	1782249	11809	substantial	none	Bacteria
W26_bin.10.fa	82.21	0.79	2243544	28851	substantial	low	Bacteria;Proteobacteria;Alphaproteobacteria
W26_bin.11.fa	89.93	0.84	1989132	54753	substantial	low	Bacteria;Firmicutes;Clostridia;Eubacteriales
W26_bin.13.fa	86.85	0	1787244	37398	substantial	none	Bacteria
W26_bin.14.fa	88.62	4.05	2062797	11313	substantial	low	Bacteria;Firmicutes;Clostridia;Eubacteriales
W26_bin.17.fa	77.82	0.2	1924489	4171	substantial	low	Bacteria
W26_bin.18.fa	98.39	0	2598440	84872	near	none	Bacteria
W26_bin.20.fa	98.55	1.55	3071775	111488	near	low	Bacteria;Firmicutes;Clostridia;Eubacteriales;Lachnospiraceae
W26_bin.21.fa	95.91	0	2633878	28874	near	none	Bacteria;Bacteroidetes;Bacteroidia;Bacteroidales;Rikenellaceae
W26_bin.22.fa	87.25	0.67	1764066	87504	substantial	low	Bacteria;Firmicutes;Clostridia;Eubacteriales
W26_bin.23.fa	89.85	0	1722678	10979	substantial	none	Bacteria
W26_bin.27.fa	99.32	0.68	3359309	56062	near	low	Bacteria
W26_bin.29.fa	96.48	0.82	4710245	22279	near	low	Bacteria;Proteobacteria;Gammaproteobacteria;Enterobacterales;Enterobacteriaceae
W26_bin.32.fa	84.51	0	1853054	14451	substantial	none	Bacteria
W26_bin.36.fa	84.41	1.61	1334400	12991	substantial	low	Bacteria
W26_bin.37.fa	91.09	1.61	1892437	10708	near	low	Bacteria;Actinobacteria;Coriobacteriia;Eggerthellales;Eggerthellaceae
W26_bin.42.fa	87.7	0	1951291	66763	substantial	none	Bacteria;Firmicutes;Clostridia;Eubacteriales
W26_bin.6.fa	74.11	1.98	1784539	3567	substantial	low	Bacteria
W26_bin.7.fa	99.3	1.17	2861391	40750	near	low	Bacteria
W26_bin.8.fa	97.32	0	1899102	332176	near	none	Bacteria;Firmicutes;Clostridia;Eubacteriales
W27_bin.2.fa	95.85	1.14	5013905	100793	near	low	Bacteria;Proteobacteria;Gammaproteobacteria;Enterobacterales;Enterobacteriaceae;Klebsiella
W27_bin.6.fa	83.82	0.31	2256835	5580	substantial	low	Bacteria;Proteobacteria;Betaproteobacteria;Burkholderiales;Burkholderiales bacterium YL45
W28_bin.1.fa	97.99	1.34	2666237	132503	near	low	Bacteria;Firmicutes;Clostridia;Eubacteriales;Oscillospiraceae
W28_bin.12.fa	90.25	0.67	2136265	14425	near	low	Bacteria
W28_bin.14.fa	92.26	1.34	2019802	77483	near	low	Bacteria
W28_bin.17.fa	92.99	1.01	1946714	36458	near	low	Bacteria;Firmicutes;Clostridia;Eubacteriales
W28_bin.2.fa	82.26	9.82	2294225	35420	substantial	medium	Bacteria;Firmicutes;Clostridia;Eubacteriales;Oscillospiraceae;Faecalibacterium;Faecalibacterium prausnitzii
W28_bin.20.fa	99.33	0.47	2065481	57572	near	low	Bacteria;Firmicutes;Clostridia;Eubacteriales;Oscillospiraceae
W28_bin.23.fa	90.6	0	2297139	70209	near	none	Bacteria;Firmicutes;Clostridia;Eubacteriales
W28_bin.24.fa	99.04	0.96	2081634	102318	near	low	Bacteria;Bacteroidetes;Bacteroidia;Bacteroidales;Rikenellaceae
W28_bin.25.fa	98.19	0.96	3577116	46303	near	low	Bacteria;Bacteroidetes;Bacteroidia;Bacteroidales
W28_bin.27.fa	97.32	0	2250774	82714	near	none	Bacteria;Firmicutes;Clostridia;Eubacteriales;Oscillospiraceae
W28_bin.7.fa	93.29	0.67	1985854	127037	near	low	Bacteria;Firmicutes;Clostridia;Eubacteriales;Oscillospiraceae
W28_bin.8.fa	100	1.61	2134697	71708	perfect	low	Bacteria;Actinobacteria;Coriobacteriia;Coriobacteriales;Coriobacteriaceae;Collinsella;Collinsella aerofaciens
W29_bin.19.fa	96.89	2.02	3078012	50353	near	low	Bacteria;Bacteroidetes;Bacteroidia;Bacteroidales;Rikenellaceae
W29_bin.21.fa	99.91	0	2374519	29542	near	none	Bacteria;Firmicutes;Negativicutes;Veillonellales;Veillonellaceae
W29_bin.3.fa	95.03	0.68	2431693	30575	near	low	Bacteria;Firmicutes;Clostridia;Eubacteriales;Oscillospiraceae
W29_bin.4.fa	95.95	0.59	3897701	54465	near	low	Bacteria;Bacteroidetes;Bacteroidia;Bacteroidales
W29_bin.6.fa	94.7	1.23	4206042	31076	near	low	Bacteria;Proteobacteria;Gammaproteobacteria;Enterobacterales;Enterobacteriaceae;Enterobacter
W29_bin.7.fa	100	0	2082862	46617	perfect	none	Bacteria;Firmicutes;Negativicutes;Acidaminococcales;Acidaminococcaceae;Acidaminococcus;Acidaminococcus intestini
W3_bin.3.fa	76.07	3.57	2167313	20320	substantial	low	Bacteria;Firmicutes;Clostridia;Eubacteriales;Oscillospiraceae;Faecalibacterium;Faecalibacterium prausnitzii
W30_bin.12.fa	100	0	2582582	56400	perfect	none	Bacteria
W30_bin.14.fa	98.39	1.61	2095288	23216	near	low	Bacteria;Actinobacteria;Coriobacteriia;Coriobacteriales;Coriobacteriaceae;Collinsella;Collinsella aerofaciens
W30_bin.15.fa	98.5	0	4334796	80032	near	none	Bacteria;Bacteroidetes;Bacteroidia;Bacteroidales;Phocaeicola;Phocaeicola vulgatus
W30_bin.16.fa	97.99	0.34	2157386	67908	near	low	Bacteria;Firmicutes;Clostridia;Eubacteriales;Oscillospiraceae
W30_bin.20.fa	98.63	0	2854195	83162	near	none	Bacteria;Firmicutes;Clostridia;Eubacteriales;Oscillospiraceae
W30_bin.3.fa	98.82	0	3996784	32635	near	none	Bacteria;Proteobacteria;Deltaproteobacteria;Desulfovibrionales;Desulfovibrionaceae
W30_bin.5.fa	99.25	0.75	5587450	135026	near	low	Bacteria;Bacteroidetes;Bacteroidia;Bacteroidales;Bacteroidaceae
W30_bin.6.fa	93.35	0.98	2490902	38785	near	low	Bacteria;Proteobacteria;Deltaproteobacteria;Desulfovibrionales;Desulfovibrionaceae
W31_bin.14.fa	81.77	2.87	1450034	3294	substantial	low	Bacteria;Proteobacteria
W31_bin.15.fa	98.12	0	2350405	65978	near	none	Bacteria;Proteobacteria;Betaproteobacteria;Burkholderiales;Sutterellaceae
W31_bin.20.fa	98.66	1.01	1986944	38210	near	low	Bacteria
W31_bin.21.fa	98.02	0.42	3221490	52249	near	low	Bacteria;Bacteroidetes;Bacteroidia;Bacteroidales
W31_bin.23.fa	95.66	1.51	2534049	99183	near	low	Bacteria
W31_bin.28.fa	100	1.61	2456304	31043	perfect	low	Bacteria;Actinobacteria;Coriobacteriia;Coriobacteriales;Coriobacteriaceae;Collinsella;Collinsella aerofaciens
W31_bin.29.fa	97.99	0	2851311	76140	near	none	Bacteria;Firmicutes;Clostridia;Eubacteriales
W31_bin.30.fa	95.02	1.28	2774272	83166	near	low	Bacteria;Firmicutes;Clostridia;Eubacteriales;Lachnospiraceae
W31_bin.4.fa	98.87	0.6	3492800	39771	near	low	Bacteria;Firmicutes;Clostridia;Eubacteriales;Lachnospiraceae

W31_bin.5.fa	100	0.11	2665617	81704	perfect	low	Bacteria;Proteobacteria;Betaproteobacteria;Burkholderiales;Burkholderiales bacterium YL45
W32_bin.12.fa	96.73	0	2258971	42728	near	none	Bacteria
W32_bin.14.fa	95.37	3.02	2241623	14669	near	low	Bacteria;Firmicutes;Clostridia;Eubacteriales;Clostridiales bacterium CCNA10
W32_bin.15.fa	89.25	0	1680264	13237	substantial	none	Bacteria;Proteobacteria;Alphaproteobacteria
W32_bin.16.fa	93.33	1.35	3042987	54093	near	low	Bacteria
W32_bin.17.fa	84.79	0	1763605	65407	substantial	none	Bacteria
W32_bin.2.fa	94.06	0.63	4082652	62870	near	low	Bacteria;Bacteroidetes;Bacteroidia;Bacteroidales
W32_bin.20.fa	90.6	0	2316106	114588	near	none	Bacteria;Firmicutes;Clostridia;Eubacteriales
W32_bin.22.fa	94.78	0	1601911	64666	near	none	Bacteria
W32_bin.25.fa	72.81	0.78	1663915	5409	substantial	low	Bacteria
W32_bin.29.fa	97.09	0	2354800	80824	near	none	Bacteria;Firmicutes;Clostridia;Eubacteriales
W32_bin.3.fa	100	0.9	2217147	99656	perfect	low	Bacteria
W32_bin.31.fa	96.6	0	2161173	104077	near	none	Bacteria
W32_bin.35.fa	76.32	1.77	3363990	47292	substantial	low	Bacteria
W32_bin.37.fa	80.89	1.55	1708045	5683	substantial	low	Bacteria;Firmicutes;Clostridia;Eubacteriales
W32_bin.4.fa	89.89	2.59	2184407	9184	substantial	low	Bacteria;Proteobacteria;Betaproteobacteria;Burkholderiales;Sutterellaceae
W32_bin.8.fa	97.58	2.69	3042793	47136	near	low	Bacteria
W32_bin.9.fa	90.03	2.19	5872321	28462	near	low	Bacteria;Bacteroidetes;Bacteroidia;Bacteroidales;Bacteroidaceae;Bacteroides;Bacteroides cellulosilyticus
W33_bin.12.fa	98.05	0.56	3452809	98276	near	low	Bacteria;Bacteroidetes;Bacteroidia;Bacteroidales
W33_bin.16.fa	93.69	0	2884444	51917	near	none	Bacteria;Bacteroidetes;Bacteroidia;Bacteroidales
W33_bin.17.fa	93.62	0	2575013	19178	near	none	Bacteria;Firmicutes;Clostridia;Eubacteriales
W33_bin.18.fa	95.04	0.4	2010285	12901	near	low	Bacteria
W33_bin.2.fa	96.31	0.67	2151450	157452	near	low	Bacteria;Firmicutes;Clostridia;Eubacteriales
W33_bin.28.fa	100	0	2566467	60632	perfect	none	Bacteria;Bacteroidetes;Bacteroidia;Bacteroidales;Rikenellaceae;Alistipes;Alistipes communis
W33_bin.8.fa	85.91	0	1585516	19583	substantial	none	Bacteria;Firmicutes;Clostridia;Eubacteriales
W34_bin.10.fa	93.29	0	2103610	313916	near	none	Bacteria;Firmicutes;Clostridia;Eubacteriales
W34_bin.11.fa	88.23	6.32	1679130	4136	substantial	medium	Bacteria;Proteobacteria;Gammaproteobacteria;Pasteurellales;Pasteurellaceae;Haemophilus;Haemophilus parainfluenzae
W34_bin.13.fa	91.01	0.22	2303106	24837	near	low	Bacteria;Firmicutes;Clostridia;Eubacteriales;Oscillospiraceae
W34_bin.18.fa	99.33	0.15	3131833	73432	near	low	Bacteria;Firmicutes;Clostridia;Eubacteriales;Lachnospiraceae
W34_bin.2.fa	97.67	0.36	3702613	70799	near	low	Bacteria;Firmicutes;Clostridia;Eubacteriales;Lachnospiraceae
W34_bin.8.fa	95.24	0	2448398	91189	near	none	Bacteria
W40_bin.1.fa	97.09	0.21	2628211	32739	near	low	Bacteria;Firmicutes;Clostridia;Eubacteriales
W40_bin.17.fa	99.42	0.19	4804713	110840	near	low	Bacteria;Bacteroidetes;Bacteroidia;Bacteroidales;Tannerellaceae
W40_bin.18.fa	98.72	0.24	3088194	87328	near	low	Bacteria;Firmicutes;Clostridia;Eubacteriales;Lachnospiraceae
W40_bin.19.fa	87.24	0.84	2187686	6212	substantial	low	Bacteria;Firmicutes;Clostridia;Eubacteriales;Oscillospiraceae
W40_bin.20.fa	98.39	0	3758524	132715	near	none	Bacteria;Bacteroidetes;Bacteroidia;Bacteroidales
W40_bin.21.fa	99.14	0	2997146	167780	near	none	Bacteria;Bacteroidetes;Bacteroidia;Bacteroidales;Rikenellaceae
W40_bin.7.fa	98.75	0	2259760	82274	near	none	Bacteria;Proteobacteria;Betaproteobacteria;Burkholderiales;Sutterellaceae
W40_bin.8.fa	99.19	2.2	3791472	26941	near	low	Bacteria;Bacteroidetes;Bacteroidia;Bacteroidales;Odoribacteraceae;Butyricimonas;Butyricimonas faecalis
W41_bin.1.fa	99.33	0.13	2960829	44387	near	low	Bacteria;Firmicutes;Clostridia;Eubacteriales;Oscillospiraceae;Flavonifractor;Flavonifractor plautii
W41_bin.10.fa	84.49	1.55	4309922	31877	substantial	low	Bacteria;Bacteroidetes;Bacteroidia;Bacteroidales;Bacteroidaceae;Bacteroides
W41_bin.2.fa	90.34	4.8	6209313	29721	near	low	Bacteria;Bacteroidetes;Bacteroidia;Bacteroidales;Bacteroidaceae;Bacteroides
W41_bin.5.fa	99.98	1.5	2311248	316590	near	low	Bacteria;Firmicutes;Negativicutes;Acidaminococcales;Acidaminococcaceae
W41_bin.6.fa	100	0	2652693	110100	perfect	none	Bacteria;Firmicutes;Negativicutes;Veillonellales;Veillonellaceae
W41_bin.8.fa	100	0	2239134	52442	perfect	none	Bacteria;Proteobacteria;Betaproteobacteria;Burkholderiales;Burkholderiales bacterium YL45
W41_bin.9.fa	97.12	0.09	3456339	104961	near	low	Bacteria;Bacteroidetes;Bacteroidia;Bacteroidales;Bacteroidaceae
W47_bin.1.fa	93.72	0	2473261	4995	near	none	Bacteria;Proteobacteria;Betaproteobacteria;Burkholderiales;Sutterellaceae
W47_bin.11.fa	100	0.63	2178163	61098	perfect	low	Bacteria;Firmicutes;Negativicutes;Selenomonadales;Selenomonadaceae
W47_bin.12.fa	98.73	0.65	3645527	78946	near	low	Bacteria;Firmicutes;Clostridia;Eubacteriales
W47_bin.3.fa	96.63	1.12	2550850	8984	near	low	Bacteria;Fusobacteria;Fusobacteriia;Fusobacteriales;Fusobacteriaceae
W47_bin.4.fa	98.3	0	2225662	66064	near	none	Bacteria;Firmicutes;Negativicutes;Veillonellales;Veillonellaceae;Megasphaera;Megasphaera elsdenii
W47_bin.8.fa	82.9	1.43	3490695	11542	substantial	low	Bacteria;Bacteroidetes;Bacteroidia;Bacteroidales;Bacteroidaceae;Bacteroides;Bacteroides caccae
W49_bin.12.fa	81.74	2.11	2280183	5234	substantial	low	Bacteria;Firmicutes;Clostridia;Eubacteriales;Lachnospiraceae
W49_bin.15.fa	99.28	0.51	3081309	171453	near	low	Bacteria;Bacteroidetes;Bacteroidia;Bacteroidales;Rikenellaceae
W49_bin.17.fa	99.53	0.08	2313059	171866	near	low	Bacteria;Firmicutes;Negativicutes;Selenomonadales;Selenomonadaceae
W49_bin.2.fa	97.09	0	2491780	60214	near	none	Bacteria;Firmicutes;Clostridia;Eubacteriales
W49_bin.20.fa	96.77	1.08	3788833	30400	near	low	Bacteria;Bacteroidetes;Bacteroidia;Bacteroidales;Odoribacteraceae;Odoribacter;Odoribacter splanchnicus
W49_bin.24.fa	86.6	0.55	2375016	53923	substantial	low	Bacteria;Firmicutes;Negativicutes;Selenomonadales;Selenomonadaceae
W49_bin.26.fa	88.37	0	1758198	32485	substantial	none	Bacteria;Firmicutes;Clostridia;Eubacteriales
W49_bin.28.fa	93.27	0.13	2334023	103214	near	low	Bacteria
W49_bin.29.fa	98.75	0	4276358	87657	near	none	Bacteria;Bacteroidetes;Bacteroidia;Bacteroidales;Bacteroidaceae
W49_bin.30.fa	93.96	0.67	2067741	90598	near	low	Bacteria;Firmicutes;Clostridia;Eubacteriales
W49_bin.4.fa	99.4	0.6	2305408	71442	near	low	Bacteria;Firmicutes;Negativicutes;Acidaminococcales;Acidaminococcaceae
W49_bin.8.fa	96.03	0	2180445	28800	near	none	Bacteria
W49_bin.9.fa	74.16	0	1833533	56207	substantial	none	Bacteria
W50_bin.1.fa	76.46	6.07	1556813	2138	substantial	medium	Bacteria;Firmicutes;Bacilli;Lactobacillales;Streptococcaceae;Streptococcus
W50_bin.10.fa	97.42	1.58	2831003	45780	near	low	Bacteria;Firmicutes;Clostridia;Eubacteriales
W50_bin.2.fa	93.71	0	2877202	27805	near	none	Bacteria;Firmicutes;Clostridia;Eubacteriales;Lachnospiraceae
W50_bin.3.fa	100	0	2102089	216266	perfect	none	Bacteria;Firmicutes;Negativicutes;Veillonellales;Veillonellaceae
W50_bin.5.fa	100	1.12	2180701	89342	perfect	low	Bacteria;Fusobacteria;Fusobacteriia;Fusobacteriales;Fusobacteriaceae
W50_bin.6.fa	96.26	0.63	2596902	32457	near	low	Bacteria;Firmicutes;Clostridia;Eubacteriales
W50_bin.8.fa	98.88	2.25	2676162	45517	near	low	Bacteria;Fusobacteria;Fusobacteriia;Fusobacteriales;Fusobacteriaceae;Fusobacterium
W53_bin.12.fa	100	0.23	2139725	102205	perfect	low	Bacteria;Actinobacteria;Actinomycetia;Bifidobacteriales;Bifidobacteriaceae
W53_bin.15.fa	100	0	2407035	79495	perfect	none	Bacteria;Firmicutes;Clostridia;Eubacteriales;Oscillospiraceae;Faecalibacterium;Faecalibacterium prausnitzii
W53_bin.18.fa	99.97	0	2902000	77084	near	none	Bacteria;Bacteroidetes;Bacteroidia;Bacteroidales;Rikenellaceae
W53_bin.19.fa	98.54	0	3546119	54331	near	none	Bacteria;Bacteroidetes;Bacteroidia;Bacteroidales
W53_bin.4.fa	99.32	0.68	2766114	16836	near	low	Bacteria;Firmicutes;Clostridia;Eubacteriales;Oscillospiraceae
W6_bin.1.fa	98	0	2431156	111571	near	none	Bacteria;Firmicutes;Negativicutes;Veillonellales;Veillonellaceae;Megasphaera;Megasphaera elsdenii
W6_bin.10.fa	97.64	0.34	2498633	43213	near	low	Bacteria;Firmicutes;Clostridia;Eubacteriales;Oscillospiraceae
W6_bin.11.fa	92.88	0.97	2112068	17404	near	low	Bacteria;Firmicutes;Clostridia;Eubacteriales
W6_bin.15.fa	97.38	1.25	2504722	75730	near	low	Bacteria;Proteobacteria;Betaproteobacteria;Burkholderiales;Sutterellaceae
W6_bin.16.fa	70.72	6.72	1809604	2486	substantial	medium	Bacteria;Firmicutes;Clostridia;Eubacteriales;Clostridiaceae
W6_bin.17.fa	98.75	0.62	2458131	136917	near	low	Bacteria;Proteobacteria;Betaproteobacteria;Burkholderiales;Sutterellaceae
W6_bin.24.fa	97.99	0.34	1992504	176127	near	low	Bacteria
W6_bin.8.fa	70.06	0.89	1489496	7172	substantial	low	Bacteria;Firmicutes;Clostridia;Eubacteriales;Oscillospiraceae
W61_bin.12.fa	100	2.25	3205305	56893	perfect	low	Bacteria;Fusobacteria;Fusobacteriia;Fusobacteriales;Fusobacteriaceae;Fusobacterium;Fusobacterium varium
W61_bin.17.fa	97.28	0.34	2967207	28481	near	low	Bacteria;Firmicutes;Clostridia;Eubacteriales;Oscillospiraceae;Ruthenibacterium;Ruthenibacterium lactatiformans
W61_bin.19.fa	97.65	1.34	2768935	71145	near	low	Bacteria
W61_bin.20.fa	97.96	0	2765671	181955	near	none	Bacteria;Verrucomicrobia;Verrucomicrobiae;Verrucomicrobiales;Akkermansiaceae;Akkermansia;Akkermansia muciniphila
W61_bin.25.fa	99.97	0.35	5018311	103724	near	low	Bacteria;Proteobacteria;Gammaproteobacteria;Enterobacterales;Enterobacteriaceae
W62_bin.1.fa	91.9	1.61	2073105	8631	near	low	Bacteria;Firmicutes;Clostridia;Eubacteriales;Lachnospiraceae
W62_bin.10.fa	98.23	0.25	3784062	62943	near	low	Bacteria;Bacteroidetes;Bacteroidia;Bacteroidales
W62_bin.15.fa	87.45	1.33	3088117	9694	substantial	low	Bacteria;Firmicutes;Clostridia;Eubacteriales;Lachnospiraceae
W62_bin.18.fa	87.71	1.01	1845344	8730	substantial	low	Bacteria;Firmicutes;Negativicutes;Selenomonadales;Selenomonadaceae
W62_bin.20.fa	97.99	0	2649748	54653	near	none	Bacteria;Firmicutes;Clostridia;Eubacteriales;Oscillospiraceae
W62_bin.27.fa	75.4	2.45	2130221	4776	substantial	low	Bacteria;Firmicutes;Clostridia;Eubacteriales;Lachnospiraceae;Coprococcus;Coprococcus sp. ART55/1
W62_bin.29.fa	97.97	2.36	3503848	37488	near	low	Bacteria
W62_bin.3.fa	98.64	0	2956496	117985	near	none	Bacteria;Firmicutes;Clostridia;Eubacteriales
W62_bin.30.fa	97.41	0.23	3422071	101080	near	low	Bacteria;Bacteroidetes;Bacteroidia;Bacteroidales
W62_bin.7.fa	99.11	0.67	4694844	80508	near	low	Bacteria;Proteobacteria;Gammaproteobacteria;Enterobacterales;Enterobacteriaceae;Enterobacter;Enterobacter bugandensis
W63_bin.12.fa	95.24	0	2553707	312124	near	none	Bacteria;Firmicutes;Clostridia;Eubacteriales;Oscillospiraceae
W63_bin.13.fa	99.09	1.68	2505479	25288	near	low	Bacteria;Bacteroidetes;Bacteroidia;Bacteroidales;Rikenellaceae
W63_bin.15.fa	82.38	0	2147516	5399	substantial	none	Bacteria;Firmicutes;Clostridia;Eubacteriales
W63_bin.6.fa	75	0.67	1493230	110404	substantial	low	Bacteria;Firmicutes;Clostridia;Eubacteriales;Oscillospiraceae
W63_bin.9.fa	99.41	0.59	2690058	61258	near	low	Bacteria;Proteobacteria;Deltaproteobacteria;Desulfovibrionales;Desulfovibrionaceae;Desulfovibrio;Desulfovibrio piger
W64_bin.13.fa	99.33	0.34	2214198	41630	near	low	Bacteria;Firmicutes;Clostridia;Eubacteriales
W64_bin.15.fa	78.79	1.68	1658398	94584	substantial	low	Bacteria;Firmicutes;Clostridia;Eubacteriales
W64_bin.6.fa	98.79	1.45	3144526	68858	near	low	Bacteria;Firmicutes;Clostridia;Eubacteriales;Lachnospiraceae;Lachnospiraceae bacterium GAM79
W66_bin.11.fa	93.29	0	2116551	160065	near	none	Bacteria;Firmicutes;Clostridia;Eubacteriales
W66_bin.12.fa	98.14	0.62	2351677	47669	near	low	Bacteria;Proteobacteria;Betaproteobacteria;Burkholderiales;Sutterellaceae
W66_bin.15.fa	75.57	0	1601582	6447	substantial	none	Bacteria;Firmicutes;Clostridia;Eubacteriales;Oscillospiraceae
W66_bin.16.fa	99.19	1.36	2320731	61234	near	low	Bacteria;Firmicutes;Clostridia;Eubacteriales;Oscillospiraceae;Faecalibacterium;Faecalibacterium prausnitzii
W66_bin.2.fa	88.91	0	2659382	44118	substantial	none	Bacteria;Firmicutes;Clostridia;Eubacteriales
W66_bin.24.fa	97.76	0	2058021	27722	near	none	Bacteria;Firmicutes;Clostridia;Eubacteriales
W66_bin.4.fa	98.66	0	2815612	259114	near	none	Bacteria;Firmicutes;Clostridia;Eubacteriales;Lachnospiraceae
W66_bin.8.fa	97.99	0	3163600	44666	near	none	Bacteria;Firmicutes;Clostridia;Eubacteriales;Oscillospiraceae
W67_bin.10.fa	98.66	0	2613981	140805	near	none	Bacteria;Firmicutes;Clostridia;Eubacteriales
W67_bin.18.fa	93.08	0.81	2075000	37158	near	low	Bacteria;Actinobacteria;Coriobacteriia;Coriobacteriales;Coriobacteriaceae;Collinsella;Collinsella aerofaciens
W67_bin.19.fa	82.5	0	2136882	21776	substantial	none	Bacteria;Firmicutes;Clostridia;Eubacteriales;Oscillospiraceae;Faecalibacterium;Faecalibacterium prausnitzii
W67_bin.2.fa	88.94	1.37	2329962	28519	substantial	low	Bacteria;Firmicutes;Clostridia;Eubacteriales
W67_bin.20.fa	98.66	0	3006589	99566	near	none	Bacteria;Bacteroidetes;Bacteroidia;Bacteroidales

W67_bin.4.fa	92.65	0.81	1891314	6411	near	low	Bacteria;Firmicutes;Clostridia;Eubacteriales
W67_bin.5.fa	100	0	4318535	247952	perfect	none	Bacteria;Proteobacteria;Gammaproteobacteria;Aeromonadales;Aeromonadaceae
W67_bin.7.fa	95.28	0.91	2156604	48547	near	low	Bacteria;Firmicutes;Clostridia;Eubacteriales;Oscillospiraceae;Faecalibacterium;Faecalibacterium prausnitzii
W67_bin.8.fa	72.79	3.12	1370963	3096	substantial	low	Bacteria;Proteobacteria;Gammaproteobacteria;Pasteurellales;Pasteurellaceae;Haemophilus;Haemophilus parainfluenzae
W70_bin.14.fa	99.37	3.22	2836587	34837	near	low	Bacteria;Firmicutes;Clostridia;Eubacteriales
W70_bin.15.fa	99.49	0.83	2364416	32451	near	low	Bacteria;Actinobacteria;Actinomycetia;Bifidobacteriales;Bifidobacteriaceae;Bifidobacterium;Bifidobacterium dentium
W70_bin.17.fa	71.69	3.49	1470628	4254	substantial	low	Bacteria;Actinobacteria;Coriobacteriia;Coriobacteriales;Coriobacteriaceae;Collinsella;Collinsella aerofaciens
W70_bin.19.fa	97.99	1.01	2383171	36305	near	low	Bacteria
W70_bin.2.fa	99.03	0.24	2945398	113996	near	low	Bacteria;Firmicutes;Clostridia;Eubacteriales;Lachnospiraceae;Lachnospiraceae bacterium
W70_bin.5.fa	89.74	0.89	1799223	6064	substantial	low	Bacteria;Firmicutes;Clostridia;Eubacteriales
W70_bin.6.fa	88.13	0.93	4193489	35922	substantial	low	Bacteria;Bacteroidetes;Bacteroidia;Bacteroidales;Bacteroidaceae;Bacteroides;Bacteroides fragilis
W71_bin.10.fa	98.83	0	2796660	62102	near	none	Bacteria;Firmicutes;Clostridia;Eubacteriales
W71_bin.11.fa	88.82	1.74	2568741	12164	substantial	low	Bacteria;Firmicutes;Clostridia;Eubacteriales
W71_bin.13.fa	100	0	3382069	63426	perfect	none	Bacteria;Firmicutes
W71_bin.14.fa	99.33	0	2915695	129386	near	none	Bacteria;Firmicutes;Clostridia;Eubacteriales
W71_bin.2.fa	99.37	3.48	4911576	39407	near	low	Bacteria;Firmicutes;Clostridia;Eubacteriales
W71_bin.7.fa	96.75	1.06	2890055	106420	near	low	Bacteria;Firmicutes;Clostridia;Eubacteriales;Clostridiaceae
W71_bin.8.fa	99.98	0	3111964	81818	near	none	Bacteria;Firmicutes;Clostridia;Eubacteriales;butyrate-producing bacterium SS3/4
W71_bin.9.fa	98.77	3.66	3338840	47608	near	low	Bacteria;Firmicutes;Clostridia;Eubacteriales;Oscillospiraceae;Faecalibacterium;Faecalibacterium prausnitzii
W9_bin.1.fa	97.5	1.25	2459598	155298	near	low	Bacteria;Proteobacteria;Betaproteobacteria;Burkholderiales;Sutterellaceae
W9_bin.8.fa	98.66	0	2676457	195171	near	none	Bacteria;Firmicutes;Clostridia;Eubacteriales;Lachnospiraceae
W9_bin.9.fa	92.24	6.43	6407517	45332	near	medium	Bacteria;Bacteroidetes;Bacteroidia;Bacteroidales;Bacteroidaceae;Bacteroides

Table S10. Expression levels of inflammatory cytokines

	IL-6	TGF-β	IL-1β	TNF-α	IL-17	IL-12	IL-33	IL-10	Aβ	BDNF
W1	14.33441	98.0501	21.76616	25.92067	106.3996	13.59691	95.44416	221.0166376	210.3374465	10.92914686
W4	13.58405	145.5197	28.08989	18.59142	127.3669	16.74566	116.8283	278.7927665	205.2021981	11.0263942
W7	16.84753	145.8522	31.09977	29.30059	90.27525	11.66669	96.72765	240.4008111	239.2095611	14.21394595
W10	11.11857	120.5622	23.1191	19.22459	98.15482	12.14655	135.3844	268.4419942	222.3014826	9.578489337
W11	10.83404	116.3687	20.14348	26.12109	101.076	14.79117	106.2124	219.08763	217.0023434	9.238123642
W12	16.6649	118.2241	21.21385	25.69746	100.6532	16.08518	158.6394	194.3398744	250.6272942	12.98754892
W14	16.69137	133.5718	21.37654	18.82829	140.0319	15.7482	132.9915	261.4316984	244.6999064	9.316461778
W17	13.42921	97.50311	19.84378	18.4821	127.5207	11.3998	148.4585	235.6959146	202.1155861	15.30527722
W19	17.6998	102.1793	25.11427	27.27355	114.183	13.39473	120.7005	245.3409524	201.7878043	9.540670926
W21	18.07564	90.03839	19.37281	27.39654	139.6668	15.99891	148.5238	206.8548991	200.5313073	14.2949854
W23	18.29665	94.36063	22.74661	29.1776	128.8467	11.51303	139.844	271.0767362	178.2967744	15.16751016
W25	16.20569	118.6317	24.84882	22.44964	148.0076	15.40043	123.9853	222.1928617	248.4693973	12.7876516
W28	15.7822	115.5857	18.37523	22.87782	91.77429	13.19254	96.11853	264.631028	256.4454214	13.13612124
W32	17.04207	123.2113	24.44208	21.14231	107.1106	17.52476	150.8515	194.1516786	233.9650521	14.57592216
W36	17.83743	113.3549	24.17235	25.35127	114.0677	16.16066	105.8426	195.3749517	225.1695735	14.24366041
W38	10.55745	136.9503	24.93445	23.24679	132.9211	11.48337	158.4001	185.8710607	180.400041	11.00478368
W40	15.96218	126.815	27.31067	30.95411	101.9793	13.26263	106.0818	249.5753593	199.4933316	13.9357105
W42	12.30962	121.7527	19.84806	20.78245	117.7	11.50494	156.9861	206.2903115	201.3234467	13.03347127
W45	14.32026	104.0454	20.64013	27.90671	114.3752	10.78245	96.4666	241.5299863	205.2568284	9.743269554
W48	16.03274	141.9532	17.84005	27.66529	120.7558	17.56519	93.07298	180.7897725	238.5813126	11.77735978
W49	13.00984	137.8909	28.84771	28.84508	115.6437	13.10088	93.16	222.1928617	205.3934042	13.67638425
W51	12.92702	143.7881	31.48082	30.42571	143.3183	11.28388	93.48631	203.7496674	250.3541427	14.19773806
W54	12.09349	138.9727	19.02601	20.53647	134.0742	11.42137	109.606	211.3245508	180.4273562	11.32083754
W57	17.24164	106.1879	23.90261	18.59142	107.0146	17.27135	121.2661	196.7864206	249.0430154	10.71304165
W58	13.48271	105.4879	23.93687	30.19796	96.63657	12.08724	115.9364	246.8935683	215.2268585	13.30090146
W59	15.72818	123.8266	23.38027	25.53803	143.3375	15.57027	131.6427	203.4203247	208.5073314	14.6191432
W61	11.17179	98.16939	26.7712	22.72295	139.5899	9.809256	97.27149	192.9754545	247.9504094	12.72011873
W63	19.0129	94.70105	25.25556	20.40893	119.9678	14.46767	99.40338	218.0996017	204.1915375	10.78867847
W65	18.96882	134.4437	18.74344	26.85903	130.7302	10.66114	158.9439	249.8576531	250.2448821	15.337693
W68	16.59912	128.6632	22.63958	18.14957	98.07795	16.96941	144.173	272.1118135	219.1329251	10.15386944
W73	12.57437	151.043	31.03555	22.31298	125.964	13.79641	98.09815	202.6675412	180.2907804	10.82379557
W2	9.973838	106.2012	17.70732	21.77548	74.68906	6.213002	92.68141	164.4167327	213.8064707	14.03836047
W5	12.54519	85.19061	21.33373	15.45292	67.19385	8.997807	92.89895	203.6555695	168.6818413	9.362384133
W8	9.220828	80.24632	19.32572	21.84836	69.74991	13.69396	130.9684	197.3510082	208.9990041	13.7033974
W13	13.16718	91.90457	24.3222	18.39555	115.5668	14.07408	85.89418	135.4345703	175.2101624	14.75420895
W15	9.624462	102.4474	22.5882	16.85591	111.0889	6.838438	74.49511	129.6475476	233.1455976	15.53759032
W18	13.42921	67.19379	24.33504	19.88508	105.9191	7.604056	89.22253	140.6570054	169.0915685	13.28469357
W20	13.41466	64.54467	21.74475	15.08851	96.34829	7.283251	88.93973	126.0718263	259.094991	10.9183416
W22	7.74657	68.77039	19.68108	13.83128	94.98378	8.1594	107.3871	226.2390727	180.7278229	13.61155269
W24	11.13445	80.78257	23.13623	22.92337	105.2849	11.81496	79.6943	218.0996017	208.9990041	12.68230032
W26	8.700735	71.60183	18.68778	14.67854	74.97733	8.941195	103.7977	132.7057304	186.9283621	8.446638335
W29	13.27438	94.16758	17.69876	20.37704	108.898	6.59042	86.65556	164.9342714	221.5366584	16.14808752
W33	12.15743	91.50774	16.91953	21.3154	100.2881	12.06298	105.0159	203.5144226	192.7738044	15.63483766
W37	12.31227	92.16197	22.25425	22.14444	109.0901	6.68747	72.2327	170.6271961	205.7758163	10.5779759
W39	7.690988	103.048	22.57679	17.1742	108.2118	10.30442	123.3192	211.2629478	215.8048545	10.55416538
W41	12.35462	102.2115	16.75262	19.92884	84.39195	6.929521	76.32234	170.7321052	212.2532696	8.402177236
W43	9.215534	73.91847	19.56695	19.03	97.89744	12.2064	129.8725	230.5002474	166.2695903	13.57222973
W46	12.92168	90.36298	17.22914	16.00706	100.0819	10.28872	131.1449	246.7674176	218.3684046	13.2870583
W50	12.67589	70.70907	25.74546	13.23901	106.7491	13.2895	104.5077	213.2734969	196.9520801	14.58353213
W52	13.77792	85.24002	24.1652	20.55042	73.65973	13.4988	102.0052	212.9993311	179.5413026	9.260332017
W55	8.430742	94.35104	19.51401	21.92326	80.93485	13.26857	131.4419	216.9747351	225.0977235	14.58353213
W60	12.03605	104.0772	24.05116	18.06409	104.4887	14.29412	126.967	189.7409333	210.4908289	13.57487021
W62	10.39302	67.1983	22.45053	21.43584	71.34233	6.701911	117.2961	170.1837736	197.1390057	11.29877968
W64	10.40772	63.83603	21.34272	17.73765	80.327	6.377502	104.5714	147.2909301	207.6602424	13.53526306
W67	13.19685	110.229	16.20685	17.70188	83.8031	11.59682	110.552	222.2295794	202.2661058	11.76614398
W69	8.581687	84.90061	20.23083	18.04173	93.0347	13.4674	115.4935	176.672364	215.1906707	10.30332014
W74	11.96525	80.08524	16.44715	15.80136	85.64562	11.62298	114.2422	131.8462573	175.2954228	9.149432014
W3	5.335351	45.62547	12.69606	17.10265	70.84847	6.435059	62.8977	164.3349034	142.503345	16.48731551
W6	3.244393	28.40086	19.93351	10.74374	68.72102	7.779786	100.86	264.1312509	164.6139642	19.72453939
W9	5.503422	30.50299	13.84053	13.39105	68.68302	7.7013	100.2025	196.3666065	128.297005	18.19042269
W16	4.461913	45.60402	17.36354	15.80136	79.64318	9.726238	104.6774	209.0696215	177.5919364	16.49787742
W27	7.315145	60.86594	12.69606	13.85165	61.48389	9.427992	101.9628	290.9081097	144.0788601	19.5898751
W30	4.459266	65.28471	14.95242	11.25353	64.12421	8.185297	73.65014	277.1998203	168.4325857	20.65134656
W31	3.973581	53.80878	14.20709	16.27984	86.27245	9.73932	90.23477	297.7622545	171.4768014	21.25337514
W34	4.322957	36.78794	19.22891	12.74711	78.12357	8.067568	61.96455	158.3489503	175.1084973	13.91813212
W35	6.106888	28.00403	12.81825	13.47154	69.34786	7.078644	79.65199	224.7884601	148.3781472	22.19074421
W44	6.666683	26.71701	17.69752	14.42851	66.70754	10.41168	81.47588	186.0396951	173.6130931	22.60529898
W47	8.524247	59.36007	18.06	17.92547	70.58253	9.503861	96.02454	207.6530982	155.7750573	18.12969174
W53	5.891398	53.00676	15.68146	15.73875	61.36992	6.173439	73.31081	257.7340492	127.3089701	20.21038702
W56	5.568137	64.62092	16.42272	15.71639	84.9428	6.994926	83.46943	227.3016465	158.4187183	21.86596563
